# Automatically visualise and analyse data on pathways using PathVisioRPC from any programming environment

**DOI:** 10.1186/s12859-015-0708-8

**Published:** 2015-08-23

**Authors:** Anwesha Bohler, Lars M. T. Eijssen, Martijn P. van Iersel, Christ Leemans, Egon L. Willighagen, Martina Kutmon, Magali Jaillard, Chris T. Evelo

**Affiliations:** Department of Bioinformatics - BiGCaT, Maastricht University, P.O. Box 616, UNS 50 Box 19, 6200 MD Maastricht, The Netherlands; Netherlands Consortium for Systems Biology (NCSB), Amsterdam, The Netherlands; General Bioinformatics, Reading, Berkshire RG4 7RT UK; Maastricht Centre for Systems Biology (MaCSBio), Maastricht University, P.O. Box 616, UNS 50 Box 19, 6200 MD Maastricht, The Netherlands; Bioinformatics Research Department, BioMérieux S.A, 69280 Marcy l’Etoile, France

**Keywords:** Automation, Biological pathways, Data visualisation, Multi-omics, Pathway analysis, Pathway building, R package, Workflow integration

## Abstract

**Background:**

Biological pathways are descriptive diagrams of biological processes widely used for functional analysis of differentially expressed genes or proteins. Primary data analysis, such as quality control, normalisation, and statistical analysis, is often performed in scripting languages like R, Perl, and Python. Subsequent pathway analysis is usually performed using dedicated external applications. Workflows involving manual use of multiple environments are time consuming and error prone. Therefore, tools are needed that enable pathway analysis directly within the same scripting languages used for primary data analyses. Existing tools have limited capability in terms of available pathway content, pathway editing and visualisation options, and export file formats. Consequently, making the full-fledged pathway analysis tool PathVisio available from various scripting languages will benefit researchers.

**Results:**

We developed PathVisioRPC, an XMLRPC interface for the pathway analysis software PathVisio. PathVisioRPC enables creating and editing biological pathways, visualising data on pathways, performing pathway statistics, and exporting results in several image formats in multiple programming environments.

We demonstrate PathVisioRPC functionalities using examples in Python. Subsequently, we analyse a publicly available NCBI GEO gene expression dataset studying tumour bearing mice treated with cyclophosphamide in R. The R scripts demonstrate how calls to existing R packages for data processing and calls to PathVisioRPC can directly work together. To further support R users, we have created RPathVisio simplifying the use of PathVisioRPC in this environment. We have also created a pathway module for the microarray data analysis portal ArrayAnalysis.org that calls the PathVisioRPC interface to perform pathway analysis. This module allows users to use PathVisio functionality online without having to download and install the software and exemplifies how the PathVisioRPC interface can be used by data analysis pipelines for functional analysis of processed genomics data.

**Conclusions:**

PathVisioRPC enables data visualisation and pathway analysis directly from within various analytical environments used for preliminary analyses. It supports the use of existing pathways from WikiPathways or pathways created using the RPC itself. It also enables automation of tasks performed using PathVisio, making it useful to PathVisio users performing repeated visualisation and analysis tasks. PathVisioRPC is freely available for academic and commercial use at http://projects.bigcat.unimaas.nl/pathvisiorpc.

**Electronic supplementary material:**

The online version of this article (doi:10.1186/s12859-015-0708-8) contains supplementary material, which is available to authorized users.

## Background

Biological pathways are descriptive diagrams used to depict complex cellular processes such as metabolism, gene regulation, and signal transduction. Disturbances in such processes can cause disease. Pathways thus can help understand the functions of individual genes and proteins in terms of the systems and processes that contribute to normal physiology and to disease. Pathway analysis integrates and visualises global high throughput measurements and is a widely applied method among researchers to gain functional understanding from biological quantifications, such as gene expression and (relative) metabolite and protein abundances [1].

Pathways can be obtained from pathway databases. The Pathguide website [2, 3], at the time of writing, listed more than 570 pathway-related databases. A recent review found that among them Reactome [4, 5], KEGG [6], WikiPathways [7], Nature Pathway Interaction Database [8], and Pathway Commons [9] are most used [10]. Similarly, a number of tools exist for working with pathways, such as Ingenuity Pathway Analysis software [11], Pathway Studio [12], Metacore, PathVisio [13], Vanted [14], Reactome [4, 5], and Pathway Tools [15].

PathVisio is an open source tool for pathway editing and analysis that has seen rapid adoption by the scientific community since its release in 2008, counting 171 citations. A selection of the citing papers demonstrates the visualisation possibilities [16–20]. In addition, PathVisio is extendable using plugins such as PathVisio-Faceted Search, PathVisio-MIM, and PathVisio-Validator, to name a few [21–23]. In PathVisio, pathways are drawn as graphical diagrams containing nodes and edges, referred to as datanodes and interactions respectively. Datanodes represent biological entities such as genes, transcripts, proteins, and metabolites. Interactions can for instance indicate activation, inhibition, conversion, and catalysis. Both datanodes and interactions can be annotated using external database identifiers and all entities on a pathway can be annotated with literature references. The BridgeDb identifier mapping framework [24] is integrated into PathVisio for resolving identifiers from different databases. This allows mapping experimental measurements, such as gene expression, metabolite concentration, and fluxes, to pathway elements irrespective of the identifier formats used in the datasets or the pathways [25]. Pathways drawn in PathVisio are stored in GPML (Graphical Pathway Mark-up Language) files. The GPML file format is also used by WikiPathways to store pathway files. Users can create their own pathways, use pathways from WikiPathways, or pathways converted to GPML from other sources, such as KEGG, Reactome, and BioPAX [26]. Data can be visually represented on the pathways using colours, colour gradients, and textual labels. Furthermore, the core PathVisio program allows calculation of pathway statistics using an over-representation analysis algorithm (Z score) [27].

However, when researchers perform the same analysis using multiple sets of data and/or pathways using the graphical user interface of PathVisio they have to click the same set of buttons repeatedly for uploading the data, creating visualisations, performing pathway statistics, and exporting results, making the analyses error prone and time consuming on the user side. In this paper we describe the newly developed XMLRPC interface PathVisioRPC, which enables direct programmatic access to PathVisio from scripting languages. It enables automation of these steps by calling the defined functions, e.g. from inside a loop. An additional advantage is that a script written to automate PathVisio usage can be stored to enable later reference for validation and sharing purposes.

RPC stands for Remote Procedure Call. An RPC is initiated by a client that sends a request to a local or remote server to execute a task with supplied parameters. The server sends a response back to the client. XMLRPC uses XML to encode its calls and uses HTTP as a transport mechanism [28]. The PathVisioRPC interface wraps PathVisio functionality into XMLRPC functions that can be called from different programming languages to execute tasks. The interface serves as a communication channel between PathVisio and the script. Implementing the interface using XMLRPC makes the layer independent of the client’s programming language or operating system. Additionally it allows the server to be run on one machine and to be called by users from other machines. Among the code bases most used for genomics data analysis are Bioconductor for R [29], BioPerl [30], and Biopython [31]. Each of these offers numerous data retrieval, normalisation, and statistical analysis packages that are often used prior to performing pathway analysis in PathVisio. XMLRPC functionality can be accessed from R and Perl using available add-on libraries; for Python no such library is necessary. The Bioconductor package repository in R provides an important toolset for bioinformatics data analysis. Therefore, we developed an R package RPathVisio for simplifying PathVisioRPC use in R [32]. For identifier mapping, RPathVisio uses the BridgeDbR package [33]. In addition, a module for pathway analysis was developed for ArrayAnalysis.org, an online microarray data analysis platform. This pathway module uses PathVisioRPC in the background [34].

## Implementation

**Fig. 1:**
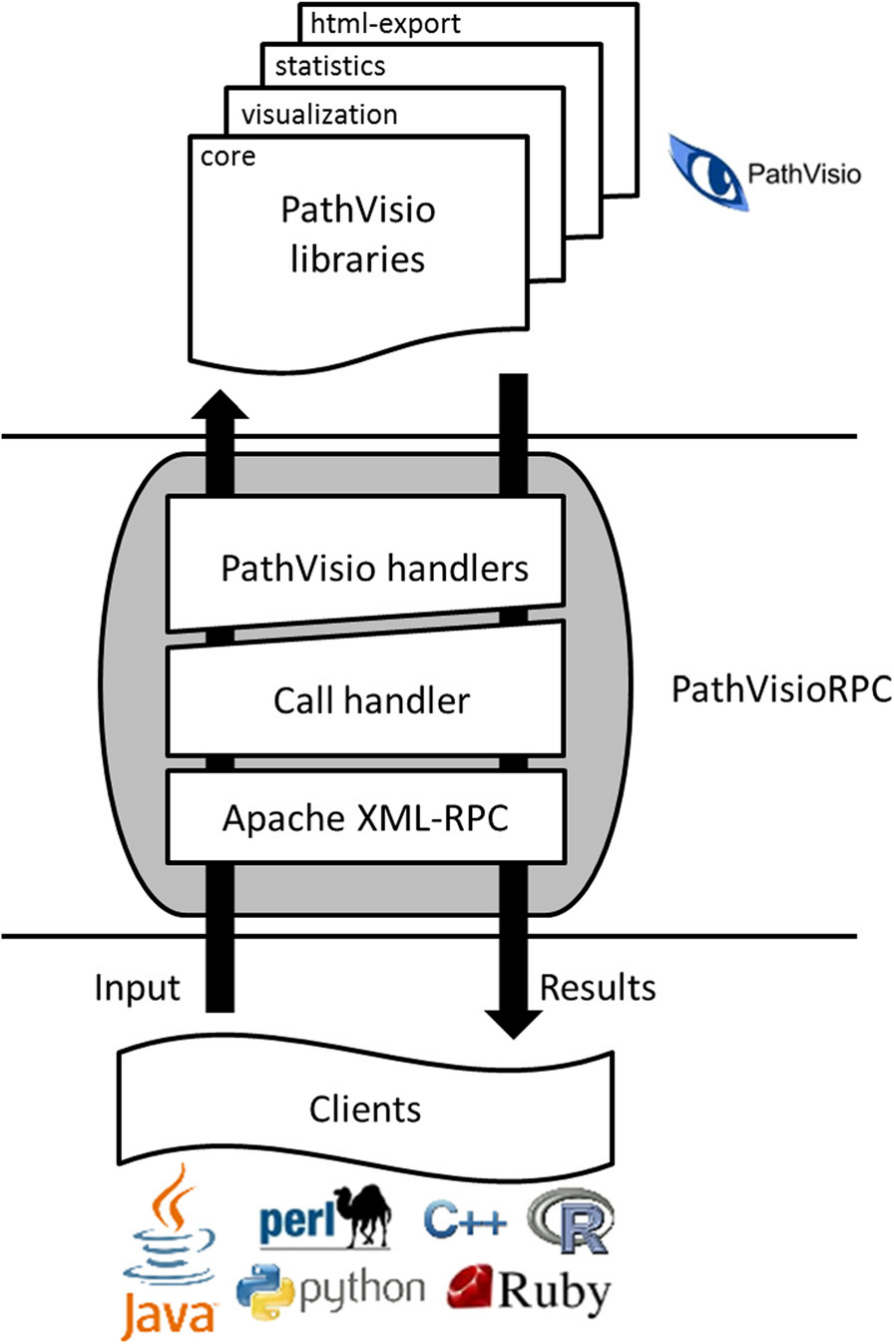
Interaction diagram of PathVisioRPC

The PathVisioRPC interface (Fig. 1) allows users to access functionality of the pathway analysis program PathVisio from R, Perl, Python, Java, C, C++, PHP, and many other programming languages. It allows access to all core functionalities of PathVisio including creation and modification of pathway diagrams, data visualisation, overrepresentation statistics, and various data exporters. An exhaustive list of Java API function calls for the program is available from Additional file 1: Table S1. The interface is available both as a PathVisio plugin [35] and as a standalone program. The current versions of both have been provided as Additional file 2 and the latest versions can be obtained from the project website [36]. PathVisio’s plugin manager provides an easy two-click installation of the PathVisioRPC plugin that automatically installs the HTML export plugin [37] as well. The latter provides the underlying functionality to create HTML exports of pathways with or without data visualisation. The standalone version is an executable Java archive (jar) file containing PathVisioRPC, the core PathVisio program, and the HTML export plugin.

PathVisioRPC is a client–server application that handles client requests to execute tasks after the server is launched. Running the PathVisioRPC.jar file launches the server on a port that is passed as an argument or on the default port 7777 of the local computer. The PathVisioRPC plugin for PathVisio adds a new “XMLRPC server setup” sub-menu item to the PathVisio “Plugins” menu. This is used to define the port for the server and to start or stop the server. A detailed API documentation along with code snippets from different languages for calling the implemented functions is available from the online documentation [38].

## Results and discussion

### Example use of PathVisioRPC functionality

The following three examples use PathVisioRPC in Python to illustrate some of the functionalities implemented in PathVisioRPC. The scripts and the data for the examples are provided in Additional file 3.

#### Using same set of functions on multiple files

The first example demonstrates how the same functionalities can be applied to multiple files. The *example1.py* script loops over three files with gene expression data to create a pathway with the genes in each one of them and visualise the measurements. The script opens and reads each file, creates a new empty pathway, adds the genes in the file as nodes to the pathway, and visualises the gene expression data on the nodes [39]. In the example we generate a separate pathway for each list, it would also be possible to create a single pathway containing all the genes from the three lists.

#### Using different functionalities on different files

The second example demonstrates how files of different types can be combined/processed together. A pathway is created with a list of genes in a file and the data from a second file is displayed on the pathway created. The same data is then also visualised on a pathway from WikiPathways. The *example2.py* script reads a file with a list of genes, creates a new empty pathway, and then adds the genes as nodes to the newly created pathway. Subsequently, the script visualises the data from the second file on the pathway. The gene expression data is also visualised on the Statin pathway [40] from WikiPathways by passing its WikiPathways identifier (WP1) [39] to a dedicated function that has been implemented in PathVisioRPC for visualising data directly on WikiPathways pathways without first having to download those first. This requires an active internet connection. Alternatively, a previously downloaded pathway can be called upon using the local file path.

#### Looping over multiple files with different settings

The third example shows how multiple data files can be processed with different settings based on a configuration file. The script reads a settings file that contains the names of data files, associated species (human or mouse), and numbers of the data columns to be visualised. For each data file name present, the script loads the file, uses the correct species identifier mapping database, and visualises the data in the desired column(s) using a gradient on a WikiPathways pathway (cholesterol biosynthesis) from the correct species.

### Multi-tissue time-series gene expression data analysis in R, for tumour-bearing mice treated with cyclophosphamide

As an illustration of the use of PathVisioRPC functionality to analyse a real genomics dataset we performed data visualisation and pathway analysis with gene expression data from a study by Moschella et al. [41]. They performed gene expression profiling at several time points to study the effects of cyclophosphamide in bone marrow, spleen, and PBMCs of tumour-bearing mice, to gain insights into the core mechanisms of chemoimmunotherapy. We retrieved and analysed the microarray data sets for each of the three tissues and all time points in R as further described below. In short, we automated the entire analysis starting from retrieval of the data sets and the pathway collection, moving on to the pre-processing of the data sets, followed by differential expression analysis of the genes, pathway analysis, and visualisation of the data sets on WikiPathways pathways, and finally Gene Ontology Analysis and visualisation. For quality control and normalisation we used arrayQC, which is included in Additional file 4. All other scripts used and provided in Additional file 4 were custom made using existing R libraries.

#### Data retrieval and pre-processing of data

The gene expression datasets ([41] accession GSE27421, GSE27422, and GSE27423) were retrieved from the NCBI GEO database [42] using GEOquery [43]. The dataset contains 40 samples for bone marrow, 30 samples for peripheral blood leukocytes, and 38 samples for spleen tissue type, where each sample has been hybridised to microarray slides spotted with 13,443 70-mer oligonucleotides (Operon version 1.1; CRIBI Microarray Service, University of Padua, Italy). Next, the curated collection of mouse (*Mus musculus*) pathways was downloaded from WikiPathways using the WikiPathways web service [44].

The arrayQC workflow was used for quality control and normalisation of the retrieved datasets as they were obtained using the Genepix scanning platform. This workflow is similar to the published affyQC workflow for Affymetrix arrays [34]. The up-to-date version of the R scripts for the arrayQC workflow can be downloaded from the tab “Download Sources” at the ArrayAnalysis.org website [45]. Based on the quality control results, all arrays were of sufficient quality for inclusion in further analysis (see Additional file 5: Figure S1). The data were normalised using Lowess [46]. Based on the different quality indicators and flags from the quality control report of the arrayQC workflow, spots of insufficient quality were discarded. Furthermore, since each probe was spotted twice on the slides, the intensities of duplicate spots of sufficient quality were averaged. The GEOquery package was used to obtain the platform annotation file (GPL13209) to annotate the normalised data with NCBI gene identifiers.

#### Differential expression analysis

Differential expression analysis of the pre-processed normalised dataset was performed using the R package limma [47]. A 3x4 factorial design comparing every tissue with every treatment including the proper pairing arrangement regarding samples from the same individuals was used for fitting the data. Student’s *t*-test comparisons were made between day 1, day 2, and day 5 vs. the control state (day 0), for each cell type: PBMCs, bone marrow cells, and splenocytes. The lists of statistical results for each of the nine comparisons were collated into a single file, which was used for further analysis (see Additional file 6: Table S2).

#### Pathway over-representation analysis and data visualisation on WikiPathways pathways using PathVisioRPC

Pathway over-representation analysis was performed with the table of gene level statistics calculated in the previous step and the curated collection of WikiPathways mouse pathways, in order to identify the biological processes that were most affected in each tissue at each time point. Nine Z score tests were conducted, one for each cell type (PBMCs, bone marrow cells, and splenocytes) at each time point (day 1, day 2, and day 5). The Z score for over-representation analysis is calculated as a score for each pathway in the pathway collection, by subtracting the expected number of genes in a pathway meeting the criterion from the observed number of genes and dividing by the standard deviation of the observed number of genes [27]. The criterion for the calculation of the Z-score was the P-value for the gene level statistics being < 0.05.

The dataset was then visualised on the pathways. Log2 fold-changes of the genes were visualised using colour gradients (with blue, white, and red corresponding to the values −1, 0, 1) and significant genes were visualised using colour rules (*P*-value ≤ 0.05 represented by green and white otherwise) in each tissue for each time point. Genes in the pathways absent in the dataset were coloured light grey.

Next, the results of the analyses were exported as hyperlinked HTML pages containing an overview of the settings used to calculate the Z scores, a clickable list of pathways ranked according to their Z scores, a legend, and a frame to display the pathway when clicked [48]. The pathway images are PNG files embedded in the HTML and use image maps to link each component to its corresponding back page containing the data uploaded for that gene as well as links to relevant database entries. The legend contains the colour codes for the gene visualisation. Clicking on a gene in the pathway shows the gene identification and data uploaded for it in a new tab.

#### Gene Ontology (GO) enrichment analysis and data visualisation on GO terms using PathVisioRPC

Furthermore, Gene Ontology (GO) enrichment analysis was performed using the topGO Bioconductor package [49] separately for the three cell types PBMCs, bone marrow cells, and splenocytes. The significant genes for each tissue type (all time points combined) were selected as the numerator list and the entire dataset was taken as the denominator list. A list of the top 50 terms for each cell type was obtained as a result (see Additional file 7: Table S3). PathVisioRPC was then used to create one pathway per cell type, with the highly enriched GO terms as nodes. Subsequently, the gene expression data for each cell type was visualised on the respective pathway. The log2 fold-changes of the genes were visualised in rows on the GO terms they belong to, using the same colour gradient as used before (blue, white, and red, corresponding to the values −1, 0, 1). For each cell type, the pathway created using enriched GO terms along with the visualised results, was exported in HTML format [50]. Clicking on the GO nodes of the pathway opens a new tab in the browser, containing columns of data for each of the genes in the dataset belonging to that GO term.

#### Interpreting the pathway and Gene Ontology (GO) analysis results

Overall, the Z score indicates whether the number of genes meeting the criterion is higher or lower than what is expected based on the complete dataset. A positive score indicates that in that pathway more genes are changed than expected; a negative score means fewer genes are changed than expected. Thus, it can be inferred that the highest-ranking pathways are potentially the most interesting ones for the given conditions and need to be evaluated further, since they were detected to contain an overabundance of differentially expressed genes. Subsequently, the involvement of the pathways in cellular functions has to be determined for a biological interpretation of the results.

**Fig. 2:**
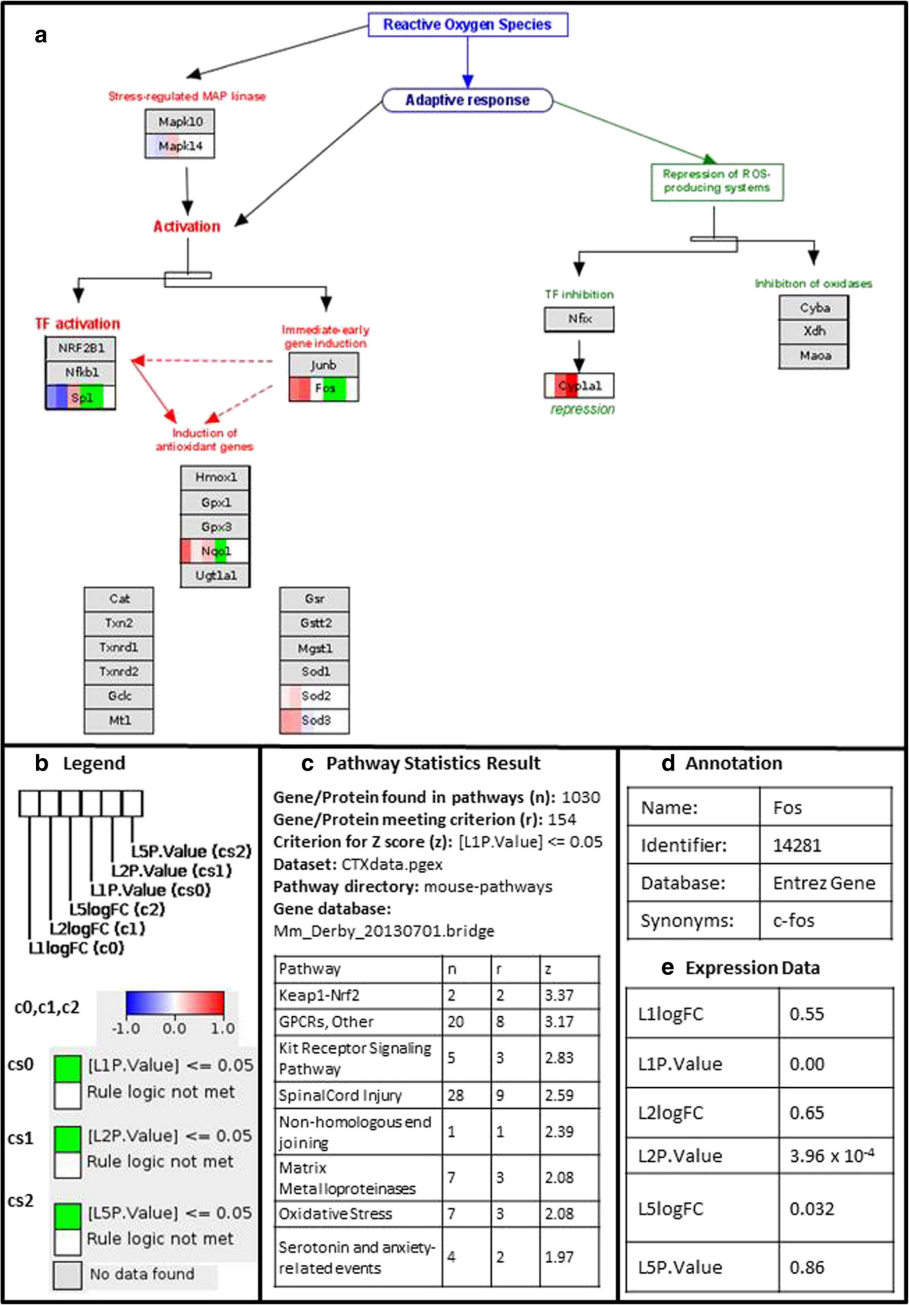
Pathway Statistics Results for PBMCs. **a** Oxidative stress pathway [49] for PBMCs showing the logFC and P-value for day 1, day 2 and day 5, (**b**) Legend showing the colour gradients and rules used to visualise logFC and P-value of the genes, every data node is divided into 6 columns, 3 for the logFCs and 3 for the P-values of PBMCs (L) at the three time points, day 1 (1), day 2 (2) and day 5 (5) (**c**) Parameters used to calculate the Z score and ranked list of pathways, and (**d**) Back page showing the annotation for the gene *Fos*, (**e**) Back page showing the gene expression data for PBMCs for the gene *Fos* for day 1, day 2, and day 5

We used the Oxidative stress pathway [51] to illustrate the visualisation produced using PathVisioRPC. Every data node on the pathway is divided into 6 columns; the first 3 columns are used to visualise the log2 fold change for the transcript at each time point (day 1, day 2, and day 5) and the last 3 columns are used to visualise the *P*-value for that time point (day 1, day 2, and day 5). In PBMCs, the transcripts for *Fos* and *Nqo1* are significantly upregulated for day 1 and/or day 2. Moreover, the transcript for *Sp1* is down regulated for day 1 and day 2 (Fig. 2). In splenocytes, the transcripts for *Mapk14*, *Sp1*, and *Cyp1a1* are all significantly down regulated for either day 1 or both day 1 and day 2 (see Additional file 8: Figure S2). However, in bone marrow cells, the transcripts for *Mapk14* and *Sp1* are both significantly upregulated for day 1 and 2, while the gene *Fos* is significantly down regulated (see Additional file 9: Figure S3). In all the three cell types, the expressions of the genes seem to return to baseline levels by day 5 as also observed in the original publication.

In addition to the pathway analysis, a GO term enrichment analysis can shed additional light on the processes that are targeted by the experimental conditions. GO terms describe functions that can span over several pathways or be involved in multiple cellular processes or are not known in large enough detail to be represented in pathway collections. Thus, the discovery of GO terms over-represented in the data sets can lead to information that is potentially not available from pathways. From the GO term enrichment analysis it can be deduced which functions are highly involved or targeted by the experimental conditions. In addition, the visualisation of the gene expression data on the enriched GO terms can allow determining whether these functions are impacted positively or negatively by the treatment, or whether they display a mixed variation. PathVisioRPC can be used for creating such a visualisation. For the dataset presented here, transcripts with decreased abundance are visualised in blue and transcripts with increased abundance are visualised in red.

**Fig. 3:**
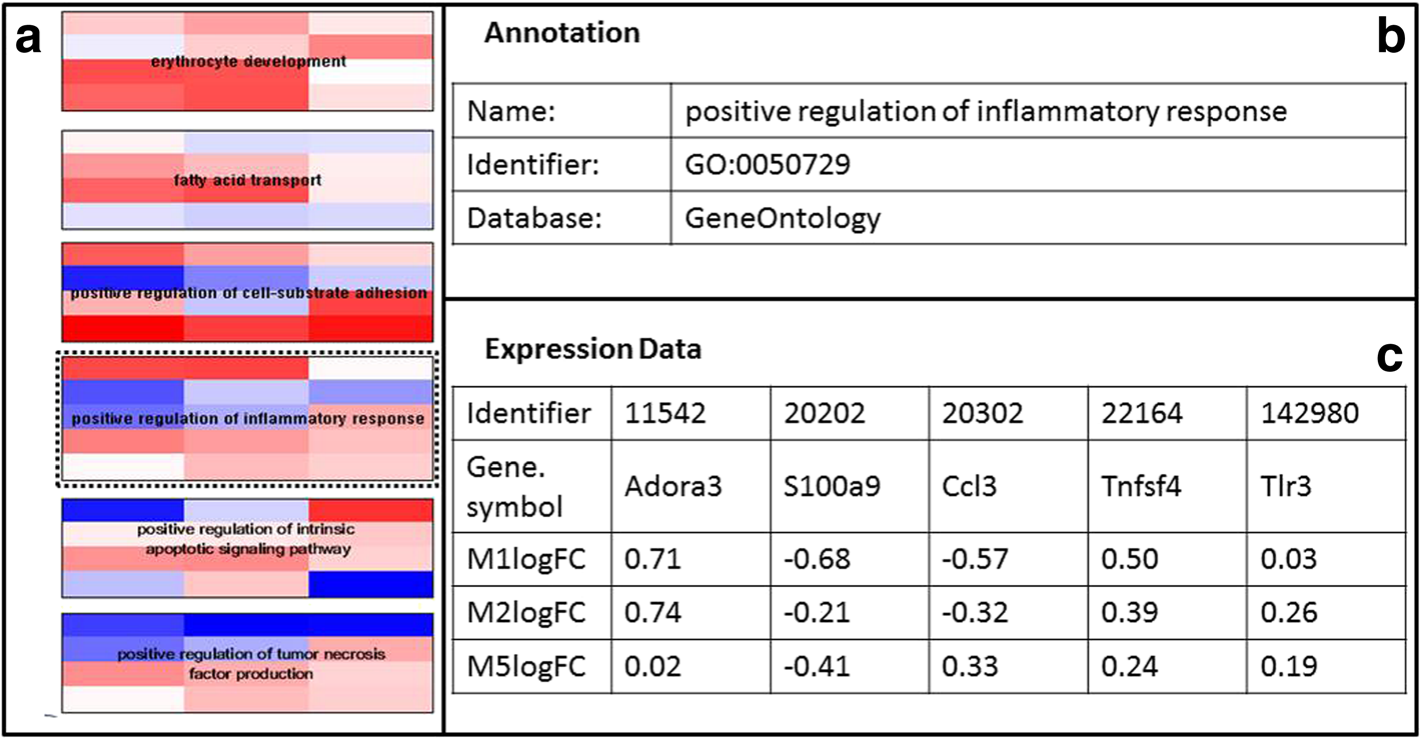
Gene Ontology Enrichment analysis for Bone Marrow Cells. **a** Gene Expression Data visualised on Gene Ontology Terms, (**b**) Back page showing the Gene Ontology Annotation for GO term GO:0050729, positive regulation of inflammatory response, (**c**) Back page showing the Gene expression data for the five genes (Adora3, S100a9,Ccl3, Tnfsf4, and Tlr3) found in the dataset which map to the GO class positive regulation of inflammatory response

In bone marrow cells, processes related to defence response, developmental processes, and response to external/chemical stimuli are highly affected, as was also observed in the original publication [50]. For instance, the GO term “positive regulation of inflammatory response” is highly stimulated and the transcripts in the dataset related to that term are mostly upregulated (Fig. 3). For splenocytes, transcripts increased in abundance are mostly involved in cell cycle arrest and regulation, whereas the original publication reported these to be down regulated. Even though the original publication reports that transcripts involved in immune response are mostly upregulated, it is clear from the present analysis that these transcripts show a 50/50 ratio between up- and down-regulation. Whether that is caused by real differences in remaining data after quality control and statistical evaluation or just differences in how data is communicated is not clear since the processed data from the original publication is not available. The GO terms RNA processing and proteolysis contain mostly transcripts down regulated under the experimental conditions [50]. In PBMCs, similar to the original publication, only few GO terms were enriched for up-regulated transcripts. For down-regulated transcripts, regulation of gene expression is the most notable term that is enriched. Other enriched GO terms for PBMCs contain approximately as many up- and down-regulated transcripts [50].

### RPathVisio: a package for performing pathway analysis and data visualisation in the R statistical environment

RPathVisio (see Additional file 10, submitted to the Bioconductor repository) makes use of the PathVisioRPC interface for providing direct access to PathVisio functionality and the wealth of WikiPathways pathways within the R statistical environment. RPathVisio not only hides the technicalities of an XMLRPC call, but also verifies the user input to check whether the correct system code has been used or not. The package depends on the XMLRPC and BridgeDbR packages. The BridgeDbR package is also new and has been submitted to the Bioconductor repository (see Additional file 10). It facilitates identifier mapping in R. When called from RPathVisio, it downloads the necessary identifier mapping databases from the BridgeDb website [52] and uses them to check whether known database identifiers have been used to annotate the data.

RPathVisio provides simple R commands to perform the PathVisioRPC API calls. For instance, the PathVisioRPC call in R for creating a pathway using the pure XMLRPC formalism is xml.rpc(server_address,“PathVisio.createPathway”,“Glycolysis”), where server_address refers to the address at which the PathVisioRPC server has been launched. However, when using RPathVisio the call is simplified to createPathway(name = “Glycolysis”).

Several other packages are available in R to access pathways as gene sets and perform pathway analyses. Notable among them are PathView [53], KEGGGraph [54], ReactomePA [55] and sigPathway [56], which provide access to the KEGG [57], Reactome [4, 5], BioCarta [58], and BioCyc [59] pathway collections. The KEGGGraph and PathView packages offer pathway visualisation on KEGG pathways. The RCytoscape package offers data visualisation styles as well, but the data can be visualised solely on networks created in Cytoscape. RPathVisio provides users access to the entire WikiPathways collection, including pathways from the Reactome [4, 5], NetPath [60], and WormBase [61] collections. Pathways from other pathway resources can also be used as long as these can be exported in BioPAX format, which can then be transformed into GPML used by WikiPathways. Results from RPathVisio can be exported as GPML files, images (PNG, SVG, PDF), and HTML files. The HTML export report is navigable as a mini self-contained website containing a list of all pathways used for the analysis sorted by their Z scores, the data visualised pathway images, and the corresponding measurements per gene, protein, or metabolite. A comparison of RPathVisio and these other packages is given in Table 1. There are alternatives available for BridgeDbR as an identifier-mapping tool. However, RPathVisio uses BridgeDbR to verify whether the annotations used in the data are suitable for use with PathVisio, which uses the same BridgeDb approach.

**Table 1:** Comparison of RPathVisio with other similar packages available in R

Software	SigPathway	ReactomePA	KEGGGraph	PathView	RPathVisio
Feature					
Pathway sources available	GO, KEGG, BioCarta, BioCyc, SuperArray	Reactome	KEGG	KEGG	WikiPathways, Reactome, NetPath, WormBase
Pathway building	—	—	+	—	+
Multi-omics support	—	—	+	+	+
Plots	—	+	—	+	—
Pathway visualisation	—	—	+	+	+
Multiple data visualisation	—	—	+	+	+
Pathway statistics	Gene set statistics	EA, GSEA FM detection	—	—	EA
Export	Text, HTML	Text	Images	Images	Text, GPML, Images, HTML

*GO* Gene Ontology, *EA* Enrichment Analysis, *GSEA* Gene Set Enrichment Analysis, *FM* Functional Module

+Present

—Absent

### Pathway module of ArrayAnalysis.org

As an example of workflow integration, we have developed a module for pathway analysis for ArrayAnalysis.org, a web-based platform for microarray data analysis. It has a modular setup with quality control and normalisation modules for several types of microarray platforms. The workflow has been extended with modules for statistical analysis and pathway analysis that accept data either from the built-in data normalisation modules or from user uploads. PathVisioRPC powers the pathway module of ArrayAnalysis.org. This workflow is available at the ArrayAnalysis.org website [45].

#### Online form description

**Fig. 4:**
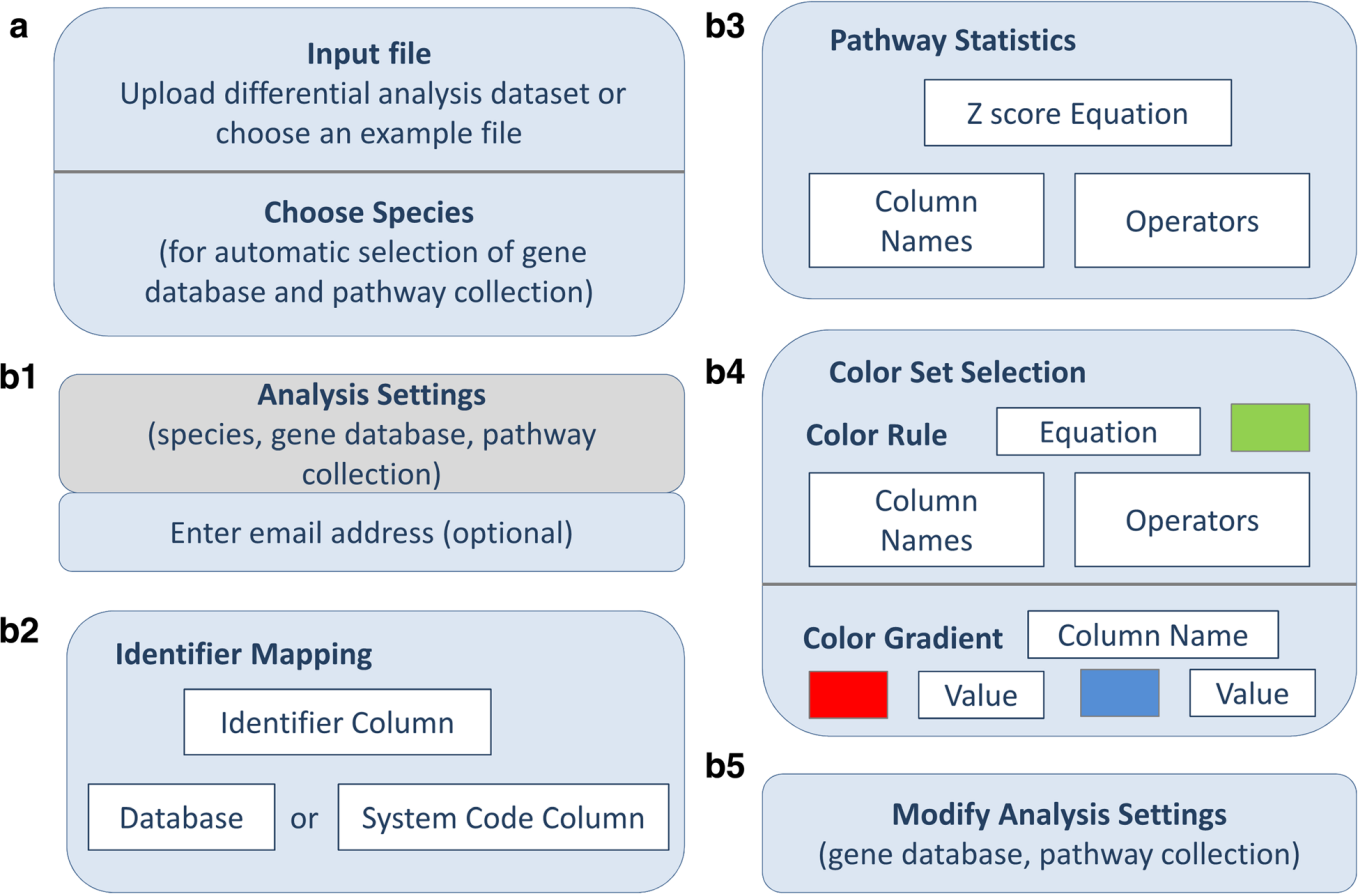
Schematic representation of the input wizard of the Pathway Analysis module of ArrayAnalysis.org. **a** Allows upload of a dataset (e.g. differential analysis data, metabolite concentration data) and to select species; (**b1**) Shows the selected species, gene identifier mapping database, pathway collection, and asks for an optional email address; (**b2**) Selects Identifier Column and a Database or a System Code Column; (**b3**) Specifies criterion for Z score calculation; (**b4**) Chooses colour rules and/or gradients; and (**b5**) Modifies gene database and pathway collection

The online pathway module (Fig. 4) in ArrayAnalysis.org accepts delimited text files of different kinds, for example a statistics results file such as produced by limma [47]. The genes, proteins, and metabolites should be annotated using database identifiers in order to be recognised by the internal BridgeDB identifier mapping support of PathVisio. When identifiers from a single database are used, the database can be selected from a drop down list, otherwise the file should have a column containing the system codes of the databases [62]. The identifier mapping database and pathway collections are determined based on the species selected by the user. The pathway collection used by default is the curated analysis collection of pathways from WikiPathways for the species selected and the default identifier-mapping file used is the gene/protein database from BridgeDb for that species. The user can choose a different pathway collection or identifier-mapping file at the end of the form.

In the next form, the user can specify the colour gradients and/or colour rules to visualise the data on the pathways. To choose a colour gradient for a variable, the user selects the variable and then two or three colours and corresponding values to create a gradient. In order to choose a colour rule, the user enters a criterion and a colour to be used if the criterion is met. For performing pathway statistics, a criterion for calculating the Z score needs to be set. After setting the options, on clicking Run, the pathway module is launched. The form inputs are converted into R commands which in turn call the PathVisioRPC server for executing the tasks. After completion of the run, results display on screen.

## Conclusions

PathVisioRPC enables automated calls to PathVisio to support users in performing repeated analyses and building workflows. We have demonstrated the advantages of using PathVisioRPC with scripts written in Python and R, commonly used scripting languages for bioinformatic analysis. PathVisioRPC simplifies the task of creating pathways and visualising data using multiple files as demonstrated by our example use cases in Python. It also allows users to perform multiple pathway statistics comparisons as shown by the biological analysis in R that compares the transcriptomics profile of three different tissues in three different time-points. We have also shown that the functionality provided by the interface can be easily combined with existing analysis packages from Bioconductor, as existing R packages can be used for statistical analysis and GO analysis and then RPathVisio can be used for pathway building, pathway analysis, data visualisation on pathways, and export. The same approach could be followed in other programming languages, example code snippets are available in R, Perl, and Python from the project website [36]. The pathway analysis and data visualisation results are presented as hyperlinked HTML pages. This makes it possible to manoeuvre through the results and explore the interesting pathways. In the case of a pathway diagram created with enriched GO terms, clicking on the nodes displays the data for all the genes annotated with that GO term. This gives a quick overview of how each enriched GO term is affected.

Writing scripts to create pathways, visualise data, perform pathway statistics comparisons, and export data visualised pathway images makes these tasks less error- prone and time- consuming for the user as compared to doing this manually. Using a script also allows the user to retain method provenance. This ensures reproducibility, reference at a later time, and sharing the method used with others. Therefore, PathVisioRPC simplifies workflows involving the visualisation of experimental data on multiple pathway sets from WikiPathways and allows repeated over-representation analyses in various programming languages and for different gene classification approaches such as GO. This allows researchers to integrate PathVisio as a visualisation and pathway analysis tool in workflows, facilitating automated downstream analysis of (multiple) datasets. These pathways can be exported with or without included data visualisation in various image formats for publication, uploaded to WikiPathways for community curation, or used in Cytoscape [63] for network analysis using the WikiPathways app [64].

Furthermore, the pathway module of ArrayAnalysis.org illustrates how the data visualisation and over-representation analysis of PathVisio can be incorporated into an existing data analysis pipeline using PathVisioRPC. This online module can also be particularly useful for researchers who only need pathway analysis once for their current dataset, allowing them to use the core functionalities of PathVisio over the internet without having to download and install the software itself.

Reactome pathways can be used in analysis already as they have been converted to GPML. Similarly other pathway sets (e.g. KEGG and BioPAX) can be used in analysis when these are converted into GPML. The functionality can also be easily extended by registering functionalities of other PathVisio plugins like the GSEA plugin [65] in the PathVisioRPC interface. There are some ongoing initiatives to use PathVisioRPC in larger analysis approaches. We are aware of pathway visualisation modules planned for a project regarding gene-based clinical assays to guide therapy in leukaemia patients and one for the Open PHACTS [66] project that will use the PathVisioRPC interface as well. Finally, the ArrayAnalysis.org pipeline is part of the larger dbNP project [67] that would contain other data analysis pipelines that are meant to make use of the pathway module provided at ArrayAnalysis.org as well. PathVisioRPC thus offers versatile solutions to integrate pathway analysis and visualisation approaches in many workflows.

## Availability and requirements

- **Project name:** PathVisioRPC
- **Project home page:**http://projects.bigcat.unimaas.nl/pathvisiorpc
- **Operating system(s):** Platform-independent
- **Programming language:** Java
- **Other requirements:** None
- **License:** Apache 2 License
- **Any restrictions to use by non-academics:** None other than those defined by the license.

## Additional files

Additional file 1: Table S1. Java API for PathVisioRPC. The table lists and provides brief descriptions of all functions implemented in PathVisioRPC, which can be called from the client languages to execute tasks. (TXT 6 kb) Additional file 2:
**PathVisioRPC jar files.** This zip archive contains both the plugin and standalone versions of the PathVisioRPC program packaged as jar files. The plugin can be installed in the PathVisio program and the standalone jar can be run in the command line to launch the PathVisioRPC server. The jar files are being provided for archival purposes, for use please obtain a recent version of the software from the project websites. (ZIP 11950 kb) Additional file 3:
**Examples in Python.** This zip archive contains the data and python script for the three python examples. (ZIP 15714 kb) Additional file 4:
**R scripts for the case study**.This zip archive contains the R scripts used in the case study including the arrayQC workflow. (ZIP 46 kb) Additional file 5: Figure S1. Box plots showing array quality control and normalisation results. Box plots showing array quality control and normalization results using different normalization methods (BGcorrected, Loess, Loess + Scale, Loess + Quantile). (PDF 424 kb) Additional file 6: Table S2. Gene level statistics. The lists of differentially expressed genes for each of the nine comparisons were collated into a single file, which was used for further analysis. (TXT 722 kb) Additional file 7: Table S3. Table containing highly enriched GO terms for bone marrow cells, PBMCs, and splenocytes. (TXT 10 kb) Additional file 8: Figure S2. Oxidative stress pathway [52] for splenocytes showing the logFC and P-value for day 1, day 2 and day 5. (PDF 104 kb) Additional file 9: Figure S3. Oxidative stress pathway [52] for bone marrow cells showing the logFC and P-value for day 1, day 2, and day 5. (PDF 105 kb) Additional file 10:
**R packages.** This zip archive contains both the RPathVisio package and the BridgeDbR package. The RPathVisio and BridgeDbR packages can be used in R. The jar files are being provided for archival purposes, for use please obtain a recent version of the software from the project websites. (ZIP 14914 kb)

## Abbreviations

KEGG: Kyoto encyclopaedia of genes and genomes
GPML: Graphical pathway mark-up language
GUI: Graphical user interface
RPC: Remote procedure call
XMLRPC: Extensible mark-up language remote procedure call
HTTP: Hypertext transfer protocol
PHP: Hypertext pre-processor
API: Application programming interface
PBMC: Peripheral blood monocytes
NCBI: National Centre for Bioinformatics
GEO: Gene expression omnibus
PNG: Portable network graphics
HTML: Hypertext mark-up language
DNA: Deoxy ribonucleic acid
GO: Gene ontology
