# Supplementary material for: Automatically visualise and analyse data on pathways using PathVisioRPC from any programming environment
Source: BMC Bioinformatics. 2015 Aug 23;16(1):267. doi: 10.1186/s12859-015-0708-8 (PMC4546821; doi:10.1186/s12859-015-0708-8)
Supplement: Additional file 3: — Examples in Python. This zip archive contains the data and python script for the three python examples. (ZIP 15714 kb) [file 12859_2015_708_MOESM3_ESM.zip › Python_Examples/result_Example_1/geneList1/backpage/L_11298.html]

 

# geneproduct annotation

  

| Name: Aanat| Identifier: 11298| Database: Entrez Gene| Synonyms: AA-NAT | | | --- | --- | | | | --- | --- | --- | --- | | | | --- | --- | --- | --- | --- | --- | | |
| --- | --- | --- | --- | --- | --- | --- | --- |

# Expression data

**Gene id on mapp: 11298**

| Sample name 11298| SystemCode L| LogFC 0.0| Pvalue 0.198048015| Type trans-PPS2 | | | --- | --- | | | | --- | --- | --- | --- | | | | --- | --- | --- | --- | --- | --- | | | | --- | --- | --- | --- | --- | --- | --- | --- | | |
| --- | --- | --- | --- | --- | --- | --- | --- | --- | --- |

  
  

---

  
  

# Cross references

  

|
|  |
| **UniGene** |
| Mm.42233 |
|
| **Agilent** |
| A\_51\_P295511 |
| A\_52\_P497424 |
| A\_55\_P2158384 |
|
| **Ensembl** |
| ENSMUSG00000020804 |
|
| **Illumina** |
| ILMN\_2671661 |
|
| **Entrez Gene** |
| 11298 |
|
| **MGI** |
| MGI:1328365 |
|
| **RefSeq** |
| NM\_009591 |
| NP\_033721 |
|
| **Uniprot/TrEMBL** |
| F2Z3V1 |
| O88816 |
| Q14A64 |
| Q9QUP1 |
|
| **GeneOntology** |
| GO:0004059 |
| GO:0004060 |
| GO:0005737 |
| GO:0006474 |
| GO:0007623 |
| GO:0009416 |
| GO:0010043 |
| GO:0014070 |
| GO:0030187 |
| GO:0032868 |
| GO:0034097 |
| GO:0034695 |
| GO:0046688 |
| GO:0048471 |
| GO:0051412 |
| GO:0051591 |
| GO:0051592 |
| GO:0071320 |
| GO:0071889 |
|
| **UCSC Genome Browser** |
| uc007mlo.2 |
| uc007mlq.1 |
|
| **WikiGenes** |
| 11298 |
|
| **Affy** |
| 10382816 |
| 1421666\_a\_at |
| 92511\_at |
| 92512\_g\_at |
