# Supplementary material for: Automatically visualise and analyse data on pathways using PathVisioRPC from any programming environment
Source: BMC Bioinformatics. 2015 Aug 23;16(1):267. doi: 10.1186/s12859-015-0708-8 (PMC4546821; doi:10.1186/s12859-015-0708-8)
Supplement: Additional file 3: — Examples in Python. This zip archive contains the data and python script for the three python examples. (ZIP 15714 kb) [file 12859_2015_708_MOESM3_ESM.zip › Python_Examples/result_Example_1/geneList1/backpage/L_11302.html]

 

# geneproduct annotation

  

| Name: Aatk| Identifier: 11302| Database: Entrez Gene| Synonyms: aatyk1 | | | --- | --- | | | | --- | --- | --- | --- | | | | --- | --- | --- | --- | --- | --- | | |
| --- | --- | --- | --- | --- | --- | --- | --- |

# Expression data

**Gene id on mapp: 11302**

| Sample name 11302| SystemCode L| LogFC 1.36686401| Pvalue 1.9763E-4| Type trans-PPS2 | | | --- | --- | | | | --- | --- | --- | --- | | | | --- | --- | --- | --- | --- | --- | | | | --- | --- | --- | --- | --- | --- | --- | --- | | |
| --- | --- | --- | --- | --- | --- | --- | --- | --- | --- |

  
  

---

  
  

# Cross references

  

|
|  |
| **UniGene** |
| Mm.488770 |
| Mm.6826 |
|
| **Agilent** |
| A\_51\_P381683 |
| A\_55\_P2042156 |
|
| **Ensembl** |
| ENSMUSG00000025375 |
|
| **Illumina** |
| ILMN\_1231439 |
|
| **Entrez Gene** |
| 11302 |
|
| **MGI** |
| MGI:1197518 |
|
| **RefSeq** |
| NM\_001198785 |
| NM\_001198787 |
| NM\_007377 |
| NP\_001185714 |
| NP\_001185716 |
| NP\_031403 |
|
| **Uniprot/TrEMBL** |
| B1AZF3 |
| B1AZF9 |
|
| **GeneOntology** |
| GO:0004713 |
| GO:0005515 |
| GO:0005524 |
| GO:0005739 |
| GO:0005783 |
| GO:0006915 |
| GO:0007420 |
| GO:0030517 |
| GO:0032482 |
| GO:0038083 |
| GO:0044295 |
| GO:0046777 |
| GO:0048471 |
| GO:0055037 |
|
| **UCSC Genome Browser** |
| uc007mrl.3 |
| uc007mrm.3 |
| uc007mrn.3 |
| uc007mro.3 |
|
| **WikiGenes** |
| 11302 |
|
| **Affy** |
| 100994\_at |
| 10393668 |
| 10393685 |
| 1416936\_at |
| AF011908\_at |
| AF011908\_g\_at |
