# Supplementary material for: Automatically visualise and analyse data on pathways using PathVisioRPC from any programming environment
Source: BMC Bioinformatics. 2015 Aug 23;16(1):267. doi: 10.1186/s12859-015-0708-8 (PMC4546821; doi:10.1186/s12859-015-0708-8)
Supplement: Additional file 3: — Examples in Python. This zip archive contains the data and python script for the three python examples. (ZIP 15714 kb) [file 12859_2015_708_MOESM3_ESM.zip › Python_Examples/result_Example_1/geneList1/backpage/L_11350.html]

 

# geneproduct annotation

  

| Name: Abl1| Identifier: 11350| Database: Entrez Gene| Synonyms: c-Abl | | | --- | --- | | | | --- | --- | --- | --- | | | | --- | --- | --- | --- | --- | --- | | |
| --- | --- | --- | --- | --- | --- | --- | --- |

# Expression data

**Gene id on mapp: 11350**

| Sample name 11350| SystemCode L| LogFC 1.185500967| Pvalue 0.036489085| Type trans-PPS2 | | | --- | --- | | | | --- | --- | --- | --- | | | | --- | --- | --- | --- | --- | --- | | | | --- | --- | --- | --- | --- | --- | --- | --- | | |
| --- | --- | --- | --- | --- | --- | --- | --- | --- | --- |

  
  

---

  
  

# Cross references

  

|
|  |
| **UniGene** |
| Mm.1318 |
|
| **Agilent** |
| A\_52\_P209101 |
| A\_52\_P333953 |
| A\_52\_P462217 |
| A\_55\_P2024808 |
| A\_55\_P2165239 |
|
| **Ensembl** |
| ENSMUSG00000026842 |
|
| **Illumina** |
| ILMN\_1233059 |
| ILMN\_1234313 |
| ILMN\_1243738 |
| ILMN\_1245370 |
| ILMN\_2675551 |
| ILMN\_2742730 |
| ILMN\_2796842 |
|
| **Entrez Gene** |
| 11350 |
|
| **MGI** |
| MGI:87859 |
|
| **PDB** |
| 1ABO |
| 1ABQ |
| 1FPU |
| 1IEP |
| 1M52 |
| 1OPJ |
| 1OPK |
| 2HZN |
| 2QOH |
| 2Z60 |
| 3DK3 |
| 3DK6 |
| 3DK7 |
| 3IK3 |
| 3K5V |
| 3KF4 |
| 3KFA |
| 3MS9 |
| 3MSS |
| 3OXZ |
| 3OY3 |
|
| **RefSeq** |
| NM\_001112703 |
| NM\_009594 |
| NP\_001106174 |
| NP\_033724 |
|
| **Uniprot/TrEMBL** |
| A2AV23 |
| P00520 |
| Q3SYK5 |
| Q3V2W1 |
|
| **GeneOntology** |
| GO:0000287 |
| GO:0003677 |
| GO:0004672 |
| GO:0004713 |
| GO:0004715 |
| GO:0005515 |
| GO:0005524 |
| GO:0005634 |
| GO:0005730 |
| GO:0005737 |
| GO:0005739 |
| GO:0005829 |
| GO:0005856 |
| GO:0006281 |
| GO:0006897 |
| GO:0006914 |
| GO:0006915 |
| GO:0006974 |
| GO:0006975 |
| GO:0007155 |
| GO:0008022 |
| GO:0016301 |
| GO:0017124 |
| GO:0018108 |
| GO:0019904 |
| GO:0019905 |
| GO:0030036 |
| GO:0030145 |
| GO:0031252 |
| GO:0042770 |
| GO:0043065 |
| GO:0048008 |
| GO:0048471 |
| GO:0050731 |
| GO:0051019 |
| GO:0051353 |
| GO:0051726 |
| GO:0070064 |
| GO:0071901 |
| GO:2001020 |
|
| **UCSC Genome Browser** |
| uc008jeb.2 |
| uc008jec.2 |
|
| **WikiGenes** |
| 11350 |
|
| **Affy** |
| 102772\_at |
| 10471201 |
| 1423999\_at |
| 1441291\_at |
| 1444134\_at |
| Msa.2144.0\_at |
| j02995-2\_s\_at |
