# Supplementary material for: Automatically visualise and analyse data on pathways using PathVisioRPC from any programming environment
Source: BMC Bioinformatics. 2015 Aug 23;16(1):267. doi: 10.1186/s12859-015-0708-8 (PMC4546821; doi:10.1186/s12859-015-0708-8)
Supplement: Additional file 3: — Examples in Python. This zip archive contains the data and python script for the three python examples. (ZIP 15714 kb) [file 12859_2015_708_MOESM3_ESM.zip › Python_Examples/result_Example_1/geneList1/backpage/L_11352.html]

 

# geneproduct annotation

  

| Name: Abl2| Identifier: 11352| Database: Entrez Gene| Synonyms: Arg | | | --- | --- | | | | --- | --- | --- | --- | | | | --- | --- | --- | --- | --- | --- | | |
| --- | --- | --- | --- | --- | --- | --- | --- |

# Expression data

**Gene id on mapp: 11352**

| Sample name 11352| SystemCode L| LogFC 0.0| Pvalue 0.777237087| Type trans-PPS2 | | | --- | --- | | | | --- | --- | --- | --- | | | | --- | --- | --- | --- | --- | --- | | | | --- | --- | --- | --- | --- | --- | --- | --- | | |
| --- | --- | --- | --- | --- | --- | --- | --- | --- | --- |

  
  

---

  
  

# Cross references

  

|
|  |
| **UniGene** |
| Mm.408854 |
| Mm.470439 |
|
| **Agilent** |
| A\_51\_P461884 |
| A\_52\_P176960 |
| A\_52\_P610121 |
|
| **Ensembl** |
| ENSMUSG00000026596 |
|
| **Illumina** |
| ILMN\_2698137 |
|
| **Entrez Gene** |
| 11352 |
|
| **MGI** |
| MGI:87860 |
|
| **RefSeq** |
| NM\_001136104 |
| NM\_009595 |
| NP\_001129576 |
| NP\_033725 |
|
| **Uniprot/TrEMBL** |
| B2RQ57 |
| F8VQH0 |
| G3UWQ2 |
| Q61055 |
| Q99P28 |
|
| **GeneOntology** |
| GO:0000287 |
| GO:0004713 |
| GO:0004715 |
| GO:0005515 |
| GO:0005524 |
| GO:0007015 |
| GO:0010976 |
| GO:0015629 |
| GO:0016023 |
| GO:0018108 |
| GO:0030145 |
| GO:0051017 |
| GO:0051353 |
| GO:0071300 |
|
| **UCSC Genome Browser** |
| uc007dcl.2 |
| uc011wuf.1 |
|
| **WikiGenes** |
| 11352 |
|
| **Affy** |
| 10350823 |
| 110513\_at |
| 115788\_at |
| 1445483\_at |
| 1455495\_at |
| 1455682\_at |
