# Supplementary material for: Automatically visualise and analyse data on pathways using PathVisioRPC from any programming environment
Source: BMC Bioinformatics. 2015 Aug 23;16(1):267. doi: 10.1186/s12859-015-0708-8 (PMC4546821; doi:10.1186/s12859-015-0708-8)
Supplement: Additional file 3: — Examples in Python. This zip archive contains the data and python script for the three python examples. (ZIP 15714 kb) [file 12859_2015_708_MOESM3_ESM.zip › Python_Examples/result_Example_1/geneList1/backpage/L_11370.html]

 

# geneproduct annotation

  

| Name: Acadvl| Identifier: 11370| Database: Entrez Gene| Synonyms: vlcad | | | --- | --- | | | | --- | --- | --- | --- | | | | --- | --- | --- | --- | --- | --- | | |
| --- | --- | --- | --- | --- | --- | --- | --- |

# Expression data

**Gene id on mapp: 11370**

| Sample name 11370| SystemCode L| LogFC 0.0| Pvalue 0.411247584| Type trans-PPS2 | | | --- | --- | | | | --- | --- | --- | --- | | | | --- | --- | --- | --- | --- | --- | | | | --- | --- | --- | --- | --- | --- | --- | --- | | |
| --- | --- | --- | --- | --- | --- | --- | --- | --- | --- |

  
  

---

  
  

# Cross references

  

|
|  |
| **UniGene** |
| Mm.18630 |
|
| **Agilent** |
| A\_51\_P518340 |
| A\_55\_P1991906 |
|
| **Ensembl** |
| ENSMUSG00000018574 |
|
| **Illumina** |
| ILMN\_2775586 |
| ILMN\_2956932 |
|
| **Entrez Gene** |
| 11370 |
|
| **MGI** |
| MGI:895149 |
|
| **RefSeq** |
| NM\_017366 |
| NP\_059062 |
|
| **Uniprot/TrEMBL** |
| B1AR28 |
| P50544 |
| Q3UJR6 |
|
| **GeneOntology** |
| GO:0001659 |
| GO:0003995 |
| GO:0005739 |
| GO:0005743 |
| GO:0033539 |
| GO:0042645 |
| GO:0045717 |
| GO:0046322 |
| GO:0050660 |
| GO:0090181 |
|
| **UCSC Genome Browser** |
| uc007jto.1 |
|
| **WikiGenes** |
| 11370 |
|
| **Affy** |
| 10387768 |
| 1424184\_at |
| af017176\_s\_at |
