# Supplementary material for: Automatically visualise and analyse data on pathways using PathVisioRPC from any programming environment
Source: BMC Bioinformatics. 2015 Aug 23;16(1):267. doi: 10.1186/s12859-015-0708-8 (PMC4546821; doi:10.1186/s12859-015-0708-8)
Supplement: Additional file 3: — Examples in Python. This zip archive contains the data and python script for the three python examples. (ZIP 15714 kb) [file 12859_2015_708_MOESM3_ESM.zip › Python_Examples/result_Example_1/geneList1/backpage/L_11416.html]

 

# geneproduct annotation

  

| Name: Slc33a1| Identifier: 11416| Database: Entrez Gene| Synonyms: AI315656 | | | --- | --- | | | | --- | --- | --- | --- | | | | --- | --- | --- | --- | --- | --- | | |
| --- | --- | --- | --- | --- | --- | --- | --- |

# Expression data

**Gene id on mapp: 11416**

| Sample name 11416| SystemCode L| LogFC 0.0| Pvalue 0.29497702| Type trans-PPS2 | | | --- | --- | | | | --- | --- | --- | --- | | | | --- | --- | --- | --- | --- | --- | | | | --- | --- | --- | --- | --- | --- | --- | --- | | |
| --- | --- | --- | --- | --- | --- | --- | --- | --- | --- |

  
  

---

  
  

# Cross references

  

|
|  |
| **UniGene** |
| Mm.135619 |
| Mm.470325 |
|
| **Agilent** |
| A\_52\_P551903 |
| A\_55\_P2176300 |
|
| **Ensembl** |
| ENSMUSG00000027822 |
|
| **Illumina** |
| ILMN\_1222499 |
|
| **Entrez Gene** |
| 11416 |
|
| **MGI** |
| MGI:1332247 |
|
| **RefSeq** |
| NM\_001272035 |
| NM\_015728 |
| NP\_001258964 |
| NP\_056543 |
|
| **Uniprot/TrEMBL** |
| Q3UXZ5 |
| Q99J27 |
|
| **GeneOntology** |
| GO:0005789 |
| GO:0008521 |
| GO:0016021 |
|
| **UCSC Genome Browser** |
| uc008pkc.1 |
| uc008pkd.1 |
|
| **WikiGenes** |
| 11416 |
|
| **Affy** |
| 10498485 |
| 1423621\_a\_at |
| 160201\_r\_at |
| AA636548\_at |
