# Supplementary material for: Automatically visualise and analyse data on pathways using PathVisioRPC from any programming environment
Source: BMC Bioinformatics. 2015 Aug 23;16(1):267. doi: 10.1186/s12859-015-0708-8 (PMC4546821; doi:10.1186/s12859-015-0708-8)
Supplement: Additional file 3: — Examples in Python. This zip archive contains the data and python script for the three python examples. (ZIP 15714 kb) [file 12859_2015_708_MOESM3_ESM.zip › Python_Examples/result_Example_1/geneList1/backpage/L_11419.html]

 

# geneproduct annotation

  

| Name: Asic1| Identifier: 11419| Database: Entrez Gene| Synonyms: AI843610 | | | --- | --- | | | | --- | --- | --- | --- | | | | --- | --- | --- | --- | --- | --- | | |
| --- | --- | --- | --- | --- | --- | --- | --- |

# Expression data

**Gene id on mapp: 11419**

| Sample name 11419| SystemCode L| LogFC 0.0| Pvalue 0.148605161| Type trans-PPS2 | | | --- | --- | | | | --- | --- | --- | --- | | | | --- | --- | --- | --- | --- | --- | | | | --- | --- | --- | --- | --- | --- | --- | --- | | |
| --- | --- | --- | --- | --- | --- | --- | --- | --- | --- |

  
  

---

  
  

# Cross references

  

|
|  |
| **UniGene** |
| Mm.440107 |
|
| **Agilent** |
| A\_51\_P140118 |
| A\_52\_P209832 |
| A\_55\_P2104988 |
|
| **Ensembl** |
| ENSMUSG00000023017 |
|
| **Illumina** |
| ILMN\_2643241 |
|
| **Entrez Gene** |
| 11419 |
|
| **MGI** |
| MGI:1194915 |
|
| **RefSeq** |
| NM\_009597 |
| NP\_033727 |
|
| **Uniprot/TrEMBL** |
| I6L9A4 |
| Q6NXK8 |
|
| **GeneOntology** |
| GO:0001101 |
| GO:0005261 |
| GO:0005515 |
| GO:0005887 |
| GO:0006812 |
| GO:0007613 |
| GO:0008306 |
| GO:0015280 |
| GO:0022839 |
| GO:0034220 |
| GO:0042391 |
| GO:0045202 |
| GO:0046929 |
| GO:0070588 |
|
| **UCSC Genome Browser** |
| uc007xqa.1 |
|
| **WikiGenes** |
| 11419 |
|
| **Affy** |
| 10426782 |
| 1455328\_at |
