# Supplementary material for: Automatically visualise and analyse data on pathways using PathVisioRPC from any programming environment
Source: BMC Bioinformatics. 2015 Aug 23;16(1):267. doi: 10.1186/s12859-015-0708-8 (PMC4546821; doi:10.1186/s12859-015-0708-8)
Supplement: Additional file 3: — Examples in Python. This zip archive contains the data and python script for the three python examples. (ZIP 15714 kb) [file 12859_2015_708_MOESM3_ESM.zip › Python_Examples/result_Example_1/geneList2/backpage/L_11432.html]

 

# geneproduct annotation

  

| Name: Acp2| Identifier: 11432| Database: Entrez Gene| Synonyms: Acp-2 | | | --- | --- | | | | --- | --- | --- | --- | | | | --- | --- | --- | --- | --- | --- | | |
| --- | --- | --- | --- | --- | --- | --- | --- |

# Expression data

**Gene id on mapp: 11432**

| Sample name 11432| SystemCode L| LogFC 0.0| Pvalue 0.953588299| Type trans-PPS2 | | | --- | --- | | | | --- | --- | --- | --- | | | | --- | --- | --- | --- | --- | --- | | | | --- | --- | --- | --- | --- | --- | --- | --- | | |
| --- | --- | --- | --- | --- | --- | --- | --- | --- | --- |

  
  

---

  
  

# Cross references

  

|
|  |
| **UniGene** |
| Mm.45570 |
|
| **Agilent** |
| A\_51\_P516456 |
| A\_52\_P215701 |
| A\_52\_P30550 |
| A\_55\_P1985385 |
|
| **Ensembl** |
| ENSMUSG00000002103 |
|
| **Illumina** |
| ILMN\_1234364 |
| ILMN\_2513205 |
| ILMN\_2513207 |
| ILMN\_2887075 |
|
| **Entrez Gene** |
| 11432 |
|
| **MGI** |
| MGI:87882 |
|
| **RefSeq** |
| NM\_007387 |
| NP\_031413 |
|
| **Uniprot/TrEMBL** |
| B7ZCF4 |
| B7ZCF5 |
| P24638 |
|
| **GeneOntology** |
| GO:0001501 |
| GO:0003993 |
| GO:0005764 |
| GO:0005765 |
| GO:0007040 |
| GO:0016021 |
| GO:0043202 |
|
| **UCSC Genome Browser** |
| uc008kvf.2 |
|
| **WikiGenes** |
| 11432 |
|
| **Affy** |
| 10473847 |
| 109116\_s\_at |
| 109117\_at |
| 109528\_at |
| 116135\_at |
| 1424654\_at |
| 1424655\_at |
| 1436788\_at |
| 92688\_at |
| Msa.2416.0\_at |
