# Supplementary material for: Automatically visualise and analyse data on pathways using PathVisioRPC from any programming environment
Source: BMC Bioinformatics. 2015 Aug 23;16(1):267. doi: 10.1186/s12859-015-0708-8 (PMC4546821; doi:10.1186/s12859-015-0708-8)
Supplement: Additional file 3: — Examples in Python. This zip archive contains the data and python script for the three python examples. (ZIP 15714 kb) [file 12859_2015_708_MOESM3_ESM.zip › Python_Examples/result_Example_1/geneList2/backpage/L_11433.html]

 

# geneproduct annotation

  

| Name: Acp5| Identifier: 11433| Database: Entrez Gene| Synonyms: TRACP | | | --- | --- | | | | --- | --- | --- | --- | | | | --- | --- | --- | --- | --- | --- | | |
| --- | --- | --- | --- | --- | --- | --- | --- |

# Expression data

**Gene id on mapp: 11433**

| Sample name 11433| SystemCode L| LogFC 0.0| Pvalue 0.108241682| Type trans-PPS2 | | | --- | --- | | | | --- | --- | --- | --- | | | | --- | --- | --- | --- | --- | --- | | | | --- | --- | --- | --- | --- | --- | --- | --- | | |
| --- | --- | --- | --- | --- | --- | --- | --- | --- | --- |

  
  

---

  
  

# Cross references

  

|
|  |
| **UniGene** |
| Mm.399836 |
| Mm.46354 |
|
| **Agilent** |
| A\_52\_P675157 |
| A\_55\_P1997766 |
| A\_55\_P2037608 |
|
| **Ensembl** |
| ENSMUSG00000001348 |
|
| **Illumina** |
| ILMN\_2735660 |
| ILMN\_2735661 |
|
| **Entrez Gene** |
| 11433 |
|
| **MGI** |
| MGI:87883 |
|
| **RefSeq** |
| NM\_001102404 |
| NM\_001102405 |
| NM\_007388 |
| NP\_001095874 |
| NP\_001095875 |
| NP\_031414 |
|
| **Uniprot/TrEMBL** |
| Q05117 |
| Q38RM9 |
|
| **GeneOntology** |
| GO:0003993 |
| GO:0005764 |
| GO:0008198 |
| GO:0008199 |
| GO:0016311 |
| GO:0032496 |
| GO:0032691 |
| GO:0032695 |
| GO:0032720 |
| GO:0032929 |
| GO:0034097 |
| GO:0045019 |
| GO:0045453 |
| GO:0050728 |
| GO:0050830 |
| GO:0060349 |
|
| **UCSC Genome Browser** |
| uc009onz.1 |
| uc009ooa.1 |
| uc009oob.1 |
|
| **WikiGenes** |
| 11433 |
|
| **Affy** |
| 10591739 |
| 1431609\_a\_at |
| 162543\_r\_at |
| 98859\_at |
| M99054\_s\_at |
