# Supplementary material for: Automatically visualise and analyse data on pathways using PathVisioRPC from any programming environment
Source: BMC Bioinformatics. 2015 Aug 23;16(1):267. doi: 10.1186/s12859-015-0708-8 (PMC4546821; doi:10.1186/s12859-015-0708-8)
Supplement: Additional file 3: — Examples in Python. This zip archive contains the data and python script for the three python examples. (ZIP 15714 kb) [file 12859_2015_708_MOESM3_ESM.zip › Python_Examples/result_Example_1/geneList2/backpage/L_11438.html]

 

# geneproduct annotation

  

| Name: Chrna4| Identifier: 11438| Database: Entrez Gene| Synonyms: ENFL1 | | | --- | --- | | | | --- | --- | --- | --- | | | | --- | --- | --- | --- | --- | --- | | |
| --- | --- | --- | --- | --- | --- | --- | --- |

# Expression data

**Gene id on mapp: 11438**

| Sample name 11438| SystemCode L| LogFC 0.0| Pvalue 0.199819661| Type trans-PPS2 | | | --- | --- | | | | --- | --- | --- | --- | | | | --- | --- | --- | --- | --- | --- | | | | --- | --- | --- | --- | --- | --- | --- | --- | | |
| --- | --- | --- | --- | --- | --- | --- | --- | --- | --- |

  
  

---

  
  

# Cross references

  

|
|  |
| **UniGene** |
| Mm.252369 |
|
| **Agilent** |
| A\_51\_P104487 |
| A\_52\_P350750 |
| A\_66\_P101862 |
|
| **Ensembl** |
| ENSMUSG00000027577 |
|
| **Illumina** |
| ILMN\_1245582 |
| ILMN\_2742576 |
| ILMN\_2832675 |
|
| **Entrez Gene** |
| 11438 |
|
| **MGI** |
| MGI:87888 |
|
| **RefSeq** |
| NM\_015730 |
| NP\_056545 |
|
| **Uniprot/TrEMBL** |
| B7ZBU7 |
| B7ZBV1 |
| E0CZ39 |
| O70174 |
| Q53YK0 |
|
| **GeneOntology** |
| GO:0001508 |
| GO:0001666 |
| GO:0004889 |
| GO:0005216 |
| GO:0005515 |
| GO:0005886 |
| GO:0005892 |
| GO:0006281 |
| GO:0006816 |
| GO:0006979 |
| GO:0007165 |
| GO:0007271 |
| GO:0007585 |
| GO:0007626 |
| GO:0009897 |
| GO:0014059 |
| GO:0015464 |
| GO:0016020 |
| GO:0019233 |
| GO:0030054 |
| GO:0035094 |
| GO:0035095 |
| GO:0035640 |
| GO:0042113 |
| GO:0042391 |
| GO:0045211 |
| GO:0050877 |
| GO:0050890 |
| GO:0051899 |
| GO:0060080 |
|
| **UCSC Genome Browser** |
| uc008okq.1 |
|
| **WikiGenes** |
| 11438 |
|
| **Affy** |
| 10490559 |
| 110658\_at |
| 1421202\_at |
| 1421203\_at |
