# Supplementary material for: Automatically visualise and analyse data on pathways using PathVisioRPC from any programming environment
Source: BMC Bioinformatics. 2015 Aug 23;16(1):267. doi: 10.1186/s12859-015-0708-8 (PMC4546821; doi:10.1186/s12859-015-0708-8)
Supplement: Additional file 3: — Examples in Python. This zip archive contains the data and python script for the three python examples. (ZIP 15714 kb) [file 12859_2015_708_MOESM3_ESM.zip › Python_Examples/result_Example_1/geneList2/backpage/L_11440.html]

 

# geneproduct annotation

  

| Name: Chrna6| Identifier: 11440| Database: Entrez Gene| Synonyms: Nica6 | | | --- | --- | | | | --- | --- | --- | --- | | | | --- | --- | --- | --- | --- | --- | | |
| --- | --- | --- | --- | --- | --- | --- | --- |

# Expression data

**Gene id on mapp: 11440**

| Sample name 11440| SystemCode L| LogFC 0.0| Pvalue 0.094558688| Type trans-PPS2 | | | --- | --- | | | | --- | --- | --- | --- | | | | --- | --- | --- | --- | --- | --- | | | | --- | --- | --- | --- | --- | --- | --- | --- | | |
| --- | --- | --- | --- | --- | --- | --- | --- | --- | --- |

  
  

---

  
  

# Cross references

  

|
|  |
| **UniGene** |
| Mm.283137 |
|
| **Agilent** |
| A\_51\_P499653 |
| A\_55\_P2041065 |
|
| **Ensembl** |
| ENSMUSG00000031491 |
|
| **Illumina** |
| ILMN\_2732437 |
| ILMN\_2732438 |
|
| **Entrez Gene** |
| 11440 |
|
| **MGI** |
| MGI:106213 |
|
| **RefSeq** |
| NM\_021369 |
| NP\_067344 |
|
| **Uniprot/TrEMBL** |
| Q9R0W9 |
|
| **GeneOntology** |
| GO:0004889 |
| GO:0005515 |
| GO:0005892 |
| GO:0014059 |
| GO:0030054 |
| GO:0045211 |
| GO:0051899 |
|
| **UCSC Genome Browser** |
| uc009liv.1 |
|
| **WikiGenes** |
| 11440 |
|
| **Affy** |
| 10577980 |
| 138177\_at |
| 1450426\_at |
| 1450427\_at |
| 96996\_at |
