# Supplementary material for: Automatically visualise and analyse data on pathways using PathVisioRPC from any programming environment
Source: BMC Bioinformatics. 2015 Aug 23;16(1):267. doi: 10.1186/s12859-015-0708-8 (PMC4546821; doi:10.1186/s12859-015-0708-8)
Supplement: Additional file 3: — Examples in Python. This zip archive contains the data and python script for the three python examples. (ZIP 15714 kb) [file 12859_2015_708_MOESM3_ESM.zip › Python_Examples/result_Example_1/geneList2/backpage/L_11441.html]

 

# geneproduct annotation

  

| Name: Chrna7| Identifier: 11441| Database: Entrez Gene| Synonyms: Acra7 | | | --- | --- | | | | --- | --- | --- | --- | | | | --- | --- | --- | --- | --- | --- | | |
| --- | --- | --- | --- | --- | --- | --- | --- |

# Expression data

**Gene id on mapp: 11441**

| Sample name 11441| SystemCode L| LogFC 0.0| Pvalue 0.872348609| Type trans-PPS2 | | | --- | --- | | | | --- | --- | --- | --- | | | | --- | --- | --- | --- | --- | --- | | | | --- | --- | --- | --- | --- | --- | --- | --- | | |
| --- | --- | --- | --- | --- | --- | --- | --- | --- | --- |

  
  

---

  
  

# Cross references

  

|
|  |
| **Agilent** |
| A\_51\_P108228 |
| A\_52\_P648773 |
|
| **Ensembl** |
| ENSMUSG00000030525 |
|
| **Illumina** |
| ILMN\_1246234 |
|
| **Entrez Gene** |
| 11441 |
|
| **MGI** |
| MGI:99779 |
|
| **RefSeq** |
| NM\_007390 |
| NP\_031416 |
|
| **Uniprot/TrEMBL** |
| P49582 |
| Q53YJ9 |
|
| **GeneOntology** |
| GO:0001988 |
| GO:0004889 |
| GO:0005515 |
| GO:0005737 |
| GO:0005886 |
| GO:0006811 |
| GO:0006816 |
| GO:0006897 |
| GO:0007271 |
| GO:0007613 |
| GO:0008144 |
| GO:0008179 |
| GO:0008306 |
| GO:0009897 |
| GO:0014061 |
| GO:0014069 |
| GO:0016020 |
| GO:0016021 |
| GO:0016324 |
| GO:0019228 |
| GO:0019901 |
| GO:0030054 |
| GO:0030317 |
| GO:0030424 |
| GO:0030425 |
| GO:0030426 |
| GO:0032094 |
| GO:0032225 |
| GO:0032279 |
| GO:0032691 |
| GO:0032715 |
| GO:0032720 |
| GO:0035094 |
| GO:0035095 |
| GO:0042110 |
| GO:0042113 |
| GO:0042166 |
| GO:0042221 |
| GO:0042391 |
| GO:0042416 |
| GO:0042698 |
| GO:0042734 |
| GO:0043025 |
| GO:0043197 |
| GO:0043198 |
| GO:0045121 |
| GO:0045211 |
| GO:0045471 |
| GO:0048149 |
| GO:0050727 |
| GO:0050728 |
| GO:0060112 |
|
| **UCSC Genome Browser** |
| uc009vel.1 |
|
| **WikiGenes** |
| 11441 |
|
| **Affy** |
| 101131\_at |
| 10564272 |
| 1450299\_at |
| L37663\_s\_at |
