# Supplementary material for: Automatically visualise and analyse data on pathways using PathVisioRPC from any programming environment
Source: BMC Bioinformatics. 2015 Aug 23;16(1):267. doi: 10.1186/s12859-015-0708-8 (PMC4546821; doi:10.1186/s12859-015-0708-8)
Supplement: Additional file 3: — Examples in Python. This zip archive contains the data and python script for the three python examples. (ZIP 15714 kb) [file 12859_2015_708_MOESM3_ESM.zip › Python_Examples/result_Example_1/geneList2/backpage/L_11444.html]

 

# geneproduct annotation

  

| Name: Chrnb2| Identifier: 11444| Database: Entrez Gene| Synonyms: [b]2-nAchR | | | --- | --- | | | | --- | --- | --- | --- | | | | --- | --- | --- | --- | --- | --- | | |
| --- | --- | --- | --- | --- | --- | --- | --- |

# Expression data

**Gene id on mapp: 11444**

| Sample name 11444| SystemCode L| LogFC 0.0| Pvalue 0.053186564| Type trans-PPS2 | | | --- | --- | | | | --- | --- | --- | --- | | | | --- | --- | --- | --- | --- | --- | | | | --- | --- | --- | --- | --- | --- | --- | --- | | |
| --- | --- | --- | --- | --- | --- | --- | --- | --- | --- |

  
  

---

  
  

# Cross references

  

|
|  |
| **UniGene** |
| Mm.35088 |
|
| **Agilent** |
| A\_52\_P222026 |
|
| **Ensembl** |
| ENSMUSG00000027950 |
|
| **Illumina** |
| ILMN\_2612392 |
| ILMN\_2759307 |
| ILMN\_2865297 |
|
| **Entrez Gene** |
| 11444 |
|
| **MGI** |
| MGI:87891 |
|
| **RefSeq** |
| NM\_009602 |
| NP\_033732 |
|
| **Uniprot/TrEMBL** |
| Q61943 |
| Q8BGP7 |
| Q8VI08 |
|
| **GeneOntology** |
| GO:0001508 |
| GO:0001661 |
| GO:0001666 |
| GO:0004889 |
| GO:0005515 |
| GO:0005886 |
| GO:0005892 |
| GO:0006816 |
| GO:0006939 |
| GO:0007165 |
| GO:0007271 |
| GO:0007601 |
| GO:0007605 |
| GO:0007612 |
| GO:0007613 |
| GO:0007626 |
| GO:0008306 |
| GO:0008542 |
| GO:0009897 |
| GO:0014059 |
| GO:0015464 |
| GO:0019233 |
| GO:0021562 |
| GO:0021631 |
| GO:0021771 |
| GO:0021952 |
| GO:0021955 |
| GO:0030054 |
| GO:0030890 |
| GO:0032225 |
| GO:0032226 |
| GO:0033603 |
| GO:0035094 |
| GO:0035095 |
| GO:0035176 |
| GO:0042053 |
| GO:0042113 |
| GO:0042166 |
| GO:0042220 |
| GO:0042320 |
| GO:0042391 |
| GO:0045188 |
| GO:0045211 |
| GO:0045471 |
| GO:0045759 |
| GO:0048814 |
| GO:0050877 |
| GO:0050890 |
| GO:0051899 |
| GO:0051963 |
| GO:0060084 |
|
| **UCSC Genome Browser** |
| uc008qaa.2 |
|
| **WikiGenes** |
| 11444 |
|
| **Affy** |
| 10499643 |
| 110137\_at |
| 115849\_at |
| 1420744\_at |
| 1436428\_at |
| 97010\_at |
