# Supplementary material for: Automatically visualise and analyse data on pathways using PathVisioRPC from any programming environment
Source: BMC Bioinformatics. 2015 Aug 23;16(1):267. doi: 10.1186/s12859-015-0708-8 (PMC4546821; doi:10.1186/s12859-015-0708-8)
Supplement: Additional file 3: — Examples in Python. This zip archive contains the data and python script for the three python examples. (ZIP 15714 kb) [file 12859_2015_708_MOESM3_ESM.zip › Python_Examples/result_Example_1/geneList2/backpage/L_11448.html]

 

# geneproduct annotation

  

| Name: Chrne| Identifier: 11448| Database: Entrez Gene| Synonyms: AChrepsilon | | | --- | --- | | | | --- | --- | --- | --- | | | | --- | --- | --- | --- | --- | --- | | |
| --- | --- | --- | --- | --- | --- | --- | --- |

# Expression data

**Gene id on mapp: 11448**

| Sample name 11448| SystemCode L| LogFC 0.0| Pvalue 0.560180124| Type trans-PPS2 | | | --- | --- | | | | --- | --- | --- | --- | | | | --- | --- | --- | --- | --- | --- | | | | --- | --- | --- | --- | --- | --- | --- | --- | | |
| --- | --- | --- | --- | --- | --- | --- | --- | --- | --- |

  
  

---

  
  

# Cross references

  

|
|  |
| **UniGene** |
| Mm.4980 |
|
| **Agilent** |
| A\_51\_P482820 |
| A\_52\_P522601 |
| A\_55\_P2180470 |
|
| **Ensembl** |
| ENSMUSG00000014609 |
|
| **Illumina** |
| ILMN\_2706323 |
| ILMN\_2706326 |
|
| **Entrez Gene** |
| 11448 |
|
| **MGI** |
| MGI:87894 |
|
| **RefSeq** |
| NM\_009603 |
| NP\_033733 |
|
| **Uniprot/TrEMBL** |
| P20782 |
| Q5SXG9 |
| Q8K1N0 |
|
| **GeneOntology** |
| GO:0004889 |
| GO:0005886 |
| GO:0016021 |
| GO:0030054 |
| GO:0042391 |
| GO:0045211 |
|
| **UCSC Genome Browser** |
| uc007jvo.1 |
|
| **WikiGenes** |
| 11448 |
|
| **Affy** |
| 10387909 |
| 1420560\_at |
| 92965\_at |
| 92966\_g\_at |
| 92967\_r\_at |
| Msa.26520.0\_s\_at |
| Msa.395.0\_s\_at |
| x55718\_at |
