# Supplementary material for: Automatically visualise and analyse data on pathways using PathVisioRPC from any programming environment
Source: BMC Bioinformatics. 2015 Aug 23;16(1):267. doi: 10.1186/s12859-015-0708-8 (PMC4546821; doi:10.1186/s12859-015-0708-8)
Supplement: Additional file 3: — Examples in Python. This zip archive contains the data and python script for the three python examples. (ZIP 15714 kb) [file 12859_2015_708_MOESM3_ESM.zip › Python_Examples/result_Example_1/geneList2/backpage/L_11451.html]

 

# geneproduct annotation

  

| Name: Acrv1| Identifier: 11451| Database: Entrez Gene| Synonyms: SP-10 | | | --- | --- | | | | --- | --- | --- | --- | | | | --- | --- | --- | --- | --- | --- | | |
| --- | --- | --- | --- | --- | --- | --- | --- |

# Expression data

**Gene id on mapp: 11451**

| Sample name 11451| SystemCode L| LogFC 0.0| Pvalue 0.747996065| Type trans-PPS2 | | | --- | --- | | | | --- | --- | --- | --- | | | | --- | --- | --- | --- | --- | --- | | | | --- | --- | --- | --- | --- | --- | --- | --- | | |
| --- | --- | --- | --- | --- | --- | --- | --- | --- | --- |

  
  

---

  
  

# Cross references

  

|
|  |
| **UniGene** |
| Mm.4776 |
|
| **Ensembl** |
| ENSMUSG00000032110 |
|
| **Illumina** |
| ILMN\_2677129 |
| ILMN\_2834622 |
|
| **Entrez Gene** |
| 11451 |
|
| **MGI** |
| MGI:104590 |
|
| **RefSeq** |
| NM\_007391 |
| NP\_031417 |
|
| **Uniprot/TrEMBL** |
| P50289 |
|
| **GeneOntology** |
| GO:0001669 |
|
| **UCSC Genome Browser** |
| uc009otu.1 |
|
| **WikiGenes** |
| 11451 |
|
| **Affy** |
| 10584254 |
| 1418878\_at |
| 92897\_at |
| U31992\_s\_at |
