# Supplementary material for: Automatically visualise and analyse data on pathways using PathVisioRPC from any programming environment
Source: BMC Bioinformatics. 2015 Aug 23;16(1):267. doi: 10.1186/s12859-015-0708-8 (PMC4546821; doi:10.1186/s12859-015-0708-8)
Supplement: Additional file 3: — Examples in Python. This zip archive contains the data and python script for the three python examples. (ZIP 15714 kb) [file 12859_2015_708_MOESM3_ESM.zip › Python_Examples/result_Example_1/geneList2/backpage/L_11461.html]

 

# geneproduct annotation

  

| Name: Actb| Identifier: 11461| Database: Entrez Gene| Synonyms: Actx | | | --- | --- | | | | --- | --- | --- | --- | | | | --- | --- | --- | --- | --- | --- | | |
| --- | --- | --- | --- | --- | --- | --- | --- |

# Expression data

**Gene id on mapp: 11461**

| Sample name 11461| SystemCode L| LogFC -1.25347113| Pvalue 0.012947146| Type trans-PPS2 | | | --- | --- | | | | --- | --- | --- | --- | | | | --- | --- | --- | --- | --- | --- | | | | --- | --- | --- | --- | --- | --- | --- | --- | | |
| --- | --- | --- | --- | --- | --- | --- | --- | --- | --- |

  
  

---

  
  

# Cross references

  

|
|  |
| **UniGene** |
| Mm.391967 |
| Mm.399519 |
| Mm.468932 |
|
| **Agilent** |
| A\_51\_P173760 |
| A\_55\_P2112737 |
|
| **Ensembl** |
| ENSMUSG00000029580 |
|
| **Illumina** |
| ILMN\_1377923 |
| ILMN\_2588055 |
| ILMN\_2617433 |
| ILMN\_2698799 |
| ILMN\_2743360 |
| ILMN\_2846865 |
|
| **Entrez Gene** |
| 11461 |
|
| **MGI** |
| MGI:87904 |
|
| **RefSeq** |
| NM\_007393 |
| NP\_031419 |
|
| **Uniprot/TrEMBL** |
| A1E281 |
| B2RRX1 |
| E9Q1F2 |
| E9Q2D1 |
| E9Q5F4 |
| E9Q606 |
| P60710 |
| Q6IWE2 |
| Q99NC5 |
|
| **GeneOntology** |
| GO:0005515 |
| GO:0005524 |
| GO:0005829 |
| GO:0007409 |
| GO:0014069 |
| GO:0019894 |
| GO:0019901 |
| GO:0030424 |
| GO:0030529 |
| GO:0030863 |
| GO:0030957 |
| GO:0035267 |
| GO:0043234 |
| GO:0050998 |
| GO:0070062 |
| GO:0070688 |
|
| **UCSC Genome Browser** |
| uc009ajk.1 |
|
| **WikiGenes** |
| 11461 |
|
| **Affy** |
| 101578\_f\_at |
| 10535381 |
| 1419734\_at |
| 1436722\_a\_at |
| 95705\_s\_at |
| AA590859\_f\_at |
| AA709861\_f\_at |
| AFFX-b-ActinMur/M12481\_3\_at |
| AFFX-b-ActinMur/M12481\_5\_at |
| AFFX-b-ActinMur/M12481\_M\_at |
| j04181\_f\_at |
