# Supplementary material for: Automatically visualise and analyse data on pathways using PathVisioRPC from any programming environment
Source: BMC Bioinformatics. 2015 Aug 23;16(1):267. doi: 10.1186/s12859-015-0708-8 (PMC4546821; doi:10.1186/s12859-015-0708-8)
Supplement: Additional file 3: — Examples in Python. This zip archive contains the data and python script for the three python examples. (ZIP 15714 kb) [file 12859_2015_708_MOESM3_ESM.zip › Python_Examples/result_Example_1/geneList2/backpage/L_11464.html]

 

# geneproduct annotation

  

| Name: Actc1| Identifier: 11464| Database: Entrez Gene| Synonyms: Actc-1 | | | --- | --- | | | | --- | --- | --- | --- | | | | --- | --- | --- | --- | --- | --- | | |
| --- | --- | --- | --- | --- | --- | --- | --- |

# Expression data

**Gene id on mapp: 11464**

| Sample name 11464| SystemCode L| LogFC 0.0| Pvalue 0.573740865| Type trans-PPS2 | | | --- | --- | | | | --- | --- | --- | --- | | | | --- | --- | --- | --- | --- | --- | | | | --- | --- | --- | --- | --- | --- | --- | --- | | |
| --- | --- | --- | --- | --- | --- | --- | --- | --- | --- |

  
  

---

  
  

# Cross references

  

|
|  |
| **UniGene** |
| Mm.686 |
|
| **Agilent** |
| A\_51\_P149469 |
| A\_55\_P2040951 |
|
| **Ensembl** |
| ENSMUSG00000068614 |
|
| **Illumina** |
| ILMN\_2598916 |
| ILMN\_2767216 |
|
| **Entrez Gene** |
| 11464 |
|
| **MGI** |
| MGI:87905 |
|
| **RefSeq** |
| NM\_009608 |
| NP\_033738 |
|
| **Uniprot/TrEMBL** |
| F6WX90 |
| P68033 |
| Q497E4 |
| Q61274 |
|
| **GeneOntology** |
| GO:0005515 |
| GO:0005524 |
| GO:0005737 |
| GO:0005884 |
| GO:0006200 |
| GO:0006915 |
| GO:0016887 |
| GO:0017022 |
| GO:0030017 |
| GO:0030048 |
| GO:0030240 |
| GO:0031032 |
| GO:0031674 |
| GO:0033275 |
| GO:0042493 |
| GO:0042643 |
| GO:0045471 |
| GO:0055003 |
| GO:0055008 |
| GO:0060047 |
| GO:0060048 |
| GO:0070062 |
|
| **UCSC Genome Browser** |
| uc008lpz.1 |
|
| **WikiGenes** |
| 11464 |
|
| **Affy** |
| 101028\_i\_at |
| 101029\_f\_at |
| 10485982 |
| 1415927\_at |
| Msa.34399.0\_f\_at |
| Msa.390.0\_f\_at |
| x03767\_f\_at |
