# Supplementary material for: Automatically visualise and analyse data on pathways using PathVisioRPC from any programming environment
Source: BMC Bioinformatics. 2015 Aug 23;16(1):267. doi: 10.1186/s12859-015-0708-8 (PMC4546821; doi:10.1186/s12859-015-0708-8)
Supplement: Additional file 3: — Examples in Python. This zip archive contains the data and python script for the three python examples. (ZIP 15714 kb) [file 12859_2015_708_MOESM3_ESM.zip › Python_Examples/result_Example_1/geneList2/backpage/L_11468.html]

 

# geneproduct annotation

  

| Name: Actg2| Identifier: 11468| Database: Entrez Gene| Synonyms: ACTA3 | | | --- | --- | | | | --- | --- | --- | --- | | | | --- | --- | --- | --- | --- | --- | | |
| --- | --- | --- | --- | --- | --- | --- | --- |

# Expression data

**Gene id on mapp: 11468**

| Sample name 11468| SystemCode L| LogFC 0.0| Pvalue 0.459856995| Type trans-PPS2 | | | --- | --- | | | | --- | --- | --- | --- | | | | --- | --- | --- | --- | --- | --- | | | | --- | --- | --- | --- | --- | --- | --- | --- | | |
| --- | --- | --- | --- | --- | --- | --- | --- | --- | --- |

  
  

---

  
  

# Cross references

  

|
|  |
| **UniGene** |
| Mm.292865 |
|
| **Agilent** |
| A\_51\_P241269 |
| A\_55\_P1963807 |
|
| **Ensembl** |
| ENSMUSG00000059430 |
|
| **Illumina** |
| ILMN\_2609642 |
| ILMN\_2839313 |
|
| **Entrez Gene** |
| 11468 |
|
| **MGI** |
| MGI:104589 |
|
| **RefSeq** |
| NM\_009610 |
| NP\_033740 |
|
| **Uniprot/TrEMBL** |
| D3YZY0 |
| D3Z2K3 |
| P63268 |
| Q3UJ36 |
|
| **GeneOntology** |
| GO:0005524 |
| GO:0005737 |
| GO:0005856 |
| GO:0071944 |
|
| **UCSC Genome Browser** |
| uc009cnr.1 |
| uc012eoa.1 |
|
| **WikiGenes** |
| 11468 |
|
| **Affy** |
| 10545707 |
| 1422340\_a\_at |
| 93102\_f\_at |
| Msa.1368.0\_f\_at |
| u20365\_f\_at |
