# Supplementary material for: Automatically visualise and analyse data on pathways using PathVisioRPC from any programming environment
Source: BMC Bioinformatics. 2015 Aug 23;16(1):267. doi: 10.1186/s12859-015-0708-8 (PMC4546821; doi:10.1186/s12859-015-0708-8)
Supplement: Additional file 3: — Examples in Python. This zip archive contains the data and python script for the three python examples. (ZIP 15714 kb) [file 12859_2015_708_MOESM3_ESM.zip › Python_Examples/result_Example_1/geneList2/backpage/L_11474.html]

 

# geneproduct annotation

  

| Name: Actn3| Identifier: 11474| Database: Entrez Gene | | | --- | --- | | | | --- | --- | --- | --- | | |
| --- | --- | --- | --- | --- | --- |

# Expression data

**Gene id on mapp: 11474**

| Sample name 11474| SystemCode L| LogFC 1.22237518| Pvalue 0.022374143| Type trans-PPS2 | | | --- | --- | | | | --- | --- | --- | --- | | | | --- | --- | --- | --- | --- | --- | | | | --- | --- | --- | --- | --- | --- | --- | --- | | |
| --- | --- | --- | --- | --- | --- | --- | --- | --- | --- |

  
  

---

  
  

# Cross references

  

|
|  |
| **UniGene** |
| Mm.5316 |
|
| **Agilent** |
| A\_52\_P656699 |
|
| **Ensembl** |
| ENSMUSG00000006457 |
|
| **Illumina** |
| ILMN\_2708303 |
| ILMN\_2747543 |
| ILMN\_3008110 |
|
| **Entrez Gene** |
| 11474 |
|
| **MGI** |
| MGI:99678 |
|
| **RefSeq** |
| NM\_013456 |
| NP\_038484 |
|
| **Uniprot/TrEMBL** |
| O88990 |
|
| **GeneOntology** |
| GO:0005509 |
| GO:0005515 |
| GO:0005865 |
| GO:0006936 |
| GO:0030017 |
| GO:0030018 |
| GO:0030674 |
| GO:0042803 |
| GO:0051015 |
| GO:0051017 |
| GO:0051764 |
|
| **UCSC Genome Browser** |
| uc008gbf.1 |
|
| **WikiGenes** |
| 11474 |
|
| **Affy** |
| 100879\_at |
| 10464836 |
| 1418677\_at |
| Msa.22717.0\_s\_at |
| Msa.41380.0\_s\_at |
| aa098356\_at |
| aa098356\_g\_at |
