# Supplementary material for: Automatically visualise and analyse data on pathways using PathVisioRPC from any programming environment
Source: BMC Bioinformatics. 2015 Aug 23;16(1):267. doi: 10.1186/s12859-015-0708-8 (PMC4546821; doi:10.1186/s12859-015-0708-8)
Supplement: Additional file 3: — Examples in Python. This zip archive contains the data and python script for the three python examples. (ZIP 15714 kb) [file 12859_2015_708_MOESM3_ESM.zip › Python_Examples/result_Example_1/geneList2/backpage/L_11477.html]

 

# geneproduct annotation

  

| Name: Acvr1| Identifier: 11477| Database: Entrez Gene| Synonyms: ActRIA | | | --- | --- | | | | --- | --- | --- | --- | | | | --- | --- | --- | --- | --- | --- | | |
| --- | --- | --- | --- | --- | --- | --- | --- |

# Expression data

**Gene id on mapp: 11477**

| Sample name 11477| SystemCode L| LogFC -1.221426299| Pvalue 0.046622247| Type trans-PPS2 | | | --- | --- | | | | --- | --- | --- | --- | | | | --- | --- | --- | --- | --- | --- | | | | --- | --- | --- | --- | --- | --- | --- | --- | | |
| --- | --- | --- | --- | --- | --- | --- | --- | --- | --- |

  
  

---

  
  

# Cross references

  

|
|  |
| **UniGene** |
| Mm.394526 |
| Mm.689 |
|
| **Agilent** |
| A\_52\_P352131 |
| A\_55\_P2102693 |
| A\_55\_P2159705 |
| A\_55\_P2159710 |
|
| **Ensembl** |
| ENSMUSG00000026836 |
|
| **Illumina** |
| ILMN\_1223443 |
| ILMN\_2442705 |
| ILMN\_2660103 |
|
| **Entrez Gene** |
| 11477 |
|
| **MGI** |
| MGI:87911 |
|
| **RefSeq** |
| NM\_001110204 |
| NM\_001110205 |
| NM\_007394 |
| NP\_001103674 |
| NP\_001103675 |
| NP\_031420 |
|
| **Uniprot/TrEMBL** |
| B1AW87 |
| P37172 |
|
| **GeneOntology** |
| GO:0000082 |
| GO:0001569 |
| GO:0001655 |
| GO:0001701 |
| GO:0001702 |
| GO:0001707 |
| GO:0001755 |
| GO:0002526 |
| GO:0003143 |
| GO:0003183 |
| GO:0003289 |
| GO:0004672 |
| GO:0004674 |
| GO:0004702 |
| GO:0005025 |
| GO:0005515 |
| GO:0005524 |
| GO:0005887 |
| GO:0006468 |
| GO:0007179 |
| GO:0007281 |
| GO:0007368 |
| GO:0007369 |
| GO:0007498 |
| GO:0007507 |
| GO:0009790 |
| GO:0009968 |
| GO:0010862 |
| GO:0016361 |
| GO:0018107 |
| GO:0019838 |
| GO:0023014 |
| GO:0030278 |
| GO:0030501 |
| GO:0030509 |
| GO:0032924 |
| GO:0032926 |
| GO:0042803 |
| GO:0043066 |
| GO:0045177 |
| GO:0045669 |
| GO:0045893 |
| GO:0045944 |
| GO:0046332 |
| GO:0046872 |
| GO:0048179 |
| GO:0048185 |
| GO:0048641 |
| GO:0050431 |
| GO:0051145 |
| GO:0060037 |
| GO:0060389 |
| GO:0060923 |
| GO:0061445 |
| GO:0071385 |
| GO:2000017 |
|
| **UCSC Genome Browser** |
| uc008jss.1 |
| uc008jst.1 |
| uc008jsu.1 |
|
| **WikiGenes** |
| 11477 |
|
| **Affy** |
| 10482824 |
| 1416786\_at |
| 1448460\_at |
| 163225\_at |
| 93460\_at |
| L15436\_s\_at |
