# Supplementary material for: Automatically visualise and analyse data on pathways using PathVisioRPC from any programming environment
Source: BMC Bioinformatics. 2015 Aug 23;16(1):267. doi: 10.1186/s12859-015-0708-8 (PMC4546821; doi:10.1186/s12859-015-0708-8)
Supplement: Additional file 3: — Examples in Python. This zip archive contains the data and python script for the three python examples. (ZIP 15714 kb) [file 12859_2015_708_MOESM3_ESM.zip › Python_Examples/result_Example_1/geneList2/backpage/L_11481.html]

 

# geneproduct annotation

  

| Name: Acvr2b| Identifier: 11481| Database: Entrez Gene| Synonyms: ActRIIB | | | --- | --- | | | | --- | --- | --- | --- | | | | --- | --- | --- | --- | --- | --- | | |
| --- | --- | --- | --- | --- | --- | --- | --- |

# Expression data

**Gene id on mapp: 11481**

| Sample name 11481| SystemCode L| LogFC 0.0| Pvalue 0.099384686| Type trans-PPS2 | | | --- | --- | | | | --- | --- | --- | --- | | | | --- | --- | --- | --- | --- | --- | | | | --- | --- | --- | --- | --- | --- | --- | --- | | |
| --- | --- | --- | --- | --- | --- | --- | --- | --- | --- |

  
  

---

  
  

# Cross references

  

|
|  |
| **Agilent** |
| A\_52\_P422618 |
| A\_55\_P2012815 |
|
| **Ensembl** |
| ENSMUSG00000061393 |
|
| **Illumina** |
| ILMN\_1230145 |
|
| **Entrez Gene** |
| 11481 |
|
| **MGI** |
| MGI:87912 |
|
| **PDB** |
| 1S4Y |
| 2H64 |
|
| **RefSeq** |
| NM\_007397 |
| NP\_031423 |
|
| **Uniprot/TrEMBL** |
| P27040 |
|
| **GeneOntology** |
| GO:0001501 |
| GO:0001702 |
| GO:0001822 |
| GO:0001946 |
| GO:0001974 |
| GO:0004674 |
| GO:0004702 |
| GO:0004712 |
| GO:0005024 |
| GO:0005515 |
| GO:0005524 |
| GO:0005737 |
| GO:0005886 |
| GO:0006355 |
| GO:0007165 |
| GO:0007368 |
| GO:0007389 |
| GO:0007498 |
| GO:0007507 |
| GO:0009749 |
| GO:0009791 |
| GO:0009952 |
| GO:0009966 |
| GO:0009986 |
| GO:0016021 |
| GO:0017002 |
| GO:0019838 |
| GO:0030073 |
| GO:0030324 |
| GO:0030501 |
| GO:0030509 |
| GO:0031016 |
| GO:0032147 |
| GO:0032927 |
| GO:0034711 |
| GO:0035265 |
| GO:0042475 |
| GO:0045669 |
| GO:0046872 |
| GO:0048185 |
| GO:0048617 |
| GO:0048705 |
| GO:0060021 |
| GO:0060836 |
| GO:0060840 |
| GO:0060841 |
| GO:0061298 |
|
| **UCSC Genome Browser** |
| uc009sax.1 |
|
| **WikiGenes** |
| 11481 |
|
| **Affy** |
| 10590169 |
| 1419140\_at |
| 93903\_at |
| Msa.403.0\_at |
| Msa.403.0\_g\_at |
