# Supplementary material for: Automatically visualise and analyse data on pathways using PathVisioRPC from any programming environment
Source: BMC Bioinformatics. 2015 Aug 23;16(1):267. doi: 10.1186/s12859-015-0708-8 (PMC4546821; doi:10.1186/s12859-015-0708-8)
Supplement: Additional file 3: — Examples in Python. This zip archive contains the data and python script for the three python examples. (ZIP 15714 kb) [file 12859_2015_708_MOESM3_ESM.zip › Python_Examples/result_Example_1/geneList2/backpage/L_11482.html]

 

# geneproduct annotation

  

| Name: Acvrl1| Identifier: 11482| Database: Entrez Gene| Synonyms: Acvrlk1 | | | --- | --- | | | | --- | --- | --- | --- | | | | --- | --- | --- | --- | --- | --- | | |
| --- | --- | --- | --- | --- | --- | --- | --- |

# Expression data

**Gene id on mapp: 11482**

| Sample name 11482| SystemCode L| LogFC 0.0| Pvalue 0.917430518| Type trans-PPS2 | | | --- | --- | | | | --- | --- | --- | --- | | | | --- | --- | --- | --- | --- | --- | | | | --- | --- | --- | --- | --- | --- | --- | --- | | |
| --- | --- | --- | --- | --- | --- | --- | --- | --- | --- |

  
  

---

  
  

# Cross references

  

|
|  |
| **UniGene** |
| Mm.279542 |
|
| **Agilent** |
| A\_52\_P279329 |
| A\_55\_P2011380 |
|
| **Ensembl** |
| ENSMUSG00000000530 |
|
| **Illumina** |
| ILMN\_1248328 |
|
| **Entrez Gene** |
| 11482 |
|
| **MGI** |
| MGI:1338946 |
|
| **RefSeq** |
| NM\_001277255 |
| NM\_001277257 |
| NM\_001277258 |
| NM\_001277259 |
| NM\_009612 |
| NP\_001264184 |
| NP\_001264186 |
| NP\_001264187 |
| NP\_001264188 |
| NP\_033742 |
|
| **Uniprot/TrEMBL** |
| D3YUY5 |
| D3Z7H9 |
| Q61288 |
| Q9CU19 |
|
| **GeneOntology** |
| GO:0001525 |
| GO:0001701 |
| GO:0001937 |
| GO:0001938 |
| GO:0001946 |
| GO:0001974 |
| GO:0004674 |
| GO:0004702 |
| GO:0005024 |
| GO:0005025 |
| GO:0005515 |
| GO:0005524 |
| GO:0005887 |
| GO:0006355 |
| GO:0006468 |
| GO:0007162 |
| GO:0007165 |
| GO:0007179 |
| GO:0008015 |
| GO:0008217 |
| GO:0008285 |
| GO:0009986 |
| GO:0010596 |
| GO:0010862 |
| GO:0019901 |
| GO:0023014 |
| GO:0030308 |
| GO:0030336 |
| GO:0030425 |
| GO:0030509 |
| GO:0030513 |
| GO:0035313 |
| GO:0042118 |
| GO:0043025 |
| GO:0043537 |
| GO:0045602 |
| GO:0045603 |
| GO:0045893 |
| GO:0045944 |
| GO:0046332 |
| GO:0046872 |
| GO:0048185 |
| GO:0048514 |
| GO:0050431 |
| GO:0051291 |
| GO:0051895 |
| GO:0060836 |
| GO:0060840 |
| GO:0060841 |
| GO:0061154 |
| GO:0061298 |
| GO:0071560 |
| GO:0071773 |
| GO:2000279 |
|
| **UCSC Genome Browser** |
| uc007xsm.1 |
| uc007xsn.1 |
| uc007xso.1 |
| uc007xsq.1 |
|
| **WikiGenes** |
| 11482 |
|
| **Affy** |
| 100448\_at |
| 100449\_g\_at |
| 100450\_r\_at |
| 10426999 |
| 1435825\_at |
| 1451604\_a\_at |
| 163190\_at |
| l48015\_s\_at |
