# Supplementary material for: Automatically visualise and analyse data on pathways using PathVisioRPC from any programming environment
Source: BMC Bioinformatics. 2015 Aug 23;16(1):267. doi: 10.1186/s12859-015-0708-8 (PMC4546821; doi:10.1186/s12859-015-0708-8)
Supplement: Additional file 3: — Examples in Python. This zip archive contains the data and python script for the three python examples. (ZIP 15714 kb) [file 12859_2015_708_MOESM3_ESM.zip › Python_Examples/result_Example_1/geneList2/backpage/L_11484.html]

 

# geneproduct annotation

  

| Name: Aspa| Identifier: 11484| Database: Entrez Gene| Synonyms: Acy2 | | | --- | --- | | | | --- | --- | --- | --- | | | | --- | --- | --- | --- | --- | --- | | |
| --- | --- | --- | --- | --- | --- | --- | --- |

# Expression data

**Gene id on mapp: 11484**

| Sample name 11484| SystemCode L| LogFC 1.82255835| Pvalue 4.42E-5| Type trans-PPS2 | | | --- | --- | | | | --- | --- | --- | --- | | | | --- | --- | --- | --- | --- | --- | | | | --- | --- | --- | --- | --- | --- | --- | --- | | |
| --- | --- | --- | --- | --- | --- | --- | --- | --- | --- |

  
  

---

  
  

# Cross references

  

|
|  |
| **UniGene** |
| Mm.293574 |
|
| **Agilent** |
| A\_51\_P259968 |
| A\_51\_P259975 |
|
| **Ensembl** |
| ENSMUSG00000020774 |
|
| **Illumina** |
| ILMN\_1214573 |
| ILMN\_1234740 |
|
| **Entrez Gene** |
| 11484 |
|
| **MGI** |
| MGI:87914 |
|
| **RefSeq** |
| NM\_023113 |
| NP\_075602 |
|
| **Uniprot/TrEMBL** |
| B0QZP3 |
| D6RJ20 |
| Q8R3P0 |
|
| **GeneOntology** |
| GO:0004046 |
| GO:0005634 |
| GO:0005737 |
| GO:0006083 |
| GO:0016788 |
| GO:0019807 |
| GO:0022010 |
| GO:0046872 |
| GO:0048714 |
|
| **UCSC Genome Browser** |
| uc007kal.1 |
| uc007kam.1 |
|
| **WikiGenes** |
| 11484 |
|
| **Affy** |
| 10388254 |
| 115745\_at |
| 1418472\_at |
