# Supplementary material for: Automatically visualise and analyse data on pathways using PathVisioRPC from any programming environment
Source: BMC Bioinformatics. 2015 Aug 23;16(1):267. doi: 10.1186/s12859-015-0708-8 (PMC4546821; doi:10.1186/s12859-015-0708-8)
Supplement: Additional file 3: — Examples in Python. This zip archive contains the data and python script for the three python examples. (ZIP 15714 kb) [file 12859_2015_708_MOESM3_ESM.zip › Python_Examples/result_Example_1/geneList2/backpage/L_11486.html]

 

# geneproduct annotation

  

| Name: Ada| Identifier: 11486| Database: Entrez Gene | | | --- | --- | | | | --- | --- | --- | --- | | |
| --- | --- | --- | --- | --- | --- |

# Expression data

**Gene id on mapp: 11486**

| Sample name 11486| SystemCode L| LogFC -1.291656857| Pvalue 0.020934716| Type trans-PPS2 | | | --- | --- | | | | --- | --- | --- | --- | | | | --- | --- | --- | --- | --- | --- | | | | --- | --- | --- | --- | --- | --- | --- | --- | | |
| --- | --- | --- | --- | --- | --- | --- | --- | --- | --- |

  
  

---

  
  

# Cross references

  

|
|  |
| **UniGene** |
| Mm.388 |
| Mm.491289 |
|
| **Agilent** |
| A\_51\_P430423 |
|
| **Ensembl** |
| ENSMUSG00000017697 |
|
| **Illumina** |
| ILMN\_1228696 |
| ILMN\_2686132 |
|
| **Entrez Gene** |
| 11486 |
|
| **MGI** |
| MGI:87916 |
|
| **PDB** |
| 1A4L |
| 1A4M |
| 1ADD |
| 1FKW |
| 1FKX |
| 1UIO |
| 1UIP |
| 2ADA |
| 3KM8 |
| 3MVI |
| 3MVT |
| 3T1G |
|
| **RefSeq** |
| NM\_001272052 |
| NM\_007398 |
| NP\_001258981 |
| NP\_031424 |
|
| **Uniprot/TrEMBL** |
| P03958 |
| Q4FK28 |
|
| **GeneOntology** |
| GO:0001666 |
| GO:0001701 |
| GO:0001821 |
| GO:0001829 |
| GO:0001883 |
| GO:0001889 |
| GO:0001890 |
| GO:0002314 |
| GO:0002636 |
| GO:0002686 |
| GO:0002906 |
| GO:0004000 |
| GO:0005515 |
| GO:0005615 |
| GO:0005737 |
| GO:0005764 |
| GO:0005829 |
| GO:0005886 |
| GO:0006154 |
| GO:0006157 |
| GO:0007155 |
| GO:0007568 |
| GO:0008270 |
| GO:0009168 |
| GO:0009897 |
| GO:0009986 |
| GO:0010460 |
| GO:0016020 |
| GO:0030054 |
| GO:0030324 |
| GO:0030890 |
| GO:0032261 |
| GO:0032839 |
| GO:0033089 |
| GO:0033632 |
| GO:0042110 |
| GO:0042323 |
| GO:0042542 |
| GO:0043025 |
| GO:0043066 |
| GO:0043103 |
| GO:0043278 |
| GO:0045187 |
| GO:0045580 |
| GO:0045582 |
| GO:0045987 |
| GO:0046061 |
| GO:0046101 |
| GO:0046103 |
| GO:0046111 |
| GO:0046638 |
| GO:0048286 |
| GO:0048541 |
| GO:0048566 |
| GO:0050728 |
| GO:0050850 |
| GO:0050862 |
| GO:0050870 |
| GO:0060169 |
| GO:0060205 |
| GO:0060407 |
| GO:0070244 |
| GO:0070256 |
|
| **UCSC Genome Browser** |
| uc008ntl.1 |
|
| **WikiGenes** |
| 11486 |
|
| **Affy** |
| 10489391 |
| 1417976\_at |
| 93205\_at |
| 93206\_g\_at |
| 98632\_at |
| m10319\_s\_at |
