# Supplementary material for: Automatically visualise and analyse data on pathways using PathVisioRPC from any programming environment
Source: BMC Bioinformatics. 2015 Aug 23;16(1):267. doi: 10.1186/s12859-015-0708-8 (PMC4546821; doi:10.1186/s12859-015-0708-8)
Supplement: Additional file 3: — Examples in Python. This zip archive contains the data and python script for the three python examples. (ZIP 15714 kb) [file 12859_2015_708_MOESM3_ESM.zip › Python_Examples/result_Example_1/geneList2/backpage/L_11487.html]

 

# geneproduct annotation

  

| Name: Adam10| Identifier: 11487| Database: Entrez Gene| Synonyms: kuzbanian | | | --- | --- | | | | --- | --- | --- | --- | | | | --- | --- | --- | --- | --- | --- | | |
| --- | --- | --- | --- | --- | --- | --- | --- |

# Expression data

**Gene id on mapp: 11487**

| Sample name 11487| SystemCode L| LogFC -1.384588662| Pvalue 0.003074423| Type trans-PPS2 | | | --- | --- | | | | --- | --- | --- | --- | | | | --- | --- | --- | --- | --- | --- | | | | --- | --- | --- | --- | --- | --- | --- | --- | | |
| --- | --- | --- | --- | --- | --- | --- | --- | --- | --- |

  
  

---

  
  

# Cross references

  

|
|  |
| **UniGene** |
| Mm.3037 |
| Mm.409520 |
| Mm.410323 |
| Mm.473247 |
|
| **Agilent** |
| A\_55\_P1967216 |
|
| **Ensembl** |
| ENSMUSG00000054693 |
|
| **Illumina** |
| ILMN\_2460257 |
|
| **Entrez Gene** |
| 11487 |
|
| **MGI** |
| MGI:109548 |
|
| **RefSeq** |
| NM\_007399 |
| NP\_031425 |
|
| **Uniprot/TrEMBL** |
| B3SRB2 |
| D3Z1E6 |
| E9PYF2 |
| O35598 |
|
| **GeneOntology** |
| GO:0001701 |
| GO:0004175 |
| GO:0004222 |
| GO:0005515 |
| GO:0005634 |
| GO:0005737 |
| GO:0005794 |
| GO:0005798 |
| GO:0005886 |
| GO:0006468 |
| GO:0006509 |
| GO:0006913 |
| GO:0007162 |
| GO:0007219 |
| GO:0007220 |
| GO:0008237 |
| GO:0008270 |
| GO:0008284 |
| GO:0009986 |
| GO:0010820 |
| GO:0014069 |
| GO:0016021 |
| GO:0017124 |
| GO:0019901 |
| GO:0030307 |
| GO:0030335 |
| GO:0034612 |
| GO:0042117 |
| GO:0042169 |
| GO:0042803 |
| GO:0051088 |
| GO:0051089 |
|
| **UCSC Genome Browser** |
| uc009qor.1 |
|
| **WikiGenes** |
| 11487 |
|
| **Affy** |
| 100751\_at |
| 10586844 |
| 1428103\_at |
| 1450104\_at |
| 1450105\_at |
| 97947\_at |
| AF011379\_at |
| aa500688\_s\_at |
