# Supplementary material for: Automatically visualise and analyse data on pathways using PathVisioRPC from any programming environment
Source: BMC Bioinformatics. 2015 Aug 23;16(1):267. doi: 10.1186/s12859-015-0708-8 (PMC4546821; doi:10.1186/s12859-015-0708-8)
Supplement: Additional file 3: — Examples in Python. This zip archive contains the data and python script for the three python examples. (ZIP 15714 kb) [file 12859_2015_708_MOESM3_ESM.zip › Python_Examples/result_Example_1/geneList2/backpage/L_11488.html]

 

# geneproduct annotation

  

| Name: Adam11| Identifier: 11488| Database: Entrez Gene| Synonyms: AW060611 | | | --- | --- | | | | --- | --- | --- | --- | | | | --- | --- | --- | --- | --- | --- | | |
| --- | --- | --- | --- | --- | --- | --- | --- |

# Expression data

**Gene id on mapp: 11488**

| Sample name 11488| SystemCode L| LogFC 0.0| Pvalue 0.120966029| Type trans-PPS2 | | | --- | --- | | | | --- | --- | --- | --- | | | | --- | --- | --- | --- | --- | --- | | | | --- | --- | --- | --- | --- | --- | --- | --- | | |
| --- | --- | --- | --- | --- | --- | --- | --- | --- | --- |

  
  

---

  
  

# Cross references

  

|
|  |
| **UniGene** |
| Mm.89854 |
|
| **Agilent** |
| A\_51\_P130773 |
| A\_51\_P419666 |
| A\_52\_P205255 |
| A\_55\_P1962876 |
|
| **Ensembl** |
| ENSMUSG00000020926 |
|
| **Illumina** |
| ILMN\_2434200 |
| ILMN\_2629383 |
| ILMN\_2812954 |
|
| **Entrez Gene** |
| 11488 |
|
| **MGI** |
| MGI:1098667 |
|
| **RefSeq** |
| NM\_001110778 |
| NM\_009613 |
| NP\_001104248 |
| NP\_033743 |
|
| **Uniprot/TrEMBL** |
| Q7TQG7 |
| Q9R1V4 |
|
| **GeneOntology** |
| GO:0004222 |
| GO:0005515 |
| GO:0006508 |
| GO:0007229 |
| GO:0008270 |
| GO:0016021 |
|
| **UCSC Genome Browser** |
| uc007lsi.2 |
| uc007lsj.2 |
|
| **WikiGenes** |
| 11488 |
|
| **Affy** |
| 10381619 |
| 116554\_at |
| 1450248\_at |
| 1455270\_at |
| 93920\_at |
