# Supplementary material for: Automatically visualise and analyse data on pathways using PathVisioRPC from any programming environment
Source: BMC Bioinformatics. 2015 Aug 23;16(1):267. doi: 10.1186/s12859-015-0708-8 (PMC4546821; doi:10.1186/s12859-015-0708-8)
Supplement: Additional file 3: — Examples in Python. This zip archive contains the data and python script for the three python examples. (ZIP 15714 kb) [file 12859_2015_708_MOESM3_ESM.zip › Python_Examples/result_Example_1/geneList2/backpage/L_11489.html]

 

# geneproduct annotation

  

| Name: Adam12| Identifier: 11489| Database: Entrez Gene| Synonyms: Mltna | | | --- | --- | | | | --- | --- | --- | --- | | | | --- | --- | --- | --- | --- | --- | | |
| --- | --- | --- | --- | --- | --- | --- | --- |

# Expression data

**Gene id on mapp: 11489**

| Sample name 11489| SystemCode L| LogFC 1.139273603| Pvalue 0.026670059| Type trans-PPS2 | | | --- | --- | | | | --- | --- | --- | --- | | | | --- | --- | --- | --- | --- | --- | | | | --- | --- | --- | --- | --- | --- | --- | --- | | |
| --- | --- | --- | --- | --- | --- | --- | --- | --- | --- |

  
  

---

  
  

# Cross references

  

|
|  |
| **UniGene** |
| Mm.439714 |
|
| **Agilent** |
| A\_51\_P510882 |
| A\_52\_P290457 |
| A\_55\_P1958823 |
| A\_55\_P2045571 |
|
| **Ensembl** |
| ENSMUSG00000054555 |
|
| **Illumina** |
| ILMN\_1243254 |
| ILMN\_2671330 |
|
| **Entrez Gene** |
| 11489 |
|
| **MGI** |
| MGI:105378 |
|
| **RefSeq** |
| NM\_007400 |
| NP\_031426 |
|
| **Uniprot/TrEMBL** |
| D3YUK3 |
| D3Z117 |
| F6YWH6 |
| Q571B5 |
| Q61824 |
|
| **GeneOntology** |
| GO:0004222 |
| GO:0005515 |
| GO:0005634 |
| GO:0005739 |
| GO:0005886 |
| GO:0006508 |
| GO:0007155 |
| GO:0008270 |
| GO:0016021 |
| GO:0017124 |
|
| **UCSC Genome Browser** |
| uc009kdo.2 |
| uc009kdp.2 |
|
| **WikiGenes** |
| 11489 |
|
| **Affy** |
| 10568668 |
| 134907\_at |
| 1421171\_at |
| 1421172\_at |
| 92414\_at |
| D50411\_s\_at |
