# Supplementary material for: Automatically visualise and analyse data on pathways using PathVisioRPC from any programming environment
Source: BMC Bioinformatics. 2015 Aug 23;16(1):267. doi: 10.1186/s12859-015-0708-8 (PMC4546821; doi:10.1186/s12859-015-0708-8)
Supplement: Additional file 3: — Examples in Python. This zip archive contains the data and python script for the three python examples. (ZIP 15714 kb) [file 12859_2015_708_MOESM3_ESM.zip › Python_Examples/result_Example_1/geneList2/backpage/L_11490.html]

 

# geneproduct annotation

  

| Name: Adam15| Identifier: 11490| Database: Entrez Gene| Synonyms: metargidin | | | --- | --- | | | | --- | --- | --- | --- | | | | --- | --- | --- | --- | --- | --- | | |
| --- | --- | --- | --- | --- | --- | --- | --- |

# Expression data

**Gene id on mapp: 11490**

| Sample name 11490| SystemCode L| LogFC 0.0| Pvalue 0.556974633| Type trans-PPS2 | | | --- | --- | | | | --- | --- | --- | --- | | | | --- | --- | --- | --- | --- | --- | | | | --- | --- | --- | --- | --- | --- | --- | --- | | |
| --- | --- | --- | --- | --- | --- | --- | --- | --- | --- |

  
  

---

  
  

# Cross references

  

|
|  |
| **UniGene** |
| Mm.274049 |
| Mm.416037 |
|
| **Agilent** |
| A\_51\_P291815 |
| A\_52\_P362670 |
| A\_55\_P2055819 |
|
| **Ensembl** |
| ENSMUSG00000028041 |
|
| **Illumina** |
| ILMN\_1236859 |
| ILMN\_1239236 |
| ILMN\_1240629 |
| ILMN\_2551988 |
| ILMN\_2718141 |
| ILMN\_2718144 |
| ILMN\_3139103 |
|
| **Entrez Gene** |
| 11490 |
|
| **MGI** |
| MGI:1333882 |
|
| **RefSeq** |
| NM\_001037722 |
| NM\_009614 |
| NP\_001032811 |
| NP\_033744 |
|
| **Uniprot/TrEMBL** |
| O88839 |
|
| **GeneOntology** |
| GO:0001525 |
| GO:0001669 |
| GO:0004222 |
| GO:0005515 |
| GO:0005912 |
| GO:0006508 |
| GO:0007155 |
| GO:0008237 |
| GO:0008270 |
| GO:0012505 |
| GO:0016021 |
| GO:0017124 |
| GO:0030574 |
| GO:0042246 |
| GO:0042995 |
| GO:0060317 |
|
| **UCSC Genome Browser** |
| uc008pyv.1 |
| uc008pyw.1 |
|
| **WikiGenes** |
| 11490 |
|
| **Affy** |
| 103588\_at |
| 10499560 |
| 1416080\_at |
| 1425170\_a\_at |
| 1438760\_x\_at |
| 1454206\_a\_at |
| Msa.32534.0\_s\_at |
| aa445319\_s\_at |
