# Supplementary material for: Automatically visualise and analyse data on pathways using PathVisioRPC from any programming environment
Source: BMC Bioinformatics. 2015 Aug 23;16(1):267. doi: 10.1186/s12859-015-0708-8 (PMC4546821; doi:10.1186/s12859-015-0708-8)
Supplement: Additional file 3: — Examples in Python. This zip archive contains the data and python script for the three python examples. (ZIP 15714 kb) [file 12859_2015_708_MOESM3_ESM.zip › Python_Examples/result_Example_1/geneList2/backpage/L_11491.html]

 

# geneproduct annotation

  

| Name: Adam17| Identifier: 11491| Database: Entrez Gene| Synonyms: Tace | | | --- | --- | | | | --- | --- | --- | --- | | | | --- | --- | --- | --- | --- | --- | | |
| --- | --- | --- | --- | --- | --- | --- | --- |

# Expression data

**Gene id on mapp: 11491**

| Sample name 11491| SystemCode L| LogFC -1.348015475| Pvalue 0.009401224| Type trans-PPS2 | | | --- | --- | | | | --- | --- | --- | --- | | | | --- | --- | --- | --- | --- | --- | | | | --- | --- | --- | --- | --- | --- | --- | --- | | |
| --- | --- | --- | --- | --- | --- | --- | --- | --- | --- |

  
  

---

  
  

# Cross references

  

|
|  |
| **UniGene** |
| Mm.27681 |
|
| **Agilent** |
| A\_52\_P337050 |
| A\_52\_P734595 |
| A\_55\_P1999097 |
| A\_55\_P2128188 |
|
| **Ensembl** |
| ENSMUSG00000052593 |
|
| **Illumina** |
| ILMN\_1243942 |
| ILMN\_2594718 |
|
| **Entrez Gene** |
| 11491 |
|
| **MGI** |
| MGI:1096335 |
|
| **RefSeq** |
| NM\_001277266 |
| NM\_009615 |
| NP\_001264195 |
| NP\_033745 |
|
| **Uniprot/TrEMBL** |
| E9PXU2 |
| J3QMF2 |
| J3QNB3 |
| Q3UEC0 |
| Q9Z0F8 |
|
| **GeneOntology** |
| GO:0001666 |
| GO:0001934 |
| GO:0002467 |
| GO:0004222 |
| GO:0005138 |
| GO:0005178 |
| GO:0005515 |
| GO:0005576 |
| GO:0005737 |
| GO:0005886 |
| GO:0005887 |
| GO:0005911 |
| GO:0005925 |
| GO:0006508 |
| GO:0006509 |
| GO:0007155 |
| GO:0007173 |
| GO:0007219 |
| GO:0007229 |
| GO:0008237 |
| GO:0008270 |
| GO:0008284 |
| GO:0009986 |
| GO:0010820 |
| GO:0015629 |
| GO:0016324 |
| GO:0017124 |
| GO:0030165 |
| GO:0030183 |
| GO:0030307 |
| GO:0030335 |
| GO:0030511 |
| GO:0031659 |
| GO:0032496 |
| GO:0032587 |
| GO:0032717 |
| GO:0032722 |
| GO:0033025 |
| GO:0033077 |
| GO:0033627 |
| GO:0035313 |
| GO:0035625 |
| GO:0042493 |
| GO:0045121 |
| GO:0045741 |
| GO:0048536 |
| GO:0048870 |
| GO:0051088 |
| GO:0051272 |
| GO:0055099 |
|
| **UCSC Genome Browser** |
| uc007ndu.1 |
| uc011yks.1 |
|
| **WikiGenes** |
| 11491 |
|
| **Affy** |
| 10399605 |
| 133576\_at |
| 1421857\_at |
| 1421858\_at |
| 1421859\_at |
| c76813\_rc\_s\_at |
