# Supplementary material for: Automatically visualise and analyse data on pathways using PathVisioRPC from any programming environment
Source: BMC Bioinformatics. 2015 Aug 23;16(1):267. doi: 10.1186/s12859-015-0708-8 (PMC4546821; doi:10.1186/s12859-015-0708-8)
Supplement: Additional file 3: — Examples in Python. This zip archive contains the data and python script for the three python examples. (ZIP 15714 kb) [file 12859_2015_708_MOESM3_ESM.zip › Python_Examples/result_Example_1/geneList2/backpage/L_11492.html]

 

# geneproduct annotation

  

| Name: Adam19| Identifier: 11492| Database: Entrez Gene| Synonyms: Mltnb | | | --- | --- | | | | --- | --- | --- | --- | | | | --- | --- | --- | --- | --- | --- | | |
| --- | --- | --- | --- | --- | --- | --- | --- |

# Expression data

**Gene id on mapp: 11492**

| Sample name 11492| SystemCode L| LogFC 0.0| Pvalue 0.297925073| Type trans-PPS2 | | | --- | --- | | | | --- | --- | --- | --- | | | | --- | --- | --- | --- | --- | --- | | | | --- | --- | --- | --- | --- | --- | --- | --- | | |
| --- | --- | --- | --- | --- | --- | --- | --- | --- | --- |

  
  

---

  
  

# Cross references

  

|
|  |
| **UniGene** |
| Mm.89940 |
|
| **Agilent** |
| A\_51\_P267447 |
| A\_52\_P280044 |
| A\_52\_P396459 |
| A\_55\_P1983095 |
|
| **Ensembl** |
| ENSMUSG00000011256 |
|
| **Illumina** |
| ILMN\_2772155 |
|
| **Entrez Gene** |
| 11492 |
|
| **MGI** |
| MGI:105377 |
|
| **RefSeq** |
| NM\_009616 |
| NP\_033746 |
|
| **Uniprot/TrEMBL** |
| O35674 |
| Q3UHT3 |
|
| **GeneOntology** |
| GO:0004222 |
| GO:0005515 |
| GO:0006509 |
| GO:0007507 |
| GO:0008270 |
| GO:0016021 |
| GO:0017124 |
|
| **UCSC Genome Browser** |
| uc007iny.1 |
|
| **WikiGenes** |
| 11492 |
|
| **Affy** |
| 103554\_at |
| 10375402 |
| 140584\_f\_at |
| 1418402\_at |
| 1418403\_at |
| aa726223\_at |
| aa726223\_g\_at |
