# Supplementary material for: Automatically visualise and analyse data on pathways using PathVisioRPC from any programming environment
Source: BMC Bioinformatics. 2015 Aug 23;16(1):267. doi: 10.1186/s12859-015-0708-8 (PMC4546821; doi:10.1186/s12859-015-0708-8)
Supplement: Additional file 3: — Examples in Python. This zip archive contains the data and python script for the three python examples. (ZIP 15714 kb) [file 12859_2015_708_MOESM3_ESM.zip › Python_Examples/result_Example_1/geneList2/backpage/L_11501.html]

 

# geneproduct annotation

  

| Name: Adam8| Identifier: 11501| Database: Entrez Gene| Synonyms: CD156 | | | --- | --- | | | | --- | --- | --- | --- | | | | --- | --- | --- | --- | --- | --- | | |
| --- | --- | --- | --- | --- | --- | --- | --- |

# Expression data

**Gene id on mapp: 11501**

| Sample name 11501| SystemCode L| LogFC 0.0| Pvalue 0.949106642| Type trans-PPS2 | | | --- | --- | | | | --- | --- | --- | --- | | | | --- | --- | --- | --- | --- | --- | | | | --- | --- | --- | --- | --- | --- | --- | --- | | |
| --- | --- | --- | --- | --- | --- | --- | --- | --- | --- |

  
  

---

  
  

# Cross references

  

|
|  |
| **UniGene** |
| Mm.15969 |
|
| **Agilent** |
| A\_51\_P319917 |
| A\_55\_P2025765 |
|
| **Ensembl** |
| ENSMUSG00000025473 |
|
| **Illumina** |
| ILMN\_1224005 |
| ILMN\_1256561 |
| ILMN\_2756613 |
|
| **Entrez Gene** |
| 11501 |
|
| **MGI** |
| MGI:107825 |
|
| **RefSeq** |
| NM\_007403 |
| NP\_031429 |
|
| **Uniprot/TrEMBL** |
| E9Q359 |
| G3UXF8 |
| Q05910 |
| Q3U1J7 |
| Q3U7G2 |
|
| **GeneOntology** |
| GO:0000902 |
| GO:0001525 |
| GO:0002102 |
| GO:0002523 |
| GO:0002675 |
| GO:0002693 |
| GO:0004222 |
| GO:0005178 |
| GO:0005509 |
| GO:0005515 |
| GO:0005737 |
| GO:0005886 |
| GO:0005887 |
| GO:0006508 |
| GO:0006944 |
| GO:0006954 |
| GO:0007160 |
| GO:0008237 |
| GO:0008270 |
| GO:0009986 |
| GO:0016337 |
| GO:0022407 |
| GO:0032010 |
| GO:0032127 |
| GO:0033089 |
| GO:0035419 |
| GO:0042581 |
| GO:0043524 |
| GO:0043534 |
| GO:0045670 |
| GO:0045780 |
| GO:0045785 |
| GO:0048247 |
| GO:0048729 |
| GO:0050729 |
| GO:0050839 |
| GO:0051044 |
| GO:0051092 |
| GO:0051897 |
| GO:0070245 |
| GO:0070820 |
| GO:0071065 |
| GO:0071133 |
| GO:0071456 |
| GO:0072675 |
| GO:2000309 |
| GO:2000391 |
| GO:2000399 |
| GO:2000406 |
| GO:2000415 |
| GO:2000418 |
|
| **UCSC Genome Browser** |
| uc009kgj.1 |
| uc009kgk.1 |
| uc012fwc.1 |
|
| **WikiGenes** |
| 11501 |
|
| **Affy** |
| 103024\_at |
| 10568873 |
| 1416871\_at |
| d10911\_s\_at |
