# Supplementary material for: Automatically visualise and analyse data on pathways using PathVisioRPC from any programming environment
Source: BMC Bioinformatics. 2015 Aug 23;16(1):267. doi: 10.1186/s12859-015-0708-8 (PMC4546821; doi:10.1186/s12859-015-0708-8)
Supplement: Additional file 3: — Examples in Python. This zip archive contains the data and python script for the three python examples. (ZIP 15714 kb) [file 12859_2015_708_MOESM3_ESM.zip › Python_Examples/result_Example_1/geneList2/backpage/L_11502.html]

 

# geneproduct annotation

  

| Name: Adam9| Identifier: 11502| Database: Entrez Gene| Synonyms: AU020942 | | | --- | --- | | | | --- | --- | --- | --- | | | | --- | --- | --- | --- | --- | --- | | |
| --- | --- | --- | --- | --- | --- | --- | --- |

# Expression data

**Gene id on mapp: 11502**

| Sample name 11502| SystemCode L| LogFC 0.0| Pvalue 0.813924287| Type trans-PPS2 | | | --- | --- | | | | --- | --- | --- | --- | | | | --- | --- | --- | --- | --- | --- | | | | --- | --- | --- | --- | --- | --- | --- | --- | | |
| --- | --- | --- | --- | --- | --- | --- | --- | --- | --- |

  
  

---

  
  

# Cross references

  

|
|  |
| **UniGene** |
| Mm.407298 |
|
| **Agilent** |
| A\_51\_P518378 |
| A\_55\_P2025760 |
|
| **Ensembl** |
| ENSMUSG00000031555 |
|
| **Illumina** |
| ILMN\_2649502 |
|
| **Entrez Gene** |
| 11502 |
|
| **MGI** |
| MGI:105376 |
|
| **RefSeq** |
| NM\_007404 |
| NP\_001257925 |
| NP\_031430 |
|
| **Uniprot/TrEMBL** |
| E9Q638 |
| Q61072 |
|
| **GeneOntology** |
| GO:0000186 |
| GO:0004222 |
| GO:0005080 |
| GO:0005178 |
| GO:0005515 |
| GO:0005518 |
| GO:0005615 |
| GO:0005737 |
| GO:0005886 |
| GO:0006508 |
| GO:0006509 |
| GO:0007155 |
| GO:0007160 |
| GO:0007179 |
| GO:0008237 |
| GO:0008270 |
| GO:0009986 |
| GO:0010042 |
| GO:0016021 |
| GO:0017124 |
| GO:0030216 |
| GO:0031233 |
| GO:0033627 |
| GO:0033630 |
| GO:0033631 |
| GO:0034241 |
| GO:0034612 |
| GO:0042117 |
| GO:0042542 |
| GO:0043236 |
| GO:0050714 |
| GO:0051044 |
| GO:0051088 |
| GO:0051384 |
| GO:0051549 |
| GO:0051592 |
| GO:0071222 |
|
| **UCSC Genome Browser** |
| uc009lfl.2 |
|
| **WikiGenes** |
| 11502 |
|
| **Affy** |
| 10577757 |
| 1416094\_at |
| 96738\_at |
| U41765\_s\_at |
