# Supplementary material for: Automatically visualise and analyse data on pathways using PathVisioRPC from any programming environment
Source: BMC Bioinformatics. 2015 Aug 23;16(1):267. doi: 10.1186/s12859-015-0708-8 (PMC4546821; doi:10.1186/s12859-015-0708-8)
Supplement: Additional file 3: — Examples in Python. This zip archive contains the data and python script for the three python examples. (ZIP 15714 kb) [file 12859_2015_708_MOESM3_ESM.zip › Python_Examples/result_Example_1/geneList2/backpage/L_11504.html]

 

# geneproduct annotation

  

| Name: Adamts1| Identifier: 11504| Database: Entrez Gene| Synonyms: C3-C5 | | | --- | --- | | | | --- | --- | --- | --- | | | | --- | --- | --- | --- | --- | --- | | |
| --- | --- | --- | --- | --- | --- | --- | --- |

# Expression data

**Gene id on mapp: 11504**

| Sample name 11504| SystemCode L| LogFC 0.0| Pvalue 0.426979618| Type trans-PPS2 | | | --- | --- | | | | --- | --- | --- | --- | | | | --- | --- | --- | --- | --- | --- | | | | --- | --- | --- | --- | --- | --- | --- | --- | | |
| --- | --- | --- | --- | --- | --- | --- | --- | --- | --- |

  
  

---

  
  

# Cross references

  

|
|  |
| **UniGene** |
| Mm.1421 |
| Mm.413379 |
|
| **Agilent** |
| A\_52\_P213932 |
| A\_52\_P489295 |
|
| **Ensembl** |
| ENSMUSG00000022893 |
|
| **Illumina** |
| ILMN\_1230129 |
| ILMN\_1238495 |
| ILMN\_2664419 |
| ILMN\_2761082 |
|
| **Entrez Gene** |
| 11504 |
|
| **MGI** |
| MGI:109249 |
|
| **RefSeq** |
| NM\_009621 |
| NP\_033751 |
|
| **Uniprot/TrEMBL** |
| E9PY08 |
| P97857 |
| Q3TQF7 |
| Q3TTE6 |
|
| **GeneOntology** |
| GO:0001542 |
| GO:0001822 |
| GO:0004222 |
| GO:0005515 |
| GO:0005604 |
| GO:0006508 |
| GO:0008201 |
| GO:0008270 |
| GO:0016525 |
| GO:0031012 |
| GO:0031410 |
| GO:0060347 |
|
| **UCSC Genome Browser** |
| uc012aho.1 |
|
| **WikiGenes** |
| 11504 |
|
| **Affy** |
| 10440522 |
| 1450716\_at |
| 160606\_r\_at |
| D67076\_s\_at |
