# Supplementary material for: Automatically visualise and analyse data on pathways using PathVisioRPC from any programming environment
Source: BMC Bioinformatics. 2015 Aug 23;16(1):267. doi: 10.1186/s12859-015-0708-8 (PMC4546821; doi:10.1186/s12859-015-0708-8)
Supplement: Additional file 3: — Examples in Python. This zip archive contains the data and python script for the three python examples. (ZIP 15714 kb) [file 12859_2015_708_MOESM3_ESM.zip › Python_Examples/result_Example_1/geneList2/backpage/L_11512.html]

 

# geneproduct annotation

  

| Name: Adcy6| Identifier: 11512| Database: Entrez Gene| Synonyms: mKIAA0422 | | | --- | --- | | | | --- | --- | --- | --- | | | | --- | --- | --- | --- | --- | --- | | |
| --- | --- | --- | --- | --- | --- | --- | --- |

# Expression data

**Gene id on mapp: 11512**

| Sample name 11512| SystemCode L| LogFC 1.54504084| Pvalue 7.4017E-4| Type trans-PPS2 | | | --- | --- | | | | --- | --- | --- | --- | | | | --- | --- | --- | --- | --- | --- | | | | --- | --- | --- | --- | --- | --- | --- | --- | | |
| --- | --- | --- | --- | --- | --- | --- | --- | --- | --- |

  
  

---

  
  

# Cross references

  

|
|  |
| **UniGene** |
| Mm.157091 |
|
| **Ensembl** |
| ENSMUSG00000022994 |
|
| **Illumina** |
| ILMN\_1249888 |
| ILMN\_2659879 |
| ILMN\_2706140 |
|
| **Entrez Gene** |
| 11512 |
|
| **MGI** |
| MGI:87917 |
|
| **RefSeq** |
| NM\_007405 |
| NP\_031431 |
|
| **Uniprot/TrEMBL** |
| F8VQ52 |
|
| **GeneOntology** |
| GO:0000166 |
| GO:0004016 |
| GO:0005515 |
| GO:0005886 |
| GO:0006171 |
| GO:0007190 |
| GO:0007212 |
| GO:0016021 |
| GO:0035556 |
| GO:0071380 |
| GO:0071870 |
|
| **UCSC Genome Browser** |
| uc007xna.2 |
|
| **WikiGenes** |
| 11512 |
|
| **Affy** |
| 102321\_at |
| 10432190 |
| 1418128\_at |
| m93422\_s\_at |
