# Supplementary material for: Automatically visualise and analyse data on pathways using PathVisioRPC from any programming environment
Source: BMC Bioinformatics. 2015 Aug 23;16(1):267. doi: 10.1186/s12859-015-0708-8 (PMC4546821; doi:10.1186/s12859-015-0708-8)
Supplement: Additional file 3: — Examples in Python. This zip archive contains the data and python script for the three python examples. (ZIP 15714 kb) [file 12859_2015_708_MOESM3_ESM.zip › Python_Examples/result_Example_1/geneList3/backpage/L_11515.html]

 

# geneproduct annotation

  

| Name: Adcy9| Identifier: 11515| Database: Entrez Gene| Synonyms: D16Wsu65e | | | --- | --- | | | | --- | --- | --- | --- | | | | --- | --- | --- | --- | --- | --- | | |
| --- | --- | --- | --- | --- | --- | --- | --- |

# Expression data

**Gene id on mapp: 11515**

| Sample name 11515| SystemCode L| LogFC 0.0| Pvalue 0.338726098| Type trans-PPS2 | | | --- | --- | | | | --- | --- | --- | --- | | | | --- | --- | --- | --- | --- | --- | | | | --- | --- | --- | --- | --- | --- | --- | --- | | |
| --- | --- | --- | --- | --- | --- | --- | --- | --- | --- |

  
  

---

  
  

# Cross references

  

|
|  |
| **UniGene** |
| Mm.431828 |
| Mm.439750 |
|
| **Agilent** |
| A\_51\_P501538 |
| A\_55\_P2106666 |
|
| **Ensembl** |
| ENSMUSG00000005580 |
|
| **Illumina** |
| ILMN\_2723474 |
|
| **Entrez Gene** |
| 11515 |
|
| **MGI** |
| MGI:108450 |
|
| **RefSeq** |
| NM\_009624 |
| NP\_033754 |
|
| **Uniprot/TrEMBL** |
| E9Q706 |
| P51830 |
|
| **GeneOntology** |
| GO:0000166 |
| GO:0004016 |
| GO:0005524 |
| GO:0005886 |
| GO:0006171 |
| GO:0007190 |
| GO:0016021 |
| GO:0035556 |
| GO:0046872 |
|
| **UCSC Genome Browser** |
| uc007xzq.1 |
| uc007xzr.1 |
| uc007xzs.1 |
|
| **WikiGenes** |
| 11515 |
|
| **Affy** |
| 10437364 |
| 1418586\_at |
| 92527\_at |
| u30602\_s\_at |
