# Supplementary material for: Automatically visualise and analyse data on pathways using PathVisioRPC from any programming environment
Source: BMC Bioinformatics. 2015 Aug 23;16(1):267. doi: 10.1186/s12859-015-0708-8 (PMC4546821; doi:10.1186/s12859-015-0708-8)
Supplement: Additional file 3: — Examples in Python. This zip archive contains the data and python script for the three python examples. (ZIP 15714 kb) [file 12859_2015_708_MOESM3_ESM.zip › Python_Examples/result_Example_1/geneList3/backpage/L_11517.html]

 

# geneproduct annotation

  

| Name: Adcyap1r1| Identifier: 11517| Database: Entrez Gene| Synonyms: 2900024I10Rik | | | --- | --- | | | | --- | --- | --- | --- | | | | --- | --- | --- | --- | --- | --- | | |
| --- | --- | --- | --- | --- | --- | --- | --- |

# Expression data

**Gene id on mapp: 11517**

| Sample name 11517| SystemCode L| LogFC 0.0| Pvalue 0.409849168| Type trans-PPS2 | | | --- | --- | | | | --- | --- | --- | --- | | | | --- | --- | --- | --- | --- | --- | | | | --- | --- | --- | --- | --- | --- | --- | --- | | |
| --- | --- | --- | --- | --- | --- | --- | --- | --- | --- |

  
  

---

  
  

# Cross references

  

|
|  |
| **UniGene** |
| Mm.397683 |
| Mm.403675 |
|
| **Agilent** |
| A\_51\_P259603 |
| A\_52\_P615225 |
|
| **Ensembl** |
| ENSMUSG00000029778 |
|
| **Illumina** |
| ILMN\_1243799 |
| ILMN\_2456845 |
| ILMN\_2717037 |
| ILMN\_2717038 |
| ILMN\_2758957 |
|
| **Entrez Gene** |
| 11517 |
|
| **MGI** |
| MGI:108449 |
|
| **RefSeq** |
| NM\_001025372 |
| NM\_007407 |
| NP\_001020543 |
| NP\_031433 |
|
| **Uniprot/TrEMBL** |
| E9PVE8 |
| E9Q3E8 |
| E9Q4B3 |
| E9Q968 |
| E9QAL0 |
| P70205 |
| Q6NXJ9 |
| Q8BGA4 |
|
| **GeneOntology** |
| GO:0004930 |
| GO:0004999 |
| GO:0005515 |
| GO:0005737 |
| GO:0005768 |
| GO:0005791 |
| GO:0005886 |
| GO:0005901 |
| GO:0005923 |
| GO:0007186 |
| GO:0007202 |
| GO:0007283 |
| GO:0008179 |
| GO:0009986 |
| GO:0010524 |
| GO:0016020 |
| GO:0016021 |
| GO:0019933 |
| GO:0030154 |
| GO:0030306 |
| GO:0030819 |
| GO:0032355 |
| GO:0033555 |
| GO:0042493 |
| GO:0042923 |
| GO:0043005 |
| GO:0045471 |
| GO:0046545 |
| GO:0060548 |
| GO:0060732 |
|
| **UCSC Genome Browser** |
| uc009cat.1 |
| uc009cau.1 |
|
| **WikiGenes** |
| 11517 |
|
| **Affy** |
| 10538482 |
| 106895\_at |
| 117286\_at |
| 129856\_at |
| 1429022\_at |
| 1439042\_at |
| 1450799\_at |
| 98361\_at |
| 98362\_s\_at |
| Msa.3144.0\_at |
