# Supplementary material for: Automatically visualise and analyse data on pathways using PathVisioRPC from any programming environment
Source: BMC Bioinformatics. 2015 Aug 23;16(1):267. doi: 10.1186/s12859-015-0708-8 (PMC4546821; doi:10.1186/s12859-015-0708-8)
Supplement: Additional file 3: — Examples in Python. This zip archive contains the data and python script for the three python examples. (ZIP 15714 kb) [file 12859_2015_708_MOESM3_ESM.zip › Python_Examples/result_Example_1/geneList3/backpage/L_11518.html]

 

# geneproduct annotation

  

| Name: Add1| Identifier: 11518| Database: Entrez Gene| Synonyms: AI256389 | | | --- | --- | | | | --- | --- | --- | --- | | | | --- | --- | --- | --- | --- | --- | | |
| --- | --- | --- | --- | --- | --- | --- | --- |

# Expression data

**Gene id on mapp: 11518**

| Sample name 11518| SystemCode L| LogFC 1.199787328| Pvalue 0.036854134| Type trans-PPS2 | | | --- | --- | | | | --- | --- | --- | --- | | | | --- | --- | --- | --- | --- | --- | | | | --- | --- | --- | --- | --- | --- | --- | --- | | |
| --- | --- | --- | --- | --- | --- | --- | --- | --- | --- |

  
  

---

  
  

# Cross references

  

|
|  |
| **UniGene** |
| Mm.289106 |
| Mm.471179 |
|
| **Agilent** |
| A\_51\_P282404 |
| A\_55\_P1990331 |
| A\_55\_P1990336 |
|
| **Ensembl** |
| ENSMUSG00000029106 |
|
| **Illumina** |
| ILMN\_1215275 |
| ILMN\_1225946 |
| ILMN\_2659062 |
| ILMN\_2659063 |
| ILMN\_2714257 |
| ILMN\_3033533 |
| ILMN\_3107228 |
|
| **Entrez Gene** |
| 11518 |
|
| **MGI** |
| MGI:87918 |
|
| **RefSeq** |
| NM\_001024458 |
| NM\_001102444 |
| NM\_013457 |
| NP\_001019629 |
| NP\_001095914 |
| NP\_038485 |
|
| **Uniprot/TrEMBL** |
| D3Z0T1 |
| E9Q1K3 |
| F6V4G5 |
| F8WGR0 |
| F8WHZ9 |
| Q9QYC0 |
|
| **GeneOntology** |
| GO:0000902 |
| GO:0001701 |
| GO:0005198 |
| GO:0005515 |
| GO:0005516 |
| GO:0005634 |
| GO:0005856 |
| GO:0005886 |
| GO:0006884 |
| GO:0008134 |
| GO:0008290 |
| GO:0014069 |
| GO:0016020 |
| GO:0020027 |
| GO:0030218 |
| GO:0030425 |
| GO:0030507 |
| GO:0032092 |
| GO:0035264 |
| GO:0042608 |
| GO:0042803 |
| GO:0043197 |
| GO:0045202 |
| GO:0045766 |
| GO:0045807 |
| GO:0046872 |
| GO:0046982 |
| GO:0048471 |
| GO:0048873 |
| GO:0051015 |
| GO:0051016 |
| GO:0051017 |
| GO:0071300 |
|
| **UCSC Genome Browser** |
| uc008xcp.1 |
| uc008xcq.1 |
| uc008xcr.1 |
|
| **WikiGenes** |
| 11518 |
|
| **Affy** |
| 10521222 |
| 141087\_f\_at |
| 141096\_f\_at |
| 1420953\_at |
| 1420954\_a\_at |
| 1450054\_at |
| 94535\_at |
| aa638800\_s\_at |
