# Supplementary material for: Automatically visualise and analyse data on pathways using PathVisioRPC from any programming environment
Source: BMC Bioinformatics. 2015 Aug 23;16(1):267. doi: 10.1186/s12859-015-0708-8 (PMC4546821; doi:10.1186/s12859-015-0708-8)
Supplement: Additional file 3: — Examples in Python. This zip archive contains the data and python script for the three python examples. (ZIP 15714 kb) [file 12859_2015_708_MOESM3_ESM.zip › Python_Examples/result_Example_1/geneList3/backpage/L_11519.html]

 

# geneproduct annotation

  

| Name: Add2| Identifier: 11519| Database: Entrez Gene| Synonyms: add97 | | | --- | --- | | | | --- | --- | --- | --- | | | | --- | --- | --- | --- | --- | --- | | |
| --- | --- | --- | --- | --- | --- | --- | --- |

# Expression data

**Gene id on mapp: 11519**

| Sample name 11519| SystemCode L| LogFC 0.0| Pvalue 0.085910324| Type trans-PPS2 | | | --- | --- | | | | --- | --- | --- | --- | | | | --- | --- | --- | --- | --- | --- | | | | --- | --- | --- | --- | --- | --- | --- | --- | | |
| --- | --- | --- | --- | --- | --- | --- | --- | --- | --- |

  
  

---

  
  

# Cross references

  

|
|  |
| **UniGene** |
| Mm.104155 |
|
| **Agilent** |
| A\_51\_P452854 |
| A\_52\_P623053 |
| A\_55\_P2063376 |
|
| **Ensembl** |
| ENSMUSG00000030000 |
|
| **Illumina** |
| ILMN\_1254225 |
|
| **Entrez Gene** |
| 11519 |
|
| **MGI** |
| MGI:87919 |
|
| **RefSeq** |
| NM\_001271857 |
| NM\_001271858 |
| NM\_001271859 |
| NM\_013458 |
| NP\_001258786 |
| NP\_001258787 |
| NP\_001258788 |
| NP\_001258789 |
| NP\_001258790 |
| NP\_038486 |
|
| **Uniprot/TrEMBL** |
| Q9QYB8 |
|
| **GeneOntology** |
| GO:0005198 |
| GO:0005516 |
| GO:0005737 |
| GO:0005886 |
| GO:0008290 |
| GO:0016020 |
| GO:0030097 |
| GO:0030507 |
| GO:0032092 |
| GO:0042803 |
| GO:0046872 |
| GO:0046982 |
| GO:0051015 |
| GO:0051016 |
| GO:0051017 |
|
| **UCSC Genome Browser** |
| uc009crh.1 |
|
| **WikiGenes** |
| 11519 |
|
| **Affy** |
| 10539669 |
| 106910\_at |
| 1421975\_a\_at |
| 1450442\_at |
| 1451914\_a\_at |
