# Supplementary material for: Automatically visualise and analyse data on pathways using PathVisioRPC from any programming environment
Source: BMC Bioinformatics. 2015 Aug 23;16(1):267. doi: 10.1186/s12859-015-0708-8 (PMC4546821; doi:10.1186/s12859-015-0708-8)
Supplement: Additional file 3: — Examples in Python. This zip archive contains the data and python script for the three python examples. (ZIP 15714 kb) [file 12859_2015_708_MOESM3_ESM.zip › Python_Examples/result_Example_1/geneList3/backpage/L_11520.html]

 

# geneproduct annotation

  

| Name: Plin2| Identifier: 11520| Database: Entrez Gene| Synonyms: AA407157 | | | --- | --- | | | | --- | --- | --- | --- | | | | --- | --- | --- | --- | --- | --- | | |
| --- | --- | --- | --- | --- | --- | --- | --- |

# Expression data

**Gene id on mapp: 11520**

| Sample name 11520| SystemCode L| LogFC 0.0| Pvalue 0.083051235| Type trans-PPS2 | | | --- | --- | | | | --- | --- | --- | --- | | | | --- | --- | --- | --- | --- | --- | | | | --- | --- | --- | --- | --- | --- | --- | --- | | |
| --- | --- | --- | --- | --- | --- | --- | --- | --- | --- |

  
  

---

  
  

# Cross references

  

|
|  |
| **UniGene** |
| Mm.381 |
|
| **Agilent** |
| A\_51\_P258150 |
| A\_55\_P1999648 |
|
| **Ensembl** |
| ENSMUSG00000028494 |
|
| **Illumina** |
| ILMN\_1222246 |
|
| **Entrez Gene** |
| 11520 |
|
| **MGI** |
| MGI:87920 |
|
| **RefSeq** |
| NM\_007408 |
| NP\_031434 |
|
| **Uniprot/TrEMBL** |
| B1AXR3 |
| B1AXR5 |
| P43883 |
| Q8C5B5 |
|
| **GeneOntology** |
| GO:0005515 |
| GO:0005634 |
| GO:0005737 |
| GO:0005811 |
| GO:0005886 |
| GO:0014070 |
| GO:0015909 |
| GO:0016020 |
| GO:0019915 |
| GO:0042493 |
|
| **UCSC Genome Browser** |
| uc008tlz.1 |
|
| **WikiGenes** |
| 11520 |
|
| **Affy** |
| 10514221 |
| 135341\_r\_at |
| 1448318\_at |
| 98589\_at |
| M93275\_s\_at |
