# Supplementary material for: Automatically visualise and analyse data on pathways using PathVisioRPC from any programming environment
Source: BMC Bioinformatics. 2015 Aug 23;16(1):267. doi: 10.1186/s12859-015-0708-8 (PMC4546821; doi:10.1186/s12859-015-0708-8)
Supplement: Additional file 3: — Examples in Python. This zip archive contains the data and python script for the three python examples. (ZIP 15714 kb) [file 12859_2015_708_MOESM3_ESM.zip › Python_Examples/result_Example_1/geneList3/backpage/L_11522.html]

 

# geneproduct annotation

  

| Name: Adh1| Identifier: 11522| Database: Entrez Gene| Synonyms: Adh-3e | | | --- | --- | | | | --- | --- | --- | --- | | | | --- | --- | --- | --- | --- | --- | | |
| --- | --- | --- | --- | --- | --- | --- | --- |

# Expression data

**Gene id on mapp: 11522**

| Sample name 11522| SystemCode L| LogFC 0.0| Pvalue 0.190542301| Type trans-PPS2 | | | --- | --- | | | | --- | --- | --- | --- | | | | --- | --- | --- | --- | --- | --- | | | | --- | --- | --- | --- | --- | --- | --- | --- | | |
| --- | --- | --- | --- | --- | --- | --- | --- | --- | --- |

  
  

---

  
  

# Cross references

  

|
|  |
| **UniGene** |
| Mm.2409 |
|
| **Agilent** |
| A\_51\_P428555 |
| A\_52\_P629895 |
|
| **Ensembl** |
| ENSMUSG00000074207 |
|
| **Illumina** |
| ILMN\_1258501 |
| ILMN\_2850077 |
|
| **Entrez Gene** |
| 11522 |
|
| **MGI** |
| MGI:87921 |
|
| **RefSeq** |
| NM\_007409 |
| NP\_031435 |
|
| **Uniprot/TrEMBL** |
| E0CXV3 |
| P00329 |
| Q3UKA4 |
|
| **GeneOntology** |
| GO:0000166 |
| GO:0001523 |
| GO:0004022 |
| GO:0005622 |
| GO:0005739 |
| GO:0006068 |
| GO:0008270 |
| GO:0032526 |
| GO:0033574 |
| GO:0042572 |
| GO:0042573 |
| GO:0042803 |
| GO:0048149 |
| GO:0048545 |
|
| **UCSC Genome Browser** |
| uc008rnf.1 |
|
| **WikiGenes** |
| 11522 |
|
| **Affy** |
| 10496438 |
| 1416225\_at |
| 94906\_at |
| m18480\_s\_at |
