# Supplementary material for: Automatically visualise and analyse data on pathways using PathVisioRPC from any programming environment
Source: BMC Bioinformatics. 2015 Aug 23;16(1):267. doi: 10.1186/s12859-015-0708-8 (PMC4546821; doi:10.1186/s12859-015-0708-8)
Supplement: Additional file 3: — Examples in Python. This zip archive contains the data and python script for the three python examples. (ZIP 15714 kb) [file 12859_2015_708_MOESM3_ESM.zip › Python_Examples/result_Example_1/geneList3/backpage/L_11532.html]

 

# geneproduct annotation

  

| Name: Adh5| Identifier: 11532| Database: Entrez Gene| Synonyms: GSNOR | | | --- | --- | | | | --- | --- | --- | --- | | | | --- | --- | --- | --- | --- | --- | | |
| --- | --- | --- | --- | --- | --- | --- | --- |

# Expression data

**Gene id on mapp: 11532**

| Sample name 11532| SystemCode L| LogFC 0.0| Pvalue 0.303541927| Type trans-PPS2 | | | --- | --- | | | | --- | --- | --- | --- | | | | --- | --- | --- | --- | --- | --- | | | | --- | --- | --- | --- | --- | --- | --- | --- | | |
| --- | --- | --- | --- | --- | --- | --- | --- | --- | --- |

  
  

---

  
  

# Cross references

  

|
|  |
| **UniGene** |
| Mm.3874 |
| Mm.473058 |
|
| **Agilent** |
| A\_51\_P404275 |
| A\_55\_P2018482 |
|
| **Ensembl** |
| ENSMUSG00000028138 |
|
| **Illumina** |
| ILMN\_1212856 |
| ILMN\_1217813 |
| ILMN\_1242178 |
| ILMN\_2852979 |
|
| **Entrez Gene** |
| 11532 |
|
| **MGI** |
| MGI:87929 |
|
| **PDB** |
| 1OTQ |
|
| **RefSeq** |
| NM\_007410 |
| NP\_031436 |
|
| **Uniprot/TrEMBL** |
| P28474 |
| Q6P5I3 |
|
| **GeneOntology** |
| GO:0000166 |
| GO:0001523 |
| GO:0003016 |
| GO:0004022 |
| GO:0005504 |
| GO:0005634 |
| GO:0005737 |
| GO:0005739 |
| GO:0006068 |
| GO:0006069 |
| GO:0007568 |
| GO:0008270 |
| GO:0018119 |
| GO:0018467 |
| GO:0032496 |
| GO:0042803 |
| GO:0045777 |
| GO:0046294 |
| GO:0051409 |
| GO:0051775 |
| GO:0051903 |
|
| **UCSC Genome Browser** |
| uc008rnk.1 |
|
| **WikiGenes** |
| 11532 |
|
| **Affy** |
| 10496475 |
| 1416185\_a\_at |
| 1443380\_at |
| 98625\_s\_at |
| Msa.1556.0\_f\_at |
| m84147\_f\_at |
