# Supplementary material for: Automatically visualise and analyse data on pathways using PathVisioRPC from any programming environment
Source: BMC Bioinformatics. 2015 Aug 23;16(1):267. doi: 10.1186/s12859-015-0708-8 (PMC4546821; doi:10.1186/s12859-015-0708-8)
Supplement: Additional file 3: — Examples in Python. This zip archive contains the data and python script for the three python examples. (ZIP 15714 kb) [file 12859_2015_708_MOESM3_ESM.zip › Python_Examples/result_Example_1/geneList3/backpage/L_11535.html]

 

# geneproduct annotation

  

| Name: Adm| Identifier: 11535| Database: Entrez Gene| Synonyms: AM | | | --- | --- | | | | --- | --- | --- | --- | | | | --- | --- | --- | --- | --- | --- | | |
| --- | --- | --- | --- | --- | --- | --- | --- |

# Expression data

**Gene id on mapp: 11535**

| Sample name 11535| SystemCode L| LogFC 0.0| Pvalue 0.429504194| Type trans-PPS2 | | | --- | --- | | | | --- | --- | --- | --- | | | | --- | --- | --- | --- | --- | --- | | | | --- | --- | --- | --- | --- | --- | --- | --- | | |
| --- | --- | --- | --- | --- | --- | --- | --- | --- | --- |

  
  

---

  
  

# Cross references

  

|
|  |
| **UniGene** |
| Mm.1408 |
| Mm.470780 |
|
| **Agilent** |
| A\_51\_P265571 |
|
| **Ensembl** |
| ENSMUSG00000030790 |
|
| **Illumina** |
| ILMN\_1247207 |
|
| **Entrez Gene** |
| 11535 |
|
| **MGI** |
| MGI:108058 |
|
| **RefSeq** |
| NM\_009627 |
| NP\_033757 |
|
| **Uniprot/TrEMBL** |
| P97297 |
|
| **GeneOntology** |
| GO:0001570 |
| GO:0001666 |
| GO:0001843 |
| GO:0002026 |
| GO:0002031 |
| GO:0005179 |
| GO:0005515 |
| GO:0005615 |
| GO:0005737 |
| GO:0006171 |
| GO:0006950 |
| GO:0007204 |
| GO:0007507 |
| GO:0007565 |
| GO:0007568 |
| GO:0008209 |
| GO:0008284 |
| GO:0008285 |
| GO:0009409 |
| GO:0009611 |
| GO:0010033 |
| GO:0010460 |
| GO:0019933 |
| GO:0030819 |
| GO:0031100 |
| GO:0031102 |
| GO:0031623 |
| GO:0031700 |
| GO:0032496 |
| GO:0032868 |
| GO:0042475 |
| GO:0042594 |
| GO:0043116 |
| GO:0045766 |
| GO:0045906 |
| GO:0045909 |
| GO:0046879 |
| GO:0048589 |
| GO:0051384 |
| GO:0055074 |
| GO:0060670 |
| GO:0060712 |
| GO:0097084 |
| GO:2001214 |
|
| **UCSC Genome Browser** |
| uc009jfj.1 |
|
| **WikiGenes** |
| 11535 |
|
| **Affy** |
| 102798\_at |
| 10556297 |
| 1416077\_at |
| u77630\_s\_at |
