# Supplementary material for: Automatically visualise and analyse data on pathways using PathVisioRPC from any programming environment
Source: BMC Bioinformatics. 2015 Aug 23;16(1):267. doi: 10.1186/s12859-015-0708-8 (PMC4546821; doi:10.1186/s12859-015-0708-8)
Supplement: Additional file 3: — Examples in Python. This zip archive contains the data and python script for the three python examples. (ZIP 15714 kb) [file 12859_2015_708_MOESM3_ESM.zip › Python_Examples/result_Example_1/geneList3/backpage/L_11537.html]

 

# geneproduct annotation

  

| Name: Cfd| Identifier: 11537| Database: Entrez Gene| Synonyms: Adn | | | --- | --- | | | | --- | --- | --- | --- | | | | --- | --- | --- | --- | --- | --- | | |
| --- | --- | --- | --- | --- | --- | --- | --- |

# Expression data

**Gene id on mapp: 11537**

| Sample name 11537| SystemCode L| LogFC 7.246696398| Pvalue 8.2E-5| Type trans-PPS2 | | | --- | --- | | | | --- | --- | --- | --- | | | | --- | --- | --- | --- | --- | --- | | | | --- | --- | --- | --- | --- | --- | --- | --- | | |
| --- | --- | --- | --- | --- | --- | --- | --- | --- | --- |

  
  

---

  
  

# Cross references

  

|
|  |
| **Agilent** |
| A\_51\_P156955 |
|
| **Ensembl** |
| ENSMUSG00000061780 |
|
| **Illumina** |
| ILMN\_1230957 |
| ILMN\_2835423 |
|
| **Entrez Gene** |
| 11537 |
|
| **MGI** |
| MGI:87931 |
|
| **RefSeq** |
| NM\_013459 |
| NP\_038487 |
|
| **Uniprot/TrEMBL** |
| B7ZNS9 |
| P03953 |
| Q3UP47 |
|
| **GeneOntology** |
| GO:0004252 |
| GO:0005615 |
| GO:0006508 |
| GO:0006957 |
|
| **UCSC Genome Browser** |
| uc007gaj.2 |
|
| **WikiGenes** |
| 11537 |
|
| **Affy** |
| 10364542 |
| 1417867\_at |
| 99671\_at |
| Msa.450.0\_f\_at |
| x04673\_s\_at |
