# Supplementary material for: Automatically visualise and analyse data on pathways using PathVisioRPC from any programming environment
Source: BMC Bioinformatics. 2015 Aug 23;16(1):267. doi: 10.1186/s12859-015-0708-8 (PMC4546821; doi:10.1186/s12859-015-0708-8)
Supplement: Additional file 3: — Examples in Python. This zip archive contains the data and python script for the three python examples. (ZIP 15714 kb) [file 12859_2015_708_MOESM3_ESM.zip › Python_Examples/result_Example_1/geneList3/backpage/L_11539.html]

 

# geneproduct annotation

  

| Name: Adora1| Identifier: 11539| Database: Entrez Gene| Synonyms: BB176431 | | | --- | --- | | | | --- | --- | --- | --- | | | | --- | --- | --- | --- | --- | --- | | |
| --- | --- | --- | --- | --- | --- | --- | --- |

# Expression data

**Gene id on mapp: 11539**

| Sample name 11539| SystemCode L| LogFC 2.13899955| Pvalue 1.84E-6| Type trans-PPS2 | | | --- | --- | | | | --- | --- | --- | --- | | | | --- | --- | --- | --- | --- | --- | | | | --- | --- | --- | --- | --- | --- | --- | --- | | |
| --- | --- | --- | --- | --- | --- | --- | --- | --- | --- |

  
  

---

  
  

# Cross references

  

|
|  |
| **UniGene** |
| Mm.298908 |
| Mm.444006 |
|
| **Agilent** |
| A\_51\_P188845 |
| A\_52\_P172272 |
| A\_52\_P661412 |
| A\_55\_P2101776 |
|
| **Ensembl** |
| ENSMUSG00000042429 |
|
| **Illumina** |
| ILMN\_1254016 |
| ILMN\_3065420 |
| ILMN\_3143358 |
|
| **Entrez Gene** |
| 11539 |
|
| **MGI** |
| MGI:99401 |
|
| **RefSeq** |
| NM\_001008533 |
| NM\_001039510 |
| NP\_001008533 |
| NP\_001034599 |
|
| **Uniprot/TrEMBL** |
| Q3URG8 |
| Q60612 |
| Q8R0M5 |
|
| **GeneOntology** |
| GO:0000186 |
| GO:0001609 |
| GO:0001659 |
| GO:0001664 |
| GO:0001666 |
| GO:0001883 |
| GO:0002087 |
| GO:0002674 |
| GO:0002686 |
| GO:0002793 |
| GO:0003093 |
| GO:0004629 |
| GO:0005515 |
| GO:0005783 |
| GO:0005886 |
| GO:0006612 |
| GO:0007186 |
| GO:0007193 |
| GO:0008285 |
| GO:0012505 |
| GO:0014050 |
| GO:0014069 |
| GO:0016021 |
| GO:0016042 |
| GO:0016323 |
| GO:0030425 |
| GO:0030673 |
| GO:0031683 |
| GO:0032229 |
| GO:0032244 |
| GO:0032279 |
| GO:0032795 |
| GO:0032900 |
| GO:0035307 |
| GO:0035814 |
| GO:0042323 |
| GO:0042734 |
| GO:0043025 |
| GO:0043066 |
| GO:0043195 |
| GO:0043268 |
| GO:0044297 |
| GO:0045211 |
| GO:0045741 |
| GO:0045776 |
| GO:0045777 |
| GO:0045822 |
| GO:0045908 |
| GO:0046888 |
| GO:0046982 |
| GO:0048167 |
| GO:0048786 |
| GO:0050728 |
| GO:0050890 |
| GO:0050965 |
| GO:0050995 |
| GO:0051967 |
| GO:0055117 |
| GO:0055118 |
| GO:0060079 |
| GO:0060087 |
| GO:0070256 |
|
| **UCSC Genome Browser** |
| uc007crj.1 |
| uc007crk.1 |
|
| **WikiGenes** |
| 11539 |
|
| **Affy** |
| 10357878 |
| 1427331\_at |
| 1435495\_at |
| 163668\_at |
| 92374\_at |
