# Supplementary material for: Automatically visualise and analyse data on pathways using PathVisioRPC from any programming environment
Source: BMC Bioinformatics. 2015 Aug 23;16(1):267. doi: 10.1186/s12859-015-0708-8 (PMC4546821; doi:10.1186/s12859-015-0708-8)
Supplement: Additional file 3: — Examples in Python. This zip archive contains the data and python script for the three python examples. (ZIP 15714 kb) [file 12859_2015_708_MOESM3_ESM.zip › Python_Examples/result_Example_1/geneList3/backpage/L_11540.html]

 

# geneproduct annotation

  

| Name: Adora2a| Identifier: 11540| Database: Entrez Gene| Synonyms: A2aR | | | --- | --- | | | | --- | --- | --- | --- | | | | --- | --- | --- | --- | --- | --- | | |
| --- | --- | --- | --- | --- | --- | --- | --- |

# Expression data

**Gene id on mapp: 11540**

| Sample name 11540| SystemCode L| LogFC 0.0| Pvalue 0.573185202| Type trans-PPS2 | | | --- | --- | | | | --- | --- | --- | --- | | | | --- | --- | --- | --- | --- | --- | | | | --- | --- | --- | --- | --- | --- | --- | --- | | |
| --- | --- | --- | --- | --- | --- | --- | --- | --- | --- |

  
  

---

  
  

# Cross references

  

|
|  |
| **UniGene** |
| Mm.333734 |
|
| **Agilent** |
| A\_52\_P240878 |
| A\_55\_P2109382 |
|
| **Ensembl** |
| ENSMUSG00000020178 |
|
| **Illumina** |
| ILMN\_2623091 |
|
| **Entrez Gene** |
| 11540 |
|
| **MGI** |
| MGI:99402 |
|
| **RefSeq** |
| NM\_009630 |
| NP\_033760 |
|
| **Uniprot/TrEMBL** |
| Q60613 |
|
| **GeneOntology** |
| GO:0001609 |
| GO:0001963 |
| GO:0001973 |
| GO:0001975 |
| GO:0005515 |
| GO:0005886 |
| GO:0006355 |
| GO:0006469 |
| GO:0007186 |
| GO:0007189 |
| GO:0007205 |
| GO:0007271 |
| GO:0007626 |
| GO:0008285 |
| GO:0012505 |
| GO:0014049 |
| GO:0014057 |
| GO:0014061 |
| GO:0014069 |
| GO:0016020 |
| GO:0016021 |
| GO:0019899 |
| GO:0030424 |
| GO:0030425 |
| GO:0030673 |
| GO:0030819 |
| GO:0031000 |
| GO:0031802 |
| GO:0032230 |
| GO:0032279 |
| GO:0035249 |
| GO:0035810 |
| GO:0035815 |
| GO:0040013 |
| GO:0042311 |
| GO:0042493 |
| GO:0042734 |
| GO:0042755 |
| GO:0043025 |
| GO:0043116 |
| GO:0043154 |
| GO:0043279 |
| GO:0043524 |
| GO:0044446 |
| GO:0045211 |
| GO:0046636 |
| GO:0046982 |
| GO:0048143 |
| GO:0048167 |
| GO:0048786 |
| GO:0048812 |
| GO:0050714 |
| GO:0050728 |
| GO:0051393 |
| GO:0051881 |
| GO:0051899 |
| GO:0051924 |
| GO:0051968 |
| GO:0060079 |
| GO:0060080 |
| GO:0060134 |
| GO:2001235 |
|
| **UCSC Genome Browser** |
| uc007fqh.1 |
|
| **WikiGenes** |
| 11540 |
|
| **Affy** |
| 101363\_at |
| 101364\_at |
| 10364030 |
| 1427519\_at |
| 1460710\_at |
