# Supplementary material for: Automatically visualise and analyse data on pathways using PathVisioRPC from any programming environment
Source: BMC Bioinformatics. 2015 Aug 23;16(1):267. doi: 10.1186/s12859-015-0708-8 (PMC4546821; doi:10.1186/s12859-015-0708-8)
Supplement: Additional file 3: — Examples in Python. This zip archive contains the data and python script for the three python examples. (ZIP 15714 kb) [file 12859_2015_708_MOESM3_ESM.zip › Python_Examples/result_Example_1/geneList3/backpage/L_11544.html]

 

# geneproduct annotation

  

| Name: Adprh| Identifier: 11544| Database: Entrez Gene| Synonyms: Arh1 | | | --- | --- | | | | --- | --- | --- | --- | | | | --- | --- | --- | --- | --- | --- | | |
| --- | --- | --- | --- | --- | --- | --- | --- |

# Expression data

**Gene id on mapp: 11544**

| Sample name 11544| SystemCode L| LogFC 0.0| Pvalue 0.078115935| Type trans-PPS2 | | | --- | --- | | | | --- | --- | --- | --- | | | | --- | --- | --- | --- | --- | --- | | | | --- | --- | --- | --- | --- | --- | --- | --- | | |
| --- | --- | --- | --- | --- | --- | --- | --- | --- | --- |

  
  

---

  
  

# Cross references

  

|
|  |
| **UniGene** |
| Mm.414986 |
| Mm.473321 |
|
| **Agilent** |
| A\_51\_P338615 |
| A\_52\_P504478 |
|
| **Ensembl** |
| ENSMUSG00000002844 |
|
| **Illumina** |
| ILMN\_2595704 |
|
| **Entrez Gene** |
| 11544 |
|
| **MGI** |
| MGI:1098234 |
|
| **RefSeq** |
| NM\_007414 |
| NP\_031440 |
|
| **Uniprot/TrEMBL** |
| P54923 |
| Q3U5N4 |
| Q9CTF5 |
|
| **GeneOntology** |
| GO:0000287 |
| GO:0003875 |
| GO:0006464 |
| GO:0051725 |
|
| **UCSC Genome Browser** |
| uc007zex.1 |
|
| **WikiGenes** |
| 11544 |
|
| **Affy** |
| 10439455 |
| 1448333\_at |
| 93540\_at |
| Msa.449.0\_s\_at |
