# Supplementary material for: Automatically visualise and analyse data on pathways using PathVisioRPC from any programming environment
Source: BMC Bioinformatics. 2015 Aug 23;16(1):267. doi: 10.1186/s12859-015-0708-8 (PMC4546821; doi:10.1186/s12859-015-0708-8)
Supplement: Additional file 3: — Examples in Python. This zip archive contains the data and python script for the three python examples. (ZIP 15714 kb) [file 12859_2015_708_MOESM3_ESM.zip › Python_Examples/result_Example_1/geneList3/backpage/L_11545.html]

 

# geneproduct annotation

  

| Name: Parp1| Identifier: 11545| Database: Entrez Gene| Synonyms: PARP | | | --- | --- | | | | --- | --- | --- | --- | | | | --- | --- | --- | --- | --- | --- | | |
| --- | --- | --- | --- | --- | --- | --- | --- |

# Expression data

**Gene id on mapp: 11545**

| Sample name 11545| SystemCode L| LogFC 0.0| Pvalue 0.212248932| Type trans-PPS2 | | | --- | --- | | | | --- | --- | --- | --- | | | | --- | --- | --- | --- | --- | --- | | | | --- | --- | --- | --- | --- | --- | --- | --- | | |
| --- | --- | --- | --- | --- | --- | --- | --- | --- | --- |

  
  

---

  
  

# Cross references

  

|
|  |
| **UniGene** |
| Mm.277779 |
| Mm.470831 |
|
| **Agilent** |
| A\_51\_P314273 |
| A\_51\_P314277 |
|
| **Ensembl** |
| ENSMUSG00000026496 |
|
| **Illumina** |
| ILMN\_1222228 |
| ILMN\_2621385 |
| ILMN\_2971744 |
|
| **Entrez Gene** |
| 11545 |
|
| **MGI** |
| MGI:1340806 |
|
| **RefSeq** |
| NM\_007415 |
| NP\_031441 |
|
| **Uniprot/TrEMBL** |
| Q3UX06 |
| Q921K2 |
|
| **GeneOntology** |
| GO:0000723 |
| GO:0003677 |
| GO:0003950 |
| GO:0005515 |
| GO:0005634 |
| GO:0005635 |
| GO:0005654 |
| GO:0005667 |
| GO:0005730 |
| GO:0006259 |
| GO:0006281 |
| GO:0006284 |
| GO:0006302 |
| GO:0006471 |
| GO:0008134 |
| GO:0008270 |
| GO:0016540 |
| GO:0032869 |
| GO:0040009 |
| GO:0042769 |
| GO:0043234 |
| GO:0047485 |
| GO:0051287 |
| GO:0070212 |
|
| **UCSC Genome Browser** |
| uc007dwi.1 |
|
| **WikiGenes** |
| 11545 |
|
| **Affy** |
| 101957\_f\_at |
| 10352242 |
| 1422502\_at |
| 1422503\_s\_at |
| 1435368\_a\_at |
| Msa.2151.0\_s\_at |
| aa119245\_s\_at |
