# Supplementary material for: Automatically visualise and analyse data on pathways using PathVisioRPC from any programming environment
Source: BMC Bioinformatics. 2015 Aug 23;16(1):267. doi: 10.1186/s12859-015-0708-8 (PMC4546821; doi:10.1186/s12859-015-0708-8)
Supplement: Additional file 3: — Examples in Python. This zip archive contains the data and python script for the three python examples. (ZIP 15714 kb) [file 12859_2015_708_MOESM3_ESM.zip › Python_Examples/result_Example_1/geneList3/backpage/L_11546.html]

 

# geneproduct annotation

  

| Name: Parp2| Identifier: 11546| Database: Entrez Gene| Synonyms: ARTD2 | | | --- | --- | | | | --- | --- | --- | --- | | | | --- | --- | --- | --- | --- | --- | | |
| --- | --- | --- | --- | --- | --- | --- | --- |

# Expression data

**Gene id on mapp: 11546**

| Sample name 11546| SystemCode L| LogFC 0.0| Pvalue 0.550018821| Type trans-PPS2 | | | --- | --- | | | | --- | --- | --- | --- | | | | --- | --- | --- | --- | --- | --- | | | | --- | --- | --- | --- | --- | --- | --- | --- | | |
| --- | --- | --- | --- | --- | --- | --- | --- | --- | --- |

  
  

---

  
  

# Cross references

  

|
|  |
| **Agilent** |
| A\_51\_P471630 |
| A\_52\_P227445 |
|
| **Ensembl** |
| ENSMUSG00000036023 |
|
| **Illumina** |
| ILMN\_1226731 |
| ILMN\_2598576 |
|
| **Entrez Gene** |
| 11546 |
|
| **MGI** |
| MGI:1341112 |
|
| **PDB** |
| 1GS0 |
|
| **RefSeq** |
| NM\_009632 |
| NP\_033762 |
|
| **Uniprot/TrEMBL** |
| O88554 |
|
| **GeneOntology** |
| GO:0003677 |
| GO:0003950 |
| GO:0005515 |
| GO:0005634 |
| GO:0005654 |
| GO:0005730 |
| GO:0006281 |
| GO:0006284 |
| GO:0006471 |
|
| **UCSC Genome Browser** |
| uc007tlq.1 |
|
| **WikiGenes** |
| 11546 |
|
| **Affy** |
| 100903\_at |
| 10414497 |
| 1417800\_at |
| aa638884\_s\_at |
