# Supplementary material for: Automatically visualise and analyse data on pathways using PathVisioRPC from any programming environment
Source: BMC Bioinformatics. 2015 Aug 23;16(1):267. doi: 10.1186/s12859-015-0708-8 (PMC4546821; doi:10.1186/s12859-015-0708-8)
Supplement: Additional file 3: — Examples in Python. This zip archive contains the data and python script for the three python examples. (ZIP 15714 kb) [file 12859_2015_708_MOESM3_ESM.zip › Python_Examples/result_Example_1/geneList3/backpage/L_11548.html]

 

# geneproduct annotation

  

| Name: Adra1b| Identifier: 11548| Database: Entrez Gene| Synonyms: [a]1b | | | --- | --- | | | | --- | --- | --- | --- | | | | --- | --- | --- | --- | --- | --- | | |
| --- | --- | --- | --- | --- | --- | --- | --- |

# Expression data

**Gene id on mapp: 11548**

| Sample name 11548| SystemCode L| LogFC 0.0| Pvalue 0.381130957| Type trans-PPS2 | | | --- | --- | | | | --- | --- | --- | --- | | | | --- | --- | --- | --- | --- | --- | | | | --- | --- | --- | --- | --- | --- | --- | --- | | |
| --- | --- | --- | --- | --- | --- | --- | --- | --- | --- |

  
  

---

  
  

# Cross references

  

|
|  |
| **UniGene** |
| Mm.39086 |
|
| **Agilent** |
| A\_51\_P276766 |
| A\_52\_P639048 |
| A\_55\_P2100290 |
| A\_55\_P2177721 |
|
| **Ensembl** |
| ENSMUSG00000050541 |
|
| **Illumina** |
| ILMN\_2466647 |
| ILMN\_2606414 |
| ILMN\_2606415 |
|
| **Entrez Gene** |
| 11548 |
|
| **MGI** |
| MGI:104774 |
|
| **RefSeq** |
| NM\_007416 |
| NP\_031442 |
|
| **Uniprot/TrEMBL** |
| B1AU41 |
| Q8CGI5 |
| Q9DBL0 |
|
| **GeneOntology** |
| GO:0001974 |
| GO:0001975 |
| GO:0001987 |
| GO:0001996 |
| GO:0001997 |
| GO:0004937 |
| GO:0005634 |
| GO:0005886 |
| GO:0007512 |
| GO:0007626 |
| GO:0008542 |
| GO:0016021 |
| GO:0016049 |
| GO:0031965 |
| GO:0035024 |
| GO:0035265 |
| GO:0042593 |
| GO:0043278 |
| GO:0043410 |
| GO:0045818 |
| GO:0045819 |
| GO:0046982 |
| GO:0048148 |
|
| **UCSC Genome Browser** |
| uc007imy.1 |
| uc007imz.1 |
| uc007ina.1 |
|
| **WikiGenes** |
| 11548 |
|
| **Affy** |
| 10385353 |
| 1422183\_a\_at |
| 92340\_at |
