# Supplementary material for: Automatically visualise and analyse data on pathways using PathVisioRPC from any programming environment
Source: BMC Bioinformatics. 2015 Aug 23;16(1):267. doi: 10.1186/s12859-015-0708-8 (PMC4546821; doi:10.1186/s12859-015-0708-8)
Supplement: Additional file 3: — Examples in Python. This zip archive contains the data and python script for the three python examples. (ZIP 15714 kb) [file 12859_2015_708_MOESM3_ESM.zip › Python_Examples/result_Example_1/geneList3/backpage/L_11552.html]

 

# geneproduct annotation

  

| Name: Adra2b| Identifier: 11552| Database: Entrez Gene| Synonyms: alpha2B | | | --- | --- | | | | --- | --- | --- | --- | | | | --- | --- | --- | --- | --- | --- | | |
| --- | --- | --- | --- | --- | --- | --- | --- |

# Expression data

**Gene id on mapp: 11552**

| Sample name 11552| SystemCode L| LogFC 0.0| Pvalue 0.348880373| Type trans-PPS2 | | | --- | --- | | | | --- | --- | --- | --- | | | | --- | --- | --- | --- | --- | --- | | | | --- | --- | --- | --- | --- | --- | --- | --- | | |
| --- | --- | --- | --- | --- | --- | --- | --- | --- | --- |

  
  

---

  
  

# Cross references

  

|
|  |
| **UniGene** |
| Mm.347390 |
| Mm.421686 |
|
| **Agilent** |
| A\_51\_P228146 |
| A\_55\_P1994027 |
|
| **Ensembl** |
| ENSMUSG00000058620 |
|
| **Illumina** |
| ILMN\_1215724 |
| ILMN\_1228610 |
| ILMN\_2688190 |
|
| **Entrez Gene** |
| 11552 |
|
| **MGI** |
| MGI:87935 |
|
| **RefSeq** |
| NM\_009633 |
| NP\_033763 |
|
| **Uniprot/TrEMBL** |
| F8VQ23 |
| Q925K6 |
|
| **GeneOntology** |
| GO:0000165 |
| GO:0000187 |
| GO:0001525 |
| GO:0003056 |
| GO:0004935 |
| GO:0004938 |
| GO:0005515 |
| GO:0005886 |
| GO:0007186 |
| GO:0007565 |
| GO:0016021 |
| GO:0032148 |
| GO:0035625 |
| GO:0045666 |
| GO:0045777 |
| GO:0051379 |
| GO:0070474 |
| GO:0071883 |
|
| **UCSC Genome Browser** |
| uc008mfi.1 |
|
| **WikiGenes** |
| 11552 |
|
| **Affy** |
| 10475800 |
| 1439645\_at |
| 1450003\_at |
| 99802\_at |
| Msa.388.0\_at |
