# Supplementary material for: Automatically visualise and analyse data on pathways using PathVisioRPC from any programming environment
Source: BMC Bioinformatics. 2015 Aug 23;16(1):267. doi: 10.1186/s12859-015-0708-8 (PMC4546821; doi:10.1186/s12859-015-0708-8)
Supplement: Additional file 3: — Examples in Python. This zip archive contains the data and python script for the three python examples. (ZIP 15714 kb) [file 12859_2015_708_MOESM3_ESM.zip › Python_Examples/result_Example_1/geneList3/backpage/L_11555.html]

 

# geneproduct annotation

  

| Name: Adrb2| Identifier: 11555| Database: Entrez Gene| Synonyms: Badm | | | --- | --- | | | | --- | --- | --- | --- | | | | --- | --- | --- | --- | --- | --- | | |
| --- | --- | --- | --- | --- | --- | --- | --- |

# Expression data

**Gene id on mapp: 11555**

| Sample name 11555| SystemCode L| LogFC 0.0| Pvalue 0.489189446| Type trans-PPS2 | | | --- | --- | | | | --- | --- | --- | --- | | | | --- | --- | --- | --- | --- | --- | | | | --- | --- | --- | --- | --- | --- | --- | --- | | |
| --- | --- | --- | --- | --- | --- | --- | --- | --- | --- |

  
  

---

  
  

# Cross references

  

|
|  |
| **UniGene** |
| Mm.417445 |
| Mm.5598 |
|
| **Agilent** |
| A\_51\_P487791 |
| A\_55\_P2029558 |
|
| **Ensembl** |
| ENSMUSG00000045730 |
|
| **Illumina** |
| ILMN\_1241610 |
|
| **Entrez Gene** |
| 11555 |
|
| **MGI** |
| MGI:87938 |
|
| **RefSeq** |
| NM\_007420 |
| NP\_031446 |
|
| **Uniprot/TrEMBL** |
| P18762 |
| Q99P03 |
|
| **GeneOntology** |
| GO:0001993 |
| GO:0002024 |
| GO:0002025 |
| GO:0002028 |
| GO:0002032 |
| GO:0004941 |
| GO:0005515 |
| GO:0005634 |
| GO:0005886 |
| GO:0005887 |
| GO:0006898 |
| GO:0007189 |
| GO:0007190 |
| GO:0008179 |
| GO:0009409 |
| GO:0015459 |
| GO:0016020 |
| GO:0016324 |
| GO:0030501 |
| GO:0031398 |
| GO:0031649 |
| GO:0040015 |
| GO:0042803 |
| GO:0043235 |
| GO:0043410 |
| GO:0045453 |
| GO:0045944 |
| GO:0045986 |
| GO:0050873 |
| GO:0051380 |
|
| **UCSC Genome Browser** |
| uc008fcy.1 |
|
| **WikiGenes** |
| 11555 |
|
| **Affy** |
| 10459288 |
| 1437302\_at |
| 93193\_at |
| Msa.2166.0\_s\_at |
| c76960\_rc\_at |
