# Supplementary material for: Automatically visualise and analyse data on pathways using PathVisioRPC from any programming environment
Source: BMC Bioinformatics. 2015 Aug 23;16(1):267. doi: 10.1186/s12859-015-0708-8 (PMC4546821; doi:10.1186/s12859-015-0708-8)
Supplement: Additional file 3: — Examples in Python. This zip archive contains the data and python script for the three python examples. (ZIP 15714 kb) [file 12859_2015_708_MOESM3_ESM.zip › Python_Examples/result_Example_1/geneList3/backpage/L_11564.html]

 

# geneproduct annotation

  

| Name: Adsl| Identifier: 11564| Database: Entrez Gene| Synonyms: Adl | | | --- | --- | | | | --- | --- | --- | --- | | | | --- | --- | --- | --- | --- | --- | | |
| --- | --- | --- | --- | --- | --- | --- | --- |

# Expression data

**Gene id on mapp: 11564**

| Sample name 11564| SystemCode L| LogFC 0.0| Pvalue 0.886795463| Type trans-PPS2 | | | --- | --- | | | | --- | --- | --- | --- | | | | --- | --- | --- | --- | --- | --- | | | | --- | --- | --- | --- | --- | --- | --- | --- | | |
| --- | --- | --- | --- | --- | --- | --- | --- | --- | --- |

  
  

---

  
  

# Cross references

  

|
|  |
| **UniGene** |
| Mm.38151 |
| Mm.446899 |
| Mm.464071 |
| Mm.465094 |
|
| **Agilent** |
| A\_51\_P269320 |
|
| **Ensembl** |
| ENSMUSG00000022407 |
|
| **Illumina** |
| ILMN\_1255090 |
| ILMN\_2597936 |
| ILMN\_2599494 |
|
| **Entrez Gene** |
| 11564 |
|
| **MGI** |
| MGI:103202 |
|
| **RefSeq** |
| NM\_009634 |
| NP\_033764 |
|
| **Uniprot/TrEMBL** |
| E9PZI0 |
| E9Q0A0 |
| E9Q242 |
| E9Q3T7 |
| P54822 |
|
| **GeneOntology** |
| GO:0001666 |
| GO:0004018 |
| GO:0005737 |
| GO:0005739 |
| GO:0005829 |
| GO:0006163 |
| GO:0006167 |
| GO:0006189 |
| GO:0007584 |
| GO:0009060 |
| GO:0009156 |
| GO:0014850 |
| GO:0042594 |
| GO:0044208 |
| GO:0051262 |
| GO:0070626 |
|
| **UCSC Genome Browser** |
| uc007wvz.1 |
|
| **WikiGenes** |
| 11564 |
|
| **Affy** |
| 10425461 |
| 1418372\_at |
| 98999\_at |
| Msa.4213.0\_s\_at |
