# Supplementary material for: Automatically visualise and analyse data on pathways using PathVisioRPC from any programming environment
Source: BMC Bioinformatics. 2015 Aug 23;16(1):267. doi: 10.1186/s12859-015-0708-8 (PMC4546821; doi:10.1186/s12859-015-0708-8)
Supplement: Additional file 3: — Examples in Python. This zip archive contains the data and python script for the three python examples. (ZIP 15714 kb) [file 12859_2015_708_MOESM3_ESM.zip › Python_Examples/result_Example_1/geneList3/backpage/L_11565.html]

 

# geneproduct annotation

  

| Name: Adssl1| Identifier: 11565| Database: Entrez Gene| Synonyms: Adss | | | --- | --- | | | | --- | --- | --- | --- | | | | --- | --- | --- | --- | --- | --- | | |
| --- | --- | --- | --- | --- | --- | --- | --- |

# Expression data

**Gene id on mapp: 11565**

| Sample name 11565| SystemCode L| LogFC 0.0| Pvalue 0.724629736| Type trans-PPS2 | | | --- | --- | | | | --- | --- | --- | --- | | | | --- | --- | --- | --- | --- | --- | | | | --- | --- | --- | --- | --- | --- | --- | --- | | |
| --- | --- | --- | --- | --- | --- | --- | --- | --- | --- |

  
  

---

  
  

# Cross references

  

|
|  |
| **UniGene** |
| Mm.3440 |
|
| **Agilent** |
| A\_51\_P190740 |
| A\_55\_P2030030 |
|
| **Ensembl** |
| ENSMUSG00000011148 |
|
| **Illumina** |
| ILMN\_1245079 |
| ILMN\_2711688 |
| ILMN\_2958099 |
|
| **Entrez Gene** |
| 11565 |
|
| **MGI** |
| MGI:87947 |
|
| **PDB** |
| 1IWE |
| 1J4B |
| 1LNY |
| 1LON |
| 1LOO |
| 1MEZ |
| 1MF0 |
| 1MF1 |
| 2DGN |
|
| **RefSeq** |
| NM\_007421 |
| NP\_031447 |
|
| **Uniprot/TrEMBL** |
| J3QN31 |
| P28650 |
| Q3UBP0 |
|
| **GeneOntology** |
| GO:0000287 |
| GO:0004019 |
| GO:0005515 |
| GO:0005525 |
| GO:0005737 |
| GO:0006163 |
| GO:0006167 |
| GO:0016020 |
| GO:0044208 |
|
| **UCSC Genome Browser** |
| uc007peu.2 |
| uc011yvf.1 |
|
| **WikiGenes** |
| 11565 |
|
| **Affy** |
| 10398859 |
| 1449383\_at |
| 98435\_at |
| M74495\_s\_at |
