# Supplementary material for: Automatically visualise and analyse data on pathways using PathVisioRPC from any programming environment
Source: BMC Bioinformatics. 2015 Aug 23;16(1):267. doi: 10.1186/s12859-015-0708-8 (PMC4546821; doi:10.1186/s12859-015-0708-8)
Supplement: Additional file 3: — Examples in Python. This zip archive contains the data and python script for the three python examples. (ZIP 15714 kb) [file 12859_2015_708_MOESM3_ESM.zip › Python_Examples/result_Example_1/geneList3/backpage/L_11566.html]

 

# geneproduct annotation

  

| Name: Adss| Identifier: 11566| Database: Entrez Gene| Synonyms: AS | | | --- | --- | | | | --- | --- | --- | --- | | | | --- | --- | --- | --- | --- | --- | | |
| --- | --- | --- | --- | --- | --- | --- | --- |

# Expression data

**Gene id on mapp: 11566**

| Sample name 11566| SystemCode L| LogFC 0.0| Pvalue 0.185149769| Type trans-PPS2 | | | --- | --- | | | | --- | --- | --- | --- | | | | --- | --- | --- | --- | --- | --- | | | | --- | --- | --- | --- | --- | --- | --- | --- | | |
| --- | --- | --- | --- | --- | --- | --- | --- | --- | --- |

  
  

---

  
  

# Cross references

  

|
|  |
| **UniGene** |
| Mm.338021 |
| Mm.470004 |
|
| **Agilent** |
| A\_51\_P361099 |
| A\_52\_P206613 |
|
| **Ensembl** |
| ENSMUSG00000015961 |
|
| **Illumina** |
| ILMN\_1251260 |
| ILMN\_2629543 |
| ILMN\_2957614 |
|
| **Entrez Gene** |
| 11566 |
|
| **MGI** |
| MGI:87948 |
|
| **RefSeq** |
| NM\_007422 |
| NP\_031448 |
|
| **Uniprot/TrEMBL** |
| B9EIE9 |
| P46664 |
|
| **GeneOntology** |
| GO:0000287 |
| GO:0004019 |
| GO:0005525 |
| GO:0005737 |
| GO:0005829 |
| GO:0005886 |
| GO:0006164 |
| GO:0006167 |
| GO:0006184 |
| GO:0006531 |
| GO:0014074 |
| GO:0044208 |
| GO:0046040 |
| GO:0060359 |
| GO:0071257 |
|
| **UCSC Genome Browser** |
| uc007dut.1 |
|
| **WikiGenes** |
| 11566 |
|
| **Affy** |
| 10360522 |
| 1460726\_at |
| 99038\_at |
| 99039\_g\_at |
| L24554\_s\_at |
