# Supplementary material for: Automatically visualise and analyse data on pathways using PathVisioRPC from any programming environment
Source: BMC Bioinformatics. 2015 Aug 23;16(1):267. doi: 10.1186/s12859-015-0708-8 (PMC4546821; doi:10.1186/s12859-015-0708-8)
Supplement: Additional file 3: — Examples in Python. This zip archive contains the data and python script for the three python examples. (ZIP 15714 kb) [file 12859_2015_708_MOESM3_ESM.zip › Python_Examples/result_Example_1/geneList3/backpage/L_11567.html]

 

# geneproduct annotation

  

| Name: Avil| Identifier: 11567| Database: Entrez Gene| Synonyms: Advil | | | --- | --- | | | | --- | --- | --- | --- | | | | --- | --- | --- | --- | --- | --- | | |
| --- | --- | --- | --- | --- | --- | --- | --- |

# Expression data

**Gene id on mapp: 11567**

| Sample name 11567| SystemCode L| LogFC 0.0| Pvalue 0.384593453| Type trans-PPS2 | | | --- | --- | | | | --- | --- | --- | --- | | | | --- | --- | --- | --- | --- | --- | | | | --- | --- | --- | --- | --- | --- | --- | --- | | |
| --- | --- | --- | --- | --- | --- | --- | --- | --- | --- |

  
  

---

  
  

# Cross references

  

|
|  |
| **UniGene** |
| Mm.10739 |
|
| **Agilent** |
| A\_51\_P166339 |
|
| **Ensembl** |
| ENSMUSG00000025432 |
|
| **Illumina** |
| ILMN\_2452201 |
| ILMN\_2753147 |
| ILMN\_2753149 |
|
| **Entrez Gene** |
| 11567 |
|
| **MGI** |
| MGI:1333798 |
|
| **RefSeq** |
| NM\_009635 |
| NP\_033765 |
|
| **Uniprot/TrEMBL** |
| D3YXP4 |
| D3Z3N6 |
| O88398 |
| Q3TBC1 |
|
| **GeneOntology** |
| GO:0003779 |
| GO:0005515 |
| GO:0005737 |
| GO:0005856 |
| GO:0006950 |
| GO:0007015 |
| GO:0010976 |
| GO:0030424 |
| GO:0042995 |
| GO:0043005 |
| GO:0051693 |
| GO:0060271 |
|
| **UCSC Genome Browser** |
| uc007hhl.2 |
|
| **WikiGenes** |
| 11567 |
|
| **Affy** |
| 102249\_at |
| 10366774 |
| 1419148\_at |
