# Supplementary material for: Automatically visualise and analyse data on pathways using PathVisioRPC from any programming environment
Source: BMC Bioinformatics. 2015 Aug 23;16(1):267. doi: 10.1186/s12859-015-0708-8 (PMC4546821; doi:10.1186/s12859-015-0708-8)
Supplement: Additional file 3: — Examples in Python. This zip archive contains the data and python script for the three python examples. (ZIP 15714 kb) [file 12859_2015_708_MOESM3_ESM.zip › Python_Examples/result_Example_1/geneList3/backpage/L_11568.html]

 

# geneproduct annotation

  

| Name: Aebp1| Identifier: 11568| Database: Entrez Gene| Synonyms: ACLP | | | --- | --- | | | | --- | --- | --- | --- | | | | --- | --- | --- | --- | --- | --- | | |
| --- | --- | --- | --- | --- | --- | --- | --- |

# Expression data

**Gene id on mapp: 11568**

| Sample name 11568| SystemCode L| LogFC 1.180495047| Pvalue 0.042365104| Type trans-PPS2 | | | --- | --- | | | | --- | --- | --- | --- | | | | --- | --- | --- | --- | --- | --- | | | | --- | --- | --- | --- | --- | --- | --- | --- | | |
| --- | --- | --- | --- | --- | --- | --- | --- | --- | --- |

  
  

---

  
  

# Cross references

  

|
|  |
| **UniGene** |
| Mm.4665 |
| Mm.481533 |
|
| **Agilent** |
| A\_51\_P336770 |
| A\_55\_P2035662 |
|
| **Ensembl** |
| ENSMUSG00000020473 |
|
| **Illumina** |
| ILMN\_2671895 |
| ILMN\_2873822 |
|
| **Entrez Gene** |
| 11568 |
|
| **MGI** |
| MGI:1197012 |
|
| **RefSeq** |
| NM\_009636 |
| NP\_033766 |
|
| **Uniprot/TrEMBL** |
| Q640N1 |
|
| **GeneOntology** |
| GO:0003677 |
| GO:0003714 |
| GO:0004180 |
| GO:0004181 |
| GO:0005516 |
| GO:0005615 |
| GO:0005634 |
| GO:0005737 |
| GO:0006351 |
| GO:0006355 |
| GO:0006508 |
| GO:0007155 |
| GO:0008270 |
| GO:0031012 |
|
| **UCSC Genome Browser** |
| uc007hxg.2 |
| uc007hxj.2 |
|
| **WikiGenes** |
| 11568 |
|
| **Affy** |
| 100411\_at |
| 100412\_g\_at |
| 10374083 |
| 1422514\_at |
| 1450637\_a\_at |
| X80478\_s\_at |
