# Supplementary material for: Automatically visualise and analyse data on pathways using PathVisioRPC from any programming environment
Source: BMC Bioinformatics. 2015 Aug 23;16(1):267. doi: 10.1186/s12859-015-0708-8 (PMC4546821; doi:10.1186/s12859-015-0708-8)
Supplement: Additional file 3: — Examples in Python. This zip archive contains the data and python script for the three python examples. (ZIP 15714 kb) [file 12859_2015_708_MOESM3_ESM.zip › Python_Examples/result_Example_1/geneList3/backpage/L_11569.html]

 

# geneproduct annotation

  

| Name: Aebp2| Identifier: 11569| Database: Entrez Gene| Synonyms: AU023766 | | | --- | --- | | | | --- | --- | --- | --- | | | | --- | --- | --- | --- | --- | --- | | |
| --- | --- | --- | --- | --- | --- | --- | --- |

# Expression data

**Gene id on mapp: 11569**

| Sample name 11569| SystemCode L| LogFC 0.0| Pvalue 0.18700565| Type trans-PPS2 | | | --- | --- | | | | --- | --- | --- | --- | | | | --- | --- | --- | --- | --- | --- | | | | --- | --- | --- | --- | --- | --- | --- | --- | | |
| --- | --- | --- | --- | --- | --- | --- | --- | --- | --- |

  
  

---

  
  

# Cross references

  

|
|  |
| **UniGene** |
| Mm.395312 |
| Mm.401473 |
| Mm.470238 |
| Mm.479349 |
|
| **Agilent** |
| A\_52\_P23212 |
| A\_52\_P59422 |
| A\_55\_P2165539 |
| A\_55\_P2165544 |
|
| **Ensembl** |
| ENSMUSG00000030232 |
|
| **Illumina** |
| ILMN\_1215960 |
| ILMN\_1237360 |
| ILMN\_1258253 |
| ILMN\_2562670 |
| ILMN\_3015437 |
| ILMN\_3040064 |
| ILMN\_3138674 |
|
| **Entrez Gene** |
| 11569 |
|
| **MGI** |
| MGI:1338038 |
|
| **RefSeq** |
| NM\_001005605 |
| NM\_009637 |
| NM\_178803 |
| NP\_001005605 |
| NP\_033767 |
| NP\_848918 |
|
| **Uniprot/TrEMBL** |
| E0CYG6 |
| F6V059 |
| F8WGQ1 |
| Q9Z248 |
|
| **GeneOntology** |
| GO:0003677 |
| GO:0003714 |
| GO:0005634 |
| GO:0006351 |
| GO:0006355 |
| GO:0008270 |
| GO:0016568 |
| GO:0035098 |
|
| **UCSC Genome Browser** |
| uc009eoi.1 |
| uc009eoj.1 |
| uc009eok.1 |
| uc009eol.1 |
|
| **WikiGenes** |
| 11569 |
|
| **Affy** |
| 10542557 |
| 1421021\_at |
| 1434080\_at |
| 1435070\_at |
| 1437743\_at |
| 94869\_at |
