# Supplementary material for: Automatically visualise and analyse data on pathways using PathVisioRPC from any programming environment
Source: BMC Bioinformatics. 2015 Aug 23;16(1):267. doi: 10.1186/s12859-015-0708-8 (PMC4546821; doi:10.1186/s12859-015-0708-8)
Supplement: Additional file 3: — Examples in Python. This zip archive contains the data and python script for the three python examples. (ZIP 15714 kb) [file 12859_2015_708_MOESM3_ESM.zip › Python_Examples/result_Example_1/geneList3/backpage/L_11576.html]

 

# geneproduct annotation

  

| Name: Afp| Identifier: 11576| Database: Entrez Gene | | | --- | --- | | | | --- | --- | --- | --- | | |
| --- | --- | --- | --- | --- | --- |

# Expression data

**Gene id on mapp: 11576**

| Sample name 11576| SystemCode L| LogFC 0.0| Pvalue 0.881087129| Type trans-PPS2 | | | --- | --- | | | | --- | --- | --- | --- | | | | --- | --- | --- | --- | --- | --- | | | | --- | --- | --- | --- | --- | --- | --- | --- | | |
| --- | --- | --- | --- | --- | --- | --- | --- | --- | --- |

  
  

---

  
  

# Cross references

  

|
|  |
| **UniGene** |
| Mm.358570 |
| Mm.398486 |
|
| **Agilent** |
| A\_51\_P510891 |
|
| **Ensembl** |
| ENSMUSG00000054932 |
|
| **Illumina** |
| ILMN\_1245472 |
|
| **Entrez Gene** |
| 11576 |
|
| **MGI** |
| MGI:87951 |
|
| **RefSeq** |
| NM\_007423 |
| NP\_031449 |
|
| **Uniprot/TrEMBL** |
| P02772 |
| Q3TGA3 |
|
| **GeneOntology** |
| GO:0001542 |
| GO:0005615 |
| GO:0005737 |
| GO:0006810 |
| GO:0019953 |
| GO:0042448 |
| GO:0046872 |
| GO:0060395 |
|
| **UCSC Genome Browser** |
| uc008yba.2 |
|
| **WikiGenes** |
| 11576 |
|
| **Affy** |
| 10523078 |
| 1416645\_a\_at |
| 1416646\_at |
| 1436879\_x\_at |
| Msa.412.0\_f\_at |
| v00743\_f\_at |
