# Supplementary material for: Automatically visualise and analyse data on pathways using PathVisioRPC from any programming environment
Source: BMC Bioinformatics. 2015 Aug 23;16(1):267. doi: 10.1186/s12859-015-0708-8 (PMC4546821; doi:10.1186/s12859-015-0708-8)
Supplement: Additional file 3: — Examples in Python. This zip archive contains the data and python script for the three python examples. (ZIP 15714 kb) [file 12859_2015_708_MOESM3_ESM.zip › Python_Examples/result_Example_1/geneList3/backpage/L_11593.html]

 

# geneproduct annotation

  

| Name: Aga| Identifier: 11593| Database: Entrez Gene| Synonyms: AW060726 | | | --- | --- | | | | --- | --- | --- | --- | | | | --- | --- | --- | --- | --- | --- | | |
| --- | --- | --- | --- | --- | --- | --- | --- |

# Expression data

**Gene id on mapp: 11593**

| Sample name 11593| SystemCode L| LogFC 1.271417432| Pvalue 0.001142798| Type trans-PPS2 | | | --- | --- | | | | --- | --- | --- | --- | | | | --- | --- | --- | --- | --- | --- | | | | --- | --- | --- | --- | --- | --- | --- | --- | | |
| --- | --- | --- | --- | --- | --- | --- | --- | --- | --- |

  
  

---

  
  

# Cross references

  

|
|  |
| **Agilent** |
| A\_51\_P119544 |
|
| **Ensembl** |
| ENSMUSG00000031521 |
|
| **Illumina** |
| ILMN\_2673077 |
|
| **Entrez Gene** |
| 11593 |
|
| **MGI** |
| MGI:104873 |
|
| **RefSeq** |
| NM\_001005847 |
| NM\_001205054 |
| NP\_001005847 |
| NP\_001191983 |
|
| **Uniprot/TrEMBL** |
| A2RSS6 |
| B7ZNK6 |
| Q64191 |
|
| **GeneOntology** |
| GO:0003948 |
| GO:0005764 |
| GO:0005783 |
| GO:0006508 |
| GO:0006517 |
| GO:0008233 |
| GO:0043621 |
| GO:0051604 |
|
| **UCSC Genome Browser** |
| uc009lrz.2 |
|
| **WikiGenes** |
| 11593 |
|
| **Affy** |
| 10571774 |
| 1434665\_at |
| 163631\_at |
