# Supplementary material for: Automatically visualise and analyse data on pathways using PathVisioRPC from any programming environment
Source: BMC Bioinformatics. 2015 Aug 23;16(1):267. doi: 10.1186/s12859-015-0708-8 (PMC4546821; doi:10.1186/s12859-015-0708-8)
Supplement: Additional file 3: — Examples in Python. This zip archive contains the data and python script for the three python examples. (ZIP 15714 kb) [file 12859_2015_708_MOESM3_ESM.zip › Python_Examples/result_Example_1/geneList3/backpage/L_11596.html]

 

# geneproduct annotation

  

| Name: Ager| Identifier: 11596| Database: Entrez Gene| Synonyms: RAGE | | | --- | --- | | | | --- | --- | --- | --- | | | | --- | --- | --- | --- | --- | --- | | |
| --- | --- | --- | --- | --- | --- | --- | --- |

# Expression data

**Gene id on mapp: 11596**

| Sample name 11596| SystemCode L| LogFC 0.0| Pvalue 0.239005325| Type trans-PPS2 | | | --- | --- | | | | --- | --- | --- | --- | | | | --- | --- | --- | --- | --- | --- | | | | --- | --- | --- | --- | --- | --- | --- | --- | | |
| --- | --- | --- | --- | --- | --- | --- | --- | --- | --- |

  
  

---

  
  

# Cross references

  

|
|  |
| **UniGene** |
| Mm.3383 |
|
| **Agilent** |
| A\_52\_P509965 |
|
| **Ensembl** |
| ENSMUSG00000015452 |
|
| **Illumina** |
| ILMN\_1224092 |
| ILMN\_1239451 |
|
| **Entrez Gene** |
| 11596 |
|
| **MGI** |
| MGI:893592 |
|
| **RefSeq** |
| NM\_001271422 |
| NM\_001271423 |
| NM\_007425 |
| NP\_001258351 |
| NP\_001258352 |
| NP\_001258353 |
| NP\_031451 |
|
| **Uniprot/TrEMBL** |
| C5H3H4 |
| C5H3H5 |
| C5H3H7 |
| C5H7W9 |
| O35444 |
|
| **GeneOntology** |
| GO:0005515 |
| GO:0016020 |
| GO:0031175 |
| GO:0044548 |
| GO:0050930 |
| GO:0051092 |
|
| **UCSC Genome Browser** |
| uc008ccw.2 |
| uc008ccx.1 |
| uc012aqf.1 |
| uc012aqg.1 |
|
| **WikiGenes** |
| 11596 |
|
| **Affy** |
| 10444407 |
| 1420428\_at |
| 1441958\_s\_at |
| 92181\_at |
| l33412\_s\_at |
