# Supplementary material for: Automatically visualise and analyse data on pathways using PathVisioRPC from any programming environment
Source: BMC Bioinformatics. 2015 Aug 23;16(1):267. doi: 10.1186/s12859-015-0708-8 (PMC4546821; doi:10.1186/s12859-015-0708-8)
Supplement: Additional file 3: — Examples in Python. This zip archive contains the data and python script for the three python examples. (ZIP 15714 kb) [file 12859_2015_708_MOESM3_ESM.zip › Python_Examples/result_Example_1/geneList3/backpage/L_11601.html]

 

# geneproduct annotation

  

| Name: Angpt2| Identifier: 11601| Database: Entrez Gene| Synonyms: Ang2 | | | --- | --- | | | | --- | --- | --- | --- | | | | --- | --- | --- | --- | --- | --- | | |
| --- | --- | --- | --- | --- | --- | --- | --- |

# Expression data

**Gene id on mapp: 11601**

| Sample name 11601| SystemCode L| LogFC 0.0| Pvalue 0.537841564| Type trans-PPS2 | | | --- | --- | | | | --- | --- | --- | --- | | | | --- | --- | --- | --- | --- | --- | | | | --- | --- | --- | --- | --- | --- | --- | --- | | |
| --- | --- | --- | --- | --- | --- | --- | --- | --- | --- |

  
  

---

  
  

# Cross references

  

|
|  |
| **UniGene** |
| Mm.439874 |
| Mm.455721 |
|
| **Agilent** |
| A\_51\_P201982 |
|
| **Ensembl** |
| ENSMUSG00000031465 |
|
| **Illumina** |
| ILMN\_1234487 |
| ILMN\_2642165 |
|
| **Entrez Gene** |
| 11601 |
|
| **MGI** |
| MGI:1202890 |
|
| **RefSeq** |
| NM\_007426 |
| NP\_031452 |
|
| **Uniprot/TrEMBL** |
| B9EHQ4 |
| O35608 |
|
| **GeneOntology** |
| GO:0001525 |
| GO:0001666 |
| GO:0001974 |
| GO:0005172 |
| GO:0005515 |
| GO:0005615 |
| GO:0005634 |
| GO:0005886 |
| GO:0007169 |
| GO:0007281 |
| GO:0007492 |
| GO:0009314 |
| GO:0009612 |
| GO:0009749 |
| GO:0010812 |
| GO:0014070 |
| GO:0014823 |
| GO:0016525 |
| GO:0030097 |
| GO:0030971 |
| GO:0031100 |
| GO:0042995 |
| GO:0043537 |
| GO:0045765 |
| GO:0045766 |
| GO:0046872 |
| GO:0048014 |
| GO:0048514 |
| GO:0050928 |
| GO:0060135 |
| GO:0071363 |
| GO:0072012 |
|
| **UCSC Genome Browser** |
| uc009kzu.1 |
|
| **WikiGenes** |
| 11601 |
|
| **Affy** |
| 10577315 |
| 1448831\_at |
| 1459636\_at |
| 92210\_at |
| AF004326\_at |
| AF004326\_g\_at |
