# Supplementary material for: Automatically visualise and analyse data on pathways using PathVisioRPC from any programming environment
Source: BMC Bioinformatics. 2015 Aug 23;16(1):267. doi: 10.1186/s12859-015-0708-8 (PMC4546821; doi:10.1186/s12859-015-0708-8)
Supplement: Additional file 3: — Examples in Python. This zip archive contains the data and python script for the three python examples. (ZIP 15714 kb) [file 12859_2015_708_MOESM3_ESM.zip › Python_Examples/result_Example_1/geneList3/backpage/L_11603.html]

 

# geneproduct annotation

  

| Name: Agrn| Identifier: 11603| Database: Entrez Gene| Synonyms: nmf380 | | | --- | --- | | | | --- | --- | --- | --- | | | | --- | --- | --- | --- | --- | --- | | |
| --- | --- | --- | --- | --- | --- | --- | --- |

# Expression data

**Gene id on mapp: 11603**

| Sample name 11603| SystemCode L| LogFC 1.319383599| Pvalue 4.35315E-4| Type trans-PPS2 | | | --- | --- | | | | --- | --- | --- | --- | | | | --- | --- | --- | --- | --- | --- | | | | --- | --- | --- | --- | --- | --- | --- | --- | | |
| --- | --- | --- | --- | --- | --- | --- | --- | --- | --- |

  
  

---

  
  

# Cross references

  

|
|  |
| **UniGene** |
| Mm.273098 |
| Mm.409890 |
| Mm.431676 |
|
| **Agilent** |
| A\_30\_P01019456 |
| A\_30\_P01029969 |
| A\_30\_P01030529 |
| A\_30\_P01033628 |
| A\_51\_P229833 |
| A\_51\_P292276 |
| A\_55\_P2009121 |
| A\_55\_P2009127 |
|
| **Ensembl** |
| ENSMUSG00000041936 |
|
| **Illumina** |
| ILMN\_1217856 |
| ILMN\_1228650 |
| ILMN\_1252685 |
| ILMN\_2674367 |
|
| **Entrez Gene** |
| 11603 |
|
| **MGI** |
| MGI:87961 |
|
| **PDB** |
| 3PVE |
|
| **RefSeq** |
| NM\_021604 |
| NP\_067617 |
|
| **Uniprot/TrEMBL** |
| A2ASQ1 |
| M0QWP1 |
| O08860 |
| Q52L98 |
| Q6PCM6 |
| Q80V56 |
| Q8K326 |
| Q9ERP2 |
|
| **GeneOntology** |
| GO:0001934 |
| GO:0002162 |
| GO:0005509 |
| GO:0005515 |
| GO:0005576 |
| GO:0005604 |
| GO:0005605 |
| GO:0005615 |
| GO:0005796 |
| GO:0005886 |
| GO:0007009 |
| GO:0007268 |
| GO:0007528 |
| GO:0008582 |
| GO:0009986 |
| GO:0016021 |
| GO:0030054 |
| GO:0030548 |
| GO:0031012 |
| GO:0032314 |
| GO:0032321 |
| GO:0033691 |
| GO:0035374 |
| GO:0043113 |
| GO:0043395 |
| GO:0043525 |
| GO:0045202 |
| GO:0045213 |
| GO:0045887 |
| GO:0045944 |
| GO:0051491 |
| GO:2000541 |
|
| **UCSC Genome Browser** |
| uc008wgf.2 |
| uc008wgg.1 |
|
| **WikiGenes** |
| 11603 |
|
| **Affy** |
| 104763\_at |
| 10519270 |
| 1426670\_at |
| 1443980\_at |
| 97921\_at |
| aa273938\_s\_at |
