# Supplementary material for: Automatically visualise and analyse data on pathways using PathVisioRPC from any programming environment
Source: BMC Bioinformatics. 2015 Aug 23;16(1):267. doi: 10.1186/s12859-015-0708-8 (PMC4546821; doi:10.1186/s12859-015-0708-8)
Supplement: Additional file 3: — Examples in Python. This zip archive contains the data and python script for the three python examples. (ZIP 15714 kb) [file 12859_2015_708_MOESM3_ESM.zip › Python_Examples/result_Example_1/geneList3/backpage/L_11604.html]

 

# geneproduct annotation

  

| Name: Agrp| Identifier: 11604| Database: Entrez Gene| Synonyms: Art | | | --- | --- | | | | --- | --- | --- | --- | | | | --- | --- | --- | --- | --- | --- | | |
| --- | --- | --- | --- | --- | --- | --- | --- |

# Expression data

**Gene id on mapp: 11604**

| Sample name 11604| SystemCode L| LogFC 0.0| Pvalue 0.098455094| Type trans-PPS2 | | | --- | --- | | | | --- | --- | --- | --- | | | | --- | --- | --- | --- | --- | --- | | | | --- | --- | --- | --- | --- | --- | --- | --- | | |
| --- | --- | --- | --- | --- | --- | --- | --- | --- | --- |

  
  

---

  
  

# Cross references

  

|
|  |
| **UniGene** |
| Mm.491630 |
|
| **Agilent** |
| A\_51\_P347965 |
| A\_55\_P2049717 |
|
| **Ensembl** |
| ENSMUSG00000005705 |
|
| **Illumina** |
| ILMN\_2703427 |
|
| **Entrez Gene** |
| 11604 |
|
| **MGI** |
| MGI:892013 |
|
| **RefSeq** |
| NM\_007427 |
| NP\_001258735 |
| NP\_031453 |
|
| **Uniprot/TrEMBL** |
| P56473 |
| Q3UU47 |
| Q5J6B1 |
|
| **GeneOntology** |
| GO:0005184 |
| GO:0005615 |
| GO:0005796 |
| GO:0007218 |
| GO:0008343 |
| GO:0009755 |
| GO:0031781 |
| GO:0031782 |
| GO:0060259 |
|
| **UCSC Genome Browser** |
| uc009ndh.1 |
|
| **WikiGenes** |
| 11604 |
|
| **Affy** |
| 10581299 |
| 1421690\_s\_at |
| 99399\_at |
