# Supplementary material for: Automatically visualise and analyse data on pathways using PathVisioRPC from any programming environment
Source: BMC Bioinformatics. 2015 Aug 23;16(1):267. doi: 10.1186/s12859-015-0708-8 (PMC4546821; doi:10.1186/s12859-015-0708-8)
Supplement: Additional file 3: — Examples in Python. This zip archive contains the data and python script for the three python examples. (ZIP 15714 kb) [file 12859_2015_708_MOESM3_ESM.zip › Python_Examples/result_Example_1/geneList3/backpage/L_11605.html]

 

# geneproduct annotation

  

| Name: Gla| Identifier: 11605| Database: Entrez Gene| Synonyms: Ags | | | --- | --- | | | | --- | --- | --- | --- | | | | --- | --- | --- | --- | --- | --- | | |
| --- | --- | --- | --- | --- | --- | --- | --- |

# Expression data

**Gene id on mapp: 11605**

| Sample name 11605| SystemCode L| LogFC -1.246761748| Pvalue 0.037526647| Type trans-PPS2 | | | --- | --- | | | | --- | --- | --- | --- | | | | --- | --- | --- | --- | --- | --- | | | | --- | --- | --- | --- | --- | --- | --- | --- | | |
| --- | --- | --- | --- | --- | --- | --- | --- | --- | --- |

  
  

---

  
  

# Cross references

  

|
|  |
| **Agilent** |
| A\_52\_P234073 |
| A\_55\_P2146136 |
|
| **Ensembl** |
| ENSMUSG00000031266 |
|
| **Illumina** |
| ILMN\_1220292 |
| ILMN\_2626086 |
|
| **Entrez Gene** |
| 11605 |
|
| **MGI** |
| MGI:1347344 |
|
| **RefSeq** |
| NM\_013463 |
| NP\_038491 |
|
| **Uniprot/TrEMBL** |
| A2BDV6 |
| P51569 |
| Q8BGZ6 |
|
| **GeneOntology** |
| GO:0003824 |
| GO:0004557 |
| GO:0005102 |
| GO:0005515 |
| GO:0005576 |
| GO:0005737 |
| GO:0005764 |
| GO:0005794 |
| GO:0009311 |
| GO:0016139 |
| GO:0016936 |
| GO:0042803 |
| GO:0045019 |
| GO:0046477 |
| GO:0051001 |
| GO:0052692 |
|
| **UCSC Genome Browser** |
| uc009ugg.1 |
|
| **WikiGenes** |
| 11605 |
|
| **Affy** |
| 102341\_at |
| 10606714 |
| 1418248\_at |
| 1449006\_at |
| Msa.1610.0\_s\_at |
| u34071\_s\_at |
