# Supplementary material for: Automatically visualise and analyse data on pathways using PathVisioRPC from any programming environment
Source: BMC Bioinformatics. 2015 Aug 23;16(1):267. doi: 10.1186/s12859-015-0708-8 (PMC4546821; doi:10.1186/s12859-015-0708-8)
Supplement: Additional file 3: — Examples in Python. This zip archive contains the data and python script for the three python examples. (ZIP 15714 kb) [file 12859_2015_708_MOESM3_ESM.zip › Python_Examples/result_Example_1/geneList3/backpage/L_11606.html]

 

# geneproduct annotation

  

| Name: Agt| Identifier: 11606| Database: Entrez Gene| Synonyms: Aogen | | | --- | --- | | | | --- | --- | --- | --- | | | | --- | --- | --- | --- | --- | --- | | |
| --- | --- | --- | --- | --- | --- | --- | --- |

# Expression data

**Gene id on mapp: 11606**

| Sample name 11606| SystemCode L| LogFC 0.0| Pvalue 0.10161371| Type trans-PPS2 | | | --- | --- | | | | --- | --- | --- | --- | | | | --- | --- | --- | --- | --- | --- | | | | --- | --- | --- | --- | --- | --- | --- | --- | | |
| --- | --- | --- | --- | --- | --- | --- | --- | --- | --- |

  
  

---

  
  

# Cross references

  

|
|  |
| **UniGene** |
| Mm.301626 |
|
| **Agilent** |
| A\_51\_P323712 |
|
| **Ensembl** |
| ENSMUSG00000031980 |
|
| **Illumina** |
| ILMN\_1227398 |
|
| **Entrez Gene** |
| 11606 |
|
| **MGI** |
| MGI:87963 |
|
| **RefSeq** |
| NM\_007428 |
| NP\_031454 |
|
| **Uniprot/TrEMBL** |
| Q3UTR7 |
|
| **GeneOntology** |
| GO:0001543 |
| GO:0001568 |
| GO:0001658 |
| GO:0001822 |
| GO:0001991 |
| GO:0001998 |
| GO:0001999 |
| GO:0002018 |
| GO:0002019 |
| GO:0002035 |
| GO:0003051 |
| GO:0003331 |
| GO:0004867 |
| GO:0005179 |
| GO:0005615 |
| GO:0006883 |
| GO:0006917 |
| GO:0007160 |
| GO:0007166 |
| GO:0007186 |
| GO:0007202 |
| GO:0007250 |
| GO:0007568 |
| GO:0007588 |
| GO:0008065 |
| GO:0008217 |
| GO:0008285 |
| GO:0009409 |
| GO:0009651 |
| GO:0010468 |
| GO:0010535 |
| GO:0010595 |
| GO:0010613 |
| GO:0010628 |
| GO:0010698 |
| GO:0010873 |
| GO:0010951 |
| GO:0014061 |
| GO:0014068 |
| GO:0014824 |
| GO:0014873 |
| GO:0030162 |
| GO:0030198 |
| GO:0030308 |
| GO:0030432 |
| GO:0031701 |
| GO:0031702 |
| GO:0031703 |
| GO:0032270 |
| GO:0032930 |
| GO:0034104 |
| GO:0035411 |
| GO:0035813 |
| GO:0035815 |
| GO:0040018 |
| GO:0042310 |
| GO:0042311 |
| GO:0042445 |
| GO:0042756 |
| GO:0042981 |
| GO:0043065 |
| GO:0043085 |
| GO:0043410 |
| GO:0043524 |
| GO:0044444 |
| GO:0045723 |
| GO:0045742 |
| GO:0045777 |
| GO:0045893 |
| GO:0046622 |
| GO:0048143 |
| GO:0048144 |
| GO:0048146 |
| GO:0048169 |
| GO:0048659 |
| GO:0050663 |
| GO:0050727 |
| GO:0050731 |
| GO:0051145 |
| GO:0051387 |
| GO:0051403 |
| GO:0051924 |
| GO:0061049 |
| GO:0061098 |
| GO:0070371 |
| GO:0071260 |
| GO:0090190 |
|
| **UCSC Genome Browser** |
| uc009nxe.1 |
|
| **WikiGenes** |
| 11606 |
|
| **Affy** |
| 101887\_at |
| 10582658 |
| 1423396\_at |
| Msa.6114.0\_s\_at |
| Msa.7127.0\_s\_at |
| Msa.8622.0\_s\_at |
