# Supplementary material for: Automatically visualise and analyse data on pathways using PathVisioRPC from any programming environment
Source: BMC Bioinformatics. 2015 Aug 23;16(1):267. doi: 10.1186/s12859-015-0708-8 (PMC4546821; doi:10.1186/s12859-015-0708-8)
Supplement: Additional file 3: — Examples in Python. This zip archive contains the data and python script for the three python examples. (ZIP 15714 kb) [file 12859_2015_708_MOESM3_ESM.zip › Python_Examples/result_Example_1/geneList3/backpage/L_11607.html]

 

# geneproduct annotation

  

| Name: Agtr1a| Identifier: 11607| Database: Entrez Gene| Synonyms: AT1a | | | --- | --- | | | | --- | --- | --- | --- | | | | --- | --- | --- | --- | --- | --- | | |
| --- | --- | --- | --- | --- | --- | --- | --- |

# Expression data

**Gene id on mapp: 11607**

| Sample name 11607| SystemCode L| LogFC -1.233967042| Pvalue 0.001011708| Type trans-PPS2 | | | --- | --- | | | | --- | --- | --- | --- | | | | --- | --- | --- | --- | --- | --- | | | | --- | --- | --- | --- | --- | --- | --- | --- | | |
| --- | --- | --- | --- | --- | --- | --- | --- | --- | --- |

  
  

---

  
  

# Cross references

  

|
|  |
| **UniGene** |
| Mm.35062 |
|
| **Agilent** |
| A\_51\_P236439 |
| A\_52\_P645862 |
|
| **Ensembl** |
| ENSMUSG00000049115 |
|
| **Illumina** |
| ILMN\_1248300 |
| ILMN\_2972521 |
|
| **Entrez Gene** |
| 11607 |
|
| **MGI** |
| MGI:87964 |
|
| **RefSeq** |
| NM\_177322 |
| NP\_796296 |
|
| **Uniprot/TrEMBL** |
| P29754 |
|
| **GeneOntology** |
| GO:0001568 |
| GO:0001596 |
| GO:0001822 |
| GO:0001921 |
| GO:0001965 |
| GO:0001991 |
| GO:0002001 |
| GO:0002018 |
| GO:0002019 |
| GO:0002035 |
| GO:0004945 |
| GO:0005515 |
| GO:0005737 |
| GO:0005768 |
| GO:0005794 |
| GO:0005886 |
| GO:0006885 |
| GO:0006954 |
| GO:0007186 |
| GO:0007200 |
| GO:0007204 |
| GO:0007507 |
| GO:0007568 |
| GO:0008284 |
| GO:0012505 |
| GO:0012506 |
| GO:0016020 |
| GO:0016021 |
| GO:0016323 |
| GO:0017046 |
| GO:0019901 |
| GO:0030425 |
| GO:0031410 |
| GO:0031968 |
| GO:0031983 |
| GO:0032930 |
| GO:0034391 |
| GO:0034392 |
| GO:0042310 |
| GO:0042416 |
| GO:0042756 |
| GO:0042976 |
| GO:0043621 |
| GO:0043627 |
| GO:0045777 |
| GO:0050715 |
| GO:0055037 |
| GO:0071549 |
| GO:0086097 |
| GO:0090190 |
|
| **UCSC Genome Browser** |
| uc007pyu.2 |
|
| **WikiGenes** |
| 11607 |
|
| **Affy** |
| 10404376 |
| 115838\_at |
| 1436739\_at |
| 166951\_at |
