# Supplementary material for: Automatically visualise and analyse data on pathways using PathVisioRPC from any programming environment
Source: BMC Bioinformatics. 2015 Aug 23;16(1):267. doi: 10.1186/s12859-015-0708-8 (PMC4546821; doi:10.1186/s12859-015-0708-8)
Supplement: Additional file 3: — Examples in Python. This zip archive contains the data and python script for the three python examples. (ZIP 15714 kb) [file 12859_2015_708_MOESM3_ESM.zip › Python_Examples/result_Example_1/geneList3/backpage/L_11610.html]

 

# geneproduct annotation

  

| Name: Agtrap| Identifier: 11610| Database: Entrez Gene| Synonyms: AT1R | | | --- | --- | | | | --- | --- | --- | --- | | | | --- | --- | --- | --- | --- | --- | | |
| --- | --- | --- | --- | --- | --- | --- | --- |

# Expression data

**Gene id on mapp: 11610**

| Sample name 11610| SystemCode L| LogFC 0.0| Pvalue 0.36757045| Type trans-PPS2 | | | --- | --- | | | | --- | --- | --- | --- | | | | --- | --- | --- | --- | --- | --- | | | | --- | --- | --- | --- | --- | --- | --- | --- | | |
| --- | --- | --- | --- | --- | --- | --- | --- | --- | --- |

  
  

---

  
  

# Cross references

  

|
|  |
| **UniGene** |
| Mm.444943 |
| Mm.46247 |
|
| **Agilent** |
| A\_51\_P271208 |
| A\_52\_P330667 |
| A\_52\_P480709 |
| A\_52\_P552550 |
|
| **Ensembl** |
| ENSMUSG00000029007 |
|
| **Illumina** |
| ILMN\_2590950 |
|
| **Entrez Gene** |
| 11610 |
|
| **MGI** |
| MGI:1339977 |
|
| **RefSeq** |
| NM\_009642 |
| NP\_033772 |
|
| **Uniprot/TrEMBL** |
| Q9WVK0 |
|
| **GeneOntology** |
| GO:0000139 |
| GO:0001666 |
| GO:0004945 |
| GO:0005515 |
| GO:0005789 |
| GO:0005886 |
| GO:0005938 |
| GO:0008217 |
| GO:0016021 |
| GO:0030659 |
| GO:0043231 |
|
| **UCSC Genome Browser** |
| uc008vtz.1 |
|
| **WikiGenes** |
| 11610 |
|
| **Affy** |
| 10518455 |
| 108417\_at |
| 130709\_at |
| 1422965\_at |
| 1460596\_at |
| 95355\_at |
| aa407794\_rc\_at |
