# Supplementary material for: Automatically visualise and analyse data on pathways using PathVisioRPC from any programming environment
Source: BMC Bioinformatics. 2015 Aug 23;16(1):267. doi: 10.1186/s12859-015-0708-8 (PMC4546821; doi:10.1186/s12859-015-0708-8)
Supplement: Additional file 3: — Examples in Python. This zip archive contains the data and python script for the three python examples. (ZIP 15714 kb) [file 12859_2015_708_MOESM3_ESM.zip › Python_Examples/result_Example_2/Statin Pathway/backpage/Ce_CHEBI_17002.html]

 

# Metabolite annotation

  

| Identifier: CHEBI:17002| Database: ChEBI | | | --- | --- | | |
| --- | --- | --- | --- |

# Expression data

**Gene id on mapp: CHEBI:17002**

| Sample name| SystemCode| LogFC| Pvalue| Type | | --- | | | --- | --- | | | --- | --- | --- | | | --- | --- | --- | --- | |
| --- | --- | --- | --- | --- |

  
  

---

  
  

# Cross references

  

|
|  |
| **ChEBI** |
| CHEBI:17002 |
