# Supplementary material for: Automatically visualise and analyse data on pathways using PathVisioRPC from any programming environment
Source: BMC Bioinformatics. 2015 Aug 23;16(1):267. doi: 10.1186/s12859-015-0708-8 (PMC4546821; doi:10.1186/s12859-015-0708-8)
Supplement: Additional file 3: — Examples in Python. This zip archive contains the data and python script for the three python examples. (ZIP 15714 kb) [file 12859_2015_708_MOESM3_ESM.zip › Python_Examples/result_Example_2/Statin Pathway/backpage/L_11806.html]

 

# GeneProduct annotation

  

| Name: Apoa1| Identifier: 11806| Database: Entrez Gene| Synonyms: Sep-1 | | | --- | --- | | | | --- | --- | --- | --- | | | | --- | --- | --- | --- | --- | --- | | |
| --- | --- | --- | --- | --- | --- | --- | --- |

# Expression data

**Gene id on mapp: 11806**

| Sample name| SystemCode| LogFC| Pvalue| Type | | --- | | | --- | --- | | | --- | --- | --- | | | --- | --- | --- | --- | |
| --- | --- | --- | --- | --- |

  
  

---

  
  

# Cross references

  

|
|  |
| **Agilent** |
| A\_51\_P408082 |
|
| **Ensembl** |
| ENSMUSG00000032083 |
|
| **Illumina** |
| ILMN\_2623393 |
|
| **Entrez Gene** |
| 11806 |
|
| **MGI** |
| MGI:88049 |
|
| **PDB** |
| 2LEM |
|
| **RefSeq** |
| NM\_009692 |
| NP\_033822 |
|
| **Uniprot/TrEMBL** |
| Q00623 |
|
| **GeneOntology** |
| GO:0001540 |
| GO:0001932 |
| GO:0001935 |
| GO:0002740 |
| GO:0005319 |
| GO:0005515 |
| GO:0005543 |
| GO:0005548 |
| GO:0005576 |
| GO:0005615 |
| GO:0005634 |
| GO:0006644 |
| GO:0006656 |
| GO:0006695 |
| GO:0007186 |
| GO:0007584 |
| GO:0008035 |
| GO:0008203 |
| GO:0008211 |
| GO:0008289 |
| GO:0010804 |
| GO:0010873 |
| GO:0010903 |
| GO:0014012 |
| GO:0015485 |
| GO:0015914 |
| GO:0017127 |
| GO:0018158 |
| GO:0018206 |
| GO:0019899 |
| GO:0019915 |
| GO:0030139 |
| GO:0030300 |
| GO:0030301 |
| GO:0030325 |
| GO:0031100 |
| GO:0031410 |
| GO:0032489 |
| GO:0033344 |
| GO:0033700 |
| GO:0034115 |
| GO:0034190 |
| GO:0034191 |
| GO:0034361 |
| GO:0034364 |
| GO:0034366 |
| GO:0034380 |
| GO:0042158 |
| GO:0042493 |
| GO:0042632 |
| GO:0042802 |
| GO:0043534 |
| GO:0043627 |
| GO:0043691 |
| GO:0050713 |
| GO:0050728 |
| GO:0050821 |
| GO:0051345 |
| GO:0051346 |
| GO:0051347 |
| GO:0055091 |
| GO:0055102 |
| GO:0060192 |
| GO:0060228 |
| GO:0060354 |
| GO:0060761 |
| GO:0070328 |
| GO:0070508 |
| GO:0070653 |
| GO:0071813 |
|
| **UCSC Genome Browser** |
| uc009phb.2 |
|
| **WikiGenes** |
| 11806 |
|
| **Affy** |
| 10585005 |
| 1419232\_a\_at |
| 1419233\_x\_at |
| 1438840\_x\_at |
| 1455201\_x\_at |
| 96094\_at |
| Msa.43196.0\_at |
