# Supplementary material for: Automatically visualise and analyse data on pathways using PathVisioRPC from any programming environment
Source: BMC Bioinformatics. 2015 Aug 23;16(1):267. doi: 10.1186/s12859-015-0708-8 (PMC4546821; doi:10.1186/s12859-015-0708-8)
Supplement: Additional file 3: — Examples in Python. This zip archive contains the data and python script for the three python examples. (ZIP 15714 kb) [file 12859_2015_708_MOESM3_ESM.zip › Python_Examples/result_Example_2/Statin Pathway/backpage/L_11808.html]

 

# GeneProduct annotation

  

| Name: Apoa4| Identifier: 11808| Database: Entrez Gene| Synonyms: Apoa-4 | | | --- | --- | | | | --- | --- | --- | --- | | | | --- | --- | --- | --- | --- | --- | | |
| --- | --- | --- | --- | --- | --- | --- | --- |

# Expression data

**Gene id on mapp: 11808**

| Sample name 11808 11808| SystemCode L L| LogFC 2.267971433 1.746109844| Pvalue 9.62633E-4 8.21793E-4| Type trans-PPS2 trans-PPS3 | | | | --- | --- | --- | | | | | --- | --- | --- | --- | --- | --- | | | | | --- | --- | --- | --- | --- | --- | --- | --- | --- | | | | | --- | --- | --- | --- | --- | --- | --- | --- | --- | --- | --- | --- | | | |
| --- | --- | --- | --- | --- | --- | --- | --- | --- | --- | --- | --- | --- | --- | --- |

  
  

---

  
  

# Cross references

  

|
|  |
| **Agilent** |
| A\_51\_P327491 |
|
| **Ensembl** |
| ENSMUSG00000032080 |
|
| **Illumina** |
| ILMN\_1250349 |
| ILMN\_2834123 |
|
| **Entrez Gene** |
| 11808 |
|
| **MGI** |
| MGI:88051 |
|
| **RefSeq** |
| NM\_007468 |
| NP\_031494 |
|
| **Uniprot/TrEMBL** |
| P06728 |
|
| **GeneOntology** |
| GO:0002227 |
| GO:0005507 |
| GO:0005615 |
| GO:0006869 |
| GO:0006982 |
| GO:0007159 |
| GO:0008203 |
| GO:0008289 |
| GO:0009986 |
| GO:0010873 |
| GO:0010898 |
| GO:0016209 |
| GO:0017127 |
| GO:0019430 |
| GO:0030300 |
| GO:0031210 |
| GO:0032374 |
| GO:0033344 |
| GO:0033700 |
| GO:0034361 |
| GO:0034364 |
| GO:0034372 |
| GO:0034445 |
| GO:0035634 |
| GO:0042157 |
| GO:0042627 |
| GO:0042632 |
| GO:0042744 |
| GO:0042803 |
| GO:0043499 |
| GO:0043691 |
| GO:0044240 |
| GO:0045723 |
| GO:0046470 |
| GO:0051006 |
| GO:0055088 |
| GO:0060228 |
| GO:0065005 |
|
| **UCSC Genome Browser** |
| uc009phd.1 |
|
| **WikiGenes** |
| 11808 |
|
| **Affy** |
| 100078\_at |
| 10585010 |
| 1417761\_at |
| 1436504\_x\_at |
| m13966\_f\_at |
