# Supplementary material for: Automatically visualise and analyse data on pathways using PathVisioRPC from any programming environment
Source: BMC Bioinformatics. 2015 Aug 23;16(1):267. doi: 10.1186/s12859-015-0708-8 (PMC4546821; doi:10.1186/s12859-015-0708-8)
Supplement: Additional file 3: — Examples in Python. This zip archive contains the data and python script for the three python examples. (ZIP 15714 kb) [file 12859_2015_708_MOESM3_ESM.zip › Python_Examples/result_Example_2/Statin Pathway/backpage/L_11812.html]

 

# GeneProduct annotation

  

| Name: Apoc1| Identifier: 11812| Database: Entrez Gene| Synonyms: ApoC-IB | | | --- | --- | | | | --- | --- | --- | --- | | | | --- | --- | --- | --- | --- | --- | | |
| --- | --- | --- | --- | --- | --- | --- | --- |

# Expression data

**Gene id on mapp: 11812**

| Sample name 11812 11812| SystemCode L L| LogFC 0.0 0.0| Pvalue 0.207131618 0.382712302| Type trans-PPS2 trans-PPS3 | | | | --- | --- | --- | | | | | --- | --- | --- | --- | --- | --- | | | | | --- | --- | --- | --- | --- | --- | --- | --- | --- | | | | | --- | --- | --- | --- | --- | --- | --- | --- | --- | --- | --- | --- | | | |
| --- | --- | --- | --- | --- | --- | --- | --- | --- | --- | --- | --- | --- | --- | --- |

  
  

---

  
  

# Cross references

  

|
|  |
| **Agilent** |
| A\_51\_P164504 |
| A\_55\_P1975370 |
|
| **Ensembl** |
| ENSMUSG00000040564 |
|
| **Illumina** |
| ILMN\_1228469 |
| ILMN\_2599794 |
|
| **Entrez Gene** |
| 11812 |
|
| **MGI** |
| MGI:88053 |
|
| **RefSeq** |
| NM\_001110009 |
| NM\_007469 |
| NP\_001103479 |
| NP\_031495 |
|
| **Uniprot/TrEMBL** |
| P34928 |
|
| **GeneOntology** |
| GO:0004859 |
| GO:0005504 |
| GO:0005783 |
| GO:0006641 |
| GO:0008203 |
| GO:0010900 |
| GO:0010916 |
| GO:0032375 |
| GO:0033344 |
| GO:0033700 |
| GO:0034361 |
| GO:0034364 |
| GO:0034369 |
| GO:0034382 |
| GO:0034447 |
| GO:0042157 |
| GO:0045717 |
| GO:0045833 |
| GO:0048261 |
| GO:0050995 |
| GO:0051005 |
| GO:0055102 |
|
| **UCSC Genome Browser** |
| uc009fmv.2 |
| uc009fmw.2 |
|
| **WikiGenes** |
| 11812 |
|
| **Affy** |
| 10560618 |
| 1417561\_at |
| 93354\_at |
| Msa.2160.0\_f\_at |
| Z22661\_f\_at |
