# Supplementary material for: Automatically visualise and analyse data on pathways using PathVisioRPC from any programming environment
Source: BMC Bioinformatics. 2015 Aug 23;16(1):267. doi: 10.1186/s12859-015-0708-8 (PMC4546821; doi:10.1186/s12859-015-0708-8)
Supplement: Additional file 3: — Examples in Python. This zip archive contains the data and python script for the three python examples. (ZIP 15714 kb) [file 12859_2015_708_MOESM3_ESM.zip › Python_Examples/result_Example_2/Statin Pathway/backpage/L_11813.html]

 

# GeneProduct annotation

  

| Name: Apoc2| Identifier: 11813| Database: Entrez Gene | | | --- | --- | | | | --- | --- | --- | --- | | |
| --- | --- | --- | --- | --- | --- |

# Expression data

**Gene id on mapp: 11813**

| Sample name 11813 11813| SystemCode L L| LogFC 0.0 1.202246384| Pvalue 0.162388785 0.006008236| Type trans-PPS2 trans-PPS3 | | | | --- | --- | --- | | | | | --- | --- | --- | --- | --- | --- | | | | | --- | --- | --- | --- | --- | --- | --- | --- | --- | | | | | --- | --- | --- | --- | --- | --- | --- | --- | --- | --- | --- | --- | | | |
| --- | --- | --- | --- | --- | --- | --- | --- | --- | --- | --- | --- | --- | --- | --- |

  
  

---

  
  

# Cross references

  

|
|  |
| **UniGene** |
| Mm.483990 |
|
| **Agilent** |
| A\_51\_P334979 |
|
| **Ensembl** |
| ENSMUSG00000002992 |
|
| **Illumina** |
| ILMN\_1234764 |
| ILMN\_2647820 |
| ILMN\_2880540 |
| ILMN\_3009225 |
|
| **Entrez Gene** |
| 11813 |
|
| **MGI** |
| MGI:88054 |
|
| **RefSeq** |
| NM\_009695 |
| NP\_033825 |
|
| **Uniprot/TrEMBL** |
| D3YXE8 |
| Q05020 |
| Q3UJG0 |
|
| **GeneOntology** |
| GO:0005615 |
| GO:0006629 |
| GO:0008289 |
| GO:0010518 |
| GO:0010898 |
| GO:0010916 |
| GO:0016004 |
| GO:0016042 |
| GO:0032375 |
| GO:0033344 |
| GO:0033700 |
| GO:0034361 |
| GO:0034362 |
| GO:0034363 |
| GO:0034366 |
| GO:0034382 |
| GO:0034384 |
| GO:0042493 |
| GO:0042627 |
| GO:0042803 |
| GO:0042953 |
| GO:0043274 |
| GO:0045723 |
| GO:0045833 |
| GO:0048261 |
| GO:0051006 |
| GO:0055102 |
| GO:0060230 |
| GO:0060697 |
| GO:0070328 |
|
| **UCSC Genome Browser** |
| uc009fmt.2 |
| uc012fbh.1 |
|
| **WikiGenes** |
| 11813 |
|
| **Affy** |
| 10560608 |
| 1418069\_at |
| 97887\_at |
| Msa.2161.0\_at |
| z15090\_f\_at |
