# Supplementary material for: Automatically visualise and analyse data on pathways using PathVisioRPC from any programming environment
Source: BMC Bioinformatics. 2015 Aug 23;16(1):267. doi: 10.1186/s12859-015-0708-8 (PMC4546821; doi:10.1186/s12859-015-0708-8)
Supplement: Additional file 3: — Examples in Python. This zip archive contains the data and python script for the three python examples. (ZIP 15714 kb) [file 12859_2015_708_MOESM3_ESM.zip › Python_Examples/result_Example_2/Statin Pathway/backpage/L_11814.html]

 

# GeneProduct annotation

  

| Name: Apoc3| Identifier: 11814| Database: Entrez Gene| Synonyms: Apoc | | | --- | --- | | | | --- | --- | --- | --- | | | | --- | --- | --- | --- | --- | --- | | |
| --- | --- | --- | --- | --- | --- | --- | --- |

# Expression data

**Gene id on mapp: 11814**

| Sample name| SystemCode| LogFC| Pvalue| Type | | --- | | | --- | --- | | | --- | --- | --- | | | --- | --- | --- | --- | |
| --- | --- | --- | --- | --- |

  
  

---

  
  

# Cross references

  

|
|  |
| **Agilent** |
| A\_51\_P310629 |
| A\_52\_P574306 |
| A\_55\_P2013710 |
| A\_55\_P2117155 |
|
| **Ensembl** |
| ENSMUSG00000032081 |
|
| **Illumina** |
| ILMN\_2651539 |
|
| **Entrez Gene** |
| 11814 |
|
| **MGI** |
| MGI:88055 |
|
| **RefSeq** |
| NM\_023114 |
| NP\_075603 |
|
| **Uniprot/TrEMBL** |
| D3YXN8 |
| E9QP56 |
| P33622 |
|
| **GeneOntology** |
| GO:0005543 |
| GO:0005576 |
| GO:0005615 |
| GO:0006641 |
| GO:0006642 |
| GO:0006869 |
| GO:0006954 |
| GO:0007186 |
| GO:0007584 |
| GO:0008203 |
| GO:0008289 |
| GO:0010897 |
| GO:0010903 |
| GO:0010916 |
| GO:0010987 |
| GO:0010989 |
| GO:0019433 |
| GO:0030234 |
| GO:0032489 |
| GO:0033344 |
| GO:0033700 |
| GO:0034361 |
| GO:0034363 |
| GO:0034366 |
| GO:0034375 |
| GO:0034382 |
| GO:0042157 |
| GO:0042493 |
| GO:0042627 |
| GO:0042632 |
| GO:0042953 |
| GO:0043434 |
| GO:0045717 |
| GO:0045833 |
| GO:0048261 |
| GO:0050995 |
| GO:0051005 |
| GO:0055102 |
| GO:0060621 |
| GO:0070328 |
| GO:0070653 |
|
| **UCSC Genome Browser** |
| uc009phc.1 |
|
| **WikiGenes** |
| 11814 |
|
| **Affy** |
| 10593169 |
| 1418278\_at |
| Msa.5103.0\_s\_at |
