# Supplementary material for: Automatically visualise and analyse data on pathways using PathVisioRPC from any programming environment
Source: BMC Bioinformatics. 2015 Aug 23;16(1):267. doi: 10.1186/s12859-015-0708-8 (PMC4546821; doi:10.1186/s12859-015-0708-8)
Supplement: Additional file 3: — Examples in Python. This zip archive contains the data and python script for the three python examples. (ZIP 15714 kb) [file 12859_2015_708_MOESM3_ESM.zip › Python_Examples/result_Example_2/Statin Pathway/backpage/L_11816.html]

 

# GeneProduct annotation

  

| Name: Apoe| Identifier: 11816| Database: Entrez Gene| Synonyms: AI255918 | | | --- | --- | | | | --- | --- | --- | --- | | | | --- | --- | --- | --- | --- | --- | | |
| --- | --- | --- | --- | --- | --- | --- | --- |

# Expression data

**Gene id on mapp: 11816**

| Sample name 11816 11816| SystemCode L L| LogFC 0.0 0.0| Pvalue 0.451373593 0.286656995| Type trans-PPS2 trans-PPS3 | | | | --- | --- | --- | | | | | --- | --- | --- | --- | --- | --- | | | | | --- | --- | --- | --- | --- | --- | --- | --- | --- | | | | | --- | --- | --- | --- | --- | --- | --- | --- | --- | --- | --- | --- | | | |
| --- | --- | --- | --- | --- | --- | --- | --- | --- | --- | --- | --- | --- | --- | --- |

  
  

---

  
  

# Cross references

  

|
|  |
| **UniGene** |
| Mm.468917 |
| Mm.475199 |
|
| **Agilent** |
| A\_51\_P171999 |
|
| **Ensembl** |
| ENSMUSG00000002985 |
|
| **Illumina** |
| ILMN\_1216042 |
|
| **Entrez Gene** |
| 11816 |
|
| **MGI** |
| MGI:88057 |
|
| **PDB** |
| 1YA9 |
|
| **RefSeq** |
| NM\_009696 |
| NP\_033826 |
|
| **Uniprot/TrEMBL** |
| E9Q327 |
| G3UWN5 |
| G3UWW2 |
| G3UZM8 |
| P08226 |
| Q3TXU4 |
| Q8C6E4 |
|
| **GeneOntology** |
| GO:0001540 |
| GO:0001937 |
| GO:0002021 |
| GO:0005319 |
| GO:0005515 |
| GO:0005543 |
| GO:0005615 |
| GO:0005737 |
| GO:0005770 |
| GO:0005794 |
| GO:0005886 |
| GO:0006629 |
| GO:0006641 |
| GO:0006707 |
| GO:0006869 |
| GO:0006874 |
| GO:0006898 |
| GO:0006917 |
| GO:0006979 |
| GO:0007186 |
| GO:0007263 |
| GO:0007568 |
| GO:0008201 |
| GO:0008203 |
| GO:0008289 |
| GO:0010468 |
| GO:0010544 |
| GO:0010873 |
| GO:0010875 |
| GO:0014012 |
| GO:0016209 |
| GO:0017127 |
| GO:0019934 |
| GO:0030195 |
| GO:0030425 |
| GO:0030828 |
| GO:0031232 |
| GO:0032489 |
| GO:0032526 |
| GO:0032805 |
| GO:0032868 |
| GO:0033344 |
| GO:0033700 |
| GO:0034361 |
| GO:0034362 |
| GO:0034363 |
| GO:0034364 |
| GO:0034372 |
| GO:0034374 |
| GO:0034375 |
| GO:0034380 |
| GO:0034382 |
| GO:0034384 |
| GO:0034447 |
| GO:0042157 |
| GO:0042158 |
| GO:0042159 |
| GO:0042311 |
| GO:0042627 |
| GO:0042632 |
| GO:0042802 |
| GO:0042803 |
| GO:0043025 |
| GO:0043066 |
| GO:0043407 |
| GO:0043524 |
| GO:0043537 |
| GO:0043691 |
| GO:0045471 |
| GO:0045541 |
| GO:0045773 |
| GO:0046848 |
| GO:0046911 |
| GO:0046982 |
| GO:0048156 |
| GO:0048709 |
| GO:0048844 |
| GO:0050728 |
| GO:0050750 |
| GO:0051000 |
| GO:0051044 |
| GO:0051651 |
| GO:0055088 |
| GO:0060228 |
| GO:0070326 |
| GO:0071347 |
| GO:0071363 |
| GO:0071397 |
| GO:0071813 |
| GO:0072358 |
|
| **UCSC Genome Browser** |
| uc009fmx.2 |
| uc009fmy.2 |
|
| **WikiGenes** |
| 11816 |
|
| **Affy** |
| 10560624 |
| 1432466\_a\_at |
| 95356\_at |
| Msa.18117.0\_f\_at |
| Msa.23293.0\_f\_at |
| Msa.4.0\_f\_at |
| d00466\_f\_at |
