# Supplementary material for: Automatically visualise and analyse data on pathways using PathVisioRPC from any programming environment
Source: BMC Bioinformatics. 2015 Aug 23;16(1):267. doi: 10.1186/s12859-015-0708-8 (PMC4546821; doi:10.1186/s12859-015-0708-8)
Supplement: Additional file 3: — Examples in Python. This zip archive contains the data and python script for the three python examples. (ZIP 15714 kb) [file 12859_2015_708_MOESM3_ESM.zip › Python_Examples/result_Example_2/Statin Pathway/backpage/L_13122.html]

 

# GeneProduct annotation

  

| Name: Cyp7a1| Identifier: 13122| Database: Entrez Gene | | | --- | --- | | | | --- | --- | --- | --- | | |
| --- | --- | --- | --- | --- | --- |

# Expression data

**Gene id on mapp: 13122**

| Sample name 13122 13122| SystemCode L L| LogFC 1.682955425 0.0| Pvalue 0.0014627 0.534974411| Type trans-PPS2 trans-PPS3 | | | | --- | --- | --- | | | | | --- | --- | --- | --- | --- | --- | | | | | --- | --- | --- | --- | --- | --- | --- | --- | --- | | | | | --- | --- | --- | --- | --- | --- | --- | --- | --- | --- | --- | --- | | | |
| --- | --- | --- | --- | --- | --- | --- | --- | --- | --- | --- | --- | --- | --- | --- |

  
  

---

  
  

# Cross references

  

|
|  |
| **UniGene** |
| Mm.57029 |
|
| **Agilent** |
| A\_51\_P290981 |
| A\_52\_P84027 |
|
| **Ensembl** |
| ENSMUSG00000028240 |
|
| **Illumina** |
| ILMN\_2604383 |
|
| **Entrez Gene** |
| 13122 |
|
| **MGI** |
| MGI:106091 |
|
| **RefSeq** |
| NM\_007824 |
| NP\_031850 |
|
| **Uniprot/TrEMBL** |
| Q64505 |
|
| **GeneOntology** |
| GO:0005506 |
| GO:0005789 |
| GO:0006699 |
| GO:0006707 |
| GO:0008123 |
| GO:0009055 |
| GO:0020037 |
| GO:0042632 |
| GO:0043231 |
| GO:0070857 |
| GO:0070859 |
| GO:0071333 |
| GO:0071397 |
|
| **UCSC Genome Browser** |
| uc008rxk.1 |
|
| **WikiGenes** |
| 13122 |
|
| **Affy** |
| 10511375 |
| 1422100\_at |
| 1438743\_at |
| 99404\_at |
| Msa.1555.0\_at |
