# Supplementary material for: Automatically visualise and analyse data on pathways using PathVisioRPC from any programming environment
Source: BMC Bioinformatics. 2015 Aug 23;16(1):267. doi: 10.1186/s12859-015-0708-8 (PMC4546821; doi:10.1186/s12859-015-0708-8)
Supplement: Additional file 3: — Examples in Python. This zip archive contains the data and python script for the three python examples. (ZIP 15714 kb) [file 12859_2015_708_MOESM3_ESM.zip › Python_Examples/result_Example_2/Statin Pathway/backpage/L_13350.html]

 

# GeneProduct annotation

  

| Name: Dgat1| Identifier: 13350| Database: Entrez Gene| Synonyms: C75990 | | | --- | --- | | | | --- | --- | --- | --- | | | | --- | --- | --- | --- | --- | --- | | |
| --- | --- | --- | --- | --- | --- | --- | --- |

# Expression data

**Gene id on mapp: 13350**

| Sample name| SystemCode| LogFC| Pvalue| Type | | --- | | | --- | --- | | | --- | --- | --- | | | --- | --- | --- | --- | |
| --- | --- | --- | --- | --- |

  
  

---

  
  

# Cross references

  

|
|  |
| **UniGene** |
| Mm.22633 |
|
| **Agilent** |
| A\_51\_P510059 |
| A\_52\_P456134 |
|
| **Ensembl** |
| ENSMUSG00000022555 |
|
| **Illumina** |
| ILMN\_1257757 |
| ILMN\_2807284 |
|
| **Entrez Gene** |
| 13350 |
|
| **MGI** |
| MGI:1333825 |
|
| **RefSeq** |
| NM\_010046 |
| NP\_034176 |
|
| **Uniprot/TrEMBL** |
| Q54AA6 |
| Q9Z2A7 |
|
| **GeneOntology** |
| GO:0003846 |
| GO:0004144 |
| GO:0005789 |
| GO:0016020 |
| GO:0016021 |
| GO:0019432 |
| GO:0019915 |
| GO:0034379 |
| GO:0035336 |
| GO:0043231 |
| GO:0046339 |
| GO:0050252 |
| GO:0055089 |
|
| **UCSC Genome Browser** |
| uc007wkn.1 |
|
| **WikiGenes** |
| 13350 |
|
| **Affy** |
| 10429926 |
| 104371\_at |
| 1418295\_s\_at |
| c76527\_rc\_at |
| c76527\_rc\_g\_at |
