# Supplementary material for: Automatically visualise and analyse data on pathways using PathVisioRPC from any programming environment
Source: BMC Bioinformatics. 2015 Aug 23;16(1):267. doi: 10.1186/s12859-015-0708-8 (PMC4546821; doi:10.1186/s12859-015-0708-8)
Supplement: Additional file 3: — Examples in Python. This zip archive contains the data and python script for the three python examples. (ZIP 15714 kb) [file 12859_2015_708_MOESM3_ESM.zip › Python_Examples/result_Example_2/Statin Pathway/backpage/L_15450.html]

 

# GeneProduct annotation

  

| Name: Lipc| Identifier: 15450| Database: Entrez Gene| Synonyms: AI256194 | | | --- | --- | | | | --- | --- | --- | --- | | | | --- | --- | --- | --- | --- | --- | | |
| --- | --- | --- | --- | --- | --- | --- | --- |

# Expression data

**Gene id on mapp: 15450**

| Sample name 15450 15450| SystemCode L L| LogFC -1.246531007 -1.173102564| Pvalue 0.001556383 0.002340734| Type trans-PPS2 trans-PPS3 | | | | --- | --- | --- | | | | | --- | --- | --- | --- | --- | --- | | | | | --- | --- | --- | --- | --- | --- | --- | --- | --- | | | | | --- | --- | --- | --- | --- | --- | --- | --- | --- | --- | --- | --- | | | |
| --- | --- | --- | --- | --- | --- | --- | --- | --- | --- | --- | --- | --- | --- | --- |

  
  

---

  
  

# Cross references

  

|
|  |
| **UniGene** |
| Mm.390187 |
|
| **Agilent** |
| A\_51\_P263993 |
|
| **Ensembl** |
| ENSMUSG00000032207 |
|
| **Illumina** |
| ILMN\_1257106 |
|
| **Entrez Gene** |
| 15450 |
|
| **MGI** |
| MGI:96216 |
|
| **RefSeq** |
| NM\_008280 |
| NP\_032306 |
|
| **Uniprot/TrEMBL** |
| P27656 |
| Q3TYU0 |
|
| **GeneOntology** |
| GO:0004806 |
| GO:0005515 |
| GO:0005615 |
| GO:0006633 |
| GO:0008201 |
| GO:0008203 |
| GO:0016298 |
| GO:0019433 |
| GO:0030301 |
| GO:0034364 |
| GO:0034372 |
| GO:0034374 |
| GO:0034375 |
| GO:0042632 |
| GO:0050253 |
| GO:0070328 |
|
| **UCSC Genome Browser** |
| uc009qos.1 |
|
| **WikiGenes** |
| 15450 |
|
| **Affy** |
| 10594812 |
| 1419560\_at |
| 98962\_at |
| X58426\_s\_at |
