# Supplementary material for: Automatically visualise and analyse data on pathways using PathVisioRPC from any programming environment
Source: BMC Bioinformatics. 2015 Aug 23;16(1):267. doi: 10.1186/s12859-015-0708-8 (PMC4546821; doi:10.1186/s12859-015-0708-8)
Supplement: Additional file 3: — Examples in Python. This zip archive contains the data and python script for the three python examples. (ZIP 15714 kb) [file 12859_2015_708_MOESM3_ESM.zip › Python_Examples/result_Example_2/Statin Pathway/backpage/L_16816.html]

 

# GeneProduct annotation

  

| Name: Lcat| Identifier: 16816| Database: Entrez Gene| Synonyms: D8Wsu61e | | | --- | --- | | | | --- | --- | --- | --- | | | | --- | --- | --- | --- | --- | --- | | |
| --- | --- | --- | --- | --- | --- | --- | --- |

# Expression data

**Gene id on mapp: 16816**

| Sample name 16816 16816| SystemCode L L| LogFC -1.159583403 0.0| Pvalue 0.014276683 0.179573305| Type trans-PPS2 trans-PPS3 | | | | --- | --- | --- | | | | | --- | --- | --- | --- | --- | --- | | | | | --- | --- | --- | --- | --- | --- | --- | --- | --- | | | | | --- | --- | --- | --- | --- | --- | --- | --- | --- | --- | --- | --- | | | |
| --- | --- | --- | --- | --- | --- | --- | --- | --- | --- | --- | --- | --- | --- | --- |

  
  

---

  
  

# Cross references

  

|
|  |
| **UniGene** |
| Mm.1593 |
| Mm.467489 |
|
| **Agilent** |
| A\_51\_P336060 |
|
| **Ensembl** |
| ENSMUSG00000035237 |
|
| **Illumina** |
| ILMN\_2627961 |
| ILMN\_2798400 |
| ILMN\_2798402 |
|
| **Entrez Gene** |
| 16816 |
|
| **MGI** |
| MGI:96755 |
|
| **RefSeq** |
| NM\_008490 |
| NP\_032516 |
|
| **Uniprot/TrEMBL** |
| P16301 |
|
| **GeneOntology** |
| GO:0004607 |
| GO:0005515 |
| GO:0005615 |
| GO:0006644 |
| GO:0006656 |
| GO:0008203 |
| GO:0030301 |
| GO:0034186 |
| GO:0034364 |
| GO:0034372 |
| GO:0034375 |
| GO:0034435 |
| GO:0042158 |
| GO:0042632 |
| GO:0043691 |
| GO:0090107 |
|
| **UCSC Genome Browser** |
| uc009neq.2 |
|
| **WikiGenes** |
| 16816 |
|
| **Affy** |
| 103023\_at |
| 10581388 |
| 1417043\_at |
| J05154\_s\_at |
