# Supplementary material for: Automatically visualise and analyse data on pathways using PathVisioRPC from any programming environment
Source: BMC Bioinformatics. 2015 Aug 23;16(1):267. doi: 10.1186/s12859-015-0708-8 (PMC4546821; doi:10.1186/s12859-015-0708-8)
Supplement: Additional file 3: — Examples in Python. This zip archive contains the data and python script for the three python examples. (ZIP 15714 kb) [file 12859_2015_708_MOESM3_ESM.zip › Python_Examples/result_Example_2/Statin Pathway/backpage/L_16835.html]

 

# GeneProduct annotation

  

| Name: Ldlr| Identifier: 16835| Database: Entrez Gene| Synonyms: Hlb301 | | | --- | --- | | | | --- | --- | --- | --- | | | | --- | --- | --- | --- | --- | --- | | |
| --- | --- | --- | --- | --- | --- | --- | --- |

# Expression data

**Gene id on mapp: 16835**

| Sample name 16835 16835| SystemCode L L| LogFC 1.265629607 1.239302673| Pvalue 0.017721518 0.005415808| Type trans-PPS2 trans-PPS3 | | | | --- | --- | --- | | | | | --- | --- | --- | --- | --- | --- | | | | | --- | --- | --- | --- | --- | --- | --- | --- | --- | | | | | --- | --- | --- | --- | --- | --- | --- | --- | --- | --- | --- | --- | | | |
| --- | --- | --- | --- | --- | --- | --- | --- | --- | --- | --- | --- | --- | --- | --- |

  
  

---

  
  

# Cross references

  

|
|  |
| **UniGene** |
| Mm.3213 |
| Mm.392278 |
| Mm.469604 |
|
| **Agilent** |
| A\_52\_P427024 |
| A\_55\_P2167999 |
|
| **Ensembl** |
| ENSMUSG00000032193 |
|
| **Illumina** |
| ILMN\_1221255 |
| ILMN\_2461018 |
|
| **Entrez Gene** |
| 16835 |
|
| **MGI** |
| MGI:96765 |
|
| **RefSeq** |
| NM\_001252658 |
| NM\_001252659 |
| NM\_010700 |
| NP\_001239587 |
| NP\_001239588 |
| NP\_034830 |
|
| **Uniprot/TrEMBL** |
| P35951 |
|
| **GeneOntology** |
| GO:0005041 |
| GO:0005509 |
| GO:0005515 |
| GO:0005764 |
| GO:0005768 |
| GO:0005769 |
| GO:0005770 |
| GO:0005794 |
| GO:0005886 |
| GO:0005905 |
| GO:0006629 |
| GO:0008203 |
| GO:0009986 |
| GO:0010867 |
| GO:0010899 |
| GO:0015914 |
| GO:0016021 |
| GO:0030169 |
| GO:0030229 |
| GO:0030301 |
| GO:0034362 |
| GO:0034383 |
| GO:0042157 |
| GO:0042159 |
| GO:0042632 |
| GO:0070508 |
|
| **UCSC Genome Browser** |
| uc009omi.2 |
|
| **WikiGenes** |
| 16835 |
|
| **Affy** |
| 10583732 |
| 1421821\_at |
| 1450383\_at |
| 160832\_at |
| x64414\_s\_at |
