# Supplementary material for: Automatically visualise and analyse data on pathways using PathVisioRPC from any programming environment
Source: BMC Bioinformatics. 2015 Aug 23;16(1):267. doi: 10.1186/s12859-015-0708-8 (PMC4546821; doi:10.1186/s12859-015-0708-8)
Supplement: Additional file 3: — Examples in Python. This zip archive contains the data and python script for the three python examples. (ZIP 15714 kb) [file 12859_2015_708_MOESM3_ESM.zip › Python_Examples/result_Example_2/Statin Pathway/backpage/L_16956.html]

 

# GeneProduct annotation

  

| Name: Lpl| Identifier: 16956| Database: Entrez Gene | | | --- | --- | | | | --- | --- | --- | --- | | |
| --- | --- | --- | --- | --- | --- |

# Expression data

**Gene id on mapp: 16956**

| Sample name 16956 16956| SystemCode L L| LogFC -1.789399818 -1.916626283| Pvalue 0.002601098 1.61E-9| Type trans-PPS2 trans-PPS3 | | | | --- | --- | --- | | | | | --- | --- | --- | --- | --- | --- | | | | | --- | --- | --- | --- | --- | --- | --- | --- | --- | | | | | --- | --- | --- | --- | --- | --- | --- | --- | --- | --- | --- | --- | | | |
| --- | --- | --- | --- | --- | --- | --- | --- | --- | --- | --- | --- | --- | --- | --- |

  
  

---

  
  

# Cross references

  

|
|  |
| **UniGene** |
| Mm.1514 |
| Mm.401817 |
| Mm.418638 |
| Mm.470568 |
|
| **Agilent** |
| A\_51\_P259296 |
| A\_52\_P257812 |
|
| **Ensembl** |
| ENSMUSG00000015568 |
|
| **Illumina** |
| ILMN\_1246258 |
| ILMN\_2585447 |
| ILMN\_2692723 |
| ILMN\_2770017 |
|
| **Entrez Gene** |
| 16956 |
|
| **MGI** |
| MGI:96820 |
|
| **RefSeq** |
| NM\_008509 |
| NP\_032535 |
|
| **Uniprot/TrEMBL** |
| P11152 |
| Q3UAX2 |
|
| **GeneOntology** |
| GO:0004465 |
| GO:0004806 |
| GO:0005102 |
| GO:0005515 |
| GO:0005615 |
| GO:0005886 |
| GO:0008201 |
| GO:0009986 |
| GO:0010744 |
| GO:0010886 |
| GO:0010890 |
| GO:0016042 |
| GO:0017129 |
| GO:0019432 |
| GO:0019433 |
| GO:0031012 |
| GO:0031225 |
| GO:0034185 |
| GO:0034361 |
| GO:0042493 |
| GO:0042627 |
| GO:0070328 |
|
| **UCSC Genome Browser** |
| uc009lwq.1 |
|
| **WikiGenes** |
| 16956 |
|
| **Affy** |
| 10572130 |
| 1415904\_at |
| 1431056\_a\_at |
| 160083\_at |
| 95611\_at |
| aa683731\_s\_at |
