# Supplementary material for: Automatically visualise and analyse data on pathways using PathVisioRPC from any programming environment
Source: BMC Bioinformatics. 2015 Aug 23;16(1):267. doi: 10.1186/s12859-015-0708-8 (PMC4546821; doi:10.1186/s12859-015-0708-8)
Supplement: Additional file 3: — Examples in Python. This zip archive contains the data and python script for the three python examples. (ZIP 15714 kb) [file 12859_2015_708_MOESM3_ESM.zip › Python_Examples/result_Example_2/Statin Pathway/backpage/L_16971.html]

 

# GeneProduct annotation

  

| Name: Lrp1| Identifier: 16971| Database: Entrez Gene| Synonyms: A2mr | | | --- | --- | | | | --- | --- | --- | --- | | | | --- | --- | --- | --- | --- | --- | | |
| --- | --- | --- | --- | --- | --- | --- | --- |

# Expression data

**Gene id on mapp: 16971**

| Sample name 16971 16971| SystemCode L L| LogFC 1.147146607 -1.112658865| Pvalue 0.034586353 0.012462337| Type trans-PPS2 trans-PPS3 | | | | --- | --- | --- | | | | | --- | --- | --- | --- | --- | --- | | | | | --- | --- | --- | --- | --- | --- | --- | --- | --- | | | | | --- | --- | --- | --- | --- | --- | --- | --- | --- | --- | --- | --- | | | |
| --- | --- | --- | --- | --- | --- | --- | --- | --- | --- | --- | --- | --- | --- | --- |

  
  

---

  
  

# Cross references

  

|
|  |
| **UniGene** |
| Mm.271854 |
| Mm.395155 |
|
| **Agilent** |
| A\_51\_P193794 |
| A\_52\_P202045 |
| A\_55\_P1985628 |
| A\_55\_P1985633 |
|
| **Ensembl** |
| ENSMUSG00000040249 |
|
| **Illumina** |
| ILMN\_1237723 |
|
| **Entrez Gene** |
| 16971 |
|
| **MGI** |
| MGI:96828 |
|
| **RefSeq** |
| NM\_008512 |
| NP\_032538 |
|
| **Uniprot/TrEMBL** |
| D3Z5M3 |
| Q3U454 |
| Q3U5J2 |
| Q811K6 |
| Q8C4T5 |
| Q91ZX7 |
|
| **GeneOntology** |
| GO:0005509 |
| GO:0005515 |
| GO:0005634 |
| GO:0005887 |
| GO:0005905 |
| GO:0006898 |
| GO:0008203 |
| GO:0010875 |
| GO:0014912 |
| GO:0016020 |
| GO:0016021 |
| GO:0030178 |
| GO:0032370 |
| GO:0032374 |
| GO:0032403 |
| GO:0032429 |
| GO:0032956 |
| GO:0034185 |
| GO:0035909 |
| GO:0043277 |
| GO:0048471 |
| GO:0097242 |
| GO:2000587 |
|
| **UCSC Genome Browser** |
| uc007hjx.1 |
| uc007hjz.1 |
|
| **WikiGenes** |
| 16971 |
|
| **Affy** |
| 101073\_at |
| 10373223 |
| 1442849\_at |
| 1448655\_at |
| X67469\_s\_at |
