# Supplementary material for: Automatically visualise and analyse data on pathways using PathVisioRPC from any programming environment
Source: BMC Bioinformatics. 2015 Aug 23;16(1):267. doi: 10.1186/s12859-015-0708-8 (PMC4546821; doi:10.1186/s12859-015-0708-8)
Supplement: Additional file 3: — Examples in Python. This zip archive contains the data and python script for the three python examples. (ZIP 15714 kb) [file 12859_2015_708_MOESM3_ESM.zip › Python_Examples/result_Example_2/Statin Pathway/backpage/L_17777.html]

 

# GeneProduct annotation

  

| Name: Mttp| Identifier: 17777| Database: Entrez Gene| Synonyms: 1810043K16Rik | | | --- | --- | | | | --- | --- | --- | --- | | | | --- | --- | --- | --- | --- | --- | | |
| --- | --- | --- | --- | --- | --- | --- | --- |

# Expression data

**Gene id on mapp: 17777**

| Sample name| SystemCode| LogFC| Pvalue| Type | | --- | | | --- | --- | | | --- | --- | --- | | | --- | --- | --- | --- | |
| --- | --- | --- | --- | --- |

  
  

---

  
  

# Cross references

  

|
|  |
| **UniGene** |
| Mm.2941 |
| Mm.470777 |
|
| **Agilent** |
| A\_51\_P178887 |
| A\_55\_P1970299 |
| A\_55\_P2344933 |
|
| **Ensembl** |
| ENSMUSG00000028158 |
|
| **Illumina** |
| ILMN\_2628594 |
|
| **Entrez Gene** |
| 17777 |
|
| **MGI** |
| MGI:106926 |
|
| **RefSeq** |
| NM\_001163457 |
| NM\_008642 |
| NP\_001156929 |
| NP\_032668 |
|
| **Uniprot/TrEMBL** |
| O08601 |
| Q3TVF2 |
| Q3UWK4 |
|
| **GeneOntology** |
| GO:0005319 |
| GO:0005515 |
| GO:0005783 |
| GO:0005791 |
| GO:0005794 |
| GO:0006497 |
| GO:0006629 |
| GO:0006641 |
| GO:0006869 |
| GO:0008203 |
| GO:0008289 |
| GO:0016323 |
| GO:0031526 |
| GO:0031528 |
| GO:0031988 |
| GO:0034185 |
| GO:0042157 |
| GO:0042632 |
| GO:0042953 |
| GO:0046982 |
| GO:0051592 |
|
| **UCSC Genome Browser** |
| uc008rmw.2 |
| uc008rmx.2 |
|
| **WikiGenes** |
| 17777 |
|
| **Affy** |
| 104448\_at |
| 10502375 |
| 110233\_at |
| 1419399\_at |
| 1419400\_at |
| 1443951\_at |
| L47970\_s\_at |
