# Supplementary material for: Automatically visualise and analyse data on pathways using PathVisioRPC from any programming environment
Source: BMC Bioinformatics. 2015 Aug 23;16(1):267. doi: 10.1186/s12859-015-0708-8 (PMC4546821; doi:10.1186/s12859-015-0708-8)
Supplement: Additional file 3: — Examples in Python. This zip archive contains the data and python script for the three python examples. (ZIP 15714 kb) [file 12859_2015_708_MOESM3_ESM.zip › Python_Examples/result_Example_2/Statin Pathway/backpage/L_18830.html]

 

# GeneProduct annotation

  

| Name: Pltp| Identifier: 18830| Database: Entrez Gene| Synonyms: Bpife | | | --- | --- | | | | --- | --- | --- | --- | | | | --- | --- | --- | --- | --- | --- | | |
| --- | --- | --- | --- | --- | --- | --- | --- |

# Expression data

**Gene id on mapp: 18830**

| Sample name 18830 18830| SystemCode L L| LogFC 0.0 1.767761171| Pvalue 0.776135998 6.6276E-4| Type trans-PPS2 trans-PPS3 | | | | --- | --- | --- | | | | | --- | --- | --- | --- | --- | --- | | | | | --- | --- | --- | --- | --- | --- | --- | --- | --- | | | | | --- | --- | --- | --- | --- | --- | --- | --- | --- | --- | --- | --- | | | |
| --- | --- | --- | --- | --- | --- | --- | --- | --- | --- | --- | --- | --- | --- | --- |

  
  

---

  
  

# Cross references

  

|
|  |
| **UniGene** |
| Mm.6105 |
|
| **Agilent** |
| A\_51\_P226655 |
| A\_55\_P1993955 |
|
| **Ensembl** |
| ENSMUSG00000017754 |
|
| **Illumina** |
| ILMN\_2589401 |
| ILMN\_2663014 |
| ILMN\_2669714 |
|
| **Entrez Gene** |
| 18830 |
|
| **MGI** |
| MGI:103151 |
|
| **RefSeq** |
| NM\_011125 |
| NP\_035255 |
|
| **Uniprot/TrEMBL** |
| A2A5K2 |
| A2A5K3 |
| A2A5K4 |
| P55065 |
| Q3UE59 |
| Q3UFS5 |
|
| **GeneOntology** |
| GO:0005576 |
| GO:0006869 |
| GO:0008289 |
| GO:0010189 |
| GO:0030317 |
|
| **UCSC Genome Browser** |
| uc008nwn.1 |
|
| **WikiGenes** |
| 18830 |
|
| **Affy** |
| 100927\_at |
| 10489569 |
| 1417963\_at |
| 1456424\_s\_at |
| u37226\_s\_at |
