# Supplementary material for: Automatically visualise and analyse data on pathways using PathVisioRPC from any programming environment
Source: BMC Bioinformatics. 2015 Aug 23;16(1):267. doi: 10.1186/s12859-015-0708-8 (PMC4546821; doi:10.1186/s12859-015-0708-8)
Supplement: Additional file 3: — Examples in Python. This zip archive contains the data and python script for the three python examples. (ZIP 15714 kb) [file 12859_2015_708_MOESM3_ESM.zip › Python_Examples/result_Example_2/Statin Pathway/backpage/L_20652.html]

 

# GeneProduct annotation

  

| Name: Soat1| Identifier: 20652| Database: Entrez Gene| Synonyms: Acact | | | --- | --- | | | | --- | --- | --- | --- | | | | --- | --- | --- | --- | --- | --- | | |
| --- | --- | --- | --- | --- | --- | --- | --- |

# Expression data

**Gene id on mapp: 20652**

| Sample name 20652| SystemCode L| LogFC 0.0| Pvalue 0.833427464| Type trans-PPS2 | | | --- | --- | | | | --- | --- | --- | --- | | | | --- | --- | --- | --- | --- | --- | | | | --- | --- | --- | --- | --- | --- | --- | --- | | |
| --- | --- | --- | --- | --- | --- | --- | --- | --- | --- |

  
  

---

  
  

# Cross references

  

|
|  |
| **UniGene** |
| Mm.28099 |
| Mm.402249 |
| Mm.488792 |
|
| **Agilent** |
| A\_51\_P391754 |
| A\_52\_P642167 |
| A\_52\_P651833 |
|
| **Ensembl** |
| ENSMUSG00000026600 |
|
| **Illumina** |
| ILMN\_1248510 |
| ILMN\_2419998 |
|
| **Entrez Gene** |
| 20652 |
|
| **MGI** |
| MGI:104665 |
|
| **RefSeq** |
| NM\_009230 |
| NP\_033256 |
|
| **Uniprot/TrEMBL** |
| Q61263 |
|
| **GeneOntology** |
| GO:0000062 |
| GO:0004772 |
| GO:0005783 |
| GO:0005789 |
| GO:0008203 |
| GO:0010742 |
| GO:0010878 |
| GO:0015485 |
| GO:0016020 |
| GO:0016021 |
| GO:0033344 |
| GO:0034379 |
| GO:0034435 |
| GO:0034736 |
| GO:0042986 |
|
| **UCSC Genome Browser** |
| uc007dcj.1 |
|
| **WikiGenes** |
| 20652 |
|
| **Affy** |
| 10359161 |
| 1417695\_a\_at |
| 1417696\_at |
| 1417697\_at |
| 1448068\_at |
| 160767\_at |
| 95887\_at |
| AA177204\_at |
| L42293\_s\_at |
