# Supplementary material for: Automatically visualise and analyse data on pathways using PathVisioRPC from any programming environment
Source: BMC Bioinformatics. 2015 Aug 23;16(1):267. doi: 10.1186/s12859-015-0708-8 (PMC4546821; doi:10.1186/s12859-015-0708-8)
Supplement: Additional file 3: — Examples in Python. This zip archive contains the data and python script for the three python examples. (ZIP 15714 kb) [file 12859_2015_708_MOESM3_ESM.zip › Python_Examples/result_Example_2/Statin Pathway/backpage/L_20778.html]

 

# GeneProduct annotation

  

| Name: Scarb1| Identifier: 20778| Database: Entrez Gene| Synonyms: AI120173 | | | --- | --- | | | | --- | --- | --- | --- | | | | --- | --- | --- | --- | --- | --- | | |
| --- | --- | --- | --- | --- | --- | --- | --- |

# Expression data

**Gene id on mapp: 20778**

| Sample name 20778 20778| SystemCode L L| LogFC 0.0 -1.219419317| Pvalue 0.131228895 7.51E-5| Type trans-PPS2 trans-PPS3 | | | | --- | --- | --- | | | | | --- | --- | --- | --- | --- | --- | | | | | --- | --- | --- | --- | --- | --- | --- | --- | --- | | | | | --- | --- | --- | --- | --- | --- | --- | --- | --- | --- | --- | --- | | | |
| --- | --- | --- | --- | --- | --- | --- | --- | --- | --- | --- | --- | --- | --- | --- |

  
  

---

  
  

# Cross references

  

|
|  |
| **UniGene** |
| Mm.282242 |
| Mm.474018 |
|
| **Agilent** |
| A\_51\_P255817 |
| A\_55\_P1954021 |
| A\_55\_P2088965 |
|
| **Ensembl** |
| ENSMUSG00000037936 |
|
| **Illumina** |
| ILMN\_1218449 |
| ILMN\_1223315 |
| ILMN\_2578984 |
| ILMN\_2636249 |
|
| **Entrez Gene** |
| 20778 |
|
| **MGI** |
| MGI:893578 |
|
| **RefSeq** |
| NM\_001205082 |
| NM\_001205083 |
| NM\_016741 |
| NP\_001192011 |
| NP\_001192012 |
| NP\_058021 |
|
| **Uniprot/TrEMBL** |
| D3Z2V4 |
| D3Z5U8 |
| F7C5U2 |
| Q4FK30 |
| Q61009 |
|
| **GeneOntology** |
| GO:0001530 |
| GO:0001875 |
| GO:0001935 |
| GO:0005737 |
| GO:0005886 |
| GO:0005887 |
| GO:0005901 |
| GO:0006702 |
| GO:0006707 |
| GO:0006869 |
| GO:0006910 |
| GO:0007155 |
| GO:0008035 |
| GO:0009986 |
| GO:0010867 |
| GO:0010886 |
| GO:0010899 |
| GO:0015914 |
| GO:0015920 |
| GO:0016021 |
| GO:0030169 |
| GO:0030301 |
| GO:0031528 |
| GO:0031663 |
| GO:0032497 |
| GO:0033344 |
| GO:0034185 |
| GO:0034186 |
| GO:0034375 |
| GO:0034383 |
| GO:0034384 |
| GO:0042632 |
| GO:0042803 |
| GO:0043534 |
| GO:0043654 |
| GO:0043691 |
| GO:0051000 |
| GO:0051856 |
| GO:0070328 |
| GO:0070506 |
| GO:0070508 |
|
| **UCSC Genome Browser** |
| uc008zrd.2 |
| uc008zre.2 |
|
| **WikiGenes** |
| 20778 |
|
| **Affy** |
| 100095\_at |
| 10533929 |
| 1416050\_a\_at |
| 1437378\_x\_at |
| 1455820\_x\_at |
| U37799\_s\_at |
