# Supplementary material for: Automatically visualise and analyse data on pathways using PathVisioRPC from any programming environment
Source: BMC Bioinformatics. 2015 Aug 23;16(1):267. doi: 10.1186/s12859-015-0708-8 (PMC4546821; doi:10.1186/s12859-015-0708-8)
Supplement: Additional file 3: — Examples in Python. This zip archive contains the data and python script for the three python examples. (ZIP 15714 kb) [file 12859_2015_708_MOESM3_ESM.zip › Python_Examples/result_Example_2/geneList/backpage/L_11303.html]

 

# geneproduct annotation

  

| Name: Abca1| Identifier: 11303| Database: Entrez Gene| Synonyms: Abc1 | | | --- | --- | | | | --- | --- | --- | --- | | | | --- | --- | --- | --- | --- | --- | | |
| --- | --- | --- | --- | --- | --- | --- | --- |

# Expression data

**Gene id on mapp: 11303**

| Sample name 11303 11303| SystemCode L L| LogFC -2.994704896 0.0| Pvalue 4.06181E-4 0.056745584| Type trans-PPS2 trans-PPS3 | | | | --- | --- | --- | | | | | --- | --- | --- | --- | --- | --- | | | | | --- | --- | --- | --- | --- | --- | --- | --- | --- | | | | | --- | --- | --- | --- | --- | --- | --- | --- | --- | --- | --- | --- | | | |
| --- | --- | --- | --- | --- | --- | --- | --- | --- | --- | --- | --- | --- | --- | --- |

  
  

---

  
  

# Cross references

  

|
|  |
| **UniGene** |
| Mm.277376 |
|
| **Agilent** |
| A\_52\_P665675 |
|
| **Ensembl** |
| ENSMUSG00000015243 |
|
| **Illumina** |
| ILMN\_1226851 |
|
| **Entrez Gene** |
| 11303 |
|
| **MGI** |
| MGI:99607 |
|
| **RefSeq** |
| NM\_013454 |
| NP\_038482 |
|
| **Uniprot/TrEMBL** |
| P41233 |
|
| **GeneOntology** |
| GO:0002790 |
| GO:0005515 |
| GO:0005524 |
| GO:0005548 |
| GO:0005794 |
| GO:0005886 |
| GO:0005887 |
| GO:0006497 |
| GO:0006820 |
| GO:0006911 |
| GO:0007040 |
| GO:0007186 |
| GO:0008203 |
| GO:0008509 |
| GO:0010875 |
| GO:0016197 |
| GO:0016887 |
| GO:0017127 |
| GO:0019905 |
| GO:0030139 |
| GO:0030819 |
| GO:0031267 |
| GO:0032367 |
| GO:0032489 |
| GO:0033344 |
| GO:0033700 |
| GO:0034185 |
| GO:0034186 |
| GO:0034188 |
| GO:0034380 |
| GO:0034616 |
| GO:0038027 |
| GO:0042157 |
| GO:0042158 |
| GO:0042632 |
| GO:0043231 |
| GO:0043691 |
| GO:0045121 |
| GO:0045332 |
| GO:0045335 |
| GO:0050702 |
| GO:0055091 |
| GO:0055098 |
| GO:0060155 |
| GO:0071222 |
| GO:0071300 |
|
| **UCSC Genome Browser** |
| uc008swu.1 |
|
| **WikiGenes** |
| 11303 |
|
| **Affy** |
| 10512949 |
| 1421839\_at |
| 1421840\_at |
| 1450392\_at |
| 94354\_at |
| 97198\_at |
| X75926\_s\_at |
| aa690738\_s\_at |
