# Supplementary material for: Automatically visualise and analyse data on pathways using PathVisioRPC from any programming environment
Source: BMC Bioinformatics. 2015 Aug 23;16(1):267. doi: 10.1186/s12859-015-0708-8 (PMC4546821; doi:10.1186/s12859-015-0708-8)
Supplement: Additional file 3: — Examples in Python. This zip archive contains the data and python script for the three python examples. (ZIP 15714 kb) [file 12859_2015_708_MOESM3_ESM.zip › Python_Examples/result_Example_2/geneList/backpage/L_11305.html]

 

# geneproduct annotation

  

| Name: Abca2| Identifier: 11305| Database: Entrez Gene| Synonyms: AI413825 | | | --- | --- | | | | --- | --- | --- | --- | | | | --- | --- | --- | --- | --- | --- | | |
| --- | --- | --- | --- | --- | --- | --- | --- |

# Expression data

**Gene id on mapp: 11305**

| Sample name 11305 11305| SystemCode L L| LogFC -1.241134469 0.0| Pvalue 0.01111258 0.128070858| Type trans-PPS2 trans-PPS3 | | | | --- | --- | --- | | | | | --- | --- | --- | --- | --- | --- | | | | | --- | --- | --- | --- | --- | --- | --- | --- | --- | | | | | --- | --- | --- | --- | --- | --- | --- | --- | --- | --- | --- | --- | | | |
| --- | --- | --- | --- | --- | --- | --- | --- | --- | --- | --- | --- | --- | --- | --- |

  
  

---

  
  

# Cross references

  

|
|  |
| **UniGene** |
| Mm.2210 |
| Mm.418609 |
|
| **Agilent** |
| A\_51\_P246844 |
| A\_52\_P485007 |
| A\_55\_P2021177 |
|
| **Ensembl** |
| ENSMUSG00000026944 |
|
| **Illumina** |
| ILMN\_1216987 |
|
| **Entrez Gene** |
| 11305 |
|
| **MGI** |
| MGI:99606 |
|
| **RefSeq** |
| NM\_007379 |
| NP\_031405 |
|
| **Uniprot/TrEMBL** |
| A2AJ26 |
| Q3TMR1 |
| Q3TZM0 |
| Q6ZPZ4 |
|
| **GeneOntology** |
| GO:0005524 |
| GO:0005764 |
| GO:0005768 |
| GO:0006200 |
| GO:0006357 |
| GO:0016021 |
| GO:0016887 |
| GO:0032383 |
| GO:0042632 |
| GO:0048545 |
|
| **UCSC Genome Browser** |
| uc008isc.1 |
|
| **WikiGenes** |
| 11305 |
|
| **Affy** |
| 104137\_at |
| 10470050 |
| 1449302\_at |
| X75927\_s\_at |
