# Supplementary material for: Automatically visualise and analyse data on pathways using PathVisioRPC from any programming environment
Source: BMC Bioinformatics. 2015 Aug 23;16(1):267. doi: 10.1186/s12859-015-0708-8 (PMC4546821; doi:10.1186/s12859-015-0708-8)
Supplement: Additional file 3: — Examples in Python. This zip archive contains the data and python script for the three python examples. (ZIP 15714 kb) [file 12859_2015_708_MOESM3_ESM.zip › Python_Examples/result_Example_2/geneList/backpage/L_11307.html]

 

# geneproduct annotation

  

| Name: Abcg1| Identifier: 11307| Database: Entrez Gene| Synonyms: White | | | --- | --- | | | | --- | --- | --- | --- | | | | --- | --- | --- | --- | --- | --- | | |
| --- | --- | --- | --- | --- | --- | --- | --- |

# Expression data

**Gene id on mapp: 11307**

| Sample name 11307 11307| SystemCode L L| LogFC 0.0 0.0| Pvalue 0.268886186 0.702274138| Type trans-PPS2 trans-PPS3 | | | | --- | --- | --- | | | | | --- | --- | --- | --- | --- | --- | | | | | --- | --- | --- | --- | --- | --- | --- | --- | --- | | | | | --- | --- | --- | --- | --- | --- | --- | --- | --- | --- | --- | --- | | | |
| --- | --- | --- | --- | --- | --- | --- | --- | --- | --- | --- | --- | --- | --- | --- |

  
  

---

  
  

# Cross references

  

|
|  |
| **UniGene** |
| Mm.15691 |
|
| **Agilent** |
| A\_51\_P222467 |
|
| **Ensembl** |
| ENSMUSG00000024030 |
|
| **Illumina** |
| ILMN\_1245964 |
|
| **Entrez Gene** |
| 11307 |
|
| **MGI** |
| MGI:107704 |
|
| **RefSeq** |
| NM\_009593 |
| NP\_033723 |
|
| **Uniprot/TrEMBL** |
| Q0VDW9 |
| Q64343 |
|
| **GeneOntology** |
| GO:0005524 |
| GO:0005548 |
| GO:0005739 |
| GO:0005768 |
| GO:0005794 |
| GO:0005886 |
| GO:0006355 |
| GO:0008203 |
| GO:0009897 |
| GO:0010033 |
| GO:0010745 |
| GO:0010872 |
| GO:0010875 |
| GO:0010888 |
| GO:0016021 |
| GO:0017127 |
| GO:0019534 |
| GO:0030301 |
| GO:0032367 |
| GO:0033344 |
| GO:0033700 |
| GO:0033993 |
| GO:0034041 |
| GO:0034374 |
| GO:0034375 |
| GO:0034436 |
| GO:0034437 |
| GO:0042632 |
| GO:0042803 |
| GO:0042987 |
| GO:0043531 |
| GO:0043691 |
| GO:0045542 |
| GO:0046982 |
| GO:0055037 |
| GO:0055091 |
| GO:0055099 |
| GO:1901998 |
|
| **UCSC Genome Browser** |
| uc008buj.2 |
|
| **WikiGenes** |
| 11307 |
|
| **Affy** |
| 10443730 |
| 1423570\_at |
| 160612\_at |
| u34920\_s\_at |
