# Supplementary material for: Automatically visualise and analyse data on pathways using PathVisioRPC from any programming environment
Source: BMC Bioinformatics. 2015 Aug 23;16(1):267. doi: 10.1186/s12859-015-0708-8 (PMC4546821; doi:10.1186/s12859-015-0708-8)
Supplement: Additional file 3: — Examples in Python. This zip archive contains the data and python script for the three python examples. (ZIP 15714 kb) [file 12859_2015_708_MOESM3_ESM.zip › Python_Examples/result_Example_2/geneList/backpage/L_11308.html]

 

# geneproduct annotation

  

| Name: Abi1| Identifier: 11308| Database: Entrez Gene| Synonyms: E3B1 | | | --- | --- | | | | --- | --- | --- | --- | | | | --- | --- | --- | --- | --- | --- | | |
| --- | --- | --- | --- | --- | --- | --- | --- |

# Expression data

**Gene id on mapp: 11308**

| Sample name 11308 11308| SystemCode L L| LogFC 0.0 0.0| Pvalue 0.101553557 0.43253426| Type trans-PPS2 trans-PPS3 | | | | --- | --- | --- | | | | | --- | --- | --- | --- | --- | --- | | | | | --- | --- | --- | --- | --- | --- | --- | --- | --- | | | | | --- | --- | --- | --- | --- | --- | --- | --- | --- | --- | --- | --- | | | |
| --- | --- | --- | --- | --- | --- | --- | --- | --- | --- | --- | --- | --- | --- | --- |

  
  

---

  
  

# Cross references

  

|
|  |
| **UniGene** |
| Mm.205647 |
| Mm.472166 |
| Mm.487780 |
|
| **Agilent** |
| A\_30\_P01026813 |
| A\_51\_P392963 |
| A\_52\_P157274 |
|
| **Ensembl** |
| ENSMUSG00000058835 |
|
| **Illumina** |
| ILMN\_1234139 |
| ILMN\_2512032 |
| ILMN\_3094886 |
|
| **Entrez Gene** |
| 11308 |
|
| **MGI** |
| MGI:104913 |
|
| **RefSeq** |
| NM\_001077190 |
| NM\_001077192 |
| NM\_001077193 |
| NM\_007380 |
| NM\_145994 |
| NP\_001070658 |
| NP\_001070660 |
| NP\_001070661 |
| NP\_031406 |
| NP\_666106 |
|
| **Uniprot/TrEMBL** |
| B7ZCU0 |
| B7ZCU2 |
| B7ZCU3 |
| B7ZCU4 |
| B7ZCU5 |
| J3QNK8 |
| Q3TJ64 |
| Q3TJR5 |
| Q3TPY5 |
| Q3UBL3 |
| Q8CBW3 |
|
| **GeneOntology** |
| GO:0001756 |
| GO:0005515 |
| GO:0005622 |
| GO:0005634 |
| GO:0005737 |
| GO:0005856 |
| GO:0006928 |
| GO:0009987 |
| GO:0018108 |
| GO:0030027 |
| GO:0030054 |
| GO:0030175 |
| GO:0030296 |
| GO:0030426 |
| GO:0031252 |
| GO:0035855 |
| GO:0043005 |
| GO:0045202 |
| GO:0061098 |
| GO:0072673 |
|
| **UCSC Genome Browser** |
| uc008ins.1 |
| uc008int.1 |
| uc008inu.1 |
| uc008inv.1 |
| uc008inw.1 |
|
| **WikiGenes** |
| 11308 |
|
| **Affy** |
| 101037\_at |
| 10480423 |
| 1423177\_a\_at |
| 1423178\_at |
| 1438506\_s\_at |
| 1450890\_a\_at |
| U17698\_s\_at |
