# Supplementary material for: Automatically visualise and analyse data on pathways using PathVisioRPC from any programming environment
Source: BMC Bioinformatics. 2015 Aug 23;16(1):267. doi: 10.1186/s12859-015-0708-8 (PMC4546821; doi:10.1186/s12859-015-0708-8)
Supplement: Additional file 3: — Examples in Python. This zip archive contains the data and python script for the three python examples. (ZIP 15714 kb) [file 12859_2015_708_MOESM3_ESM.zip › Python_Examples/result_Example_2/geneList/backpage/L_11363.html]

 

# geneproduct annotation

  

| Name: Acadl| Identifier: 11363| Database: Entrez Gene| Synonyms: AA960361 | | | --- | --- | | | | --- | --- | --- | --- | | | | --- | --- | --- | --- | --- | --- | | |
| --- | --- | --- | --- | --- | --- | --- | --- |

# Expression data

**Gene id on mapp: 11363**

| Sample name 11363 11363| SystemCode L L| LogFC 0.0 1.07332229| Pvalue 0.130076623 0.040766615| Type trans-PPS2 trans-PPS3 | | | | --- | --- | --- | | | | | --- | --- | --- | --- | --- | --- | | | | | --- | --- | --- | --- | --- | --- | --- | --- | --- | | | | | --- | --- | --- | --- | --- | --- | --- | --- | --- | --- | --- | --- | | | |
| --- | --- | --- | --- | --- | --- | --- | --- | --- | --- | --- | --- | --- | --- | --- |

  
  

---

  
  

# Cross references

  

|
|  |
| **UniGene** |
| Mm.2445 |
| Mm.400243 |
|
| **Agilent** |
| A\_51\_P149455 |
|
| **Ensembl** |
| ENSMUSG00000026003 |
|
| **Illumina** |
| ILMN\_2689473 |
| ILMN\_2735084 |
| ILMN\_2783519 |
|
| **Entrez Gene** |
| 11363 |
|
| **MGI** |
| MGI:87866 |
|
| **RefSeq** |
| NM\_007381 |
| NP\_031407 |
|
| **Uniprot/TrEMBL** |
| P51174 |
|
| **GeneOntology** |
| GO:0000062 |
| GO:0001659 |
| GO:0004466 |
| GO:0005737 |
| GO:0005739 |
| GO:0005759 |
| GO:0016401 |
| GO:0019254 |
| GO:0031966 |
| GO:0033539 |
| GO:0042413 |
| GO:0042758 |
| GO:0044242 |
| GO:0045717 |
| GO:0046322 |
| GO:0050660 |
| GO:0051289 |
| GO:0055114 |
| GO:0090181 |
|
| **UCSC Genome Browser** |
| uc007bir.1 |
|
| **WikiGenes** |
| 11363 |
|
| **Affy** |
| 10355246 |
| 1448987\_at |
| 1448988\_at |
| 95425\_at |
| U21489\_s\_at |
