# Supplementary material for: Automatically visualise and analyse data on pathways using PathVisioRPC from any programming environment
Source: BMC Bioinformatics. 2015 Aug 23;16(1):267. doi: 10.1186/s12859-015-0708-8 (PMC4546821; doi:10.1186/s12859-015-0708-8)
Supplement: Additional file 3: — Examples in Python. This zip archive contains the data and python script for the three python examples. (ZIP 15714 kb) [file 12859_2015_708_MOESM3_ESM.zip › Python_Examples/result_Example_2/geneList/backpage/L_11364.html]

 

# geneproduct annotation

  

| Name: Acadm| Identifier: 11364| Database: Entrez Gene| Synonyms: MCAD | | | --- | --- | | | | --- | --- | --- | --- | | | | --- | --- | --- | --- | --- | --- | | |
| --- | --- | --- | --- | --- | --- | --- | --- |

# Expression data

**Gene id on mapp: 11364**

| Sample name 11364 11364| SystemCode L L| LogFC 1.205299046 1.086842073| Pvalue 0.006149239 0.030289045| Type trans-PPS2 trans-PPS3 | | | | --- | --- | --- | | | | | --- | --- | --- | --- | --- | --- | | | | | --- | --- | --- | --- | --- | --- | --- | --- | --- | | | | | --- | --- | --- | --- | --- | --- | --- | --- | --- | --- | --- | --- | | | |
| --- | --- | --- | --- | --- | --- | --- | --- | --- | --- | --- | --- | --- | --- | --- |

  
  

---

  
  

# Cross references

  

|
|  |
| **UniGene** |
| Mm.10530 |
| Mm.363481 |
| Mm.404254 |
|
| **Agilent** |
| A\_51\_P319879 |
|
| **Ensembl** |
| ENSMUSG00000062908 |
|
| **Illumina** |
| ILMN\_1256019 |
| ILMN\_2776209 |
| ILMN\_2810473 |
|
| **Entrez Gene** |
| 11364 |
|
| **MGI** |
| MGI:87867 |
|
| **RefSeq** |
| NM\_007382 |
| NP\_031408 |
|
| **Uniprot/TrEMBL** |
| D3Z2A5 |
| D6RFD7 |
| P45952 |
| Q3V2I9 |
|
| **GeneOntology** |
| GO:0001889 |
| GO:0003995 |
| GO:0005739 |
| GO:0005759 |
| GO:0005978 |
| GO:0006082 |
| GO:0006111 |
| GO:0006635 |
| GO:0007507 |
| GO:0009409 |
| GO:0009437 |
| GO:0009791 |
| GO:0016627 |
| GO:0019254 |
| GO:0030424 |
| GO:0033539 |
| GO:0042594 |
| GO:0042802 |
| GO:0045329 |
| GO:0050660 |
| GO:0051791 |
| GO:0051793 |
| GO:0055007 |
| GO:0055114 |
| GO:0070991 |
|
| **UCSC Genome Browser** |
| uc008ruj.1 |
|
| **WikiGenes** |
| 11364 |
|
| **Affy** |
| 10502951 |
| 1415984\_at |
| 92581\_at |
| Msa.1660.0\_f\_at |
| U07159\_f\_at |
