# Supplementary material for: Automatically visualise and analyse data on pathways using PathVisioRPC from any programming environment
Source: BMC Bioinformatics. 2015 Aug 23;16(1):267. doi: 10.1186/s12859-015-0708-8 (PMC4546821; doi:10.1186/s12859-015-0708-8)
Supplement: Additional file 3: — Examples in Python. This zip archive contains the data and python script for the three python examples. (ZIP 15714 kb) [file 12859_2015_708_MOESM3_ESM.zip › Python_Examples/result_Example_2/geneList/backpage/L_11409.html]

 

# geneproduct annotation

  

| Name: Acads| Identifier: 11409| Database: Entrez Gene| Synonyms: Bcd-1 | | | --- | --- | | | | --- | --- | --- | --- | | | | --- | --- | --- | --- | --- | --- | | |
| --- | --- | --- | --- | --- | --- | --- | --- |

# Expression data

**Gene id on mapp: 11409**

| Sample name 11409 11409| SystemCode L L| LogFC 1.492693834 1.134587196| Pvalue 0.003003625 0.010977924| Type trans-PPS2 trans-PPS3 | | | | --- | --- | --- | | | | | --- | --- | --- | --- | --- | --- | | | | | --- | --- | --- | --- | --- | --- | --- | --- | --- | | | | | --- | --- | --- | --- | --- | --- | --- | --- | --- | --- | --- | --- | | | |
| --- | --- | --- | --- | --- | --- | --- | --- | --- | --- | --- | --- | --- | --- | --- |

  
  

---

  
  

# Cross references

  

|
|  |
| **UniGene** |
| Mm.18759 |
|
| **Agilent** |
| A\_52\_P367745 |
|
| **Ensembl** |
| ENSMUSG00000029545 |
|
| **Illumina** |
| ILMN\_1256725 |
| ILMN\_2806676 |
|
| **Entrez Gene** |
| 11409 |
|
| **MGI** |
| MGI:87868 |
|
| **RefSeq** |
| NM\_007383 |
| NP\_031409 |
|
| **Uniprot/TrEMBL** |
| Q07417 |
|
| **GeneOntology** |
| GO:0000062 |
| GO:0003995 |
| GO:0004085 |
| GO:0005739 |
| GO:0005759 |
| GO:0031966 |
| GO:0033539 |
| GO:0042594 |
| GO:0046359 |
| GO:0050660 |
| GO:0051289 |
| GO:0051384 |
|
| **UCSC Genome Browser** |
| uc008zdb.1 |
|
| **WikiGenes** |
| 11409 |
|
| **Affy** |
| 103401\_at |
| 10532926 |
| 1460216\_at |
| L11163\_s\_at |
