# Supplementary material for: Automatically visualise and analyse data on pathways using PathVisioRPC from any programming environment
Source: BMC Bioinformatics. 2015 Aug 23;16(1):267. doi: 10.1186/s12859-015-0708-8 (PMC4546821; doi:10.1186/s12859-015-0708-8)
Supplement: Additional file 3: — Examples in Python. This zip archive contains the data and python script for the three python examples. (ZIP 15714 kb) [file 12859_2015_708_MOESM3_ESM.zip › Python_Examples/result_Example_2/geneList/backpage/L_11425.html]

 

# geneproduct annotation

  

| Name: Apoc4| Identifier: 11425| Database: Entrez Gene| Synonyms: Acl | | | --- | --- | | | | --- | --- | --- | --- | | | | --- | --- | --- | --- | --- | --- | | |
| --- | --- | --- | --- | --- | --- | --- | --- |

# Expression data

**Gene id on mapp: 11425**

| Sample name 11425 11425| SystemCode L L| LogFC 0.0 0.0| Pvalue 0.623450147 0.66757898| Type trans-PPS2 trans-PPS3 | | | | --- | --- | --- | | | | | --- | --- | --- | --- | --- | --- | | | | | --- | --- | --- | --- | --- | --- | --- | --- | --- | | | | | --- | --- | --- | --- | --- | --- | --- | --- | --- | --- | --- | --- | | | |
| --- | --- | --- | --- | --- | --- | --- | --- | --- | --- | --- | --- | --- | --- | --- |

  
  

---

  
  

# Cross references

  

|
|  |
| **Agilent** |
| A\_51\_P460332 |
|
| **Ensembl** |
| ENSMUSG00000074336 |
|
| **Illumina** |
| ILMN\_1234764 |
| ILMN\_3009225 |
|
| **Entrez Gene** |
| 11425 |
|
| **MGI** |
| MGI:87878 |
|
| **RefSeq** |
| NM\_007385 |
| NP\_031411 |
|
| **Uniprot/TrEMBL** |
| Q61268 |
|
| **GeneOntology** |
| GO:0006869 |
| GO:0034361 |
|
| **UCSC Genome Browser** |
| uc009fmu.1 |
|
| **WikiGenes** |
| 11425 |
|
| **Affy** |
| 101463\_at |
| 10560614 |
| 1418708\_at |
| Msa.2146.0\_f\_at |
