# Supplementary material for: Automatically visualise and analyse data on pathways using PathVisioRPC from any programming environment
Source: BMC Bioinformatics. 2015 Aug 23;16(1):267. doi: 10.1186/s12859-015-0708-8 (PMC4546821; doi:10.1186/s12859-015-0708-8)
Supplement: Additional file 3: — Examples in Python. This zip archive contains the data and python script for the three python examples. (ZIP 15714 kb) [file 12859_2015_708_MOESM3_ESM.zip › Python_Examples/result_Example_2/geneList/backpage/L_11426.html]

 

# geneproduct annotation

  

| Name: Macf1| Identifier: 11426| Database: Entrez Gene| Synonyms: Aclp7 | | | --- | --- | | | | --- | --- | --- | --- | | | | --- | --- | --- | --- | --- | --- | | |
| --- | --- | --- | --- | --- | --- | --- | --- |

# Expression data

**Gene id on mapp: 11426**

| Sample name 11426 11426| SystemCode L L| LogFC 0.0 -1.126572286| Pvalue 0.777479937 0.025064495| Type trans-PPS2 trans-PPS3 | | | | --- | --- | --- | | | | | --- | --- | --- | --- | --- | --- | | | | | --- | --- | --- | --- | --- | --- | --- | --- | --- | | | | | --- | --- | --- | --- | --- | --- | --- | --- | --- | --- | --- | --- | | | |
| --- | --- | --- | --- | --- | --- | --- | --- | --- | --- | --- | --- | --- | --- | --- |

  
  

---

  
  

# Cross references

  

|
|  |
| **UniGene** |
| Mm.402299 |
| Mm.466074 |
| Mm.472205 |
| Mm.485351 |
| Mm.487445 |
|
| **Agilent** |
| A\_30\_P01020812 |
| A\_30\_P01030784 |
| A\_30\_P01032994 |
| A\_51\_P115315 |
| A\_51\_P220615 |
| A\_55\_P2017684 |
|
| **Ensembl** |
| ENSMUSG00000028649 |
|
| **Illumina** |
| ILMN\_1216022 |
| ILMN\_1242789 |
| ILMN\_1251266 |
| ILMN\_1259884 |
| ILMN\_2620934 |
| ILMN\_2744146 |
|
| **Entrez Gene** |
| 11426 |
|
| **MGI** |
| MGI:108559 |
|
| **RefSeq** |
| NM\_001199136 |
| NM\_001199137 |
| NP\_001186065 |
| NP\_001186066 |
|
| **Uniprot/TrEMBL** |
| B1ARU1 |
| B1ARU4 |
| E9PVY8 |
| E9QA63 |
| E9QNP1 |
| F6Q750 |
| F6SHS0 |
| F6XCT0 |
| F6YKN8 |
| F7AB76 |
| F7ACR9 |
| Q3TRV6 |
| Q3UPG9 |
| Q4QQN2 |
| Q80TZ5 |
| Q80ZZ3 |
| Q9CYG3 |
| Q9QXZ0 |
|
| **GeneOntology** |
| GO:0001707 |
| GO:0003779 |
| GO:0005509 |
| GO:0005515 |
| GO:0005737 |
| GO:0005794 |
| GO:0005874 |
| GO:0005886 |
| GO:0006200 |
| GO:0006620 |
| GO:0006928 |
| GO:0007050 |
| GO:0007163 |
| GO:0008017 |
| GO:0010632 |
| GO:0015629 |
| GO:0015630 |
| GO:0016020 |
| GO:0016055 |
| GO:0016887 |
| GO:0030177 |
| GO:0032587 |
| GO:0032886 |
| GO:0042060 |
| GO:0043001 |
| GO:0051893 |
|
| **UCSC Genome Browser** |
| uc008upi.2 |
| uc008upj.2 |
| uc008upl.2 |
| uc008upq.1 |
| uc008upr.2 |
|
| **WikiGenes** |
| 11426 |
|
| **Affy** |
| 10516103 |
| 134288\_at |
| 1428847\_a\_at |
| 1428848\_a\_at |
| 98402\_at |
| aa289470\_s\_at |
