# Supplementary material for: Automatically visualise and analyse data on pathways using PathVisioRPC from any programming environment
Source: BMC Bioinformatics. 2015 Aug 23;16(1):267. doi: 10.1186/s12859-015-0708-8 (PMC4546821; doi:10.1186/s12859-015-0708-8)
Supplement: Additional file 3: — Examples in Python. This zip archive contains the data and python script for the three python examples. (ZIP 15714 kb) [file 12859_2015_708_MOESM3_ESM.zip › Python_Examples/result_Example_2/geneList/backpage/L_11428.html]

 

# geneproduct annotation

  

| Name: Aco1| Identifier: 11428| Database: Entrez Gene| Synonyms: Irebp | | | --- | --- | | | | --- | --- | --- | --- | | | | --- | --- | --- | --- | --- | --- | | |
| --- | --- | --- | --- | --- | --- | --- | --- |

# Expression data

**Gene id on mapp: 11428**

| Sample name 11428 11428| SystemCode L L| LogFC 1.146311791 1.147109788| Pvalue 0.017452393 1.93755E-4| Type trans-PPS2 trans-PPS3 | | | | --- | --- | --- | | | | | --- | --- | --- | --- | --- | --- | | | | | --- | --- | --- | --- | --- | --- | --- | --- | --- | | | | | --- | --- | --- | --- | --- | --- | --- | --- | --- | --- | --- | --- | | | |
| --- | --- | --- | --- | --- | --- | --- | --- | --- | --- | --- | --- | --- | --- | --- |

  
  

---

  
  

# Cross references

  

|
|  |
| **UniGene** |
| Mm.331547 |
|
| **Agilent** |
| A\_51\_P391082 |
| A\_52\_P299115 |
| A\_55\_P2085880 |
|
| **Ensembl** |
| ENSMUSG00000028405 |
|
| **Illumina** |
| ILMN\_1216382 |
|
| **Entrez Gene** |
| 11428 |
|
| **MGI** |
| MGI:87879 |
|
| **RefSeq** |
| NM\_007386 |
| NP\_031412 |
|
| **Uniprot/TrEMBL** |
| P28271 |
| Q8VDC3 |
|
| **GeneOntology** |
| GO:0003994 |
| GO:0005515 |
| GO:0005737 |
| GO:0005739 |
| GO:0005783 |
| GO:0005794 |
| GO:0005829 |
| GO:0006099 |
| GO:0006101 |
| GO:0006417 |
| GO:0006879 |
| GO:0009791 |
| GO:0010040 |
| GO:0010468 |
| GO:0030350 |
| GO:0046872 |
| GO:0050892 |
| GO:0051539 |
| GO:0052632 |
| GO:0052633 |
|
| **UCSC Genome Browser** |
| uc008shd.1 |
|
| **WikiGenes** |
| 11428 |
|
| **Affy** |
| 100147\_at |
| 10503966 |
| 137501\_f\_at |
| 1423644\_at |
| 1456728\_x\_at |
| X61147\_s\_at |
