# Supplementary material for: Automatically visualise and analyse data on pathways using PathVisioRPC from any programming environment
Source: BMC Bioinformatics. 2015 Aug 23;16(1):267. doi: 10.1186/s12859-015-0708-8 (PMC4546821; doi:10.1186/s12859-015-0708-8)
Supplement: Additional file 3: — Examples in Python. This zip archive contains the data and python script for the three python examples. (ZIP 15714 kb) [file 12859_2015_708_MOESM3_ESM.zip › Python_Examples/result_Example_2/geneList/backpage/L_11429.html]

 

# geneproduct annotation

  

| Name: Aco2| Identifier: 11429| Database: Entrez Gene| Synonyms: Aco3 | | | --- | --- | | | | --- | --- | --- | --- | | | | --- | --- | --- | --- | --- | --- | | |
| --- | --- | --- | --- | --- | --- | --- | --- |

# Expression data

**Gene id on mapp: 11429**

| Sample name 11429 11429| SystemCode L L| LogFC 0.0 0.0| Pvalue 0.845095676 0.152083731| Type trans-PPS2 trans-PPS3 | | | | --- | --- | --- | | | | | --- | --- | --- | --- | --- | --- | | | | | --- | --- | --- | --- | --- | --- | --- | --- | --- | | | | | --- | --- | --- | --- | --- | --- | --- | --- | --- | --- | --- | --- | | | |
| --- | --- | --- | --- | --- | --- | --- | --- | --- | --- | --- | --- | --- | --- | --- |

  
  

---

  
  

# Cross references

  

|
|  |
| **Agilent** |
| A\_52\_P200359 |
|
| **Ensembl** |
| ENSMUSG00000022477 |
|
| **Illumina** |
| ILMN\_1235786 |
| ILMN\_2748837 |
|
| **Entrez Gene** |
| 11429 |
|
| **MGI** |
| MGI:87880 |
|
| **RefSeq** |
| NM\_080633 |
| NP\_542364 |
|
| **Uniprot/TrEMBL** |
| Q99KI0 |
|
| **GeneOntology** |
| GO:0003994 |
| GO:0005506 |
| GO:0005515 |
| GO:0005634 |
| GO:0005739 |
| GO:0006099 |
| GO:0006101 |
| GO:0006102 |
| GO:0051538 |
| GO:0051539 |
| GO:0052632 |
| GO:0052633 |
|
| **UCSC Genome Browser** |
| uc007wxp.1 |
|
| **WikiGenes** |
| 11429 |
|
| **Affy** |
| 10425611 |
| 1436934\_s\_at |
| 1451002\_at |
| 96870\_at |
| aa560856\_at |
| aa560856\_g\_at |
