# Supplementary material for: Automatically visualise and analyse data on pathways using PathVisioRPC from any programming environment
Source: BMC Bioinformatics. 2015 Aug 23;16(1):267. doi: 10.1186/s12859-015-0708-8 (PMC4546821; doi:10.1186/s12859-015-0708-8)
Supplement: Additional file 3: — Examples in Python. This zip archive contains the data and python script for the three python examples. (ZIP 15714 kb) [file 12859_2015_708_MOESM3_ESM.zip › Python_Examples/result_Example_3/Cholesterol Biosynthesis/backpage/L_10654.html]

 

# GeneProduct annotation

  

| Name: PMVK| Identifier: 10654| Database: Entrez Gene| Synonyms: HUMPMKI | | | --- | --- | | | | --- | --- | --- | --- | | | | --- | --- | --- | --- | --- | --- | | |
| --- | --- | --- | --- | --- | --- | --- | --- |

# Expression data

**Gene id on mapp: 10654**

| Sample name 10654| logFC1 0.503529322| Pvalue1 0.484834459| logFC2 1.304714294| Pvalue2 0.45091839 | | | --- | --- | | | | --- | --- | --- | --- | | | | --- | --- | --- | --- | --- | --- | | | | --- | --- | --- | --- | --- | --- | --- | --- | | |
| --- | --- | --- | --- | --- | --- | --- | --- | --- | --- |

  
  

---

  
  

# Cross references

  

|
|  |
| **UniGene** |
| Hs.30954 |
|
| **Agilent** |
| A\_23\_P114774 |
|
| **Ensembl** |
| ENSG00000163344 |
|
| **Gene Wiki** |
| 10654 |
|
| **HGNC** |
| PMVK |
|
| **Illumina** |
| 0001400438 |
| ILMN\_1715896 |
|
| **Entrez Gene** |
| 10654 |
|
| **OMIM** |
| 607622 |
|
| **PDB** |
| 3CH4 |
|
| **RefSeq** |
| NM\_006556 |
| NP\_006547 |
|
| **Uniprot/TrEMBL** |
| Q15126 |
| Q6FGV9 |
|
| **GeneOntology** |
| GO:0004631 |
| GO:0005524 |
| GO:0005737 |
| GO:0005777 |
| GO:0005829 |
| GO:0006695 |
| GO:0008299 |
| GO:0016126 |
| GO:0019287 |
| GO:0044281 |
| GO:0070723 |
|
| **UCSC Genome Browser** |
| uc001ffq.3 |
|
| **WikiGenes** |
| 10654 |
|
| **Affy** |
| 11730547\_a\_at |
| 11754085\_a\_at |
| 11756161\_x\_at |
| 203515\_s\_at |
| 35621\_at |
| 7920567 |
| L77213\_at |
