# Supplementary material for: Automatically visualise and analyse data on pathways using PathVisioRPC from any programming environment
Source: BMC Bioinformatics. 2015 Aug 23;16(1):267. doi: 10.1186/s12859-015-0708-8 (PMC4546821; doi:10.1186/s12859-015-0708-8)
Supplement: Additional file 3: — Examples in Python. This zip archive contains the data and python script for the three python examples. (ZIP 15714 kb) [file 12859_2015_708_MOESM3_ESM.zip › Python_Examples/result_Example_3/Cholesterol Biosynthesis/backpage/L_110196.html]

 

# GeneProduct annotation

  

| Name: Fdps| Identifier: 110196| Database: Entrez Gene| Synonyms: 6030492I17Rik | | | --- | --- | | | | --- | --- | --- | --- | | | | --- | --- | --- | --- | --- | --- | | |
| --- | --- | --- | --- | --- | --- | --- | --- |

# Expression data

**Gene id on mapp: 110196**

| Sample name 110196 110196| logFC 0.064564752 1.169488202| Pvalue 0.052880943 2.85879E-4 | | | | --- | --- | --- | | | | | --- | --- | --- | --- | --- | --- | | | |
| --- | --- | --- | --- | --- | --- | --- | --- | --- |

  
  

---

  
  

# Cross references

  

|
|  |
| **UniGene** |
| Mm.39472 |
| Mm.467570 |
|
| **Agilent** |
| A\_51\_P379798 |
| A\_55\_P1966804 |
|
| **Ensembl** |
| ENSMUSG00000059743 |
|
| **Illumina** |
| ILMN\_1225730 |
|
| **Entrez Gene** |
| 110196 |
|
| **MGI** |
| MGI:104888 |
|
| **RefSeq** |
| NM\_001253751 |
| NM\_134469 |
| NP\_001240680 |
| NP\_608219 |
|
| **Uniprot/TrEMBL** |
| Q4FJN9 |
| Q920E5 |
|
| **GeneOntology** |
| GO:0004161 |
| GO:0004337 |
| GO:0005634 |
| GO:0005737 |
| GO:0005739 |
| GO:0006695 |
| GO:0033384 |
| GO:0045337 |
| GO:0046872 |
|
| **UCSC Genome Browser** |
| uc008pxo.2 |
|
| **WikiGenes** |
| 110196 |
|
| **Affy** |
| 10499483 |
| 1423418\_at |
| 160424\_f\_at |
| 99098\_at |
| Msa.4694.0\_f\_at |
