# Supplementary material for: Automatically visualise and analyse data on pathways using PathVisioRPC from any programming environment
Source: BMC Bioinformatics. 2015 Aug 23;16(1):267. doi: 10.1186/s12859-015-0708-8 (PMC4546821; doi:10.1186/s12859-015-0708-8)
Supplement: Additional file 3: — Examples in Python. This zip archive contains the data and python script for the three python examples. (ZIP 15714 kb) [file 12859_2015_708_MOESM3_ESM.zip › Python_Examples/result_Example_3/Cholesterol Biosynthesis/backpage/L_13121.html]

 

# GeneProduct annotation

  

| Name: Cyp51| Identifier: 13121| Database: Entrez Gene| Synonyms: Cyp51a1 | | | --- | --- | | | | --- | --- | --- | --- | | | | --- | --- | --- | --- | --- | --- | | |
| --- | --- | --- | --- | --- | --- | --- | --- |

# Expression data

**Gene id on mapp: 13121**

| Sample name 13121| logFC 1.544758333| Pvalue 0.820338686 | | | --- | --- | | | | --- | --- | --- | --- | | |
| --- | --- | --- | --- | --- | --- |

  
  

---

  
  

# Cross references

  

|
|  |
| **UniGene** |
| Mm.140158 |
| Mm.396259 |
| Mm.398854 |
| Mm.452707 |
|
| **Agilent** |
| A\_51\_P485791 |
| A\_52\_P164161 |
| A\_52\_P636752 |
|
| **Ensembl** |
| ENSMUSG00000001467 |
|
| **Illumina** |
| ILMN\_2428754 |
| ILMN\_2688075 |
|
| **Entrez Gene** |
| 13121 |
|
| **MGI** |
| MGI:106040 |
|
| **RefSeq** |
| NM\_020010 |
| NP\_064394 |
|
| **Uniprot/TrEMBL** |
| Q8K0C4 |
| Q9CW87 |
|
| **GeneOntology** |
| GO:0005506 |
| GO:0005789 |
| GO:0006694 |
| GO:0006695 |
| GO:0008398 |
| GO:0009055 |
| GO:0016021 |
| GO:0020037 |
| GO:0033488 |
| GO:0043231 |
|
| **UCSC Genome Browser** |
| uc008wie.1 |
|
| **WikiGenes** |
| 13121 |
|
| **Affy** |
| 10527920 |
| 109608\_at |
| 131655\_s\_at |
| 137321\_at |
| 1422533\_at |
| 1422534\_at |
| 1450646\_at |
| 94916\_at |
