# Supplementary material for: Automatically visualise and analyse data on pathways using PathVisioRPC from any programming environment
Source: BMC Bioinformatics. 2015 Aug 23;16(1):267. doi: 10.1186/s12859-015-0708-8 (PMC4546821; doi:10.1186/s12859-015-0708-8)
Supplement: Additional file 3: — Examples in Python. This zip archive contains the data and python script for the three python examples. (ZIP 15714 kb) [file 12859_2015_708_MOESM3_ESM.zip › Python_Examples/result_Example_3/Cholesterol Biosynthesis/backpage/L_13360.html]

 

# GeneProduct annotation

  

| Name: Dhcr7| Identifier: 13360| Database: Entrez Gene| Synonyms: AA409147 | | | --- | --- | | | | --- | --- | --- | --- | | | | --- | --- | --- | --- | --- | --- | | |
| --- | --- | --- | --- | --- | --- | --- | --- |

# Expression data

**Gene id on mapp: 13360**

| Sample name 13360| logFC 1.290774071| Pvalue 0.790754162 | | | --- | --- | | | | --- | --- | --- | --- | | |
| --- | --- | --- | --- | --- | --- |

  
  

---

  
  

# Cross references

  

|
|  |
| **UniGene** |
| Mm.249342 |
|
| **Agilent** |
| A\_51\_P290986 |
| A\_52\_P535946 |
| A\_55\_P2022337 |
|
| **Ensembl** |
| ENSMUSG00000058454 |
|
| **Illumina** |
| ILMN\_2537782 |
| ILMN\_2660471 |
|
| **Entrez Gene** |
| 13360 |
|
| **MGI** |
| MGI:1298378 |
|
| **RefSeq** |
| NM\_007856 |
| NP\_031882 |
|
| **Uniprot/TrEMBL** |
| D3YXR4 |
| D3Z7G1 |
| O88455 |
|
| **GeneOntology** |
| GO:0001568 |
| GO:0005640 |
| GO:0005783 |
| GO:0005789 |
| GO:0006695 |
| GO:0009791 |
| GO:0016020 |
| GO:0016021 |
| GO:0016126 |
| GO:0030154 |
| GO:0030324 |
| GO:0035264 |
| GO:0042127 |
| GO:0043231 |
| GO:0045540 |
| GO:0047598 |
|
| **UCSC Genome Browser** |
| uc009kqc.1 |
|
| **WikiGenes** |
| 13360 |
|
| **Affy** |
| 10559312 |
| 129090\_at |
| 1442696\_at |
| 1448619\_at |
| 98989\_at |
| aa409148\_at |
