# Supplementary material for: Automatically visualise and analyse data on pathways using PathVisioRPC from any programming environment
Source: BMC Bioinformatics. 2015 Aug 23;16(1):267. doi: 10.1186/s12859-015-0708-8 (PMC4546821; doi:10.1186/s12859-015-0708-8)
Supplement: Additional file 3: — Examples in Python. This zip archive contains the data and python script for the three python examples. (ZIP 15714 kb) [file 12859_2015_708_MOESM3_ESM.zip › Python_Examples/result_Example_3/Cholesterol Biosynthesis/backpage/L_14137.html]

 

# GeneProduct annotation

  

| Name: Fdft1| Identifier: 14137| Database: Entrez Gene| Synonyms: SS | | | --- | --- | | | | --- | --- | --- | --- | | | | --- | --- | --- | --- | --- | --- | | |
| --- | --- | --- | --- | --- | --- | --- | --- |

# Expression data

**Gene id on mapp: 14137**

| Sample name 14137| logFC 2.169939763| Pvalue 0.080974901 | | | --- | --- | | | | --- | --- | --- | --- | | |
| --- | --- | --- | --- | --- | --- |

  
  

---

  
  

# Cross references

  

|
|  |
| **UniGene** |
| Mm.422233 |
| Mm.474432 |
|
| **Agilent** |
| A\_51\_P485946 |
| A\_52\_P136138 |
| A\_55\_P2033250 |
|
| **Ensembl** |
| ENSMUSG00000021273 |
|
| **Illumina** |
| ILMN\_2593604 |
|
| **Entrez Gene** |
| 14137 |
|
| **MGI** |
| MGI:102706 |
|
| **RefSeq** |
| NM\_010191 |
| NP\_034321 |
|
| **Uniprot/TrEMBL** |
| P53798 |
|
| **GeneOntology** |
| GO:0004310 |
| GO:0005783 |
| GO:0005789 |
| GO:0006695 |
| GO:0008299 |
| GO:0016021 |
| GO:0016491 |
| GO:0043231 |
| GO:0045338 |
| GO:0051996 |
|
| **UCSC Genome Browser** |
| uc007uhi.2 |
|
| **WikiGenes** |
| 14137 |
|
| **Affy** |
| 10412909 |
| 10420730 |
| 1438322\_x\_at |
| 1448130\_at |
| 97518\_at |
| D29016\_s\_at |
