# Supplementary material for: Automatically visualise and analyse data on pathways using PathVisioRPC from any programming environment
Source: BMC Bioinformatics. 2015 Aug 23;16(1):267. doi: 10.1186/s12859-015-0708-8 (PMC4546821; doi:10.1186/s12859-015-0708-8)
Supplement: Additional file 3: — Examples in Python. This zip archive contains the data and python script for the three python examples. (ZIP 15714 kb) [file 12859_2015_708_MOESM3_ESM.zip › Python_Examples/result_Example_3/Cholesterol Biosynthesis/backpage/L_15357.html]

 

# GeneProduct annotation

  

| Name: Hmgcr| Identifier: 15357| Database: Entrez Gene| Synonyms: Red | | | --- | --- | | | | --- | --- | --- | --- | | | | --- | --- | --- | --- | --- | --- | | |
| --- | --- | --- | --- | --- | --- | --- | --- |

# Expression data

**Gene id on mapp: 15357**

| Sample name 15357| logFC 2.931028676| Pvalue 0.510081313 | | | --- | --- | | | | --- | --- | --- | --- | | |
| --- | --- | --- | --- | --- | --- |

  
  

---

  
  

# Cross references

  

|
|  |
| **UniGene** |
| Mm.316652 |
| Mm.489666 |
|
| **Agilent** |
| A\_51\_P507410 |
| A\_52\_P137371 |
| A\_52\_P232287 |
| A\_52\_P578922 |
|
| **Ensembl** |
| ENSMUSG00000021670 |
|
| **Illumina** |
| ILMN\_1225718 |
| ILMN\_1239292 |
|
| **Entrez Gene** |
| 15357 |
|
| **MGI** |
| MGI:96159 |
|
| **RefSeq** |
| NM\_008255 |
| NP\_032281 |
|
| **Uniprot/TrEMBL** |
| E9PVG9 |
| E9PY17 |
| E9Q149 |
| E9Q5H3 |
| F2Z470 |
| F6X166 |
| Q01237 |
| Q61671 |
|
| **GeneOntology** |
| GO:0004420 |
| GO:0005778 |
| GO:0005783 |
| GO:0005789 |
| GO:0006695 |
| GO:0006743 |
| GO:0007568 |
| GO:0007584 |
| GO:0008284 |
| GO:0008299 |
| GO:0008542 |
| GO:0009790 |
| GO:0010664 |
| GO:0010666 |
| GO:0015936 |
| GO:0016021 |
| GO:0032874 |
| GO:0042282 |
| GO:0042803 |
| GO:0043066 |
| GO:0043231 |
| GO:0043407 |
| GO:0045445 |
| GO:0045471 |
| GO:0045908 |
| GO:0048643 |
| GO:0048661 |
| GO:0050661 |
| GO:0050662 |
| GO:0051262 |
| GO:0061045 |
| GO:0061179 |
| GO:0070374 |
| GO:0070402 |
|
| **UCSC Genome Browser** |
| uc007rnm.3 |
| uc007rnn.1 |
|
| **WikiGenes** |
| 15357 |
|
| **Affy** |
| 10411332 |
| 104285\_at |
| 1427229\_at |
| 1451766\_at |
| 166342\_r\_at |
| 99425\_at |
| Msa.2345.0\_at |
| Msa.756.0\_at |
| Msa.756.0\_g\_at |
