# Supplementary material for: Automatically visualise and analyse data on pathways using PathVisioRPC from any programming environment
Source: BMC Bioinformatics. 2015 Aug 23;16(1):267. doi: 10.1186/s12859-015-0708-8 (PMC4546821; doi:10.1186/s12859-015-0708-8)
Supplement: Additional file 3: — Examples in Python. This zip archive contains the data and python script for the three python examples. (ZIP 15714 kb) [file 12859_2015_708_MOESM3_ESM.zip › Python_Examples/result_Example_3/Cholesterol Biosynthesis/backpage/L_1595.html]

 

# GeneProduct annotation

  

| Name: CYP51A1| Identifier: 1595| Database: Entrez Gene| Synonyms: CYP51 | | | --- | --- | | | | --- | --- | --- | --- | | | | --- | --- | --- | --- | --- | --- | | |
| --- | --- | --- | --- | --- | --- | --- | --- |

# Expression data

**Gene id on mapp: 1595**

| Sample name 1595| logFC1 1.313450141| Pvalue1 0.002111193| logFC2 1.414471821| Pvalue2 0.001037498 | | | --- | --- | | | | --- | --- | --- | --- | | | | --- | --- | --- | --- | --- | --- | | | | --- | --- | --- | --- | --- | --- | --- | --- | | |
| --- | --- | --- | --- | --- | --- | --- | --- | --- | --- |

  
  

---

  
  

# Cross references

  

|
|  |
| **UniGene** |
| Hs.417077 |
| Hs.602628 |
|
| **Agilent** |
| A\_23\_P257716 |
|
| **Ensembl** |
| ENSG00000001630 |
|
| **Gene Wiki** |
| 1595 |
|
| **HGNC** |
| CYP51A1 |
|
| **Illumina** |
| 0002650053 |
| ILMN\_1664718 |
|
| **Entrez Gene** |
| 1595 |
|
| **OMIM** |
| 601637 |
|
| **PDB** |
| 3JUS |
| 3JUV |
| 3LD6 |
|
| **RefSeq** |
| NM\_000786 |
| NM\_001146152 |
| NP\_000777 |
| NP\_001139624 |
|
| **Uniprot/TrEMBL** |
| C9IYR8 |
| Q16850 |
|
| **GeneOntology** |
| GO:0005506 |
| GO:0005789 |
| GO:0006694 |
| GO:0006695 |
| GO:0006805 |
| GO:0008398 |
| GO:0009055 |
| GO:0016021 |
| GO:0016705 |
| GO:0020037 |
| GO:0033488 |
| GO:0043231 |
| GO:0044281 |
| GO:0055114 |
|
| **UCSC Genome Browser** |
| uc003ulm.4 |
| uc011khn.2 |
|
| **WikiGenes** |
| 1595 |
|
| **Affy** |
| 11722563\_x\_at |
| 11722564\_at |
| 202314\_at |
| 33389\_at |
| 8140864 |
| 8180268 |
| U23942\_at |
