# Supplementary material for: Automatically visualise and analyse data on pathways using PathVisioRPC from any programming environment
Source: BMC Bioinformatics. 2015 Aug 23;16(1):267. doi: 10.1186/s12859-015-0708-8 (PMC4546821; doi:10.1186/s12859-015-0708-8)
Supplement: Additional file 3: — Examples in Python. This zip archive contains the data and python script for the three python examples. (ZIP 15714 kb) [file 12859_2015_708_MOESM3_ESM.zip › Python_Examples/result_Example_3/Cholesterol Biosynthesis/backpage/L_16987.html]

 

# GeneProduct annotation

  

| Name: Lss| Identifier: 16987| Database: Entrez Gene| Synonyms: BC029082 | | | --- | --- | | | | --- | --- | --- | --- | | | | --- | --- | --- | --- | --- | --- | | |
| --- | --- | --- | --- | --- | --- | --- | --- |

# Expression data

**Gene id on mapp: 16987**

| Sample name 16987| logFC 2.60184848| Pvalue 0.778458319 | | | --- | --- | | | | --- | --- | --- | --- | | |
| --- | --- | --- | --- | --- | --- |

  
  

---

  
  

# Cross references

  

|
|  |
| **UniGene** |
| Mm.55075 |
|
| **Agilent** |
| A\_51\_P296487 |
| A\_52\_P118161 |
| A\_52\_P168028 |
|
| **Ensembl** |
| ENSMUSG00000033105 |
|
| **Illumina** |
| ILMN\_1228641 |
| ILMN\_2444540 |
| ILMN\_2614161 |
|
| **Entrez Gene** |
| 16987 |
|
| **MGI** |
| MGI:1336155 |
|
| **RefSeq** |
| NM\_146006 |
| NP\_666118 |
|
| **Uniprot/TrEMBL** |
| F7BJL0 |
| Q8BLN5 |
|
| **GeneOntology** |
| GO:0000250 |
| GO:0005789 |
| GO:0005811 |
| GO:0006694 |
|
| **UCSC Genome Browser** |
| uc007fuq.1 |
|
| **WikiGenes** |
| 16987 |
|
| **Affy** |
| 10364194 |
| 107541\_at |
| 136392\_at |
| 1420013\_s\_at |
| 1426913\_at |
| 160737\_at |
| aa670835\_at |
| aa670835\_g\_at |
