# Supplementary material for: Automatically visualise and analyse data on pathways using PathVisioRPC from any programming environment
Source: BMC Bioinformatics. 2015 Aug 23;16(1):267. doi: 10.1186/s12859-015-0708-8 (PMC4546821; doi:10.1186/s12859-015-0708-8)
Supplement: Additional file 3: — Examples in Python. This zip archive contains the data and python script for the three python examples. (ZIP 15714 kb) [file 12859_2015_708_MOESM3_ESM.zip › Python_Examples/result_Example_3/Cholesterol Biosynthesis/backpage/L_1717.html]

 

# GeneProduct annotation

  

| Name: DHCR7| Identifier: 1717| Database: Entrez Gene| Synonyms: SLOS | | | --- | --- | | | | --- | --- | --- | --- | | | | --- | --- | --- | --- | --- | --- | | |
| --- | --- | --- | --- | --- | --- | --- | --- |

# Expression data

**Gene id on mapp: 1717**

| Sample name 1717| logFC1 1.57486396| Pvalue1 0.0448699| logFC2 2.155661388| Pvalue2 0.009624315 | | | --- | --- | | | | --- | --- | --- | --- | | | | --- | --- | --- | --- | --- | --- | | | | --- | --- | --- | --- | --- | --- | --- | --- | | |
| --- | --- | --- | --- | --- | --- | --- | --- | --- | --- |

  
  

---

  
  

# Cross references

  

|
|  |
| **UniGene** |
| Hs.503134 |
| Hs.639004 |
|
| **Agilent** |
| A\_23\_P24444 |
|
| **Ensembl** |
| ENSG00000172893 |
|
| **Gene Wiki** |
| 1717 |
|
| **HGNC** |
| DHCR7 |
|
| **Illumina** |
| 0003990725 |
| ILMN\_1815626 |
| ILMN\_2165867 |
|
| **Entrez Gene** |
| 1717 |
|
| **OMIM** |
| 270400 |
| 602858 |
|
| **RefSeq** |
| NM\_001163817 |
| NM\_001360 |
| NP\_001157289 |
| NP\_001351 |
|
| **Uniprot/TrEMBL** |
| B4E1K5 |
| E9PIP9 |
| E9PJ54 |
| E9PLZ2 |
| E9PM00 |
| E9PQ71 |
| E9PRL8 |
| H0YE57 |
| H0YEJ5 |
| Q9UBM7 |
|
| **GeneOntology** |
| GO:0001568 |
| GO:0005640 |
| GO:0005783 |
| GO:0005789 |
| GO:0006695 |
| GO:0009791 |
| GO:0016020 |
| GO:0016021 |
| GO:0016126 |
| GO:0016628 |
| GO:0030154 |
| GO:0030324 |
| GO:0035264 |
| GO:0042127 |
| GO:0043231 |
| GO:0044281 |
| GO:0045540 |
| GO:0047598 |
|
| **UCSC Genome Browser** |
| uc001oqk.3 |
| uc001oql.3 |
|
| **WikiGenes** |
| 1717 |
|
| **Affy** |
| 11743808\_a\_at |
| 201791\_s\_at |
| 39059\_at |
| 7950067 |
