# Supplementary material for: Automatically visualise and analyse data on pathways using PathVisioRPC from any programming environment
Source: BMC Bioinformatics. 2015 Aug 23;16(1):267. doi: 10.1186/s12859-015-0708-8 (PMC4546821; doi:10.1186/s12859-015-0708-8)
Supplement: Additional file 3: — Examples in Python. This zip archive contains the data and python script for the three python examples. (ZIP 15714 kb) [file 12859_2015_708_MOESM3_ESM.zip › Python_Examples/result_Example_3/Cholesterol Biosynthesis/backpage/L_17855.html]

 

# GeneProduct annotation

  

| Name: Mvk| Identifier: 17855| Database: Entrez Gene| Synonyms: MK | | | --- | --- | | | | --- | --- | --- | --- | | | | --- | --- | --- | --- | --- | --- | | |
| --- | --- | --- | --- | --- | --- | --- | --- |

# Expression data

**Gene id on mapp: 17855**

| Sample name 17855| logFC -0.992632602| Pvalue 0.028739453 | | | --- | --- | | | | --- | --- | --- | --- | | |
| --- | --- | --- | --- | --- | --- |

  
  

---

  
  

# Cross references

  

|
|  |
| **UniGene** |
| Mm.28088 |
|
| **Agilent** |
| A\_51\_P169527 |
| A\_52\_P569067 |
| A\_55\_P2058861 |
| A\_55\_P2058864 |
|
| **Ensembl** |
| ENSMUSG00000041939 |
|
| **Illumina** |
| ILMN\_1239601 |
| ILMN\_1244594 |
|
| **Entrez Gene** |
| 17855 |
|
| **MGI** |
| MGI:107624 |
|
| **RefSeq** |
| NM\_023556 |
| NP\_076045 |
|
| **Uniprot/TrEMBL** |
| D3YV77 |
| Q3UEB4 |
| Q9R008 |
|
| **GeneOntology** |
| GO:0004496 |
| GO:0005524 |
| GO:0005737 |
| GO:0005829 |
| GO:0006695 |
| GO:0008299 |
| GO:0019287 |
| GO:0042802 |
| GO:0050728 |
|
| **UCSC Genome Browser** |
| uc008yzr.1 |
| uc008yzs.1 |
|
| **WikiGenes** |
| 17855 |
|
| **Affy** |
| 10524555 |
| 1418052\_at |
| 1430619\_a\_at |
| 95632\_f\_at |
| 95633\_r\_at |
| Msa.33048.0\_s\_at |
