# Supplementary material for: Automatically visualise and analyse data on pathways using PathVisioRPC from any programming environment
Source: BMC Bioinformatics. 2015 Aug 23;16(1):267. doi: 10.1186/s12859-015-0708-8 (PMC4546821; doi:10.1186/s12859-015-0708-8)
Supplement: Additional file 3: — Examples in Python. This zip archive contains the data and python script for the three python examples. (ZIP 15714 kb) [file 12859_2015_708_MOESM3_ESM.zip › Python_Examples/result_Example_3/Cholesterol Biosynthesis/backpage/L_18194.html]

 

# GeneProduct annotation

  

| Name: Nsdhl| Identifier: 18194| Database: Entrez Gene| Synonyms: Bpa | | | --- | --- | | | | --- | --- | --- | --- | | | | --- | --- | --- | --- | --- | --- | | |
| --- | --- | --- | --- | --- | --- | --- | --- |

# Expression data

**Gene id on mapp: 18194**

| Sample name 18194| logFC 2.372677242| Pvalue 0.611846621 | | | --- | --- | | | | --- | --- | --- | --- | | |
| --- | --- | --- | --- | --- | --- |

  
  

---

  
  

# Cross references

  

|
|  |
| **Agilent** |
| A\_51\_P326942 |
| A\_52\_P297803 |
| A\_55\_P1976395 |
| A\_66\_P137462 |
|
| **Ensembl** |
| ENSMUSG00000031349 |
|
| **Illumina** |
| ILMN\_2594521 |
| ILMN\_2594525 |
| ILMN\_2609273 |
| ILMN\_2725402 |
| ILMN\_2958207 |
|
| **Entrez Gene** |
| 18194 |
|
| **MGI** |
| MGI:1099438 |
|
| **RefSeq** |
| NM\_010941 |
| NP\_035071 |
|
| **Uniprot/TrEMBL** |
| Q3US15 |
| Q9R1J0 |
|
| **GeneOntology** |
| GO:0000166 |
| GO:0001942 |
| GO:0003854 |
| GO:0005783 |
| GO:0005811 |
| GO:0006695 |
| GO:0007224 |
| GO:0008203 |
| GO:0016021 |
| GO:0043231 |
| GO:0047012 |
| GO:0060716 |
|
| **UCSC Genome Browser** |
| uc009tkv.1 |
|
| **WikiGenes** |
| 18194 |
|
| **Affy** |
| 105991\_at |
| 10600082 |
| 1416222\_at |
| 168360\_f\_at |
| 93868\_at |
| 98630\_at |
| 98631\_g\_at |
