# Supplementary material for: Automatically visualise and analyse data on pathways using PathVisioRPC from any programming environment
Source: BMC Bioinformatics. 2015 Aug 23;16(1):267. doi: 10.1186/s12859-015-0708-8 (PMC4546821; doi:10.1186/s12859-015-0708-8)
Supplement: Additional file 3: — Examples in Python. This zip archive contains the data and python script for the three python examples. (ZIP 15714 kb) [file 12859_2015_708_MOESM3_ESM.zip › Python_Examples/result_Example_3/Cholesterol Biosynthesis/backpage/L_192156.html]

 

# GeneProduct annotation

  

| Name: Mvd| Identifier: 192156| Database: Entrez Gene| Synonyms: C78718 | | | --- | --- | | | | --- | --- | --- | --- | | | | --- | --- | --- | --- | --- | --- | | |
| --- | --- | --- | --- | --- | --- | --- | --- |

# Expression data

**Gene id on mapp: 192156**

| Sample name 192156| logFC 0.065002378| Pvalue 0.604680942 | | | --- | --- | | | | --- | --- | --- | --- | | |
| --- | --- | --- | --- | --- | --- |

  
  

---

  
  

# Cross references

  

|
|  |
| **Agilent** |
| A\_51\_P355943 |
|
| **Ensembl** |
| ENSMUSG00000006517 |
|
| **Illumina** |
| ILMN\_2645275 |
|
| **Entrez Gene** |
| 192156 |
|
| **MGI** |
| MGI:2179327 |
|
| **PDB** |
| 3F0N |
|
| **RefSeq** |
| NM\_138656 |
| NP\_619597 |
|
| **Uniprot/TrEMBL** |
| Q3UYC1 |
| Q99JF5 |
|
| **GeneOntology** |
| GO:0004163 |
| GO:0005524 |
| GO:0005829 |
| GO:0006695 |
| GO:0008284 |
| GO:0008299 |
| GO:0030544 |
| GO:0042803 |
|
| **UCSC Genome Browser** |
| uc009nss.2 |
|
| **WikiGenes** |
| 192156 |
|
| **Affy** |
| 10582310 |
| 1417303\_at |
| 1448663\_s\_at |
| 160770\_at |
| aa059528\_s\_at |
