# Supplementary material for: Automatically visualise and analyse data on pathways using PathVisioRPC from any programming environment
Source: BMC Bioinformatics. 2015 Aug 23;16(1):267. doi: 10.1186/s12859-015-0708-8 (PMC4546821; doi:10.1186/s12859-015-0708-8)
Supplement: Additional file 3: — Examples in Python. This zip archive contains the data and python script for the three python examples. (ZIP 15714 kb) [file 12859_2015_708_MOESM3_ESM.zip › Python_Examples/result_Example_3/Cholesterol Biosynthesis/backpage/L_20775.html]

 

# GeneProduct annotation

  

| Name: Sqle| Identifier: 20775| Database: Entrez Gene| Synonyms: AI323792 | | | --- | --- | | | | --- | --- | --- | --- | | | | --- | --- | --- | --- | --- | --- | | |
| --- | --- | --- | --- | --- | --- | --- | --- |

# Expression data

**Gene id on mapp: 20775**

| Sample name 20775| logFC 2.272476102| Pvalue 0.276951062 | | | --- | --- | | | | --- | --- | --- | --- | | |
| --- | --- | --- | --- | --- | --- |

  
  

---

  
  

# Cross references

  

|
|  |
| **UniGene** |
| Mm.296169 |
|
| **Agilent** |
| A\_51\_P450487 |
| A\_55\_P1975185 |
|
| **Ensembl** |
| ENSMUSG00000022351 |
|
| **Illumina** |
| ILMN\_2573600 |
| ILMN\_2600348 |
| ILMN\_2737163 |
|
| **Entrez Gene** |
| 20775 |
|
| **MGI** |
| MGI:109296 |
|
| **RefSeq** |
| NM\_009270 |
| NP\_033296 |
|
| **Uniprot/TrEMBL** |
| P52019 |
| Q3TQK8 |
| Q3TSS7 |
|
| **GeneOntology** |
| GO:0004506 |
| GO:0005789 |
| GO:0006725 |
| GO:0008203 |
| GO:0010033 |
| GO:0016021 |
| GO:0043231 |
| GO:0050660 |
|
| **UCSC Genome Browser** |
| uc007vxq.1 |
| uc007vxr.1 |
|
| **WikiGenes** |
| 20775 |
|
| **Affy** |
| 10424349 |
| 1415993\_at |
| 94322\_at |
| D42048\_s\_at |
