# Supplementary material for: Automatically visualise and analyse data on pathways using PathVisioRPC from any programming environment
Source: BMC Bioinformatics. 2015 Aug 23;16(1):267. doi: 10.1186/s12859-015-0708-8 (PMC4546821; doi:10.1186/s12859-015-0708-8)
Supplement: Additional file 3: — Examples in Python. This zip archive contains the data and python script for the three python examples. (ZIP 15714 kb) [file 12859_2015_708_MOESM3_ESM.zip › Python_Examples/result_Example_3/Cholesterol Biosynthesis/backpage/L_208715.html]

 

# GeneProduct annotation

  

| Name: Hmgcs1| Identifier: 208715| Database: Entrez Gene| Synonyms: B130032C06Rik | | | --- | --- | | | | --- | --- | --- | --- | | | | --- | --- | --- | --- | --- | --- | | |
| --- | --- | --- | --- | --- | --- | --- | --- |

# Expression data

**Gene id on mapp: 208715**

| Sample name 208715| logFC -0.152331943| Pvalue 0.016926324 | | | --- | --- | | | | --- | --- | --- | --- | | |
| --- | --- | --- | --- | --- | --- |

  
  

---

  
  

# Cross references

  

|
|  |
| **UniGene** |
| Mm.470344 |
| Mm.470503 |
| Mm.61526 |
|
| **Agilent** |
| A\_51\_P146941 |
| A\_52\_P388072 |
| A\_55\_P1954569 |
| A\_55\_P2032966 |
|
| **Ensembl** |
| ENSMUSG00000093930 |
|
| **Illumina** |
| ILMN\_2654952 |
|
| **Entrez Gene** |
| 208715 |
|
| **MGI** |
| MGI:107592 |
|
| **RefSeq** |
| NM\_145942 |
| NP\_666054 |
|
| **Uniprot/TrEMBL** |
| Q8JZK9 |
|
| **GeneOntology** |
| GO:0001101 |
| GO:0001889 |
| GO:0004421 |
| GO:0005634 |
| GO:0005737 |
| GO:0005829 |
| GO:0005886 |
| GO:0006695 |
| GO:0007420 |
| GO:0008144 |
| GO:0008299 |
| GO:0008584 |
| GO:0009645 |
| GO:0009725 |
| GO:0010243 |
| GO:0014070 |
| GO:0014074 |
| GO:0016853 |
| GO:0033197 |
| GO:0034698 |
| GO:0042493 |
| GO:0042803 |
| GO:0043177 |
| GO:0046690 |
| GO:0055094 |
| GO:0070723 |
| GO:0071372 |
| GO:0071397 |
| GO:0071407 |
|
| **UCSC Genome Browser** |
| uc007rzw.1 |
|
| **WikiGenes** |
| 208715 |
|
| **Affy** |
| 10412466 |
| 1433443\_a\_at |
| 1433444\_at |
| 1433445\_x\_at |
| 1433446\_at |
| 94325\_at |
| aa275198\_i\_at |
| aa275198\_s\_at |
