# Supplementary material for: Automatically visualise and analyse data on pathways using PathVisioRPC from any programming environment
Source: BMC Bioinformatics. 2015 Aug 23;16(1):267. doi: 10.1186/s12859-015-0708-8 (PMC4546821; doi:10.1186/s12859-015-0708-8)
Supplement: Additional file 3: — Examples in Python. This zip archive contains the data and python script for the three python examples. (ZIP 15714 kb) [file 12859_2015_708_MOESM3_ESM.zip › Python_Examples/result_Example_3/Cholesterol Biosynthesis/backpage/L_2222.html]

 

# GeneProduct annotation

  

| Name: FDFT1| Identifier: 2222| Database: Entrez Gene| Synonyms: DGPT | | | --- | --- | | | | --- | --- | --- | --- | | | | --- | --- | --- | --- | --- | --- | | |
| --- | --- | --- | --- | --- | --- | --- | --- |

# Expression data

**Gene id on mapp: 2222**

| Sample name 2222| logFC1 2.041001419| Pvalue1 0.795197626| logFC2 2.328053683| Pvalue2 0.741041158 | | | --- | --- | | | | --- | --- | --- | --- | | | | --- | --- | --- | --- | --- | --- | | | | --- | --- | --- | --- | --- | --- | --- | --- | | |
| --- | --- | --- | --- | --- | --- | --- | --- | --- | --- |

  
  

---

  
  

# Cross references

  

|
|  |
| **UniGene** |
| Hs.733088 |
| Hs.745428 |
|
| **Agilent** |
| A\_14\_P129904 |
| A\_23\_P71319 |
|
| **Ensembl** |
| ENSG00000079459 |
|
| **Gene Wiki** |
| 2222 |
|
| **HGNC** |
| FDFT1 |
|
| **Illumina** |
| 0006110167 |
| ILMN\_1741096 |
| ILMN\_2144088 |
|
| **Entrez Gene** |
| 2222 |
|
| **OMIM** |
| 184420 |
|
| **PDB** |
| 1EZF |
| 3ASX |
| 3LEE |
| 3Q2Z |
| 3Q30 |
| 3V66 |
| 3VJ8 |
| 3VJ9 |
| 3VJA |
| 3VJB |
| 3VJC |
|
| **RefSeq** |
| NM\_004462 |
| NP\_004453 |
|
| **Uniprot/TrEMBL** |
| B3KQ95 |
| B4DJE5 |
| B4DT56 |
| B7Z1J3 |
| E9PJG4 |
| E9PNJ2 |
| E9PNM1 |
| E9PQ90 |
| E9PS69 |
| E9PSH1 |
| P37268 |
| Q6IAX1 |
|
| **GeneOntology** |
| GO:0004310 |
| GO:0005783 |
| GO:0005789 |
| GO:0006694 |
| GO:0006695 |
| GO:0008299 |
| GO:0008610 |
| GO:0009058 |
| GO:0016021 |
| GO:0016491 |
| GO:0016740 |
| GO:0043231 |
| GO:0044255 |
| GO:0044281 |
| GO:0045338 |
| GO:0051996 |
|
| **UCSC Genome Browser** |
| uc003wuh.3 |
| uc003wui.3 |
| uc010lsb.3 |
| uc011kxf.2 |
| uc011kxi.2 |
| uc011kxj.2 |
| uc011kxk.2 |
| uc022ary.1 |
|
| **WikiGenes** |
| 2222 |
|
| **Affy** |
| 11721242\_s\_at |
| 11745322\_x\_at |
| 11748274\_s\_at |
| 11748275\_x\_at |
| 11748801\_s\_at |
| 11754259\_x\_at |
| 210950\_s\_at |
| 34848\_at |
| 57523\_at |
| 63122\_at |
| 8144669 |
| X69141\_at |
