# Supplementary material for: Automatically visualise and analyse data on pathways using PathVisioRPC from any programming environment
Source: BMC Bioinformatics. 2015 Aug 23;16(1):267. doi: 10.1186/s12859-015-0708-8 (PMC4546821; doi:10.1186/s12859-015-0708-8)
Supplement: Additional file 3: — Examples in Python. This zip archive contains the data and python script for the three python examples. (ZIP 15714 kb) [file 12859_2015_708_MOESM3_ESM.zip › Python_Examples/result_Example_3/Cholesterol Biosynthesis/backpage/L_2224.html]

 

# GeneProduct annotation

  

| Name: FDPS| Identifier: 2224| Database: Entrez Gene| Synonyms: FPS | | | --- | --- | | | | --- | --- | --- | --- | | | | --- | --- | --- | --- | --- | --- | | |
| --- | --- | --- | --- | --- | --- | --- | --- |

# Expression data

**Gene id on mapp: 2224**

| Sample name 2224 2224| logFC1 2.803830576 1.660911515| Pvalue1 0.049858682 0.377594176| logFC2 3.007603228 1.586854914| Pvalue2 0.008435299 0.319473648 | | | | --- | --- | --- | | | | | --- | --- | --- | --- | --- | --- | | | | | --- | --- | --- | --- | --- | --- | --- | --- | --- | | | | | --- | --- | --- | --- | --- | --- | --- | --- | --- | --- | --- | --- | | | |
| --- | --- | --- | --- | --- | --- | --- | --- | --- | --- | --- | --- | --- | --- | --- |

  
  

---

  
  

# Cross references

  

|
|  |
| **Agilent** |
| A\_14\_P118865 |
| A\_14\_P200040 |
| A\_24\_P114183 |
|
| **Ensembl** |
| ENSG00000160752 |
|
| **Gene Wiki** |
| 2224 |
|
| **HGNC** |
| FDPS |
|
| **Illumina** |
| 0000130731 |
| ILMN\_1804248 |
|
| **Entrez Gene** |
| 2224 |
|
| **OMIM** |
| 134629 |
|
| **PDB** |
| 1YQ7 |
| 1YV5 |
| 1ZW5 |
| 2F7M |
| 2F89 |
| 2F8C |
| 2F8Z |
| 2F92 |
| 2F94 |
| 2F9K |
| 2OPM |
| 2OPN |
| 2QIS |
| 2RAH |
| 2VF6 |
| 3B7L |
| 3CP6 |
| 3N1V |
| 3N1W |
| 3N3L |
| 3N45 |
| 3N46 |
| 3N49 |
| 3N5H |
| 3N5J |
| 3N6K |
| 3RYE |
| 3S4J |
| 4DEM |
| 4H5C |
| 4H5D |
| 4H5E |
|
| **RefSeq** |
| NM\_001135821 |
| NM\_001135822 |
| NM\_001242824 |
| NM\_001242825 |
| NM\_002004 |
| NP\_001129293 |
| NP\_001129294 |
| NP\_001229753 |
| NP\_001229754 |
| NP\_001995 |
|
| **Uniprot/TrEMBL** |
| E9PCI9 |
| P14324 |
|
| **GeneOntology** |
| GO:0004161 |
| GO:0004337 |
| GO:0005634 |
| GO:0005737 |
| GO:0005739 |
| GO:0005829 |
| GO:0006695 |
| GO:0008299 |
| GO:0016740 |
| GO:0019048 |
| GO:0033384 |
| GO:0044281 |
| GO:0045337 |
| GO:0046872 |
|
| **UCSC Genome Browser** |
| uc001fkc.2 |
| uc001fkd.2 |
| uc001fke.2 |
|
| **WikiGenes** |
| 2224 |
|
| **Affy** |
| 11718366\_s\_at |
| 11758249\_s\_at |
| 201275\_at |
| 37325\_at |
| 7905986 |
| Z47055\_s\_at |
