# Supplementary material for: Automatically visualise and analyse data on pathways using PathVisioRPC from any programming environment
Source: BMC Bioinformatics. 2015 Aug 23;16(1):267. doi: 10.1186/s12859-015-0708-8 (PMC4546821; doi:10.1186/s12859-015-0708-8)
Supplement: Additional file 3: — Examples in Python. This zip archive contains the data and python script for the three python examples. (ZIP 15714 kb) [file 12859_2015_708_MOESM3_ESM.zip › Python_Examples/result_Example_3/Cholesterol Biosynthesis/backpage/L_235293.html]

 

# GeneProduct annotation

  

| Name: Sc5d| Identifier: 235293| Database: Entrez Gene| Synonyms: A830037K02 | | | --- | --- | | | | --- | --- | --- | --- | | | | --- | --- | --- | --- | --- | --- | | |
| --- | --- | --- | --- | --- | --- | --- | --- |

# Expression data

**Gene id on mapp: 235293**

| Sample name 235293| logFC 2.150843562| Pvalue 0.036089345 | | | --- | --- | | | | --- | --- | --- | --- | | |
| --- | --- | --- | --- | --- | --- |

  
  

---

  
  

# Cross references

  

|
|  |
| **UniGene** |
| Mm.32700 |
|
| **Agilent** |
| A\_51\_P418056 |
|
| **Ensembl** |
| ENSMUSG00000032018 |
|
| **Illumina** |
| ILMN\_1231026 |
|
| **Entrez Gene** |
| 235293 |
|
| **MGI** |
| MGI:1353611 |
|
| **RefSeq** |
| NM\_172769 |
| NP\_766357 |
|
| **Uniprot/TrEMBL** |
| Q8BGI0 |
|
| **GeneOntology** |
| GO:0000248 |
| GO:0005506 |
| GO:0006633 |
| GO:0016021 |
| GO:0033490 |
| GO:0043231 |
|
| **UCSC Genome Browser** |
| uc009par.1 |
| uc009pas.2 |
|
| **WikiGenes** |
| 235293 |
|
| **Affy** |
| 102768\_i\_at |
| 102769\_f\_at |
| 10592585 |
| 1424709\_at |
| 1451457\_at |
