# Supplementary material for: Automatically visualise and analyse data on pathways using PathVisioRPC from any programming environment
Source: BMC Bioinformatics. 2015 Aug 23;16(1):267. doi: 10.1186/s12859-015-0708-8 (PMC4546821; doi:10.1186/s12859-015-0708-8)
Supplement: Additional file 3: — Examples in Python. This zip archive contains the data and python script for the three python examples. (ZIP 15714 kb) [file 12859_2015_708_MOESM3_ESM.zip › Python_Examples/result_Example_3/Cholesterol Biosynthesis/backpage/L_3156.html]

 

# GeneProduct annotation

  

| Name: HMGCR| Identifier: 3156| Database: Entrez Gene| Synonyms: LDLCQ3 | | | --- | --- | | | | --- | --- | --- | --- | | | | --- | --- | --- | --- | --- | --- | | |
| --- | --- | --- | --- | --- | --- | --- | --- |

# Expression data

**Gene id on mapp: 3156**

| Sample name 3156| logFC1 -0.372265331| Pvalue1 0.014070437| logFC2 -0.27207029| Pvalue2 0.012034666 | | | --- | --- | | | | --- | --- | --- | --- | | | | --- | --- | --- | --- | --- | --- | | | | --- | --- | --- | --- | --- | --- | --- | --- | | |
| --- | --- | --- | --- | --- | --- | --- | --- | --- | --- |

  
  

---

  
  

# Cross references

  

|
|  |
| **UniGene** |
| Hs.628096 |
|
| **Agilent** |
| A\_14\_P126678 |
| A\_14\_P201160 |
| A\_23\_P30495 |
|
| **Ensembl** |
| ENSG00000113161 |
|
| **Gene Wiki** |
| 3156 |
|
| **HGNC** |
| HMGCR |
|
| **Illumina** |
| 0005900324 |
|
| **Entrez Gene** |
| 3156 |
|
| **OMIM** |
| 142910 |
|
| **PDB** |
| 1DQ8 |
| 1DQ9 |
| 1DQA |
| 1HW8 |
| 1HW9 |
| 1HWI |
| 1HWJ |
| 1HWK |
| 1HWL |
| 2Q1L |
| 2Q6B |
| 2Q6C |
| 2R4F |
| 3BGL |
| 3CCT |
| 3CCW |
| 3CCZ |
| 3CD0 |
| 3CD5 |
| 3CD7 |
| 3CDA |
| 3CDB |
|
| **RefSeq** |
| NM\_000859 |
| NM\_001130996 |
| NP\_000850 |
| NP\_001124468 |
|
| **Uniprot/TrEMBL** |
| C9JKX7 |
| D6RIW0 |
| H0Y8F6 |
| P04035 |
|
| **GeneOntology** |
| GO:0004420 |
| GO:0005778 |
| GO:0005783 |
| GO:0005789 |
| GO:0006695 |
| GO:0006743 |
| GO:0007568 |
| GO:0007584 |
| GO:0008158 |
| GO:0008284 |
| GO:0008299 |
| GO:0008542 |
| GO:0009790 |
| GO:0010664 |
| GO:0010666 |
| GO:0015936 |
| GO:0016020 |
| GO:0016021 |
| GO:0016616 |
| GO:0032874 |
| GO:0042282 |
| GO:0042803 |
| GO:0043066 |
| GO:0043231 |
| GO:0043407 |
| GO:0044255 |
| GO:0044281 |
| GO:0045445 |
| GO:0045471 |
| GO:0045908 |
| GO:0048643 |
| GO:0048661 |
| GO:0050661 |
| GO:0050662 |
| GO:0051262 |
| GO:0055114 |
| GO:0061045 |
| GO:0061179 |
| GO:0070374 |
| GO:0070402 |
|
| **UCSC Genome Browser** |
| uc003kdp.3 |
| uc003kdq.3 |
| uc010izo.3 |
| uc010izp.3 |
| uc011cst.2 |
|
| **WikiGenes** |
| 3156 |
|
| **Affy** |
| 11727374\_s\_at |
| 11727375\_a\_at |
| 11727376\_a\_at |
| 202539\_s\_at |
| 39328\_at |
| 8106280 |
| M11058\_at |
