# Supplementary material for: Automatically visualise and analyse data on pathways using PathVisioRPC from any programming environment
Source: BMC Bioinformatics. 2015 Aug 23;16(1):267. doi: 10.1186/s12859-015-0708-8 (PMC4546821; doi:10.1186/s12859-015-0708-8)
Supplement: Additional file 3: — Examples in Python. This zip archive contains the data and python script for the three python examples. (ZIP 15714 kb) [file 12859_2015_708_MOESM3_ESM.zip › Python_Examples/result_Example_3/Cholesterol Biosynthesis/backpage/L_3157.html]

 

# GeneProduct annotation

  

| Name: HMGCS1| Identifier: 3157| Database: Entrez Gene| Synonyms: HMGCS | | | --- | --- | | | | --- | --- | --- | --- | | | | --- | --- | --- | --- | --- | --- | | |
| --- | --- | --- | --- | --- | --- | --- | --- |

# Expression data

**Gene id on mapp: 3157**

| Sample name 3157| logFC1 -0.323824977| Pvalue1 0.355396581| logFC2 0.101520432| Pvalue2 0.259833209 | | | --- | --- | | | | --- | --- | --- | --- | | | | --- | --- | --- | --- | --- | --- | | | | --- | --- | --- | --- | --- | --- | --- | --- | | |
| --- | --- | --- | --- | --- | --- | --- | --- | --- | --- |

  
  

---

  
  

# Cross references

  

|
|  |
| **UniGene** |
| Hs.397729 |
| Hs.602526 |
|
| **Agilent** |
| A\_23\_P133263 |
| A\_24\_P63522 |
|
| **Ensembl** |
| ENSG00000112972 |
|
| **Gene Wiki** |
| 3157 |
|
| **HGNC** |
| HMGCS1 |
|
| **Illumina** |
| 0001690433 |
| ILMN\_1797728 |
|
| **Entrez Gene** |
| 3157 |
|
| **OMIM** |
| 142940 |
|
| **PDB** |
| 2P8U |
|
| **RefSeq** |
| NM\_001098272 |
| NM\_002130 |
| NP\_001091742 |
| NP\_002121 |
|
| **Uniprot/TrEMBL** |
| D6RIW1 |
| Q01581 |
| Q8N995 |
|
| **GeneOntology** |
| GO:0001101 |
| GO:0001889 |
| GO:0003824 |
| GO:0004421 |
| GO:0005634 |
| GO:0005737 |
| GO:0005829 |
| GO:0005886 |
| GO:0006629 |
| GO:0006695 |
| GO:0007420 |
| GO:0008144 |
| GO:0008152 |
| GO:0008299 |
| GO:0008584 |
| GO:0009645 |
| GO:0009725 |
| GO:0010243 |
| GO:0014070 |
| GO:0014074 |
| GO:0016853 |
| GO:0033197 |
| GO:0034698 |
| GO:0042493 |
| GO:0042803 |
| GO:0043177 |
| GO:0044255 |
| GO:0044281 |
| GO:0046690 |
| GO:0055094 |
| GO:0070723 |
| GO:0071372 |
| GO:0071397 |
| GO:0071407 |
|
| **UCSC Genome Browser** |
| uc003jnq.4 |
| uc003jnr.4 |
|
| **WikiGenes** |
| 3157 |
|
| **Affy** |
| 11716987\_a\_at |
| 11754918\_s\_at |
| 221750\_at |
| 34517\_at |
| 54968\_at |
| 78228\_s\_at |
| 8111941 |
| 89646\_r\_at |
| L25798\_at |
