# Supplementary material for: Automatically visualise and analyse data on pathways using PathVisioRPC from any programming environment
Source: BMC Bioinformatics. 2015 Aug 23;16(1):267. doi: 10.1186/s12859-015-0708-8 (PMC4546821; doi:10.1186/s12859-015-0708-8)
Supplement: Additional file 3: — Examples in Python. This zip archive contains the data and python script for the three python examples. (ZIP 15714 kb) [file 12859_2015_708_MOESM3_ESM.zip › Python_Examples/result_Example_3/Cholesterol Biosynthesis/backpage/L_319554.html]

 

# GeneProduct annotation

  

| Name: Idi1| Identifier: 319554| Database: Entrez Gene| Synonyms: 4832416K17Rik | | | --- | --- | | | | --- | --- | --- | --- | | | | --- | --- | --- | --- | --- | --- | | |
| --- | --- | --- | --- | --- | --- | --- | --- |

# Expression data

**Gene id on mapp: 319554**

| Sample name 319554| logFC 0.595289997| Pvalue 0.177755094 | | | --- | --- | | | | --- | --- | --- | --- | | |
| --- | --- | --- | --- | --- | --- |

  
  

---

  
  

# Cross references

  

|
|  |
| **UniGene** |
| Mm.29847 |
| Mm.398604 |
|
| **Agilent** |
| A\_51\_P329711 |
| A\_52\_P441634 |
| A\_55\_P2028961 |
| A\_55\_P2068184 |
|
| **Ensembl** |
| ENSMUSG00000058258 |
|
| **Illumina** |
| ILMN\_2590923 |
|
| **Entrez Gene** |
| 319554 |
|
| **MGI** |
| MGI:2442264 |
|
| **RefSeq** |
| NM\_145360 |
| NP\_663335 |
|
| **Uniprot/TrEMBL** |
| G3XA48 |
| P58044 |
|
| **GeneOntology** |
| GO:0000287 |
| GO:0004452 |
| GO:0005739 |
| GO:0005777 |
| GO:0006695 |
| GO:0008299 |
| GO:0016787 |
| GO:0030145 |
| GO:0035634 |
| GO:0050992 |
|
| **UCSC Genome Browser** |
| uc007pkn.2 |
|
| **WikiGenes** |
| 319554 |
|
| **Affy** |
| 10403413 |
| 10482762 |
| 1423804\_a\_at |
| 1451122\_at |
| 164172\_at |
| 96269\_at |
| aa267683\_s\_at |
