# Supplementary material for: Automatically visualise and analyse data on pathways using PathVisioRPC from any programming environment
Source: BMC Bioinformatics. 2015 Aug 23;16(1):267. doi: 10.1186/s12859-015-0708-8 (PMC4546821; doi:10.1186/s12859-015-0708-8)
Supplement: Additional file 5: Figure S1. — Box plots showing array quality control and normalisation results. Box plots showing array quality control and normalization results using different normalization methods (BGcorrected, Loess, Loess + Scale, Loess + Quantile). (PDF 424 kb) [file 12859_2015_708_MOESM5_ESM.pdf]

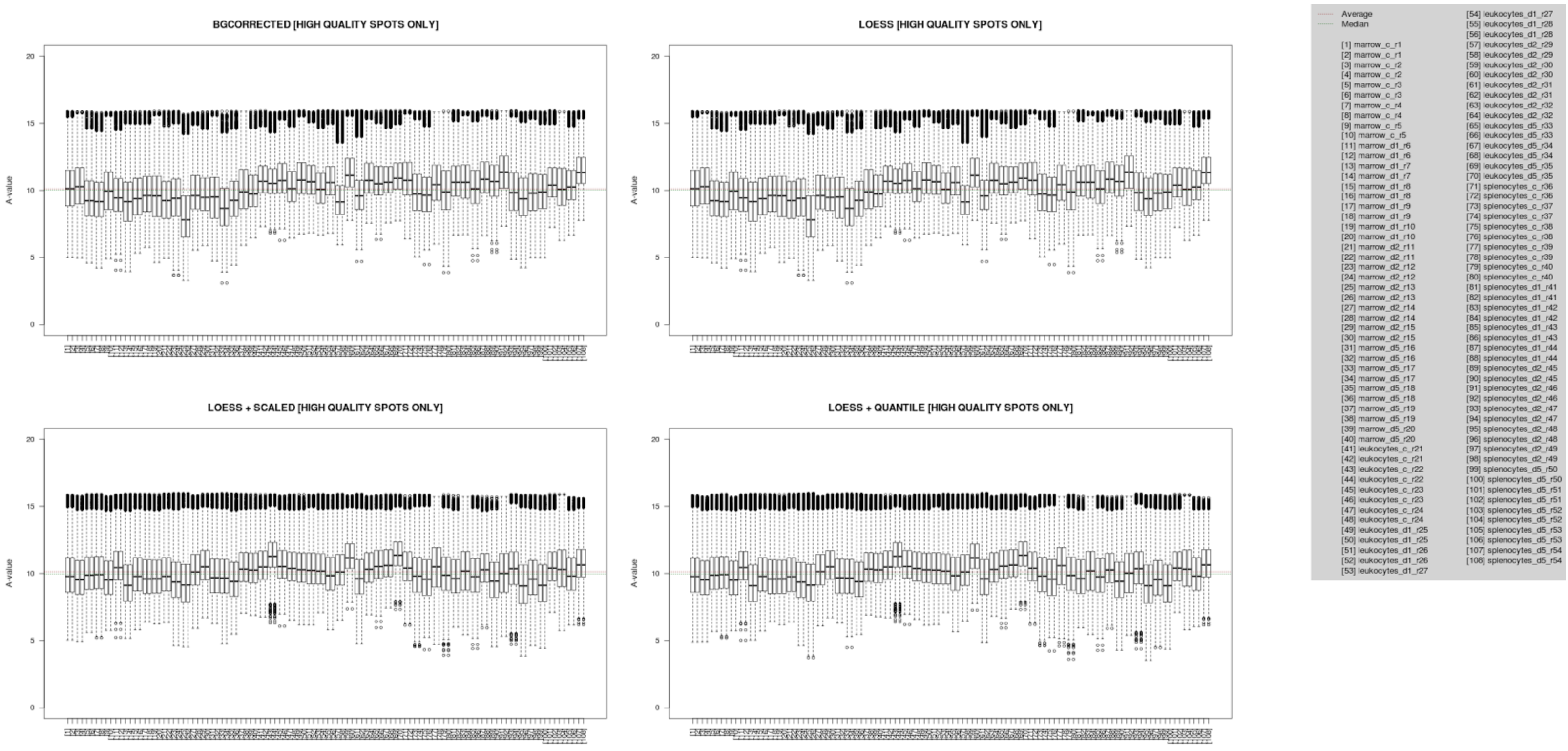

Supplemental Fig. 1: Box plots showing array quality control and normalization results using different normalization methods (BGcorrected, Loess, Loess + Scale, Loess + Quantile). The normalized data table and flags data table are available at http://projects.bigcat.unimaas.nl/data/pathvisiorpc/data/arrayQC_normData.table and http://projects.bigcat.unimaas.nl/data/pathvisiorpc/data/arayQC_flags.table respectively.
