# Supplementary material for: Automatically visualise and analyse data on pathways using PathVisioRPC from any programming environment
Source: BMC Bioinformatics. 2015 Aug 23;16(1):267. doi: 10.1186/s12859-015-0708-8 (PMC4546821; doi:10.1186/s12859-015-0708-8)
Supplement: Additional file 8: Figure S2. — Oxidative stress pathway [52] for splenocytes showing the logFC and P-value for day 1, day 2 and day 5. (PDF 104 kb) [file 12859_2015_708_MOESM8_ESM.pdf]

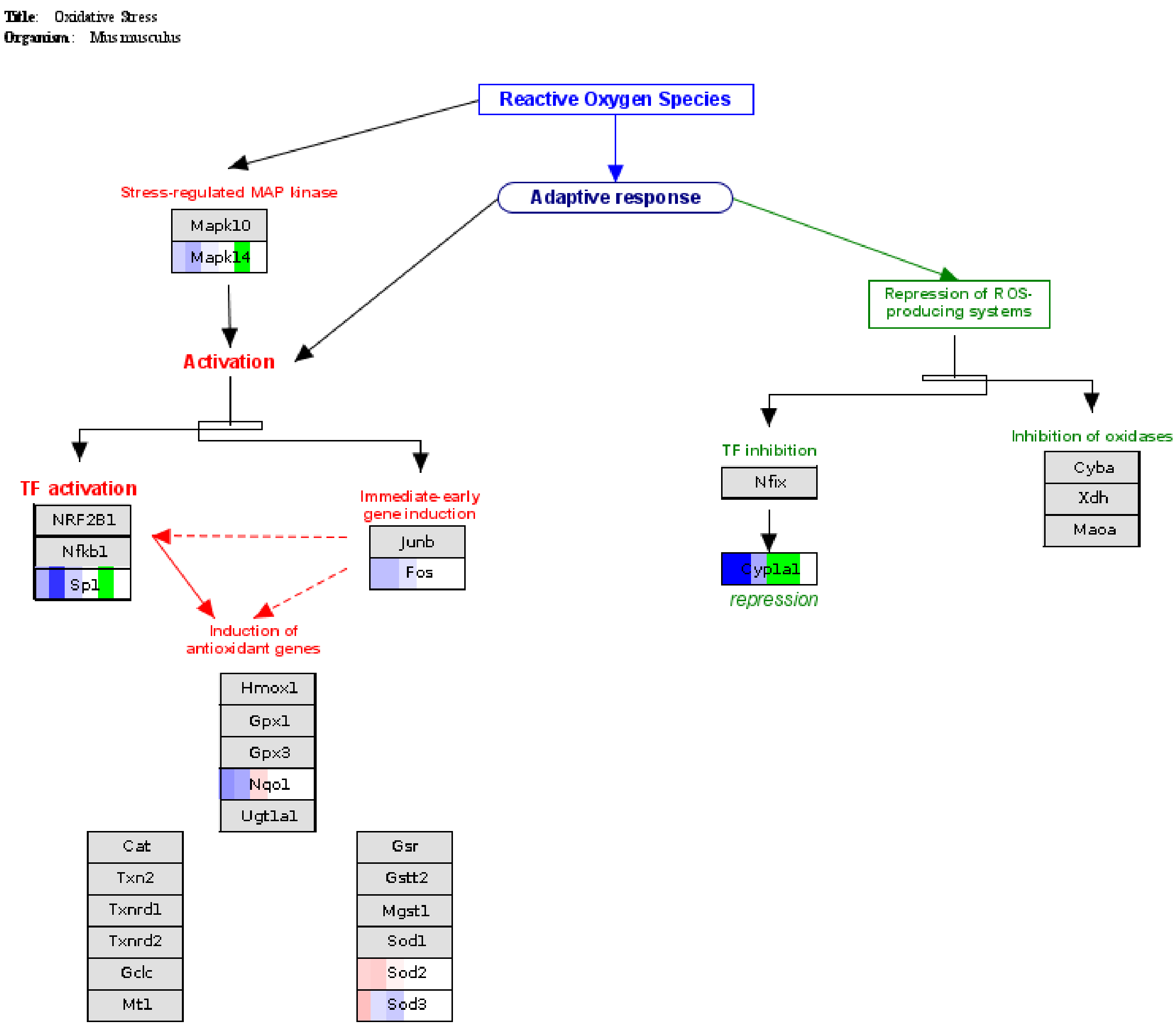

Supplemental Fig. 2: *Oxidative Stress Pathway* (WP412) in Splenocytes after cyclophosphamide treatment *vs.* control comparison (*Mus musculus*). *Oxidative Stress Pathway* ranks in 7th position of relevant pathways with a z-score of 2.08 at 1 day of treatment, making it a highly-relevant pathway in the experimental conditions.
