# Supplementary material for: Automatically visualise and analyse data on pathways using PathVisioRPC from any programming environment
Source: BMC Bioinformatics. 2015 Aug 23;16(1):267. doi: 10.1186/s12859-015-0708-8 (PMC4546821; doi:10.1186/s12859-015-0708-8)
Supplement: Additional file 9: Figure S3. — Oxidative stress pathway [52] for bone marrow cells showing the logFC and P-value for day 1, day 2, and day 5. (PDF 105 kb) [file 12859_2015_708_MOESM9_ESM.pdf]

Title: Oxidative Stress
Organism: Mus musculus

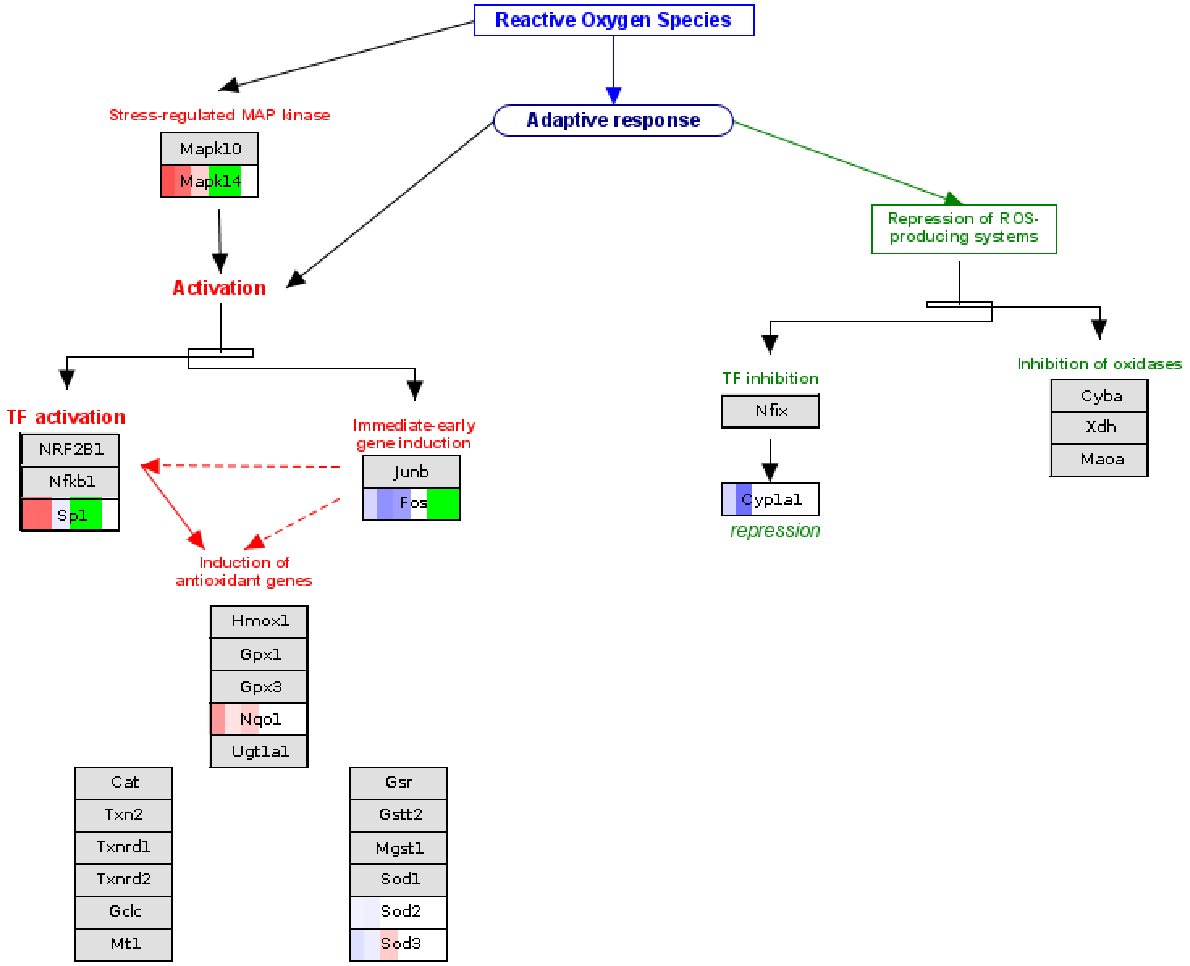

Supplemental Fig. 3: *Oxidative Stress Pathway* (WP412) in Bone Marrow cells after cyclophosphamide treatment *vs.* control comparison (*Mus musculus*). *Oxidative Stress Pathway* ranks in 7th position of relevant pathways with a z-score of 2.08 at 1 day of treatment, making it a highly-relevant pathway in the experimental conditions.
