# Supplementary material for: Automatically visualise and analyse data on pathways using PathVisioRPC from any programming environment
Source: BMC Bioinformatics. 2015 Aug 23;16(1):267. doi: 10.1186/s12859-015-0708-8 (PMC4546821; doi:10.1186/s12859-015-0708-8)
Supplement: Additional file 3: — Examples in Python. This zip archive contains the data and python script for the three python examples. (ZIP 15714 kb) [file 12859_2015_708_MOESM3_ESM.zip › Python_Examples/result_Example_3/Cholesterol Biosynthesis/backpage/L_3422.html]

 

# GeneProduct annotation

  

| Name: IDI1| Identifier: 3422| Database: Entrez Gene| Synonyms: IPP1 | | | --- | --- | | | | --- | --- | --- | --- | | | | --- | --- | --- | --- | --- | --- | | |
| --- | --- | --- | --- | --- | --- | --- | --- |

# Expression data

**Gene id on mapp: 3422**

| Sample name 3422| logFC1 0.317827493| Pvalue1 0.096527947| logFC2 1.000568495| Pvalue2 0.095472667 | | | --- | --- | | | | --- | --- | --- | --- | | | | --- | --- | --- | --- | --- | --- | | | | --- | --- | --- | --- | --- | --- | --- | --- | | |
| --- | --- | --- | --- | --- | --- | --- | --- | --- | --- |

  
  

---

  
  

# Cross references

  

|  |
| --- |
|  |
| **Agilent** |
| A\_24\_P103886 |
|  |
| **Ensembl** |
| ENSG00000067064 |
|  |
| **Gene Wiki** |
| 3422 |
|  |
| **HGNC** |
| IDI1 |
|  |
| **Illumina** |
| 0006220064 |
| ILMN\_1755075 |
|  |
| **Entrez Gene** |
| 3422 |
|  |
| **OMIM** |
| 604055 |
|  |
| **PDB** |
| 2DHO |
| 2I6K |
| 2ICJ |
| 2ICK |
|  |
| **RefSeq** |
| NM\_004508 |
| NP\_004499 |
|  |
| **Uniprot/TrEMBL** |
| C9JD53 |
| C9JKM8 |
| Q13907 |
|  |
| **GeneOntology** |
| GO:0000287 |
| GO:0004452 |
| GO:0005739 |
| GO:0005777 |
| GO:0005829 |
| GO:0006695 |
| GO:0008299 |
| GO:0016787 |
| GO:0030145 |
| GO:0035634 |
| GO:0044281 |
| GO:0046872 |
| GO:0050992 |
|  |
| **UCSC Genome Browser** |
| uc001ifz.3 |
| uc001iga.3 |
| uc001igb.3 |
|  |
| **WikiGenes** |
| 3422 |
|  |
| **Affy** |
| 11739383\_a\_at |
| 11744474\_s\_at |
| 208881\_x\_at |
| 36985\_at |
| 54619\_at |
| 7931754 |
| X17025\_at |
