# Supplementary material for: Automatically visualise and analyse data on pathways using PathVisioRPC from any programming environment
Source: BMC Bioinformatics. 2015 Aug 23;16(1):267. doi: 10.1186/s12859-015-0708-8 (PMC4546821; doi:10.1186/s12859-015-0708-8)
Supplement: Additional file 3: — Examples in Python. This zip archive contains the data and python script for the three python examples. (ZIP 15714 kb) [file 12859_2015_708_MOESM3_ESM.zip › Python_Examples/result_Example_3/Cholesterol Biosynthesis/backpage/L_4047.html]

 

# GeneProduct annotation

  

| Name: LSS| Identifier: 4047| Database: Entrez Gene| Synonyms: OSC | | | --- | --- | | | | --- | --- | --- | --- | | | | --- | --- | --- | --- | --- | --- | | |
| --- | --- | --- | --- | --- | --- | --- | --- |

# Expression data

**Gene id on mapp: 4047**

| Sample name 4047| logFC1 2.51638984| Pvalue1 0.81855573| logFC2 2.517321162| Pvalue2 0.73316153 | | | --- | --- | | | | --- | --- | --- | --- | | | | --- | --- | --- | --- | --- | --- | | | | --- | --- | --- | --- | --- | --- | --- | --- | | |
| --- | --- | --- | --- | --- | --- | --- | --- | --- | --- |

  
  

---

  
  

# Cross references

  

|  |
| --- |
|  |
| **UniGene** |
| Hs.596543 |
| Hs.734274 |
|  |
| **Agilent** |
| A\_23\_P211252 |
| A\_24\_P110799 |
| A\_24\_P162211 |
| A\_24\_P384839 |
|  |
| **Ensembl** |
| ENSG00000160285 |
|  |
| **Gene Wiki** |
| 4047 |
|  |
| **HGNC** |
| LSS |
|  |
| **Illumina** |
| 0000870017 |
| ILMN\_1658271 |
| ILMN\_1687863 |
| ILMN\_1715024 |
| ILMN\_1753269 |
|  |
| **Entrez Gene** |
| 4047 |
|  |
| **OMIM** |
| 600909 |
|  |
| **PDB** |
| 1W6J |
| 1W6K |
|  |
| **RefSeq** |
| NM\_001001438 |
| NM\_001145436 |
| NM\_001145437 |
| NM\_002340 |
| NP\_001001438 |
| NP\_001138908 |
| NP\_001138909 |
| NP\_002331 |
|  |
| **Uniprot/TrEMBL** |
| C9J315 |
| P48449 |
|  |
| **GeneOntology** |
| GO:0000250 |
| GO:0003824 |
| GO:0005789 |
| GO:0005811 |
| GO:0006695 |
| GO:0008202 |
| GO:0016866 |
| GO:0044281 |
|  |
| **UCSC Genome Browser** |
| uc002zij.3 |
| uc002zik.2 |
| uc002zil.2 |
| uc011afv.1 |
|  |
| **WikiGenes** |
| 4047 |
|  |
| **Affy** |
| 11717620\_a\_at |
| 11724199\_x\_at |
| 11731324\_a\_at |
| 202245\_at |
| 39107\_at |
| 39108\_at |
| 8070961 |
| S81221\_at |
| U22526\_at |
