# Supplementary material for: Automatically visualise and analyse data on pathways using PathVisioRPC from any programming environment
Source: BMC Bioinformatics. 2015 Aug 23;16(1):267. doi: 10.1186/s12859-015-0708-8 (PMC4546821; doi:10.1186/s12859-015-0708-8)
Supplement: Additional file 3: — Examples in Python. This zip archive contains the data and python script for the three python examples. (ZIP 15714 kb) [file 12859_2015_708_MOESM3_ESM.zip › Python_Examples/result_Example_3/Cholesterol Biosynthesis/backpage/L_4597.html]

 

# GeneProduct annotation

  

| Name: MVD| Identifier: 4597| Database: Entrez Gene| Synonyms: MPD | | | --- | --- | | | | --- | --- | --- | --- | | | | --- | --- | --- | --- | --- | --- | | |
| --- | --- | --- | --- | --- | --- | --- | --- |

# Expression data

**Gene id on mapp: 4597**

| Sample name 4597| logFC1 0.893993224| Pvalue1 0.107576602| logFC2 1.081102643| Pvalue2 0.020682577 | | | --- | --- | | | | --- | --- | --- | --- | | | | --- | --- | --- | --- | --- | --- | | | | --- | --- | --- | --- | --- | --- | --- | --- | | |
| --- | --- | --- | --- | --- | --- | --- | --- | --- | --- |

  
  

---

  
  

# Cross references

  

|  |
| --- |
|  |
| **UniGene** |
| Hs.252457 |
|  |
| **Agilent** |
| A\_23\_P152125 |
|  |
| **Ensembl** |
| ENSG00000167508 |
|  |
| **Gene Wiki** |
| 4597 |
|  |
| **HGNC** |
| MVD |
|  |
| **Illumina** |
| 0106840497 |
| ILMN\_1657550 |
|  |
| **Entrez Gene** |
| 4597 |
|  |
| **OMIM** |
| 603236 |
|  |
| **PDB** |
| 3D4J |
|  |
| **RefSeq** |
| NM\_002461 |
| NP\_002452 |
|  |
| **Uniprot/TrEMBL** |
| H3BP35 |
| H3BQ47 |
| H3BRZ1 |
| H3BVC4 |
| P53602 |
|  |
| **GeneOntology** |
| GO:0004163 |
| GO:0005524 |
| GO:0005829 |
| GO:0006695 |
| GO:0008284 |
| GO:0008299 |
| GO:0016301 |
| GO:0030544 |
| GO:0042803 |
| GO:0044281 |
|  |
| **UCSC Genome Browser** |
| uc002flg.1 |
|  |
| **WikiGenes** |
| 4597 |
|  |
| **Affy** |
| 11729695\_a\_at |
| 203027\_s\_at |
| 34410\_at |
| 8003332 |
| 88679\_at |
| U49260\_at |
