# Supplementary material for: Automatically visualise and analyse data on pathways using PathVisioRPC from any programming environment
Source: BMC Bioinformatics. 2015 Aug 23;16(1):267. doi: 10.1186/s12859-015-0708-8 (PMC4546821; doi:10.1186/s12859-015-0708-8)
Supplement: Additional file 3: — Examples in Python. This zip archive contains the data and python script for the three python examples. (ZIP 15714 kb) [file 12859_2015_708_MOESM3_ESM.zip › Python_Examples/result_Example_3/Cholesterol Biosynthesis/backpage/L_4598.html]

 

# GeneProduct annotation

  

| Name: MVK| Identifier: 4598| Database: Entrez Gene| Synonyms: MK | | | --- | --- | | | | --- | --- | --- | --- | | | | --- | --- | --- | --- | --- | --- | | |
| --- | --- | --- | --- | --- | --- | --- | --- |

# Expression data

**Gene id on mapp: 4598**

| Sample name 4598| logFC1 1.552862591| Pvalue1 0.187850729| logFC2 1.840064182| Pvalue2 0.13177333 | | | --- | --- | | | | --- | --- | --- | --- | | | | --- | --- | --- | --- | --- | --- | | | | --- | --- | --- | --- | --- | --- | --- | --- | | |
| --- | --- | --- | --- | --- | --- | --- | --- | --- | --- |

  
  

---

  
  

# Cross references

  

|  |
| --- |
|  |
| **UniGene** |
| Hs.130607 |
|  |
| **Agilent** |
| A\_14\_P115327 |
| A\_23\_P105592 |
| A\_24\_P372901 |
|  |
| **Ensembl** |
| ENSG00000110921 |
|  |
| **Gene Wiki** |
| 4598 |
|  |
| **HGNC** |
| MVK |
|  |
| **Illumina** |
| 0001660358 |
| ILMN\_1786310 |
|  |
| **Entrez Gene** |
| 4598 |
|  |
| **OMIM** |
| 251170 |
| 260920 |
| 610377 |
|  |
| **PDB** |
| 2R3V |
|  |
| **RefSeq** |
| NM\_000431 |
| NM\_001114185 |
| NP\_000422 |
| NP\_001107657 |
|  |
| **Uniprot/TrEMBL** |
| B2RDU6 |
| B7Z1C2 |
| F5GXC0 |
| F5H092 |
| F5H163 |
| F5H368 |
| F5H8H2 |
| Q03426 |
|  |
| **GeneOntology** |
| GO:0004496 |
| GO:0005524 |
| GO:0005737 |
| GO:0005829 |
| GO:0006695 |
| GO:0008299 |
| GO:0019287 |
| GO:0042802 |
| GO:0044281 |
| GO:0050728 |
|  |
| **UCSC Genome Browser** |
| uc001toz.4 |
| uc009zvk.3 |
| uc010sxr.2 |
| uc021rdo.1 |
|  |
| **WikiGenes** |
| 4598 |
|  |
| **Affy** |
| 11719098\_a\_at |
| 11747747\_a\_at |
| 36907\_at |
| 59750\_at |
| 59893\_at |
| 7958565 |
| M88468\_at |
