# Supplementary material for: Automatically visualise and analyse data on pathways using PathVisioRPC from any programming environment
Source: BMC Bioinformatics. 2015 Aug 23;16(1):267. doi: 10.1186/s12859-015-0708-8 (PMC4546821; doi:10.1186/s12859-015-0708-8)
Supplement: Additional file 3: — Examples in Python. This zip archive contains the data and python script for the three python examples. (ZIP 15714 kb) [file 12859_2015_708_MOESM3_ESM.zip › Python_Examples/result_Example_3/Cholesterol Biosynthesis/backpage/L_50814.html]

 

# GeneProduct annotation

  

| Name: NSDHL| Identifier: 50814| Database: Entrez Gene| Synonyms: H105E3 | | | --- | --- | | | | --- | --- | --- | --- | | | | --- | --- | --- | --- | --- | --- | | |
| --- | --- | --- | --- | --- | --- | --- | --- |

# Expression data

**Gene id on mapp: 50814**

| Sample name 50814| logFC1 0.515842872| Pvalue1 0.330654601| logFC2 1.305519406| Pvalue2 0.317723484 | | | --- | --- | | | | --- | --- | --- | --- | | | | --- | --- | --- | --- | --- | --- | | | | --- | --- | --- | --- | --- | --- | --- | --- | | |
| --- | --- | --- | --- | --- | --- | --- | --- | --- | --- |

  
  

---

  
  

# Cross references

  

|  |
| --- |
|  |
| **UniGene** |
| Hs.57698 |
|  |
| **Agilent** |
| A\_14\_P100798 |
| A\_23\_P171034 |
| A\_33\_P3236082 |
|  |
| **Ensembl** |
| ENSG00000147383 |
| ENSG00000269336 |
|  |
| **Gene Wiki** |
| 50814 |
|  |
| **HGNC** |
| NSDHL |
|  |
| **Illumina** |
| 0006350524 |
| ILMN\_1692163 |
|  |
| **Entrez Gene** |
| 50814 |
|  |
| **OMIM** |
| 300275 |
| 300831 |
| 308050 |
|  |
| **RefSeq** |
| NM\_001129765 |
| NM\_015922 |
| NP\_001123237 |
| NP\_057006 |
|  |
| **Uniprot/TrEMBL** |
| C9JDR0 |
| Q15738 |
|  |
| **GeneOntology** |
| GO:0001942 |
| GO:0003824 |
| GO:0003854 |
| GO:0005783 |
| GO:0005789 |
| GO:0005811 |
| GO:0006694 |
| GO:0006695 |
| GO:0007224 |
| GO:0008203 |
| GO:0008831 |
| GO:0009058 |
| GO:0016021 |
| GO:0016616 |
| GO:0043231 |
| GO:0044237 |
| GO:0044281 |
| GO:0045226 |
| GO:0047012 |
| GO:0050662 |
| GO:0055114 |
| GO:0060716 |
|  |
| **UCSC Genome Browser** |
| uc004fgs.1 |
| uc004fgt.1 |
|  |
| **WikiGenes** |
| 50814 |
|  |
| **Affy** |
| 11720522\_a\_at |
| 215093\_at |
| 36500\_at |
| 8170590 |
| U47105\_at |
