# Supplementary material for: Automatically visualise and analyse data on pathways using PathVisioRPC from any programming environment
Source: BMC Bioinformatics. 2015 Aug 23;16(1):267. doi: 10.1186/s12859-015-0708-8 (PMC4546821; doi:10.1186/s12859-015-0708-8)
Supplement: Additional file 3: — Examples in Python. This zip archive contains the data and python script for the three python examples. (ZIP 15714 kb) [file 12859_2015_708_MOESM3_ESM.zip › Python_Examples/result_Example_3/Cholesterol Biosynthesis/backpage/L_6307.html]

 

# GeneProduct annotation

  

| Name: MSMO1| Identifier: 6307| Database: Entrez Gene| Synonyms: ERG25 | | | --- | --- | | | | --- | --- | --- | --- | | | | --- | --- | --- | --- | --- | --- | | |
| --- | --- | --- | --- | --- | --- | --- | --- |

# Expression data

**Gene id on mapp: 6307**

| Sample name 6307| logFC1 2.973829438| Pvalue1 0.471936395| logFC2 3.126039813| Pvalue2 0.457187068 | | | --- | --- | | | | --- | --- | --- | --- | | | | --- | --- | --- | --- | --- | --- | | | | --- | --- | --- | --- | --- | --- | --- | --- | | |
| --- | --- | --- | --- | --- | --- | --- | --- | --- | --- |

  
  

---

  
  

# Cross references

  

|  |
| --- |
|  |
| **UniGene** |
| Hs.105269 |
|  |
| **Agilent** |
| A\_14\_P110787 |
| A\_14\_P125113 |
| A\_23\_P110184 |
|  |
| **Ensembl** |
| ENSG00000052802 |
|  |
| **Gene Wiki** |
| 6307 |
|  |
| **HGNC** |
| MSMO1 |
|  |
| **Illumina** |
| ILMN\_1689842 |
| ILMN\_1720889 |
| ILMN\_2402499 |
|  |
| **Entrez Gene** |
| 6307 |
|  |
| **OMIM** |
| 607545 |
|  |
| **RefSeq** |
| NM\_001017369 |
| NM\_006745 |
| NP\_001017369 |
| NP\_006736 |
|  |
| **Uniprot/TrEMBL** |
| D6R952 |
| D6RDP9 |
| D6REA2 |
| Q15800 |
|  |
| **GeneOntology** |
| GO:0000254 |
| GO:0005506 |
| GO:0005783 |
| GO:0005789 |
| GO:0005886 |
| GO:0006631 |
| GO:0006633 |
| GO:0006695 |
| GO:0008202 |
| GO:0016021 |
| GO:0016491 |
| GO:0044281 |
| GO:0055114 |
|  |
| **UCSC Genome Browser** |
| uc003ire.3 |
| uc003irf.3 |
| uc010irb.3 |
|  |
| **WikiGenes** |
| 6307 |
|  |
| **Affy** |
| 11740092\_x\_at |
| 11741012\_a\_at |
| 11757764\_s\_at |
| 11763767\_at |
| 209146\_at |
| 33369\_at |
| 33370\_r\_at |
| 8098195 |
| 88287\_at |
| U60205\_at |
