# Supplementary material for: Automatically visualise and analyse data on pathways using PathVisioRPC from any programming environment
Source: BMC Bioinformatics. 2015 Aug 23;16(1):267. doi: 10.1186/s12859-015-0708-8 (PMC4546821; doi:10.1186/s12859-015-0708-8)
Supplement: Additional file 3: — Examples in Python. This zip archive contains the data and python script for the three python examples. (ZIP 15714 kb) [file 12859_2015_708_MOESM3_ESM.zip › Python_Examples/result_Example_3/Cholesterol Biosynthesis/backpage/L_6309.html]

 

# GeneProduct annotation

  

| Name: SC5D| Identifier: 6309| Database: Entrez Gene| Synonyms: ERG3 | | | --- | --- | | | | --- | --- | --- | --- | | | | --- | --- | --- | --- | --- | --- | | |
| --- | --- | --- | --- | --- | --- | --- | --- |

# Expression data

**Gene id on mapp: 6309**

| Sample name 6309| logFC1 0.303569346| Pvalue1 0.047007119| logFC2 0.400072243| Pvalue2 0.039725122 | | | --- | --- | | | | --- | --- | --- | --- | | | | --- | --- | --- | --- | --- | --- | | | | --- | --- | --- | --- | --- | --- | --- | --- | | |
| --- | --- | --- | --- | --- | --- | --- | --- | --- | --- |

  
  

---

  
  

# Cross references

  

|  |
| --- |
|  |
| **UniGene** |
| Hs.287749 |
| Hs.593340 |
| Hs.657840 |
|  |
| **Agilent** |
| A\_14\_P200325 |
| A\_23\_P372888 |
| A\_23\_P98446 |
|  |
| **Ensembl** |
| ENSG00000109929 |
|  |
| **Gene Wiki** |
| 6309 |
|  |
| **HGNC** |
| SC5D |
|  |
| **Illumina** |
| 0006350746 |
| ILMN\_1672680 |
| ILMN\_1677607 |
| ILMN\_1727236 |
|  |
| **Entrez Gene** |
| 6309 |
|  |
| **OMIM** |
| 602286 |
| 607330 |
|  |
| **RefSeq** |
| NM\_001024956 |
| NM\_006918 |
| NP\_001020127 |
| NP\_008849 |
|  |
| **Uniprot/TrEMBL** |
| E9PPW5 |
| E9PQ91 |
| O75845 |
| Q6GTM5 |
|  |
| **GeneOntology** |
| GO:0000248 |
| GO:0005506 |
| GO:0005789 |
| GO:0006629 |
| GO:0006633 |
| GO:0006695 |
| GO:0016021 |
| GO:0016491 |
| GO:0033490 |
| GO:0043231 |
| GO:0044281 |
| GO:0050046 |
| GO:0055114 |
|  |
| **UCSC Genome Browser** |
| uc001pxs.1 |
| uc001pxu.3 |
| uc001pxv.3 |
|  |
| **WikiGenes** |
| 6309 |
|  |
| **Affy** |
| 11717950\_s\_at |
| 11733780\_a\_at |
| 11757184\_a\_at |
| 211423\_s\_at |
| 33421\_s\_at |
| 7944656 |
| D85181\_at |
