# Supplementary material for: Automatically visualise and analyse data on pathways using PathVisioRPC from any programming environment
Source: BMC Bioinformatics. 2015 Aug 23;16(1):267. doi: 10.1186/s12859-015-0708-8 (PMC4546821; doi:10.1186/s12859-015-0708-8)
Supplement: Additional file 3: — Examples in Python. This zip archive contains the data and python script for the three python examples. (ZIP 15714 kb) [file 12859_2015_708_MOESM3_ESM.zip › Python_Examples/result_Example_3/Cholesterol Biosynthesis/backpage/L_66234.html]

 

# GeneProduct annotation

  

| Name: Sc4mol| Identifier: 66234| Database: Entrez Gene| Synonyms: ERG25 | | | --- | --- | | | | --- | --- | --- | --- | | | | --- | --- | --- | --- | --- | --- | | |
| --- | --- | --- | --- | --- | --- | --- | --- |

# Expression data

**Gene id on mapp: 66234**

| Sample name 66234| logFC 1.759683091| Pvalue 0.650539306 | | | --- | --- | | | | --- | --- | --- | --- | | |
| --- | --- | --- | --- | --- | --- |

  
  

---

  
  

# Cross references

  

|  |
| --- |
|  |
| **UniGene** |
| Mm.30119 |
| Mm.475452 |
|  |
| **Agilent** |
| A\_51\_P209372 |
|  |
| **Ensembl** |
| ENSMUSG00000031604 |
|  |
| **Illumina** |
| ILMN\_1250562 |
| ILMN\_2823778 |
|  |
| **Entrez Gene** |
| 66234 |
|  |
| **MGI** |
| MGI:1913484 |
|  |
| **RefSeq** |
| NM\_025436 |
| NP\_079712 |
|  |
| **Uniprot/TrEMBL** |
| D3YTN3 |
| D3Z458 |
| Q9CRA4 |
|  |
| **GeneOntology** |
| GO:0000254 |
| GO:0005506 |
| GO:0005789 |
| GO:0006633 |
| GO:0016021 |
| GO:0016126 |
|  |
| **UCSC Genome Browser** |
| uc009lux.1 |
| uc009luy.1 |
|  |
| **WikiGenes** |
| 66234 |
|  |
| **Affy** |
| 10578916 |
| 1423078\_a\_at |
| 1459627\_at |
| 160388\_at |
