# Supplementary material for: Automatically visualise and analyse data on pathways using PathVisioRPC from any programming environment
Source: BMC Bioinformatics. 2015 Aug 23;16(1):267. doi: 10.1186/s12859-015-0708-8 (PMC4546821; doi:10.1186/s12859-015-0708-8)
Supplement: Additional file 3: — Examples in Python. This zip archive contains the data and python script for the three python examples. (ZIP 15714 kb) [file 12859_2015_708_MOESM3_ESM.zip › Python_Examples/result_Example_3/Cholesterol Biosynthesis/backpage/L_6713.html]

 

# GeneProduct annotation

  

| Name: SQLE| Identifier: 6713| Database: Entrez Gene | | | --- | --- | | | | --- | --- | --- | --- | | |
| --- | --- | --- | --- | --- | --- |

# Expression data

**Gene id on mapp: 6713**

| Sample name 6713| logFC1 0.026767304| Pvalue1 0.013625508| logFC2 0.062051026| Pvalue2 0.013089221 | | | --- | --- | | | | --- | --- | --- | --- | | | | --- | --- | --- | --- | --- | --- | | | | --- | --- | --- | --- | --- | --- | --- | --- | | |
| --- | --- | --- | --- | --- | --- | --- | --- | --- | --- |

  
  

---

  
  

# Cross references

  

|  |
| --- |
|  |
| **UniGene** |
| Hs.597714 |
| Hs.71465 |
|  |
| **Agilent** |
| A\_14\_P129257 |
| A\_23\_P146284 |
|  |
| **Ensembl** |
| ENSG00000104549 |
|  |
| **Gene Wiki** |
| 6713 |
|  |
| **HGNC** |
| SQLE |
|  |
| **Illumina** |
| 0004120047 |
| ILMN\_1772241 |
| ILMN\_2041293 |
|  |
| **Entrez Gene** |
| 6713 |
|  |
| **OMIM** |
| 602019 |
|  |
| **RefSeq** |
| NM\_003129 |
| NP\_003120 |
|  |
| **Uniprot/TrEMBL** |
| B4DHX0 |
| E5RJH9 |
| E7EVQ6 |
| H0YBN7 |
| Q14534 |
| Q9UNR6 |
|  |
| **GeneOntology** |
| GO:0004506 |
| GO:0005789 |
| GO:0006695 |
| GO:0006725 |
| GO:0008203 |
| GO:0010033 |
| GO:0016021 |
| GO:0016126 |
| GO:0016491 |
| GO:0043231 |
| GO:0044281 |
| GO:0050660 |
| GO:0055114 |
|  |
| **UCSC Genome Browser** |
| uc011liq.2 |
|  |
| **WikiGenes** |
| 6713 |
|  |
| **Affy** |
| 11717729\_a\_at |
| 11717730\_s\_at |
| 11717731\_at |
| 11750969\_a\_at |
| 209218\_at |
| 35839\_at |
| 8148280 |
| D78129\_at |
