# Supplementary material for: Automatically visualise and analyse data on pathways using PathVisioRPC from any programming environment
Source: BMC Bioinformatics. 2015 Aug 23;16(1):267. doi: 10.1186/s12859-015-0708-8 (PMC4546821; doi:10.1186/s12859-015-0708-8)
Supplement: Additional file 3: — Examples in Python. This zip archive contains the data and python script for the three python examples. (ZIP 15714 kb) [file 12859_2015_708_MOESM3_ESM.zip › Python_Examples/result_Example_3/Cholesterol Biosynthesis/backpage/L_68603.html]

 

# GeneProduct annotation

  

| Name: Pmvk| Identifier: 68603| Database: Entrez Gene| Synonyms: 2900002L22Rik | | | --- | --- | | | | --- | --- | --- | --- | | | | --- | --- | --- | --- | --- | --- | | |
| --- | --- | --- | --- | --- | --- | --- | --- |

# Expression data

**Gene id on mapp: 68603**

| Sample name 68603| logFC 0.282031| Pvalue 3.86654E-4 | | | --- | --- | | | | --- | --- | --- | --- | | |
| --- | --- | --- | --- | --- | --- |

  
  

---

  
  

# Cross references

  

|  |
| --- |
|  |
| **UniGene** |
| Mm.34242 |
|  |
| **Agilent** |
| A\_51\_P492408 |
| A\_51\_P492410 |
| A\_55\_P2076533 |
|  |
| **Ensembl** |
| ENSMUSG00000027952 |
|  |
| **Illumina** |
| ILMN\_1227596 |
|  |
| **Entrez Gene** |
| 68603 |
|  |
| **MGI** |
| MGI:1915853 |
|  |
| **RefSeq** |
| NM\_026784 |
| NP\_081060 |
| NP\_081624 |
|  |
| **Uniprot/TrEMBL** |
| D3YXP6 |
| Q9D1G2 |
|  |
| **GeneOntology** |
| GO:0004631 |
| GO:0005524 |
| GO:0005737 |
| GO:0005777 |
| GO:0005829 |
| GO:0006695 |
| GO:0008299 |
| GO:0016126 |
| GO:0019287 |
| GO:0070723 |
|  |
| **UCSC Genome Browser** |
| uc008pzt.2 |
|  |
| **WikiGenes** |
| 68603 |
|  |
| **Affy** |
| 10493548 |
| 111380\_at |
| 1427893\_a\_at |
| 167266\_f\_at |
