# Supplementary material for: Automatically visualise and analyse data on pathways using PathVisioRPC from any programming environment
Source: BMC Bioinformatics. 2015 Aug 23;16(1):267. doi: 10.1186/s12859-015-0708-8 (PMC4546821; doi:10.1186/s12859-015-0708-8)
Supplement: Additional file 10: — R packages. This zip archive contains both the RPathVisio package and the BridgeDbR package. The RPathVisio and BridgeDbR packages can be used in R. The jar files are being provided for archival purposes, for use please obtain a recent version of the software from the project websites. (ZIP 14914 kb) [file 12859_2015_708_MOESM10_ESM.zip › rPackages/BridgeDbR/BridgeDbR_1.0.0.tar.gz!/BridgeDbR/inst/doc/tutorial.pdf]

An R package with BridgeDb for identifier mapping

Egon Willighagen

March 16, 2014

1 Introduction

BridgeDb is a combination of an application programming interface (API), library, and set of data files for mapping identifiers for identical objects [2]. Because BridgeDb is use by projects in bioinformatics, like WikiPathways and PathVisio [1, 3], identifier mapping databases are available for gene products and metabolites.

2 Concepts

BridgeDb has a few core concepts which are explained in this section. Much of the API requires one to be familiar with these concepts, though some are not always applicable. The first concept is an example of that: organisms, which do not apply to metabolites.

2.1 Organisms

However, for genes the organism is important: the same gene has different identifiers in different organisms. BridgeDb identifies organisms by their latin name and with a two character code. Because identifier mapping files provided by PathVisio have names with these short codes, it can be useful to have a conversion method:

```
> code = getOrganismCode("Rattus norvegicus")
> code
```

```
[1] "Rn"
```

2.2 Data Sources

Identifiers have a context and this context is often a database. For example, metabolite identfiers can be provided by the Human Metabolome Database (HMDB), ChemSpider, PubChem, and ChEBI. Similarly, gene product identifiers can be provided by databases like Ensemble. Such a database providing identifiers is in BridgeDb called a data source.

Importantly, each such data source is identified by a human readable long name and by a short system code. This package has methods to interconvert one into the other:

```
> fullName <- getFullName("Ce")
> fullName

[1] "ChEBI"

> code <- getSystemCode("ChEBI")
> code

[1] "Ce"
```

2.3 Identifier Patterns

Another useful aspect of BridgeDb is that it knows about the patterns of identifiers. If this pattern is unique enough, it can be used used to automatically find the data sources that match a particular identifier. For example:

```
> getMatchingSources("HMDB00555")

 [1] "NCBI Protein"                    "KEGG Pathway"
 [3] "HMDB"                            "Ensembl Plants"
 [5] "LipidBank"                       "HGNC"
 [7] "NCI Pathway Interaction Database" "EMBL"
 [9] "SUPFAM"                          "SWISS-MODEL"

> getMatchingSources("ENSG00000100030")

 [1] "NCBI Protein"                    "Ensembl"
 [3] "Ensembl Plants"                  "LipidBank"
 [5] "HGNC"                            "NCI Pathway Interaction Database"
 [7] "Ensembl Human"                   "EMBL"
 [9] "SUPFAM"                          "SWISS-MODEL"
```

3 Identifier Mapping Databases

The BridgeDb package primarily provides the software framework, and not identifier mapping data. Identifier Mapping databases can be downloaded from various websites. The package knows about the download location provided by PathVisio, and we can query for all gene product identifier mapping databases:

```
> getBridgeNames()
```

```
 [1] "Ag_Derby_20130701.bridge" "An_Derby_20130701.bridge"
 [3] "At_Derby_20130701.bridge" "Bs_Derby_20130701.bridge"
 [5] "Bt_Derby_20130701.bridge" "Ce_Derby_20130701.bridge"
 [7] "Cf_Derby_20130701.bridge" "Ci_Derby_20130701.bridge"
 [9] "Ct_Derby_20120212.bridge" "Dm_Derby_20130701.bridge"
[11] "Dr_Derby_20130701.bridge" "Ec_Derby_20130701.bridge"
[13] "Gg_Derby_20130701.bridge" "Gm_Derby_20130701.bridge"
[15] "Gz_Derby_20130701.bridge" "Hs_Derby_20130701.bridge"
[17] "Ml_Derby_20130701.bridge" "Mm_Derby_20130701.bridge"
[19] "Mx_Derby_20130701.bridge" "Oa_Derby_20130701.bridge"
[21] "Oi_Derby_20130701.bridge" "Oj_Derby_20130701.bridge"
[23] "Pi_Derby_20130701.bridge" "Pt_Derby_20130701.bridge"
[25] "Qc_Derby_20130701.bridge" "Rn_Derby_20130701.bridge"
[27] "Sc_Derby_20130701.bridge" "Sl_Derby_20130701.bridge"
[29] "Ss_Derby_20130701.bridge" "Vv_Derby_20130701.bridge"
[31] "Xt_Derby_20130701.bridge" "Zm_Derby_20130701.bridge"
```

3.1 Downloading

The package provides a convenience method to download such identifier mapping databases. For example, we can save the identifier mapping database for rat to the current folder with:

```
> dbLocation <- getDatabase("Rattus norvegicus",location=getwd())
```

The dbLocation variable then contains the location of the identifier mapping file that was downloaded.

3.2 Loading Databases

Once you have downloaded an identifier mapping database, either manually or via the getDatabase() method, you need to load the database for the identifier mappings to become available.

```
> mapper <- loadDatabase(dbLocation)
```

4 Mapping Identifiers

With a loaded database, identifiers can be mapped. The mapping method uses system codes. So, to map the human Entrez Gene identifier (system code: L) 196410 to Affy identifiers (system code: X) we use:

```
> location <- getDatabase("Homo sapiens")
> mapper <- loadDatabase(location)
> map(mapper, "L", "196410", "X")
```

Mind you, this returns more than one identifier, as BridgeDb is generally a one to many mapping database.

References

[1] A. R. Pico, T. Kelder, M. P. van Iersel, K. Hanspers, B. R. Conklin, and C. Evelo. WikiPathways: Pathway editing for the people. *PLoS Biol*, 6(7):e184+, July 2008.

[2] M. van Iersel, A. Pico, T. Kelder, J. Gao, I. Ho, K. Hanspers, B. Conklin, and C. Evelo. The BridgeDb framework: standardized access to gene, protein and metabolite identifier mapping services. *BMC Bioinformatics*, 11(1):5+, 2010.

[3] M. P. van Iersel, T. Kelder, A. R. Pico, K. Hanspers, S. Coort, B. R. Conklin, and C. Evelo. Presenting and exploring biological pathways with PathVisio. *BMC Bioinformatics*, 9, SEP 25 2008.
