# Supplementary material for: Automatically visualise and analyse data on pathways using PathVisioRPC from any programming environment
Source: BMC Bioinformatics. 2015 Aug 23;16(1):267. doi: 10.1186/s12859-015-0708-8 (PMC4546821; doi:10.1186/s12859-015-0708-8)
Supplement: Additional file 10: — R packages. This zip archive contains both the RPathVisio package and the BridgeDbR package. The RPathVisio and BridgeDbR packages can be used in R. The jar files are being provided for archival purposes, for use please obtain a recent version of the software from the project websites. (ZIP 14914 kb) [file 12859_2015_708_MOESM10_ESM.zip › rPackages/RPathVisio/RPathVisio_1.0.0.tar.gz!/RPathVisio/inst/doc/tutorial.pdf]

A RPathVisio Tutorial

Christ Leemans Egon Willighagen Anwesha Bohler

September 30, 2014

1 Introduction

This tutorial will talk you try the basic RPathVisio commands. The RPathVisio package enables the use of the pathway analysis tool PathVisio `http://www.pathvisio.org` and the wealth of Wikipathways `http://wikipathways.org/index.php/WikiPathways` pathways in the R eironment. This package wraps xml.rpc() calls made to the PathVisioRPC Server`https://github.com/pennatula/PathVisioRPC`, which is an XML-RPC based implementation of PathVisio[2], into an R API that hides more of the technologies of XML-RPC. The package also sets a number of typical defaults and performs some argument validation in order to simplify usage and provide better feedback to the user.

2 Running the server

Running the server on the default port can be performed from within R:

```
> library(pvxmlrpc)
> port = pathvisioServer()
> cat(paste("Port:", port, "\n"))
```

```
Port: 9000
```

3 XML-RPC

This package uses the XML-RPC extension of PathVisio to interact with it. Therefore, you can use the normal XML-RPC code. However, we decided to

provide a number of convenience methods to hide the more technical aspects of this communication, and make some reasonable assumptions for default values. Therefore, the following two commands are equivalent.

```
> pathway = createPathway("Test pathway", author="Jan Janssen")
> pathway = xml.rpc(
+   "http://localhost:9000/",
+   "PathVisio.createPathway",
+   "Jan Janssen", "Test pathway", "Homo Sapiens","/home/user/PathVisioRPC-Resul
+ )
```

You decide what approach you prefer to use.

3.1 Server and port

This requires the port it runs on to be free. Need you use a different port, than you can use the *port* parameter:

```
> port = pathvisioServer(port=8080)
> cat(paste("Port:", port, "\n"))

Port: 8080
```

The advantage of using the default port, is that the other methods will accept those default so that you do not have to give them explicitly. If you run your PathVisio-RPC server on a different machine, or if you use a non-default port, you can use the *host* and *port* parameters available for all methods.

4 First Steps

4.1 Creating pathways

The pathway is the central object in PathVisio. Any thing, metabolite, protein, gene, etc, are contained in a pathway. Therefore, the first thing to do is create a pathway. This can be done with:

```
> pathway = createPathway("Test pathway")
```

Optionally, you can provide author information and/or the organism in which the pathway is found:

```
> pathway = createPathway("Test pathway", author="Jan Janssen", organism="Homo s
```

These pathways are stored as GPML files in the PathVisioRPC-Results folder in the home directory. When you prefer the file to be stored in another folder you can provide an alternative path:

```
> altpath = getwd()
> pathway = createPathway("Test pathway", author="Jan Janssen", organism="Homo s
```

The createPathway function also creates a named list with a *name*, *author*, *organism* and *path*.

```
> pathway$name

[1] "Test pathway"

> pathway$author

[1] "Jan Janssen"

> pathway$organism

[1] "Homo sapiens"

> pathway$path

[1] "/tmp/RtmpbgqydF/Rbuild4df91fd03778/RPathVisio/vignettes"
```

4.2 Adding content

Next thing is to add content to the pathway. The central piece of content in a pathway is a data node, which must have a name and a type:

```
> n1 = addDataNode("Test pathway", "SomeGene", "GeneProduct")
```

Possible types are *Metabolite*, *Protein*, *GeneProduct*, *RNA*, *Unknown* and *Pathway*. The last one is for refering to related pathways.

In this function the following additional information on the data node can be provided as well: *dataId* - an unique identifier for the object, e.g. an enseml identifier *datasource* - source of the identifier given, e.g. ensembl

an alternative path where the pathway is stored can be given as well.

```
> n1 = addDataNode("Test pathway","SomeGene","GeneProduct",dataId="ENSG000111",d
```

The values are returned in a list and are as follows:

```
> n1$pathway

[1] "Test pathway"

> n1$name

[1] "SomeGene"

> n1$type

NULL

> n1$dataId

[1] "ENSG000111"

> n1$dataSource

[1] "Ensembl"

> n1$path

[1] "/tmp/RtmpbgqydF/Rbuild4df91fd03778/RPathVisio/vignettes"
```

Making connections between nodes define the paths of the pathway, and is done with the *addLine* method.

```
> pwy = createPathway("Butyrate")
> aa = addDataNode(pwy$name, "Acetoacetate", "Metabolite")
> hb =addDataNode(pwy$name, "3-Hydroxy-butyrate", "Metabolite")
> l = addLine(pwy$name, "A->B", "Acetoacetate", "3-Hydroxy-butyrate")
```

4.3 Convenience methods

To further simply the R code to create pathways, we have added methods specific for the various data node types. For example, we can also add a metabolite with:

```
> m = addMetaboliteNode("Test pathway", "Acetoacetate")
```

With line types we've done this differently, in this case we have chosen to make a seperate function to add Mim-types.

```
> name = "ACE inhibitor"
> pwy = createPathway(name)
> m = addMetaboliteNode(name,"captopril")
> p = addProteinNode(name,"ACE")
> l = addMim(name,"inhibitor mim-type",m$name,p$name,"inhibition")
```

4.4 exporting a pathway

When you've created your own pathway, you can use the exportPathway function to export your pathway into a file format like png.

```
> exportPathway("ACE inhibitor","png")
```

The result can be seen in Figure 1.

5 Creating graphics

But of course it is all about the looks. For example, we can plot pathways from Wikipathways [1] in R with:

```
> plotWikipathway("WP2366")
```

The result is shown in Figure 2.

6 Visualizing experimental data

With this package it's also possible to visualize your experimental data on pathways. This can be done in three steps:

6.1 import data in a PathVisio expression file(.pgex)

First a Pathvisio expression file needs to be created. This can be done in two ways: with a text file and with a dataframe. I will show how it's done with the a dataframe. The function goes as follows:

```
> importData(name, dataframe, dbname, host = "localhost", port = 9000, dbpath =
```

The required paremeter "name" will be used to name the file created ("name.pgex"). The parameter dataframe is for the input dataframe and the parameter dbname is for the name of the database file used(e.g. a .bridge file).

The host and port are for the address the server is running on and dbpath and outputdir are the directory where the database is in and the directory where the results are saved. row.names is for when the row names are the gene identifiers, if the gene identifiers are in the first collumn, row.names needs to be set to "FALSE".

The source parameter can be set in order to specify what the source of the identifiers is. The data sources supported by PathVisio can be found at: `http://www.pathvisio.org/wiki/DatabasesMapps`. The import data function will use this to create a column with a "system code", this makes sure that the correct source is set when the expression set is created.

Users can either set the source parameter using the full name of the data source, or with the system code. When this parameter is not set, the function will use the first identifier to try and match the pattern to a possible source. This can have three different outcomes: - If only one source matches the pattern, this source is set as data source - If there are more than one source's that match the pattern, an error is reported with the possible data sources. - If there are no matches, an error is reported that the data source can not be found

In this example we have the following data frame:

```
> head(dataframe)
```

```
  ENTREZG_ID    logFC Fold.Change  AveExpr        t      P.Value    adj.P.Val
1      54242 3.591656    12.05581 3.936865 37.87144 2.307490e-11 9.970666e-08
2     286958 5.883800    59.04733 5.147393 25.31430 8.875152e-10 1.917477e-06
3     498659 7.091297   136.36193 6.045063 22.48891 2.577609e-09 3.411729e-06
4      54231 4.570566    23.76170 6.878709 21.52817 3.816052e-09 3.411729e-06
```

```
5      25374 4.525257    23.02705 5.274847 21.44692 3.947846e-09 3.411729e-06
6      29266 4.248603    19.00890 4.945616 20.26089 6.575952e-09 4.143711e-06
         B
1 15.77209
2 12.98266
3 12.05014
4 11.69601
5 11.66510
6 11.19593
```

As can be seen by the top of the data frame, it has a column with Entrez Gene identifiers and columns with statistical results. The identifiers are not the row name, so row.names=FALSE. Let's first see what happens if we don't use a system code:

```
> importData("example",dataframe,"Rn_Derby",row.names=FALSE)
```

As we can see, there are three different source's that have the same pattern as the first identifier. Entrez Gene indeed is one of them, so now let's try again with the system code:

```
> importData("example",dataframe,"Rn_Derby",row.names=FALSE,source="Entrez Gene"
[1] "example.txt imported & example.txt.pgex created in/tmp/RtmpbgqydF/Rbuild4df91
```

Success! With "Entrez Gene" as source name, the expression set was created. Another way of doing this was by using the system code "L" directly.

6.2 creating a visualization

Data can be visualized on pathways using colours. A gradient colouring scheme can be used to visualize a range of data on a gene (eg. Fold Change) while a rule can be applied for certain criteria allowing only the genes which qualify to be coloured (e.g P Value < 0.05) There are three functions for creating visualizations: createVisualization, createVisualizationGradient and createVisualizationRule. The createVizualization can be used to create both a rule and a gradient. If the user wants to create more then one rule, or gradient, values need to be seperated with a ";". Let's create a visualization rule and gradient:

```
> createVisualization("example.txt.pgex","logFC","blue,white,red","-1,0,4","adj.
[1] "/tmp/RtmpbgqydF/Rbuild4df91fd03778/RPathVisio/vignettes/example.txt.pgex.xml-
```

6.3 visualizing data on a Pathway

Now that the visualization is created, we can visualize data on a pathway either on a local pathway using visualizeData(), or on a pathway from WikiPathways using visualizeDataByURI(). Both functions need to have 3 parameters: the pathway(either file or URI), the expression file and the database file. Let's visualize it on the cell cycle pathway from wikipathways, seen here. The code for this pathway is "WP429", this identifier can be seen in the url on wikipathways.

```
> visualizeDataByURI("WP429","example.txt.pgex","Rn_Derby")

[1] "Data Visualized on Cell cycle, results in /tmp/RtmpbgqydF/Rbuild4df91fd03778/
```

Now a folder is created in the working directory, this folder has a html page to view the results and an image, the image created can be seen below in Figure 3.

References

[1] A.~R. Pico, T.~Kelder, M.~P. van Iersel, K.~Hanspers, B.~R. Conklin, and C.~Evelo. WikiPathways: Pathway editing for the people. *PLoS Biol*, 6(7):e184+, July 2008.

[2] M.~P. van Iersel, T.~Kelder, A.~R. Pico, K.~Hanspers, S.~Coort, B.~R. Conklin, and C.~Evelo. Presenting and exploring biological pathways with PathVisio. *BMC Bioinformatics*, 9, SEP 25 2008.

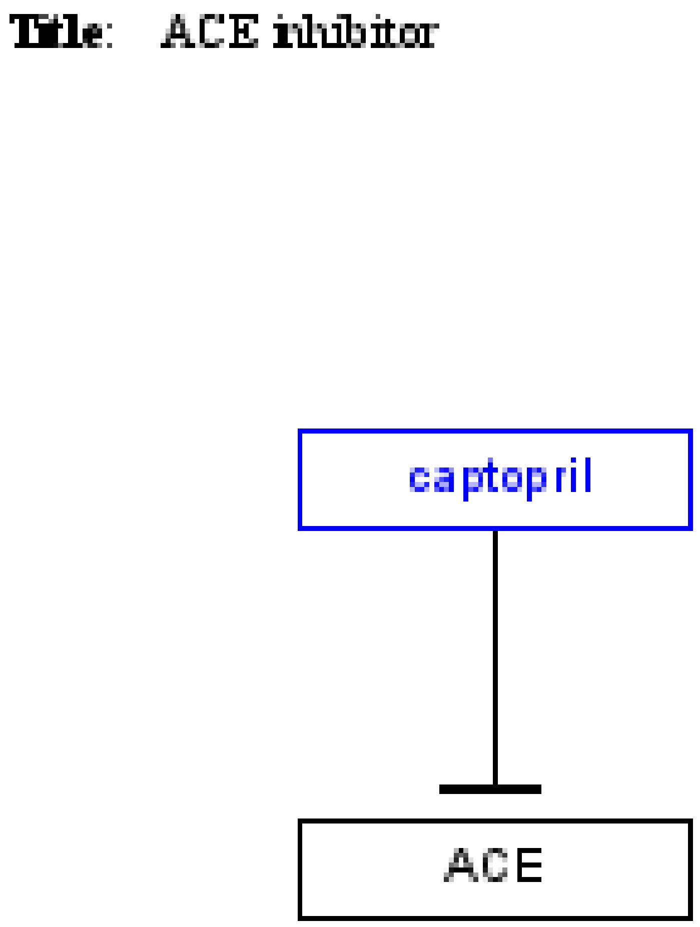

inhibitor.png

Figure 1: ACE inhibitor.png

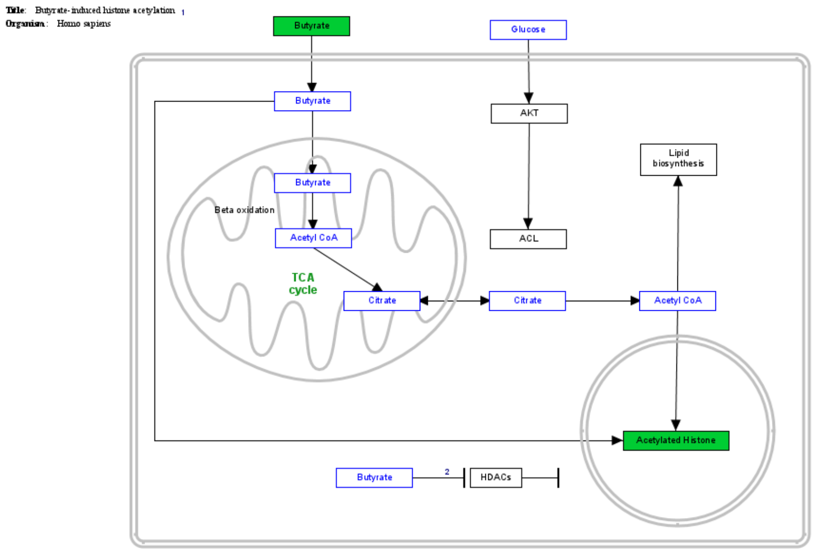

Figure 2: WikiPathways WP2366.

cycle/image/Cell cycle.png

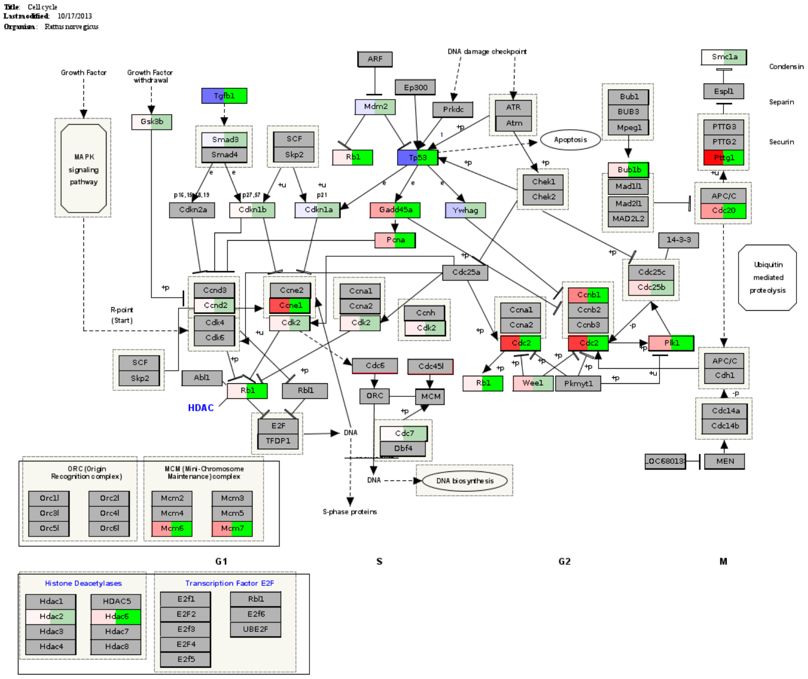

Figure 3: Cell cycle.png
